# Grignard Reagent-Catalyzed Hydroboration of Esters, Nitriles, and Imines

**DOI:** 10.3390/molecules28207090

**Published:** 2023-10-14

**Authors:** Hyun Ji Han, Suh Youn Park, So Eun Jeon, Jae Seok Kwak, Ji Hye Lee, Ashok Kumar Jaladi, Hyonseok Hwang, Duk Keun An

**Affiliations:** Department of Chemistry, Institute for Molecular Science and Fusion Technology, Kangwon National University, Chuncheon 24341, Republic of Korea; hj893456@naver.com (H.J.H.); qkrtjdbs98@naver.com (S.Y.P.); thdms2347@naver.com (S.E.J.); newwotjrbjel@naver.com (J.S.K.); jhlee81@kangwon.ac.kr (J.H.L.); jaladiashok05@gmail.com (A.K.J.); hhwang@kangwon.ac.kr (H.H.)

**Keywords:** hydroboration, esters, nitriles, imines, Grignard reagents

## Abstract

The reduction in esters, nitriles, and imines requires harsh conditions (highly reactive reagents, high temperatures, and pressures) or complex metal-ligand catalytic systems. Catalysts comprising earth-abundant and less toxic elements are desirable from the perspective of green chemistry. In this study, we developed a green hydroboration protocol for the reduction in esters, nitriles, and imines at room temperature (25 °C) using pinacolborane as the reducing agent and a commercially available Grignard reagent as the catalyst. Screening of various alkyl magnesium halides revealed MeMgCl as the optimal catalyst for the reduction. The hydroboration and subsequent hydrolysis of various esters yielded corresponding alcohols over a short reaction time (~0.5 h). The hydroboration of nitriles and imines produced various primary and secondary amines in excellent yields. Chemoselective reduction and density functional theory calculations are also performed. The proposed green hydroboration protocol eliminates the requirements for complex ligand systems and elevated temperatures, providing an effective method for the reduction in esters, nitriles, and imines at room temperature.

## 1. Introduction

The reduction in esters to valuable functionalized alcohols, which are used as starting materials or solvents, is a common reaction in organic synthesis. These alcohols are typically used for synthesizing bioactive molecules and agrochemicals, as well as for further functional group transformations [1]. However, owing to electronic and steric reasons, the transformation of esters into alcohols is relatively challenging compared with the corresponding reduction in aldehydes and ketones (i.e., aldehyde > ketone > ester). In particular, the conversion of esters to alcohols in the presence of other reducible groups requires additional steps. Highly reactive hydride reagents such as LiAlH_4_ or LiBH_4_ are commonly used for the conversion of esters to alcohols; however, these reactions afford low yields of alcohols and are not selective [2,3,4]. In addition, the pressurized hydrogenation reaction requires high pressures and temperatures which limit functional group tolerance [5]. To address these issues, catalyzed hydroelementation reactions, namely hydrosilylation and hydroboration reactions, have been developed using different catalytic systems, and the hydrosilylation of esters has previously been achieved [6,7,8,9,10,11,12]. Hydroboration of esters using a mild reagent, namely pinacolborane (HBpin), as the reducing agent is convenient to handle and forms stable borylated intermediates [13,14,15,16,17,18,19,20,21,22,23,24,25,26,27,28].

Similar to alcohols, amines are ubiquitous in natural compounds and are important building blocks in the synthesis of drugs, agrochemicals, coupling partners, dyes, and ligands for metal complexes [29,30,31]. Hence, amine synthesis is of considerable interest to researchers in industry and academia. In this context, the reduction in nitriles and imines via hydroboration is a straightforward and convenient method to obtain amines in good yields [32,33,34,35,36,37].

In the last decade, catalytic hydroboration reactions have been developed for polar (e.g., carbonyl-containing compounds) and nonpolar unsaturated substrates (e.g., alkenes and alkynes) with catalysts based on transition, main-group, and f-block metals [38,39,40,41,42,43,44,45,46]. However, the use of less toxic and earth-abundant elements to replace expensive and toxic metal catalysts is desirable for developing sustainable and environmentally friendly protocols. Alkaline earth metals are suitable for hydroboration because of their low cost, easy accessibility, and eco-friendliness [47]. For example, magnesium-based catalysts are known to be extremely reactive because of their high nucleophilicity and Brønsted-basic character. Consequently, magnesium-catalyzed hydroboration reactions have recently been investigated [48]. Hill et al. [49] used a β-diketiminate *n*-butylmagnesium complex as the catalyst to dearomatize pyridines in the reduction in methyl nicotinate using HBpin. Sadow et al. used a To^M^MgMe [To^M^ = tris(4,4-dimethyl-2-oxazolinyl)phenylborate (ToMMgMe] catalyst for ester hydroboration [13]. More recently, Ma et al. [14], Nembenna et al. [15], and Okuda et al. [16] used Mg complexes to catalyze hydroboration of esters. Hill et al. used a β-diketiminato *n*-butylmagnesium complex in the hydroboration of nitriles [50]. Furthermore, Ma et al. used a series of unsymmetrical β-diketiminate Mg(I) complexes for nitrile hydroboration [51].

**Figure 1:**
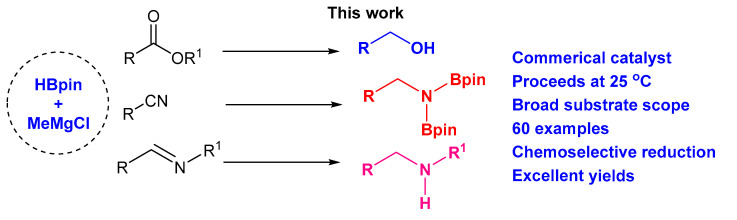
Grignard reagent-catalyzed hydroboration of esters, nitriles, and imines.

In this context, we believe that a more economic and robust protocol without complex ligand systems is urgently required for the hydroboration of esters, nitriles, and imines. Grignard reagents are known to be effective synthetic partners for their tremendous applicability in numerous organic reactions, such as C-C cross coupling reactions to increase the carbon–carbon chain, and as alkylating reagents for carbonyl electrophiles etc. Ma et al. used Grignard reagents in the hydroboration of aldehydes and ketones and obtained good conversions [52]. An et al. also observed good results for the hydroboration of esters and carbonyls using magnesium-based catalysts that are synthesized from Grignard reagents [53]. Considering the findings of Rueping et al. [54,55] and Ma et al. [52], we aimed to identify the scope of readily available Mg reagents as catalysts in the reduction in C=O, C≡N, and C=N bonds (Figure 1).

**Scheme 1:**
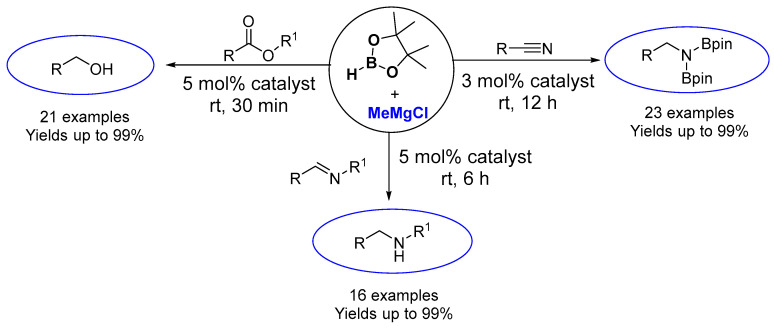
Hydroboration of esters, nitriles, and imines using Grignard reagents.

In this study, we developed Grignard reagent-catalyzed hydroboration protocols for esters, nitriles, and imines using HBpin at room temperature (25 °C). Subsequently, we investigated the Grignard reagent-catalyzed chemoselective hydroboration of esters in substrates comprising both esters and reducible groups such as nitriles, alkenes, and alkynes, and performed density functional theory (DFT) calculations to elucidate the mechanism of catalytic hydroboration (Scheme 1).

## 2. Results and Discussion

Initially, the hydroboration of ethyl benzoate ester with HBpin was investigated using various alkyl magnesium halides at room temperature for 30 min. The ester hydroboration proceeded smoothly (99% conversion) with 5 mol% loading of methyl magnesium chloride and bromide catalysts (Table 1, entries 1 and 2). However, the conversion rate for methyl magnesium iodide was significantly lower than that of entry 1 (75%; Table 1, entry 3). In addition, *n*-butyl and *tert*-butyl magnesium chlorides provided >90% conversions (Table 1, entries 4 and 5), whereas *iso*-propyl and phenyl magnesium chlorides afforded 82% and 89% conversions, respectively (Table 1, entries 6 and 7). Hence, methyl magnesium chloride (MeMgCl) was selected for the further evaluation of reaction conditions.

First, we determined a suitable solvent and catalyst concentration for the reaction (Tables S1 and S2). A 0.5 M MeMgCl solution prepared in dry THF afforded the highest conversion. Next, the catalyst loading and the amount of HBpin required for the reaction were optimized (Table 2). A decrease in the HBpin content from 2.5 to 2.0 equiv. decreased the conversion from 99% to 94% (entries 1–4). Similarly, a reduction in the catalyst loading by 1 mol% (i.e., to 4 and 3 mol%) led to an approximate 20% decrease in the conversion (entries 5 and 6). Therefore, the optimal conditions for the ester hydroboration reaction were 5 mol% catalyst loading (0.5 M in THF), 2.5 equiv. of HBpin, a reaction time of 30 min, and a reaction temperature of 25 °C (entry 4).

Subsequently, the substrate scope of ester hydroboration under the optimized conditions was examined using different aromatic and aliphatic esters (Table 3). Ethyl isopropyloxybenzoate (**1a**′) and ethyl benzoate (**1a**) afforded the corresponding alcohol in 99% yields in 30 min. Similarly, bulky *tert*-butoxy benzoate (**1a**″) afforded a 99% product yield. An electron-donating substituent on the ester slightly increased the reaction time required to achieve full conversion (i.e., 1 h for *para*-methyl- and *para*-methoxy-containing esters; **1c**–**d**), whereas the corresponding *ortho*-methyl-substituted ester (**1b**) afforded the desired product in 99% yield within 30 min. Electron-deficient *para*-fluoro, bromo, and iodo substituents (**1e**, **1h**, and **1i**) furnished the corresponding products in excellent yields (99%) in 30 min, whereas *para*-chloro and nitro substituents (**1f** and **1j**) required 60 min to obtain comparable product yields. Similarly, conjugated ester ethyl cinnamate (**1l**) as well as the aliphatic esters ethyl hexanoate (**1m**) and isopropyl hexanoate (**1m**′) afforded good yields. The methyl cyclohexanecarboxylate (**1n**), ethyl 4-chlorobutanoate (**1p**) afforded corresponding products in 98, 99% within 3 h. Ethyl 3-methylbut-2-enoate took 24 h to furnish corresponding alcohol (**1o**, 90%). The homologated ethylbenzoate, ethyl 3-(4-bromophenyl)propanoate (**1q**), and benzyloxy substituted ester, methyl 4-(benzyloxy)butanoate (**1r**) afforded corresponding products in 99% within 12 h reaction time.

Next, we explored the hydroboration of nitriles using various Grignard reagents. The hydroboration of benzonitrile using the simple Grignard reagent MeMgCl yielded 99% conversion within 12 h (Table 4, entry 1). MeMgBr and MeMgI afforded moderate conversions (58% and 57%, respectively; entries 2 and 3). However, quantitative conversion was achieved using isopropyl and *tert*-butyl magnesium chloride (entries 5 and 6). A 94% conversion was achieved with *n*-butyl magnesium chloride (entry 4). PhMgCl was more effective than PhMgBr, affording 92% and 70% conversions, respectively (entries 7 and 8). Hence, MeMgCl was selected as the optimal catalyst for further optimization of reaction conditions.

First, a suitable solvent and catalyst concentration were determined for the reaction (Tables S3 and S4). A 0.5 M MeMgCl solution in dry THF afforded the highest conversion. Hence, both the catalyst loading and amount of HBpin required for the reaction were optimized using the 0.5 M MeMgCl solution in dry THF. Three equivalents of HBpin and 3 mol% of catalyst afforded 97% conversion in 6 h (Table 5, entry 1). The conversion improved with increasing reaction time up to 12 h (entry 2), whereas it dramatically decreased when the catalyst loading was reduced to 2.0 mol% (entry 3). Moreover, 3 mol% of the catalyst and 2.5 equivalents of HBpin afforded a 99% conversion in 12 h (entry 4). In contrast, the conversion decreased when the catalyst loading was reduced to 2.0 mol% at the same reaction time (12 h, 83%, entry 5). However, the conversion increased to 99% with an increase in the reaction time (entry 6). The conversion slightly reduced upon decreasing the amount of HBpin from 2.5 to 2.2 equivalents (entry 7). Finally, the optimal conditions for nitrile hydroboration were 3 mol% catalyst loading (0.5 M in THF), 2.5 equiv. of HBpin, a reaction time of 12 h, and a reaction temperature of 25 °C (entry 4).

Next, we evaluated the substrate scope of nitrile hydroboration using optimized conditions and a variety of nitriles, including aromatic, hetero-aromatic, and aliphatic nitriles with electron-excess and -deficient substitutions. Aromatic substrates with electron-deficient groups, such as fluoro (**3g**), chloro (**3h**), bromo (**3i**), iodo (**3j**), and trifluoromethyl (**3k**), showed high reactivities to afford the corresponding double hydroborated products (**4g**, **4h**, **4h**, **4i**, **4j**, and **4k**) with 3 mol% catalyst within 12 h compared with nitriles with excess electrons [2-methyl (**3b**), 3-methyl (**3c**), and *N,N*-dimethyl (**3f**)]. In contrast, 4-nitro-benzonitrile (**3l**) afforded only a 90% yield (**4l**) in 24 h. The 2-pyridyl benzo nitrile (**3m**) furnished the corresponding product in moderate yields (**4m**), while 4-pyridyl benzonitrile (**3n**) underwent hydroboration at 80 °C to afford a 99% yield (**4n**). Moreover, furan-2-carbonitrile (**3o**) required 24 h to afford 99% yield (**4o**), while thiophene-2-carbonitrile (**3p**) furnished a 92% yield (**4p**) with 5 mol% catalyst loading. In addition, 1-Naphthonitrile (**3q**) and 2-naphthonitrile (**3r**) afforded 99% (**4q**) and 97% (**4r**) yields at 5 and 3 mol% catalyst loadings, respectively. In contrast, 2-phenylacetonitrile (**3s**) afforded a comparatively moderate yield (**4s**; 89%) with 10 mol% catalyst loading. Aliphatic nitriles (**3t**–**w**) were also amenable to this hydroboration affording the corresponding dihydroborated amines with a 10 mol% catalyst loading. Cyclic cyclohexanecarbonitrile (**3t**) afforded the product in a 66% yield (**4t**). Open-chain hexanenitrile (**3u**) and dodecanenitrile (**3v**) afforded the corresponding products in 95% (**4u**) and 99% (**4v**) yields, respectively. The 2-methoxyacetonitrile (**3w**) produced the di-hydroborated product (**4w**) in a 99% yield (Table 6).

The same method was then extended to imines. Even though imine hydroboration afforded good yields with the optimal conditions established for ester hydroboration, we further optimized the reaction conditions (Table 7).

In terms of the catalyst, methyl- and *tert*-butyl magnesium chlorides provided excellent conversions (99%, entries 1 and 6), whereas BuMgCl afforded 97% conversion (entry 5). In addition, MeMgBr and MeMgI afforded conversions of 91% and 78%, respectively (entries 2 and 3), iPrMgCl and PhMgCl afforded 83% conversions (entries 4 and 7). Subsequent investigations of the catalyst loading, HBpin content, and reaction time (Table 7, Tables S5 and S6) revealed the optimal conditions as 5 mol% MeMgCl, 1.5 equiv. HBpin, and a reaction time of 6 h. Although these conditions afforded the 99% conversion, the conversion was considerably reduced upon decreasing the catalyst loading and reaction time.

Subsequently, the substrate scope of imine hydroboration was investigated using the optimized reaction conditions (Table 8, entry 2) and a range of imines (aldimines and ketimines, Table 9) containing electron-rich [methyl (**5b**) and methoxy (**5c**)] and -deficient [fluoro (**5g**), chloro (**5h**), bromo (**5i**), and trifluoro (**5j**)] substituents. These substrates were amenable to hydroboration at room temperature (25 °C), affording the corresponding products (**6b**–**j**) in excellent yields (99% isolated yields from aldemines). Notably, polyaromatic substrates, including (*E*)-1-(naphthalen-2-yl)-*N*-phenylmethanimine (**5k**) and (*E*)-*N*-phenyl-1-(pyren-1-yl)methanimine (**5l**), as well as the heteroaromatic imine (*E*)-*N*-(4-bromophenyl)-1-(thiophen-3-yl)methanimine (**5m**), afforded the desired products in 99% yields (**6k**–**m**). Ketimines (**5n**–**p**) also underwent smooth hydroboration, affording the corresponding amines in good yields (72–96%, Table 9).

**Scheme 2:**
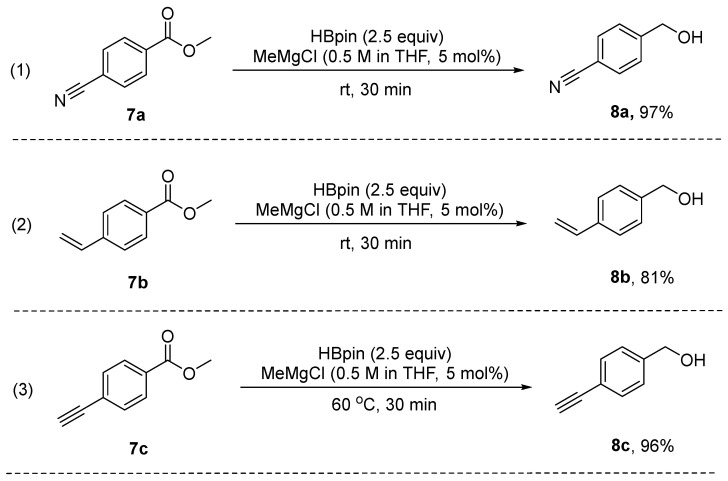
Chemoselective hydroboration; ester and nitrile (**1**), ester and alkene (**2**), ester and alkyne (**3**).

Finally, the efficiency of the catalyst was investigated using chemoselective hydroboration. Intramolecular chemoselective hydroboration was performed using esters containing other reducible functional groups, nitriles, alkenes, and alkynes. The ester group was selectively reduced to afford the corresponding alcohols in good yields (Scheme 2).

**Scheme 3:**
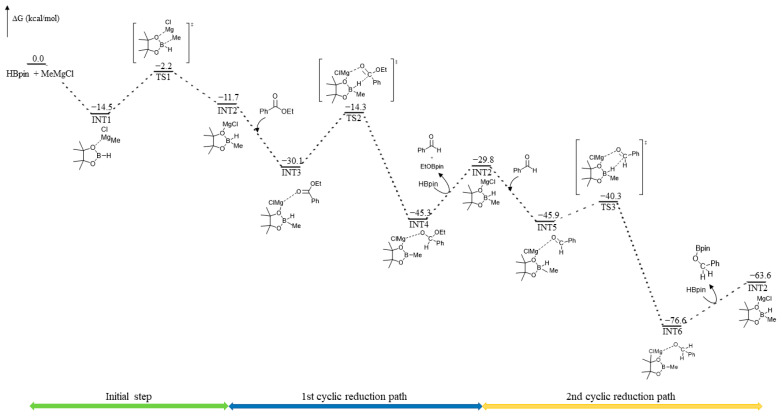
Free energy profile (kcal/mol) for the MeMgCl-catalyzed hydroboration of ethyl benzoate.

**Scheme 4:**
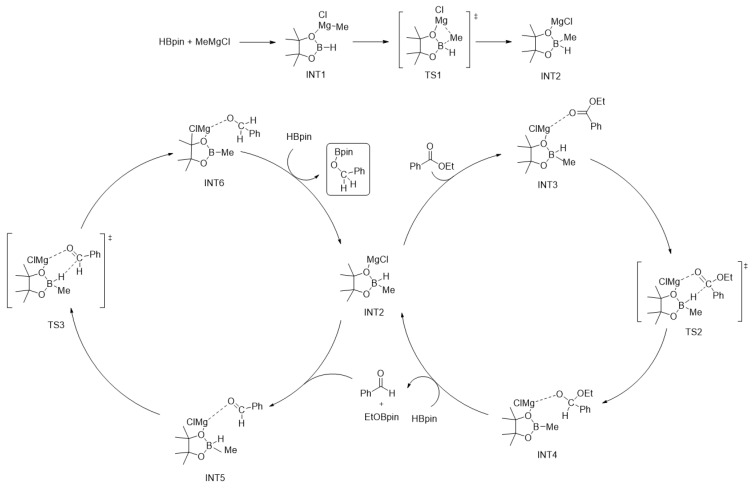
Plausible mechanism for the ester hydroboration in the presence of a Grignard reagent catalyst (MeMgCl) and the hydroboration reagent (HBpin) based on the free energy profile shown in Scheme 3.

The reaction pathway for MeMgCl-catalyzed hydroboration of benzoate was investigated using DFT calculations at the M06-2X/6-31G(d,p) level of theory [56]. A schematic of the free energy profile for the reaction pathway is shown in Scheme 3. The reaction is divided into two catalytic cycles. The initial step is an exergonic reaction (−14.5 kcal/mol) which involves the binding of MeMgCl to HBpin to form intermediate INT1. Subsequently, INT1 undergoes intramolecular rearrangement, where the Me group migrates from Mg to the B atom via the cyclic transition state TS1, which has an energy barrier of 12.3 kcal/mol relative to INT1, affording the zwitterionic intermediate INT2. The first catalytic cyclic is initiated by the approach of benzoate toward INT2, another exergonic reaction (18.4 kcal/mol), yielding INT3. Then, INT3 is rearranged to INT4 (exergonic by 15.2 kcal/mol) via the six-membered ring transition state TS2, with an energy barrier of 15.8 kcal/mol. Another HBpin molecule binds to INT4, regenerating INT2, producing benzaldehyde and EtOBpin, and thus completing the first catalytic cycle. In the second catalytic cycle, INT2 reacts with benzaldehyde, forming INT5 (exergonic by 16.1 kcal/mol). INT5 rearranges into INT6 (exergonic by 30.7 kcal/mol) via the six-membered ring transition state TS3 with an energy barrier of 5.6 kcal/mol. Another HBpin molecule reacts with INT6, regenerating INT2 and producing PhCH_2_OBpin through a ligand exchange reaction. Based on the free energy profile (Scheme 3), we propose a plausible mechanism for the hydroboration of esters using a Grignard reagent as the catalyst (Scheme 4).

## 3. Materials and Methods

### 3.1. General Information

All glassware used was dried thoroughly in an oven, assembled hot, and cooled under a stream of dry nitrogen prior to use. All reactions and manipulations of air- and moisture-sensitive materials were carried out using standard techniques for the handling of such materials. All chemicals were commercial products of the highest purity which were further purified before use by using standard methods. HBpin, aldehydes, ketones, and alkenes were purchased from Aldrich Chemical Company, Alfa Aesar, and Tokyo Chemical Industry Company (TCI). ^1^H NMR spectra were measured at 400 MHz with CDCl_3_ as a solvent at ambient temperature unless otherwise indicated and the chemical shifts were recorded in parts per million downfield from tetramethylsilane (δ = 0 ppm) or based on residual CDCl_3_ (δ = 7.26 ppm) as the internal standard. The coupling constants (*J*) are reported in hertz. Analytical thin-layer chromatography (TLC) was performed on glass precoated with silica gel (Merck, Rahway, NJ, USA, silica gel 60 F_254_). Column chromatography was carried out using 70–230 mesh silica gel (Merck) at normal pressure. GC analyses were performed on a Younglin Acme 6100M and 6500 GC FID chromatography, using an HP-5 capillary column (30 m). All GC yields were determined with the use of naphthalene as the internal standard and the authentic sample.

### 3.2. General Procedure

#### 3.2.1. Catalytic Hydroboration of Ester

A 10 mL test tube was charged with a magnet, closed with septum, and flushed with argon. To this, 0.0751 g (1.0 eq) of ethyl benzoate, 0.18 mL (2.5 eq) of pinacolborane, and 0.05 mL (5 mol%) of 0.5 M methyl magnesium chloride were added at room temperature. Contents were stirred for the given time (mentioned in Table 3) at the same temperature. After completion of the reaction (analyzed by GC), the reaction was terminated by the addition of water (1 mL). The crude mixture was hydrolyzed to alcohol by adding 1 *N* aqueous NaOH solution (1 mL). The resulting mixture was extracted with diethyl ether, washed with brine, and the combined organic layers were dried over MgSO_4_. After filtration, the solvents were evaporated under reduced pressure and the mixed residue was purified by silica gel column chromatography.

#### 3.2.2. Catalytic Hydroboration of Nitrile

A 10 mL test tube was charged with a magnet, closed with septum, and flushed with argon. To this, 0.0515 g (1.0 eq) of benzonitrile, 0.18 mL (2.5 eq) of pinacolborane, and 0.03 mL (3 mol%) of 0.5 M methyl magnesium chloride were added at room temperature. The contents were stirred for 12 h at the same temperature. After completion of the reaction (analyzed by GC), the solvents were evaporated under reduced pressure. The crude mixture was analyzed by NMR using 1,3,5-trimethoxybenzene as an internal standard.

#### 3.2.3. Catalytic Hydroboration of Imine

A 10 mL test tube was charged with a magnet, closed with septum, and flushed with argon. To this, 0.0906 g (1.0 eq) of benzylideneaniline, 0.11 mL (1.5 eq) of pinacolborane, and 0.05 mL (5 mol%) of 0.5 M methyl magnesium chloride were added at room temperature. The contents were stirred for 6 h at the same temperature. After completion of the reaction (analyzed by GC), the reaction was terminated by the addition of water (1 mL). The crude mixture was extracted with ethyl acetate and the combined organic layers were dried over MgSO_4_. After filtration, the solvents were evaporated under reduced pressure and the mixed residue was purified by silica gel column chromatography.

### 3.3. Characterization of Products

Benzyl alcohol (**2a**) [57]: Colorless oil (53 mg, 99% yield); 1H NMR (400 MHz, Chloroform-d) δ 7.36 (d, *J* = 4.3 Hz, 4H), 7.33–7.26 (m, 1H), 4.66 (d, *J* = 2.2 Hz, 2H), 2.06–1.86 (m, 1H); 13C NMR (100 MHz, Chloroform-d) δ 140.96, 128.67, 127.76, 127.11, 65.42.
2-Metylbenzyl alcohol (**2b**) [57]: White solid (61 mg, 99% yield); 1H NMR (400 MHz, Chloroform-d) δ 7.34 (dd, *J* = 6.3, 2.6 Hz, 1H), 7.26–7.14 (m, 3H), 4.66 (s, 2H), 2.35 (s, 3H), 1.97–1.87 (m, 1H); 13C NMR (100 MHz, Chloroform-d) δ 138.80, 136.20, 130.42, 127.88, 127.63, 126.16, 63.54, 18.75.
4-Metylbenzyl alcohol (**2c**) [57]: White solid (61 mg, 99% yield); 1H NMR (400 MHz, Chloroform-d) δ 7.24 (d, *J* = 8.0 Hz, 2H), 7.17 (d, *J* = 7.8 Hz, 2H), 4.62 (d, *J* = 2.6 Hz, 2H), 2.35 (s, 3H), 1.98–1.76 (m, 1H); 13C NMR (100 MHz, Chloroform-d) δ 138.02, 137.48, 129.34, 127.23, 65.30, 21.26.
4-Methoxybenzyl alcohol (**2d**) [57]: White solid (69 mg, 99% yield);1H NMR (400 MHz, Chloroform-d) δ 7.27 (d, *J* = 8.5 Hz, 2H), 6.88 (d, *J* = 8.6 Hz, 2H), 4.59 (s, 2H), 3.79 (s, 3H), 1.90–1.76 (m, 1H); 13C NMR (100 MHz, Chloroform-d) δ 159.27, 133.22, 128.76, 114.04, 65.08, 55.39.
4-Fluorobenzyl alcohol (**2e**) [57]: Colorless oil (54 mg, 99% yield); 1H NMR (400 MHz, Chloroform-d) δ 7.37–7.28 (m, 2H), 7.07–6.99 (m, 2H), 4.64 (s, 2H), 1.86 (s, 1H); 13C NMR (100 MHz, Chloroform-d) δ 162.40 (d, *J* C-F = 245.5 Hz), 136.66 (d, *J* C-F = 3.2 Hz), 128.86 (d, *J* C-F = 8.1 Hz), 115.48 (d, *J* C-F = 21.5 Hz), 64.72.
4-Chlorobenzyl alcohol (**2f**) [7]: White solid (71 mg, 99% yield); 1H NMR (400 MHz, Chloroform-d) δ 7.29 (q, *J* = 8.6 Hz, 4H), 4.64 (s, 2H), 1.94 (s, 1H); 13C NMR (100 MHz, Chloroform-d) δ 139.33, 133.44, 128.77, 128.38, 64.62.
2-Bromobenzyl alcohol (**2g**) [58]: White solid (94 mg, 99% yield); 1H NMR (400 MHz, Chloroform-d) δ 7.54 (d, *J* = 7.9 Hz, 1H), 7.47 (d, *J* = 8.4 Hz, 1H), 7.32 (t, *J* = 7.5 Hz, 1H), 7.16 (td, *J* = 7.7, 1.8 Hz, 1H), 4.74 (s, 2H), 2.01 (s, 1H); 13C NMR (100 MHz, Chloroform-d) δ 139.80, 132.70, 129.24, 129.02, 127.77, 122.69, 65.20.
4-Bromobenzyl alcohol (**2h**) [7]: White solid (93 mg, 99% yield); 1H NMR (400 MHz, Chloroform-d) δ 7.48 (d, *J* = 8.3 Hz, 2H), 7.23 (s, 2H), 4.65 (d, *J* = 5.9 Hz, 2H), 1.72–1.59 (m, 1H); 13C NMR (100 MHz, Chloroform-d) δ 139.83, 131.71, 128.69, 121.53, 64.58.
4-Iodobenzyl alcohol (**2i**) [58]: White solid (116 mg, 99% yield); 1H NMR (400 MHz, Chloroform-d) δ 7.67 (d, *J* = 8.3 Hz, 2H), 7.09 (d, *J* = 8.1 Hz, 2H), 4.62 (s, 2H), 1.80 (s, 1H); 13C NMR (100 MHz, Chloroform-d) δ 140.52, 137.69, 128.91, 93.11, 64.74.
4-Nitrobenzyl alcohol (**2j**) [7]: Pale yellow solid (76 mg, 99% yield); 1H NMR (400 MHz, Chloroform-d) δ 8.19 (d, *J* = 8.7 Hz, 2H), 7.51 (d, *J* = 8.8 Hz, 2H), 4.82 (s, 2H), 2.07 (s, 1H); 13C NMR (100 MHz, Chloroform-d) δ 148.32, 147.33, 127.10, 123.83, 64.08.
2-Naphthalenemethanol (**2k**) [57]: White solid (75 mg, 99% yield); 1H NMR (400 MHz, Chloroform-d) δ 7.89–7.78 (m, 4H), 7.52–7.44 (m, 3H), 4.85 (s, 2H), 1.74 (s, 1H); 13C NMR (100 MHz, Chloroform-d) δ 138.39, 133.46, 133.04, 128.46, 127.99, 127.82, 126.31, 126.02, 125.55, 125.27, 65.62.
Cinnamyl alcohol (**2l**) [59]: White solid (67 mg, 99% yield); 1H NMR (400 MHz, Chloroform-d) δ 7.38 (d, *J* = 7.3 Hz, 2H), 7.32 (t, *J* = 7.4 Hz, 2H), 7.24 (t, *J* = 7.2 Hz, 1H), 6.61 (d, *J* = 15.9 Hz, 1H), 6.36 (dt, *J* = 15.9, 5.7 Hz, 1H), 4.31 (dd, *J* = 5.7, 1.6 Hz, 2H), 1.92 (s, 1H); 13C NMR (100 MHz, Chloroform-d) δ 136.78, 131.19, 128.72, 128.62, 127.81, 126.58, 63.77.
Hexanol (**2m**) [59]: Colorless oil (50 mg, 99% yield); 1H NMR (400 MHz, Chloroform-d) δ 3.62 (tq, *J* = 6.7, 1.4 Hz, 2H), 1.63–1.52 (m, 2H), 1.49–1.39 (m, 1H), 1.37–1.27 (m, 6H), 0.87 (td, *J* = 6.9, 2.2 Hz, 3H); 13C NMR (100 MHz, Chloroform-d) δ 63.16, 32.84, 31.72, 25.50, 22.72, 14.12.
Cyclohexylmethanol (**2n**) [59]: Colorless oil (57 mg, 99% yield); 1H NMR (400 MHz, Chloroform-d) δ 3.42 (d, *J* = 6.7 Hz, 2H), 1.76–1.63 (m, 5H), 1.52–1.42 (m, 1H), 1.42–1.30 (m, 1H), 1.20 (dq, *J* = 24.5, 12.1, 11.7 Hz, 3H), 0.91 (q, *J* = 11.2, 10.5 Hz, 2H); 13C NMR (100 MHz, Chloroform-d) δ 68.86, 40.57, 29.64, 26.67, 25.92.
3-Methylbut-2-en-1-ol (**2o**) [60]: Colorless oil (38mg, 90% yield); 1H NMR (400 MHz, Chloroform-d) δ 5.40 (t, *J* = 7.3 Hz, 1H), 4.12 (d, *J* = 7.1 Hz, 2H), 1.73 (s, 3H), 1.67 (s, 3H), 1.16 (dt, *J* = 21.1, 8.5 Hz, 1H); 13C NMR (100 MHz, Chloroform-d) δ 123.67, 59.48, 25.84, 17.91.
4-Chlorobutan-1-ol (**2p**) [61]: 1H NMR (400 MHz, Chloroform-d) δ 3.66 (t, *J* = 6.3 Hz, 2H), 3.56 (t, *J* = 6.6 Hz, 2H), 1.86 (dt, *J* = 14.5, 6.7 Hz, 2H), 1.74–1.67 (m, 2H), 1.65 (s, 1H); 13C NMR (100 MHz, Chloroform-d) δ 62.13, 45.01, 29.99, 29.10.
3-(4-Bromophenyl)propan-1-ol (**2q**) [62]: Colorless oil (107 mg, 99% yield); 1H NMR (400 MHz, Chloroform-d) δ 7.39 (d, *J* = 8.3 Hz, 2H), 7.06 (d, *J* = 8.0 Hz, 2H), 3.65 (t, *J* = 6.4 Hz, 2H), 2.71–2.61 (m, 2H), 1.85 (dt, *J* = 13.6, 6.5 Hz, 2H), 1.29 (s, 1H); 13C NMR (100 MHz, Chloroform-d) δ 140.85, 131.53, 130.30, 119.67, 62.09, 34.09, 31.53.
4-(benzyloxy)butan-1-ol (**2r**) [63]: Colorless oil (89 mg, 99% yield); 1H NMR (400 MHz, Chloroform-d) δ 7.37–7.25 (m, 5H), 4.51 (s, 2H), 3.64 (q, *J* = 5.6 Hz, 2H), 3.51 (t, *J* = 5.6 Hz, 2H), 2.12 (t, *J* = 5.7 Hz, 1H), 1.69 (dq, *J* = 11.9, 6.2 Hz, 4H); 13C NMR (100 MHz, Chloroform-d) δ 138.21, 128.53, 127.83, 73.17, 70.43, 62.86, 30.29, 26.82.
*N*-Benzyl-4,4,5,5-tetramethyl-*N*-(4,4,5,5-tetramethyl-1,3,2-dioxaborolan-2-yl)-1,3,2 dioxaborolan-2-amine (**4a**) [64]: 1H NMR (400 MHz, Benzene-d6) δ 7.53 (d, *J* = 7.6 Hz, 2H), 7.19 (t, *J* = 7.6 Hz, 2H), 7.06 (t, *J* = 7.6 Hz, 1H), 4.56 (s, 2H), 0.97 (s, 24H); 13C NMR (100 MHz, Benzene-d6) δ 143.49, 128.00, 126.37, 82.29, 47.62, 24.42.
4,4,5,5-Tetramethyl-*N*-(2-methylbenzyl)-*N*-(4,4,5,5-tetramethyl-1,3,2-dioxaborolan-2-yl)-1,3,2-dioxaborolan-2-amine (**4b**) [64]: 1H NMR (400 MHz, Benzene-d6) δ 7.61 (d, *J* = 7.7 Hz, 1H), 7.20 (t, *J* = 7.5 Hz, 1H), 7.02 (t, *J* = 7.4 Hz, 1H), 6.93 (d, *J* = 7.4 Hz, 1H), 4.56 (s, 2H), 2.07 (s, 3H), 0.97 (s, 24H); 13C NMR (100 MHz, Benzene-d6) δ 140.89, 135.06, 129.86, 125.90, 125.72, 125.33, 82.29, 45.14, 24.35, 18.75.
4,4,5,5-Tetramethyl-*N*-(3-methylbenzyl)-*N*-(1,4,4,5,5-pentamethyl-1,3,2-dioxaborolan-2-yl)-1,3,2-dioxaborolan-2-amine (**4c**) [65]: 1H NMR (400 MHz, Benzene-d6) δ 7.33 (d, *J* = 7.7 Hz, 1H), 7.29 (s, 1H), 7.13 (d, *J* = 7.5 Hz, 1H), 6.88 (d, *J* = 7.5 Hz, 1H), 4.49 (s, 2H), 2.12 (s, 3H), 0.96 (s, 24H); 13C NMR (100 MHz, Benzene-d6) δ 143.33, 137.12, 128.64, 127.05, 124.74, 82.21, 47.48, 24.42, 21.22.
4,4,5,5-Tetramethyl-*N*-(4-methylbenzyl)-*N*-(4,4,5,5-tetramethyl-1,3,2-dioxaborolan-2-yl)-1,3,2-dioxaborolan-2-amine (**4d**) [64]: 1H NMR (400 MHz, Benzene-d6) δ 7.46 (d, *J* = 7.5 Hz, 2H), 7.01 (d, *J* = 7.7 Hz, 2H), 4.54 (s, 2H), 2.09 (s, 3H), 0.98 (s, 24H); 13C NMR (100 MHz, Benzene-d6) δ 140.58, 135.46, 128.78, 82.23, 47.30, 24.44, 20.85.
*N*-(4-methoxybenzyl)-4,4,5,5-tetramethyl-*N*-(4,4,5,5-tetramethyl-1,3,2-dioxaborolan-2-yl)-1,3,2-dioxaborolan-2-amine (**4e**) [64]: 1H NMR (400 MHz, Benzene-d6) δ 7.47 (d, *J* = 8.5 Hz, 2H), 6.79 (d, *J* = 8.4 Hz, 2H), 4.48 (s, 2H), 3.30 (s, 3H), 0.97 (s, 24H); 13C NMR (100 MHz, Benzene-d6) δ 158.66, 135.71, 129.14, 113.52, 82.20, 54.45, 46.96, 24.45.
*N*-(4-(dimethylamino)benzyl)-4,4,5,5-tetramethyl-*N*-(4,4,5,5-tetramethyl-1,3,2-dioxaborolan-2-yl)-1,3,2-dioxaborolan-2-amine) (**4f**) [64]: 1H NMR (400 MHz, Benzene-d6) δ 7.57 (d, *J* = 8.5 Hz, 2H), 6.65 (d, *J* = 8.6 Hz, 2H), 4.58 (s, 2H), 2.50 (s, 6H), 1.01 (s, 24H); 13C NMR (100 MHz, Benzene-d6) δ 149.65, 132.05, 128.99, 112.72, 82.16, 47.08, 40.29, 24.51.
*N*-(4-fluorobenzyl)-4,4,5,5-tetramethyl-*N*-(4,4,5,5-tetramethyl-1,3,2-dioxaborolan-2-yl)-1,3,2-dioxaborolan-2-amine (**4g**) [64]: 1H NMR (400 MHz, Benzene-d6) δ 7.36 (dd, *J* = 8.5, 5.6 Hz, 2H), 6.83 (t, *J* = 8.7 Hz, 2H), 4.42 (s, 2H), 0.96 (s, 24H); 13C NMR (100 MHz, Benzene-d6) δ 161.95 (d, J C-F = 243.2 Hz), 139.24 (d, J C-F = 3.1 Hz), 129.50 (d, J C-F = 7.7 Hz), 114.71 (d, J C-F = 21.1 Hz).
*N*-(4-chlorobenzyl)-4,4,5,5-tetramethyl-*N*-(4,4,5,5-tetramethyl-1,3,2-dioxaborolan-2-yl)-1,3,2-dioxaborolan-2-amine (**4h**) [64]: 1H NMR (400 MHz, Benzene-d6) δ 7.29 (d, *J* = 8.1 Hz, 2H), 7.13 (d, *J* = 7.8 Hz, 2H), 4.39 (s, 2H), 0.96 (s, 24H); 13C NMR (100 MHz, Benzene-d6) δ 141.96, 129.26, 128.00, 82.40, 46.88, 24.39.
*N*-(4-bromobenzyl)-4,4,5,5-tetramethyl-*N*-(4,4,5,5-tetramethyl-1,3,2-dioxaborolan-2-yl)-1,3,2-S4 dioxaborolan-2-amine (**4i**) [64]: 1H NMR (400 MHz, Benzene-d6) δ 7.27 (d, *J* = 8.5 Hz, 2H), 7.22 (d, *J* = 6.8 Hz, 2H), 4.36 (s, 2H), 0.95 (s, 24H); 13C NMR (100 MHz, Benzene-d6) δ 142.42, 131.17, 129.63, 120.31, 82.40, 46.92, 24.39.
*N*-(4-iodobenzyl)-4,4,5,5-tetramethyl-*N*-(4,4,5,5-tetramethyl-1,3,2-dioxaborolan-2-yl)-1,3,2-dioxaborolan-2-amine (**4j**) [64]:1H NMR (400 MHz, Benzene-d6) δ 7.46 (d, *J* = 8.0 Hz, 2H), 7.09 (d, *J* = 7.8 Hz, 2H), 4.35 (s, 2H), 0.94 (s, 24H); 13C NMR (100 MHz, Benzene-d6) δ 143.04, 137.17, 129.89, 91.76, 82.39, 46.99, 24.41.
4,4,5,5-Tetramethyl-*N*-(4,4,5,5-tetramethyl-1,3,2-dioxaborolan-2-yl)-*N*-(4-(trifluoromethyl)benzyl)-1,3,2-dioxaborolan-2-amine (**4k**) [64]: 1H NMR (400 MHz, Benzene-d6) δ 7.36 (s, 4H), 4.43 (s, 2H), 0.95 (s, 24H); 13C NMR (100 MHz, Benzene-d6) δ 147.39, 129.05, 129.01, 128.73, 128.42, 126.30, 125.04, 125.00, 124.96, 124.92, 123.60, 82.47, 47.14, 24.35.
4,4,5,5-Tetramethyl-*N*-(4-nitrobenzyl)-*N*-(4,4,5,5-tetramethyl-1,3,2-dioxaborolan-2-yl)-1,3,2-dioxaborolan-2-amine (**4l**) [64]: 1H NMR (400 MHz, Benzene-d6) δ 7.85 (d, *J* = 8.7 Hz, 2H), 7.20 (d, *J* = 8.5 Hz, 2H), 4.30 (s, 2H), 0.94 (s, 24H); 13C NMR (100 MHz, Benzene-d6) δ 150.34, 146.83, 127.87, 123.45, 82.55, 46.99, 24.33.
4,4,5,5-Tetramethyl-*N*-(pyridin-2-ylmethyl)-*N*-(4,4,5,5-tetramethyl-1,3,2-dioxaborolan-2-yl)-1,3,2-dioxaborolan-2-amine (**4m**) [64]: 1H NMR (400 MHz, Benzene-d6) δ 8.42 (d, *J* = 5.0 Hz, 1H), 7.19–7.12 (m, 2H), 6.62–6.56 (m, 1H), 4.80 (s, 2H), 0.96 (s, 24H); 13C NMR (100 MHz, Benzene-d6) δ 162.62, 148.98, 135.39, 120.79, 119.48, 82.25, 49.61, 24.38.
4,4,5,5-Tetramethyl-*N*-(pyridin-4-ylmethyl)-*N*-(4,4,5,5-tetramethyl-1,3,2-dioxaborolan-2-yl)-1,3,2-dioxaborolan-2-amine (**4n**) [64]: 1H NMR (400 MHz, Benzene-d6) δ 8.55 (d, *J* = 5.8 Hz, 2H), 7.12 (d, *J* = 5.8 Hz, 2H), 4.35 (s, 2H), 0.94 (s, 24H); 13C NMR (100 MHz, Benzene-d6) δ 151.43, 149.91, 122.13, 82.52, 46.68, 24.33.
*N*-(Furan-2-ylmethyl)-4,4,5,5-tetramethyl-*N*-(4,4,5,5-tetramethyl-1,3,2-dioxaborolan-2-yl)-1,3,2-dioxaborolan-2-amine (**4o**) [64]: 1H NMR (400 MHz, Benzene-d6) δ 7.07 (s, 1H), 6.18 (d, *J* = 3.2 Hz, 1H), 6.12–6.05 (m, 1H), 4.47 (s, 2H), 0.96 (s, 24H); 13C NMR (100 MHz, Benzene-d6) δ 156.95, 140.98, 110.05, 105.43, 82.30, 40.97, 24.38.
4,4,5,5-Tetramethyl-*N*-(4,4,5,5-tetramethyl-1,3,2-dioxaborolan-2-yl)-*N*-(thiophen-2-ylmethyl)-1,3,2-dioxaborolan-2-amine (**4p**) [65]: 1H NMR (400 MHz, Benzene-d6) δ 7.05 (d, *J* = 3.5 Hz, 1H), 6.85 (d, *J* = 5.0 Hz, 1H), 6.74 (dd, *J* = 5.2, 3.5 Hz, 1H), 4.64 (s, 2H), 0.99 (s, 24H); 13C NMR (100 MHz, Benzene-d6) δ 147.02, 126.33, 124.94, 123.85, 82.47, 42.40, 24.48.
4,4,5,5-Tetramethyl-*N*-(naphthalen-1-ylmethyl)-*N*-(4,4,5,5-tetramethyl-1,3,2-dioxaborolan-2-yl)-1,3,2-dioxaborolan-2-amine (**4q**) [64]: 1H NMR (400 MHz, Benzene-d6) δ 8.09–8.05 (m, 1H), 7.75 (d, *J* = 7.2 Hz, 1H), 7.62–7.58 (m, 1H), 7.53 (d, *J* = 8.2 Hz, 1H), 7.35 (t, *J* = 7.7 Hz, 1H), 7.20–7.15 (m, 2H), 5.08 (s, 2H), 0.97 (s, 24H); 13C NMR (100 MHz, Benzene-d6) δ 138.51, 133.95, 131.59, 128.57, 126.88, 123.49, 123.03, 82.34, 45.12, 24.35.
4,4,5,5-Tetramethyl-*N*-(naphthalen-2-ylmethyl)-*N*-(4,4,5,5-tetramethyl-1,3,2-dioxaborolan-2-yl)-1,3,2-dioxaborolan-2-amine (**4r**) [66,67]: 1H NMR (400 MHz, Benzene-d6) δ 7.98 (s, 1H), 7.72–7.57 (m, 4H), 7.21 (pd, *J* = 6.9, 1.5 Hz, 2H), 4.72 (s, 2H), 0.98 (s, 24H); 13C NMR (100 MHz, Benzene-d6) δ 140.96, 133.93, 132.87, 126.63, 126.12, 125.74, 125.20, 82.63, 82.37, 47.74, 24.44.
4,4,5,5-Tetramethyl-*N*-phenethyl-*N*-(4,4,5,5-tetramethyl-1,3,2-dioxaborolan-2-yl)-1,3,2-dioxaborolan-2-amine (**4s**) [64]: 1H NMR (400 MHz, Benzene-d6) δ 7.23 (d, *J* = 7.5 Hz, 2H), 7.09 (d, *J* = 5.6 Hz, 2H), 7.00 (t, *J* = 7.5 Hz, 1H), 3.63 (t, *J* = 7.3 Hz, 2H), 2.95 (t, *J* = 7.3 Hz, 2H), 0.98 (s, 24H); 13C NMR (100 MHz, Benzene-d6) δ 140.48, 129.47, 128.19, 125.81, 82.00, 45.77, 39.95, 24.44.
*N*-(cyclohexylmethyl)-4,4,5,5-tetramethyl-*N*-(4,4,5,5-tetramethyl-1,3,2-dioxaborolan-2-yl)-1,3,2-dioxaborolan-2-amine (**4t**) [35]: 1H NMR (400 MHz, Benzene-d6) δ 3.27 (d, *J* = 7.4 Hz, 2H), 1.85 (d, *J* = 11.0 Hz, 2H), 1.76–1.63 (m, 4H), 1.54 (d, *J* = 1.3 Hz, 1H), 1.26–1.16 (m, 4H), 1.02 (s, 24H); 13C NMR (100 MHz, Benzene-d6) δ 81.94, 50.21, 40.64, 30.89, 26.95, 26.35, 24.45.
*N*-hexyl-4,4,5,5-tetramethyl-*N*-(4,4,5,5-tetramethyl-1,3,2-dioxaborolan-2-yl)-1,3,2-dioxaborolan-2-amine (**4u**) [64]: 1H NMR (400 MHz, Benzene-d6) δ 3.42 (t, *J* = 7.1 Hz, 2H), 1.70 (q, *J* = 7.8 Hz, 2H), 1.36–1.21 (m, 6H), 1.02 (s, 24H), 0.81 (t, *J* = 3.6 Hz, 3H); 13C NMR (100 MHz, Benzene-d6) δ 81.95, 44.12, 33.69, 31.95, 26.72, 24.47, 22.84, 14.01.
*N*-dodecyl-4,4,5,5-tetramethyl-*N*-(4,4,5,5-tetramethyl-1,3,2-dioxaborolan-2-yl)-1,3,2-dioxaborolan-2-amine (**4v**) [20,33]: 1H NMR (400 MHz, Benzene-d6) δ 3.37 (t, *J* = 7.2 Hz, 2H), 1.69 (p, *J* = 7.1 Hz, 2H), 1.23–1.16 (m, 18H), 1.01 (s, 24H), 0.84 (d, *J* = 6.4 Hz, 3H); 13C NMR (100 MHz, Benzene-d6) δ 81.89, 44.05, 33.65, 32.02, 29.86, 29.50, 27.01, 24.45, 22.80, 14.06.
*N*-(2-methoxyethyl)-4,4,5,5-tetramethyl-*N*-(4,4,5,5-tetramethyl-1,3,2-dioxaborolan-2-yl)-1,3,2-dioxaborolan-2-amine (**4w**) [64]: 1H NMR (400 MHz, Benzene-d6) δ 3.43 (t, *J* = 7.2 Hz, 2H), 3.30 (t, *J* = 6.7 Hz, 2H), 3.06 (s, 3H), 0.98 (s, 24H); 13C NMR (100 MHz, Benzene-d6) δ 81.96, 70.75, 57.90, 41.37, 24.42.
*N*-Benzylaniline (**6a**) [68]: White solid (91 mg, 99% yield); 1H NMR (400 MHz, Chloroform-d) δ 7.41–7.30 (m, 4H), 7.30–7.26 (m, 1H), 7.21–7.12 (m, 2H), 6.73 (d, *J* = 1.2 Hz, 1H), 6.63 (d, *J* = 7.7 Hz, 2H), 4.33 (s, 2H), 4.02 (s, 1H); 13C NMR (100 MHz, Chloroform-d) δ 148.39, 139.70, 129.51, 128.88, 127.74, 127.46, 117.77, 113.07, 48.48.
*N*-Benzyl-4-methylaniline (**6b**) [68]: Pale yellow oil (98 mg, 99% yield); 1H NMR (400 MHz, Chloroform-d) δ 7.39–7.30 (m, 4H), 7.29–7.25 (m, 1H), 6.98 (d, *J* = 8.5 Hz, 2H), 6.56 (d, *J* = 8.3 Hz, 2H), 4.30 (s, 2H), 3.90 (s, 1H), 2.23 (s, 3H); 13C NMR (100 MHz, Chloroform-d) δ 152.33, 142.65, 139.92, 128.78, 127.73, 127.35, 115.07, 114.27, 55.93, 49.35.
*N*-Benzyl-4-methoxyaniline (**6c**) [68]: Pale yellow oil (106 mg, 99% yield); 1H NMR (400 MHz, Chloroform-d) δ 7.39–7.30 (m, 4H), 7.29–7.25 (m, 1H), 6.82–6.72 (m, 2H), 6.60 (d, *J* = 8.8 Hz, 2H), 4.28 (s, 2H), 3.83 (s, 1H), 3.73 (s, 3H); 13C NMR (100 MHz, Chloroform-d) δ 152.32, 142.63, 139.89, 128.77, 127.72, 127.33, 115.06, 114.26, 55.93, 49.35.
*N*-Benzyl-4-bromoaniline (**6d**) [68]: Pale yellow solid (131 mg, 99% yield); 1H NMR (400 MHz, Chloroform-d) δ 7.34 (d, *J* = 4.3 Hz, 4H), 7.31–7.25 (m, 1H), 7.25–7.20 (m, 2H), 6.57–6.45 (m, 2H), 4.29 (s, 2H), 4.07 (s, 1H); 13C NMR (100 MHz, Chloroform-d) δ 147.29, 139.12, 132.14, 128.94, 127.62, 127.59, 114.66, 109.23, 48.35.
*N*-(4-Methylbenzyl)aniline (**6e**) [68]: Pale yellow solid (98 mg, 99% yield); 1H NMR (400 MHz, Chloroform-d) δ 7.26 (d, *J* = 8.6 Hz, 2H), 7.21–7.12 (m, 4H), 6.71 (t, *J* = 7.3 Hz, 1H), 6.63 (d, *J* = 8.4 Hz, 2H), 4.28 (s, 2H), 3.97 (s, 1H), 2.34 (s, 3H); 13C NMR (100 MHz, Chloroform-d) δ 148.46, 137.06, 136.62, 129.54, 129.49, 127.75, 117.69, 113.06, 48.25, 21.35.
*N*-(4-Methoxybenzyl)aniline (**6f**) [68]: Pale yellow solid (106 mg, 99% yield); 1H NMR (400 MHz, Chloroform-d) δ 7.29 (d, *J* = 8.6 Hz, 2H), 7.23–7.11 (m, 2H), 6.87 (d, *J* = 8.6 Hz, 2H), 6.71 (t, *J* = 7.3 Hz, 1H), 6.63 (d, *J* = 7.7 Hz, 2H), 4.25 (s, 2H), 3.94 (s, 1H), 3.80 (s, 3H); 13C NMR (100 MHz, Chloroform-d) δ 159.05, 148.44, 131.65, 129.47, 129.01, 117.67, 114.22, 113.05, 55.46, 47.93.
*N*-(4-Fluorobenzyl)aniline (**6g**) [68]: Pale yellow oil (100 mg, 99% yield); 1H NMR (400 MHz, Chloroform-d) δ 7.33 (dd, *J* = 8.3, 5.5 Hz, 2H), 7.17 (t, *J* = 7.7 Hz, 2H), 7.02 (t, *J* = 8.6 Hz, 2H), 6.72 (td, *J* = 7.3, 1.0 Hz, 1H), 6.61 (d, *J* = 8.4 Hz, 2H), 4.29 (s, 2H), 4.01 (s, 1H); 13C NMR (100 MHz, Chloroform-d) δ 162.21 (d, J C-F= 245.0 Hz), 148.15, 135.33 (d, J C-F= 3.1 Hz), 129.49, 129.18 (d, J C-F = 8.1 Hz), 117.90, 115.61 (d, J C-F = 21.3 Hz), 113.07, 47.72.
*N*-(4-Chlorobenzyl)aniline (**6h**) [68]: Pale yellow solid (108 mg, 99% yield); 1H NMR (400 MHz, Chloroform-d) δ 7.29 (s, 4H), 7.16 (ddd, *J* = 8.5, 7.4, 1.1 Hz, 2H), 6.71 (tt, *J* = 7.4, 1.1 Hz, 1H), 6.60 (dq, *J* = 7.5, 1.1 Hz, 2H), 4.31 (s, 2H), 4.05 (s, 1H); 13C NMR (100 MHz, Chloroform-d) δ 148.07, 138.27, 133.01, 129.54, 128.95, 128.91, 117.98, 113.11, 47.73.
*N*-(4-Bromobenzyl)aniline (**6i**) [68]: Pale yellow solid (131 mg, 99% yield); 1H NMR (400 MHz, Chloroform-d) δ 7.45 (d, *J* = 8.3 Hz, 2H), 7.24 (d, *J* = 8.1 Hz, 2H), 7.16 (t, *J* = 7.7 Hz, 2H), 6.72 (t, *J* = 7.3 Hz, 1H), 6.60 (d, *J* = 8.4 Hz, 2H), 4.29 (s, 2H), 4.05 (s, 1H); 13C NMR (100 MHz, Chloroform-d) δ 148.00, 138.78, 131.88, 129.52, 129.25, 121.09, 117.98, 113.09, 47.76.
*N*-(4-(Trifluoromethyl)benzyl)aniline (**6j**) [68]: Pale yellow oil (124 mg, 99% yield); 1H NMR (400 MHz, Chloroform-d) δ 7.62–7.55 (m, 2H), 7.48 (d, *J* = 8.1 Hz, 2H), 7.17 (t, *J* = 7.7 Hz, 2H), 6.73 (t, *J* = 7.3 Hz, 1H), 6.60 (d, *J* = 8.4 Hz, 2H), 4.43–4.39 (m, 2H), 4.14 (s, 1H); 13C NMR (100 MHz, Chloroform-d) δ 147.09, 140.07, 132.10, 127.19, 126.51, 121.98, 114.64, 109.36, 43.82.
*N*-(Naphthalen-2-ylmethyl)aniline (**6k**) [68]: Pale yellow solid (115 mg, 99% yield); 1H NMR (400 MHz, Chloroform-d) δ 7.86–7.78 (m, 4H), 7.53–7.41 (m, 3H), 7.18 (tt, *J* = 7.4, 1.1 Hz, 2H), 6.77–6.69 (m, 2H), 6.67 (q, *J* = 1.0 Hz, 2H), 4.50 (s, 2H), 4.14 (s, 1H); 13C NMR (100 MHz, Chloroform-d) δ 148.27, 137.04, 133.59, 132.86, 129.41, 128.49, 127.87, 127.81, 126.27, 126.02, 125.84, 117.75, 113.03, 48.61.
*N*-(Pyren-1-ylmethyl)aniline (**6l**) [68]: Yellow solid (152 mg, 99% yield); 1H NMR (400 MHz, Chloroform-d) δ 8.32 (d, *J* = 9.2 Hz, 1H), 8.20 (d, *J* = 8.0 Hz, 2H), 8.17–8.10 (m, 2H), 8.09–8.04 (m, 3H), 8.02 (t, *J* = 7.6 Hz, 1H), 7.25–7.19 (m, 2H), 6.81–6.72 (m, 3H), 4.99 (s, 2H), 4.10 (s, 1H); 13C NMR (100 MHz, Chloroform-d) δ 148.38, 132.20, 131.40, 131.10, 130.91, 129.50, 129.07, 128.03, 127.55, 127.45, 126.80, 126.15, 125.42, 125.35, 124.95, 124.90, 123.10, 117.82, 112.93, 46.82.
4-Bromo-*N*-(thiophen-3-ylmethyl)aniline (**6m**) [68]: Pale yellow solid (133 mg, 99% yield); 1H NMR (400 MHz, Chloroform-d) δ 7.30 (dd, *J* = 4.9, 3.0 Hz, 1H), 7.24 (d, *J* = 8.3 Hz, 2H), 7.17 (dt, *J* = 2.9, 1.3 Hz, 1H), 7.05 (dd, *J* = 5.0, 1.4 Hz, 1H), 6.57–6.46 (m, 2H), 4.29 (d, *J* = 4.7 Hz, 2H), 4.01 (s, 1H); 13C NMR (100 MHz, Chloroform-d) δ 147.12, 140.10, 132.12, 127.23, 126.53, 122.01, 114.67, 109.35, 43.82.
*N*-(1-Phenylethyl)aniline (**6n**) [68]: Pale yellow oil (71 mg, 72% yield); 1H NMR (400 MHz, Chloroform-d) δ 7.36 (d, *J* = 7.6 Hz, 2H), 7.31 (t, *J* = 7.6 Hz, 2H), 7.22 (t, *J* = 7.2 Hz, 1H), 7.09 (t, *J* = 7.9 Hz, 2H), 6.65 (t, *J* = 7.3 Hz, 1H), 6.53 (d, *J* = 8.0 Hz, 2H), 4.48 (q, *J* = 6.7 Hz, 1H), 1.52 (d, *J* = 6.7 Hz, 3H); 13C NMR (100 MHz, Chloroform-d) δ 147.37, 145.32, 129.20, 128.74, 126.96, 125.94, 117.31, 113.36, 53.54, 25.16.
4-Bromo-*N*-(1-phenylethyl)aniline (**6o**) [68]: Pale yellow solid (128 mg, 92% yield); 1H NMR (400 MHz, Chloroform-d) δ 7.34–7.28 (m, 4H), 7.24–7.18 (m, 1H), 7.14 (d, *J* = 8.7 Hz, 2H), 6.37 (d, *J* = 8.6 Hz, 2H), 4.42 (q, *J* = 6.8 Hz, 1H), 4.07 (s, 1H), 1.50 (d, *J* = 6.7 Hz, 3H); 13C NMR (100 MHz, Chloroform-d) δ 146.25, 144.70, 131.87, 128.83, 127.15, 125.84, 114.96, 108.73, 53.58, 25.09.
4-Methoxy-*N*-(1-phenylethyl)aniline (**6p**) [68]: Pale yellow soild (109 mg, 96% yield); 1H NMR (400 MHz, Chloroform-d) δ 7.37–7.28 (m, 4H), 7.24–7.18 (m, 1H), 6.70–6.66 (m, 2H), 6.49–6.44 (m, 2H), 4.40 (q, *J* = 6.7 Hz, 1H), 3.68 (s, 3H), 1.50 (s, 3H); 3H); 13C NMR (100 MHz, Chloroform-d) δ 151.99, 145.62, 141.69, 128.74, 126.94, 126.01, 114.86, 114.65, 55.84, 54.36, 25.28.
4-(Hydroxymethyl)benzonitrile (**8a**) [69]: 1H NMR (400 MHz, Chloroform-d) δ 7.65 (d, *J* = 8.0 Hz, 2H), 7.47 (d, *J* = 7.9 Hz, 2H), 4.78 (d, *J* = 5.6 Hz, 2H), 1.84 (t, *J* = 5.8 Hz, 1H); 13C NMR (100 MHz, Chloroform-d) δ 146.41, 132.41, 127.11, 118.99, 111.12, 64.24.
(4-Vinylphenyl)methanol (**8b**) [70]: 1H NMR (400 MHz, Chloroform-d) δ 7.40 (d, *J* = 8.1 Hz, 2H), 7.32 (d, *J* = 8.1 Hz, 2H), 6.71 (dd, *J* = 17.6, 10.9 Hz, 1H), 5.75 (d, *J* = 17.6 Hz, 1H), 5.24 (d, *J* = 10.9 Hz, 1H), 4.68 (s, 2H); 13C NMR (100 MHz, Chloroform-d) δ 140.55, 137.05, 136.60, 127.32, 126.48, 113.98, 65.02.
(4-Ethynylphenyl)methanol (**8c**) [71]: 1H NMR (400 MHz, Chloroform-d) δ 7.48 (d, *J* = 8.1 Hz, 2H), 7.31 (d, *J* = 8.0 Hz, 2H), 4.69 (s, 2H), 3.06 (s, 1H), 1.74 (s, 1H); 13C NMR (100 MHz, Chloroform-d) δ 141.69, 132.37, 126.83, 121.28, 83.63, 77.40, 64.68.

All products were characterized by comparing their NMR with those reported in literature. For details, see Supplementary Materials.

## 4. Conclusions

In this study, we developed a Grignard reagent-catalyzed hydroboration reaction for esters, nitriles, and imines using HBpin as the hydroboration reagent at room temperature. Various alkyl magnesium halides were screened for this reaction, and the commercially available Grignard reagent MeMgCl was determined to be the optimal catalyst, as it afforded excellent yields (99%). Various esters, nitriles, and imines having aromatic, hetero-aromatic, and aliphatic substrates underwent smooth hydroboration which afforded the corresponding products excellent yields. We also investigated the intramolecular chemoselectivity of this hydroboration by using substrates containing both esters and other reducible groups such as nitriles, alkenes, and alkynes and observed that the reaction is highly selective for esters, affording good conversions (81–97%). In addition, based on the free energy profiles obtained from DFT calculations, we proposed a plausible mechanism for the ester hydroboration. Our green hydroboration protocol eliminates the requirements of complex ligand systems and elevated temperatures, providing an effective method for the reduction in esters, nitriles, and imines at room temperature.

## Figures and Tables

**Table 1:** Hydroboration of ester using various alkyl magnesium halides.

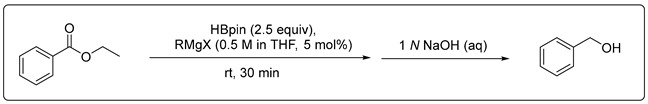		
Entry	RMgX	Conversion ^a^ (%)
1	MeMgCl	99
2	MeMgBr	99
3	MeMgI	75
4	BuMgCl	93
5	*t*-BuMgCl	96
6	*i*-PrMgCl	82
7	PhMgCl	89

^a^ The conversion percentages were determined using gas chromatography based on the consumption of the starting material.

**Table 2:** Optimization of the pinacolborane (HBpin) content and the catalyst (0.5 M MeMgCl in THF) loading for the ester hydroboration.

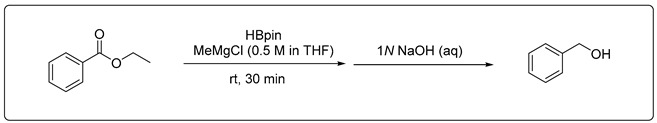			
Entry	HBpin (Equiv)	MeMgCl (mol%)	Conversion ^a^ (%)
1	2.0	5	94
2	2.2	5	96
3	2.4	5	98
4	2.5	5	99
5	2.5	3	63
6	2.5	4	82

^a^ The conversion percentages were determined using gas chromatography based on the consumption of the starting material.

**Table 3:** Substrate scope of ester hydroboration ^a^.

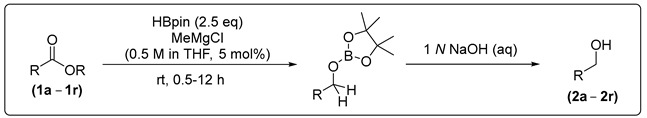		
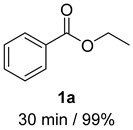	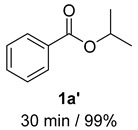	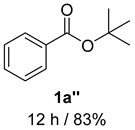
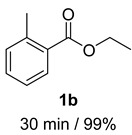	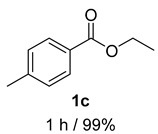	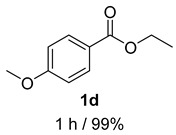
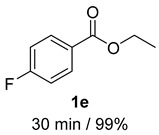	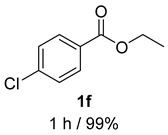	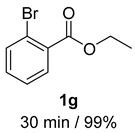
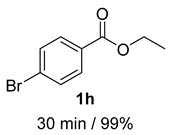	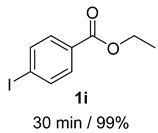	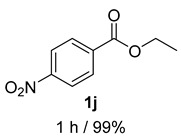
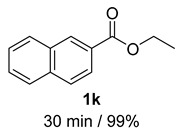	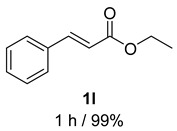	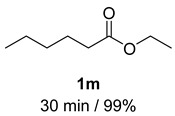
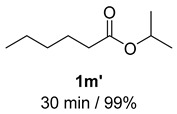	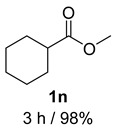	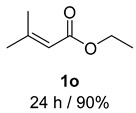
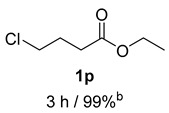	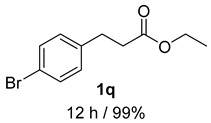	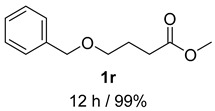

^a^ Isolated yields after silica column chromatography. ^b^ Yields were calculated by ^1^H NMR with 1,3,5-trimethoxybenzene as the internal standard.

**Table 4:** Hydroboration of nitrile using various alkyl magnesium halides.

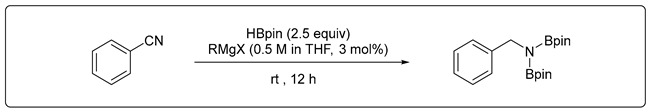		
Entry	RMgX	Conversion ^a^ (%)
1	MeMgCl	99
2	MeMgBr	58
3	MeMgI	57
4	BuMgCl	94
5	*i*-PrMgCl	99
6	*t*-BuMgCl	99
7	PhMgCl	92
8	PhMgBr	70

^a^ The conversion percentages were determined using gas chromatography based on the consumption of the starting material.

**Table 5:** Optimization of the pinacolborane (HBpin) content and catalyst loading (MeMgCl in THF) for nitrile hydroboration.

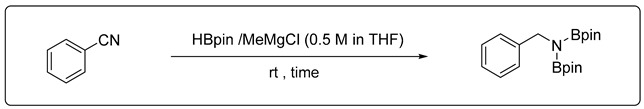				
Entry	HBpin (Equiv)	MeMgCl (mol%)	Time (h)	Conversion ^a^ (%)
1	3.0	3.0	6	97
2	3.0	3.0	12	99
3	3.0	2.0	12	45
4	2.5	3.0	12	99
5	2.5	2.0	12	83
6	2.5	2.0	24	99
7	2.2	3.0	12	98
8	2.2	3.0	24	99

^a^ The conversion percentages were determined using gas chromatography based on the consumption of the starting material.

**Table 6:** Substrate scope of the nitrile hydroboration ^a^.

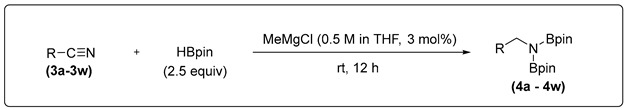			
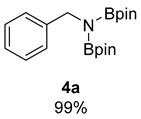	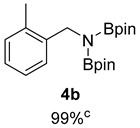	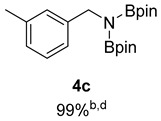	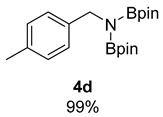
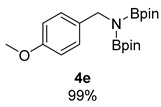	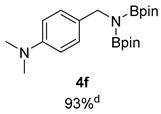	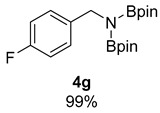	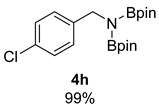
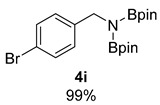	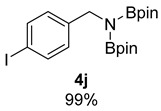	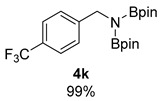	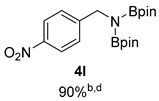
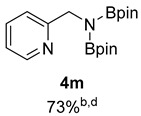	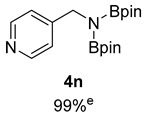	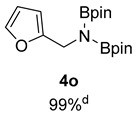	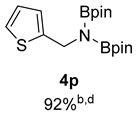
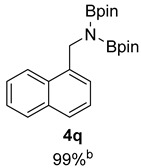	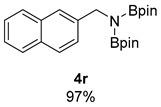	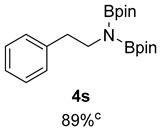	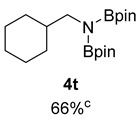
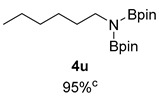	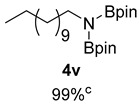	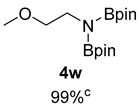	

^a^ Yields were calculated by ^1^H NMR with 1,3,5-trimethoxybenzene as the internal standard. ^b^ 5 mol%. ^c^ 10 mol%, ^d^ 24 h, ^e^ 80 °C.

**Table 7:** Hydroboration of imine by various alkyl magnesium halides.

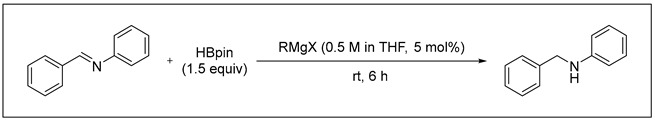		
Entry	RMgX	Conversion ^a^ (%)
1	MeMgCl	99
2	MeMgBr	91
3	MeMgI	78
4	*i*-PrMgCl	83
5	BuMgCl	97
6	*t*-BuMgCl	99
7	PhMgCl	83

^a^ The conversion percentages were determined using gas chromatography based on the consumption of the starting material.

**Table 8:** Optimization of the pinacolborane (HBpin) content and catalyst loading (MeMgCl in THF) for imine hydroboration.

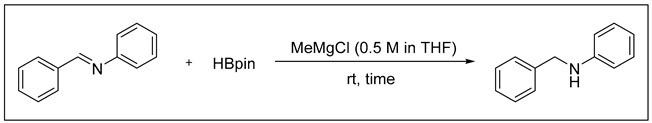				
Entry	MeMgCl (mol%)	HBpin (Equiv)	Time (h)	Conversion ^a^ (%)
1	5	2.0	3	77
2	5	1.5	6	99
3	5	1.5	3	52
4	5	1.3	6	64
5	5	1.3	12	89
6	3	1.5	6	21
7	3	2.0	12	53

^a^ The conversion percentages were determined using gas chromatography based on the consumption of the starting material.

**Table 9:** Substrate scope of the imine hydroboration ^a^.

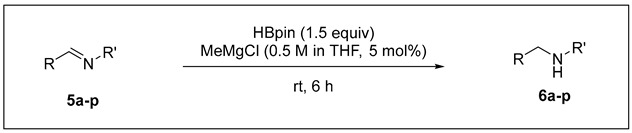		
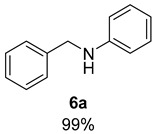	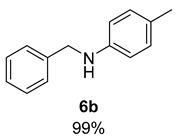	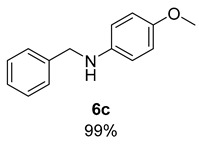
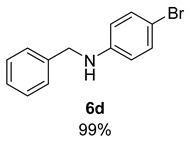	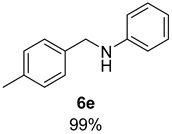	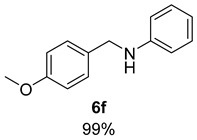
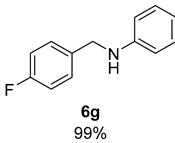	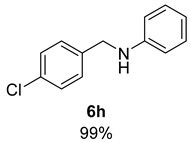	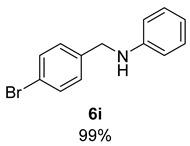
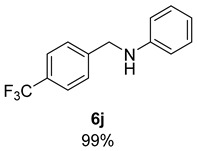	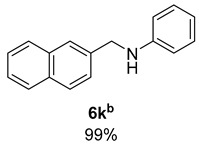	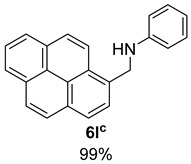
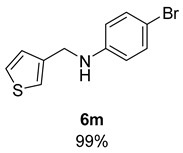	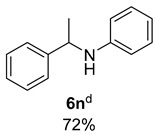	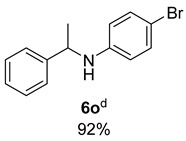
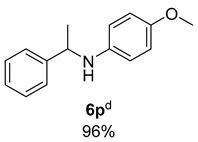		

^a^ Isolated yield after silica column chromatography. ^b^ HBpin (2.0 eq). ^c^ THF 0.2 mL. ^d^ MeMgCl (7 mol%), HBpin (2.5 eq), 40 °C.

## Data Availability

Data are in the Supplementary Materials.

## Supplementary Materials

The following supporting information can be downloaded at: https://www.mdpi.com/article/10.3390/molecules28207090/s1, Optimization of conditions for ester hydroboration (Table S1. Hydroboration of ester under various concentrations of catalyst; Table S2. Identification of solvent for the preparation of various concentrations of catalyst); Optimization of conditions for nitrile hydroboration (Table S3. Hydroboration of nitriles under various concentrations of catalyst; Table S4. Identification of solvent for the preparation of various concentrations of catalyst); Optimization of conditions for imine hydroboration (Table S5. Hydroboration of imines under various concentrations of catalyst; Table S6. Identification of solvent for the preparation of various concentrations of catalyst); and copies of ^1^H and ^13^C NMR spectra (Figures S1–S126) of products; DFT calculation data. Refs. [56,57,58,59,60,61,62,63,64,65,66,67,68,69,70,71] are cited in Supplementary Materials.

## Sample Availability

Samples of the compounds are available from the authors.
