# Supplementary material for: Grignard Reagent-Catalyzed Hydroboration of Esters, Nitriles, and Imines
Source: Molecules. 2023 Oct 14;28(20):7090. doi: 10.3390/molecules28207090 (PMC10609653; doi:10.3390/molecules28207090)
Supplement: Supplementary file 1 [file molecules-28-07090-s001.zip › molecules-2632194-supplementary.pdf]

Supporting Information

Grignard reagents Catalyzed Hydroboration of Esters, Nitriles and Imines

Hyun Ji Han, Suh Youn Park, So Eun Jeon, Jae Seok Kwak, Ji Hye Lee, Ashok Kumar Jaladi, Hyonseok Hwang, and Duk Keun An*

Department of Chemistry, Kangwon National University

Chuncheon 24341, Republic of Korea

E-mail: dkan@kangwon.ac.kr

Table of Contents

1. Optimization of conditions for ester hydroboration.. S1

2. Optimization of conditions for nitrile hydroboration.. S2

3. Optimization of conditions for imine hydroboration.. S3

4. Copies of analysis data... S4-S66

5. Computational Section S67-S80

6. References.. S81-S82

1. Optimization of conditions for ester hydroboration

Table S1. Hydroboration of ester under various concentrations of catalyst

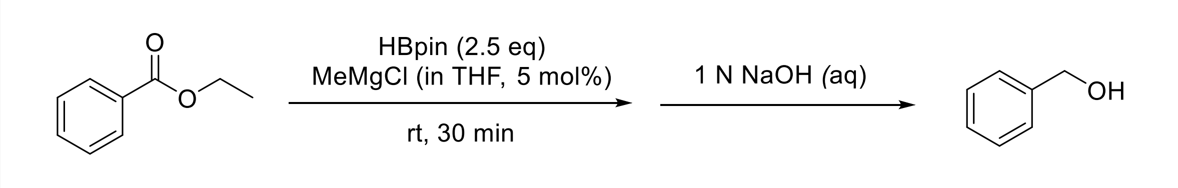

Entry	MeMgCl	Conversion[a] (%)
1	3 M	95 / 94 / 93
2	1 M	94 / 91 / 93
3	0.5 M	99 / 99 / 99
4	0.1 M	97 / 98 / 97
5	0.05 M	95 / 93 / 84

[a]The conversion percentages were determined using gas chromatograph based on the consumption of the starting material.

Table S2. Identification of solvent for the preparation of various concentrations of catalyst

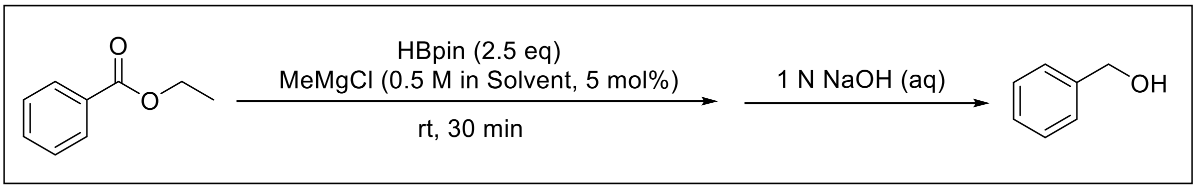

Entry	Solvent	Conversion[a] (%)
1	Ether	90
2	Hexane	16
3	MC	97
4	Toluene	88
5	THF	99

[a]The conversion percentages were determined using gas chromatograph based on the consumption of the starting material.

2. Optimization of conditions for nitrile hydroboration

Table S3. Hydroboration of nitriles under various concentrations of catalyst

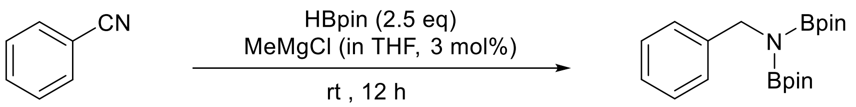

Entry	MeMgCl	Conversion (%)[a]
1	3 M	37 / 47 / 17
2	1 M	55 / 94 / 64
3	0.5 M	98 / 99 / 99
4	0.1 M	91 / 97 / 97
5	0.05 M	97 / 89 / 80
6	0.01 M	15 / 27 / 38

[a]The conversion percentages were determined using gas chromatograph based on the consumption of the starting material.

Table S4. Identification of solvent for the preparation of various concentrations of catalyst

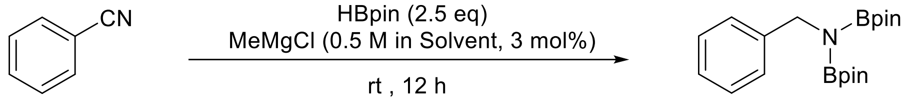

Entry	Solvent	Conversion (%)[a]
1	hexane	24
2	ether	82
3	toluene	25
4	MC	62
5	benzene	37
6	THF	99

[a]The conversion percentages were determined using gas chromatograph based on the consumption of the starting material.

3. Optimization of conditions for imine hydroboration

Table S5. Hydroboration of imines under various concentrations of catalyst

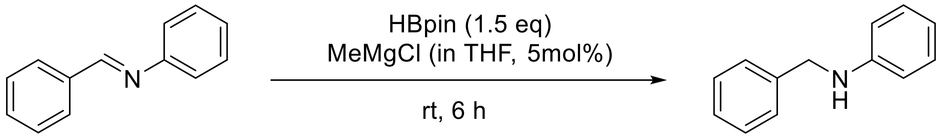

Entry	MeMgCl	Conversion[a] (%)
1	0.05 M	45
2	0.1 M	65
3	0.5 M	99
4	1 M	94
5	3 M	85

[a]The conversion percentages were determined using gas chromatograph based on the consumption of the starting material.

Table S6. Identification of solvent for the preparation of various concentrations of catalyst

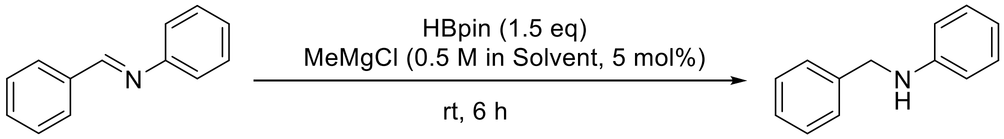

Entry	Solvent	Conversion[a] (%)
1	Toluene	79
2	MC	96
3	Hexane	55
4	Ether	93
5	THF	99

[a]The conversion percentages were determined using gas chromatograph based on the consumption of the starting material.

4. Copies of analysis data

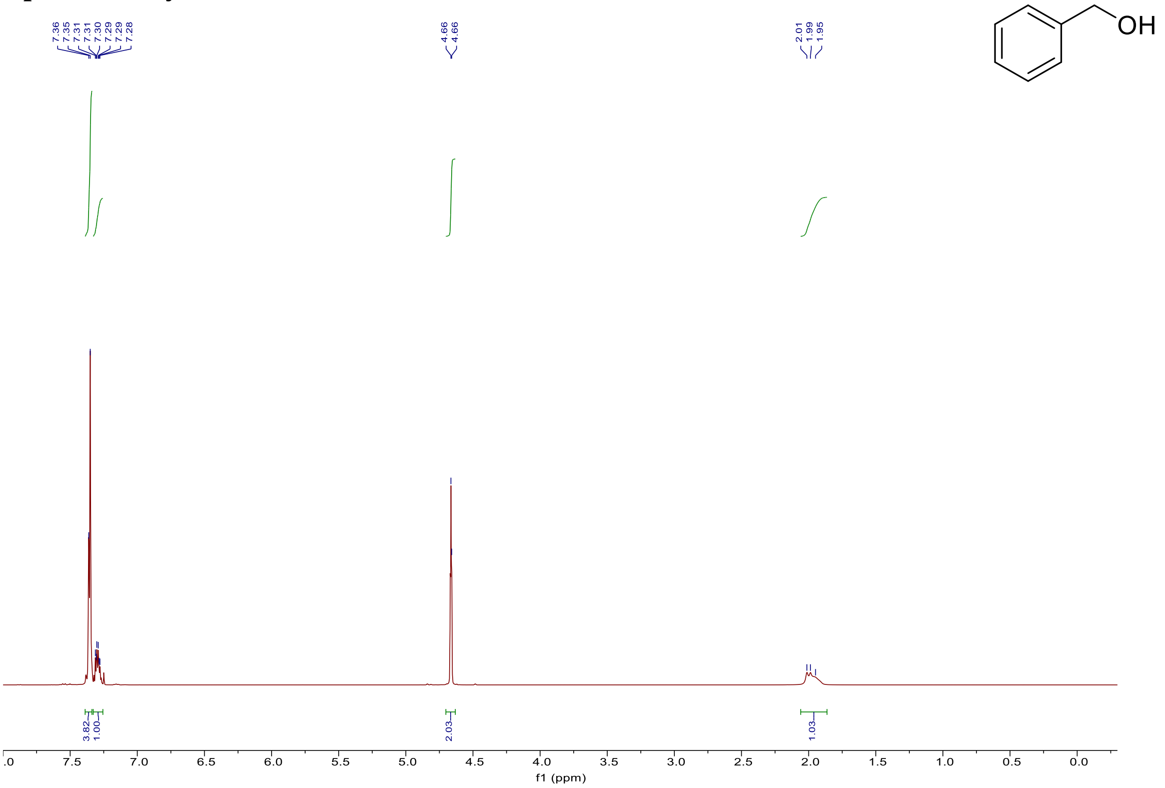

Figure S1 ^{1}H NMR of benzyl alcohol[S1](2a)

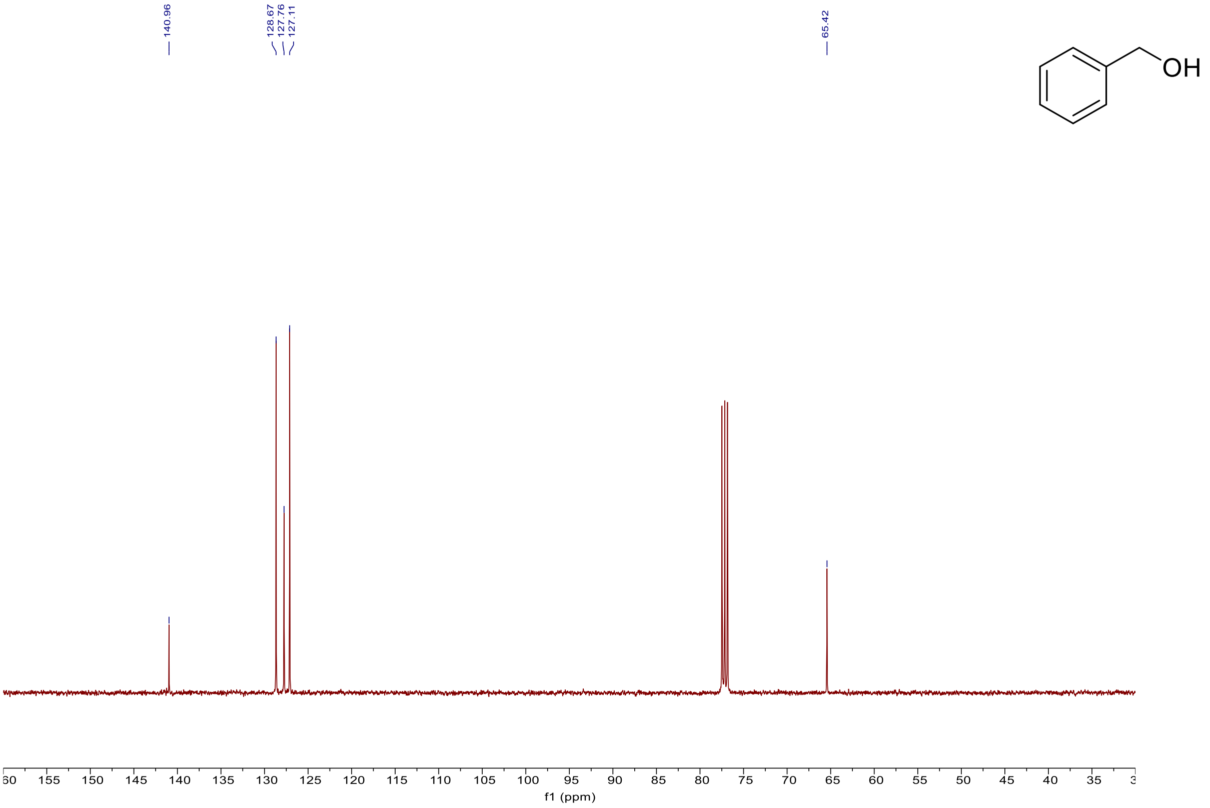

Figure S2 ^{13}C NMR of benzyl alcohol[S1](2a)

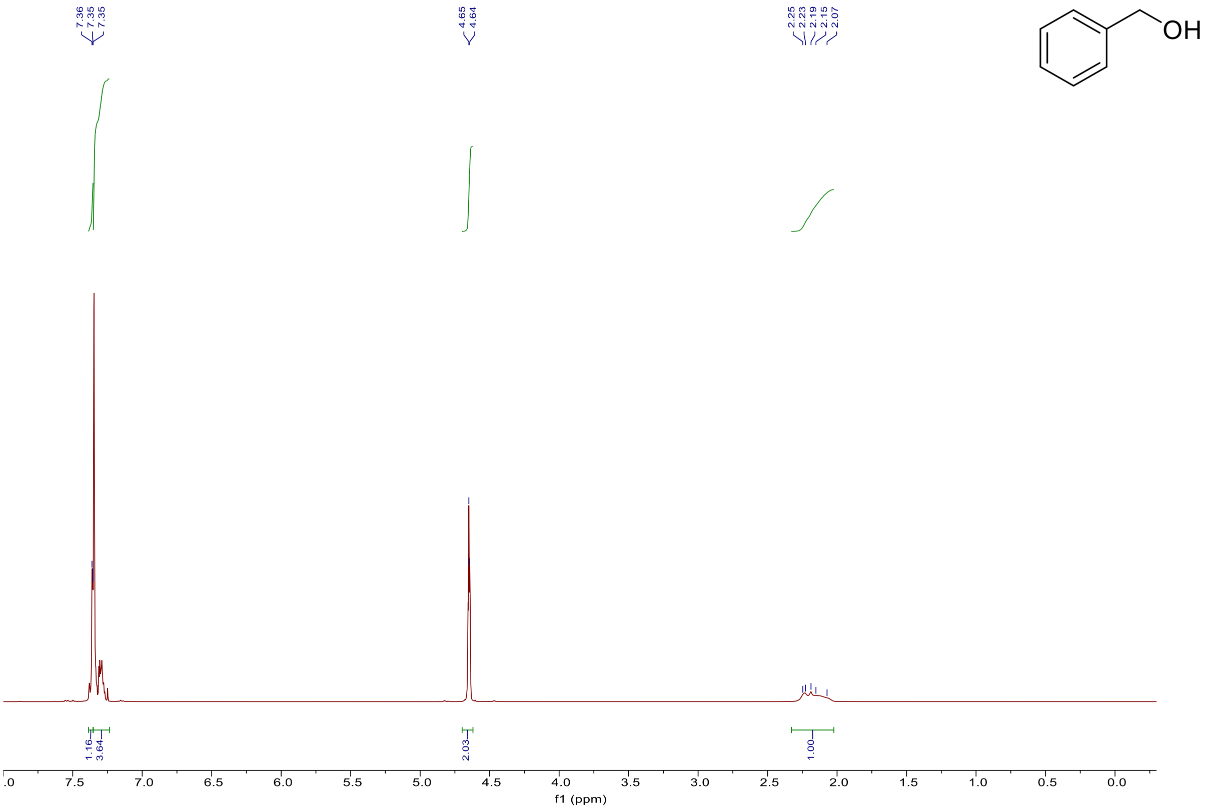

Figure S3 ^{1}H NMR of benzyl alcohol [S1] (2a')

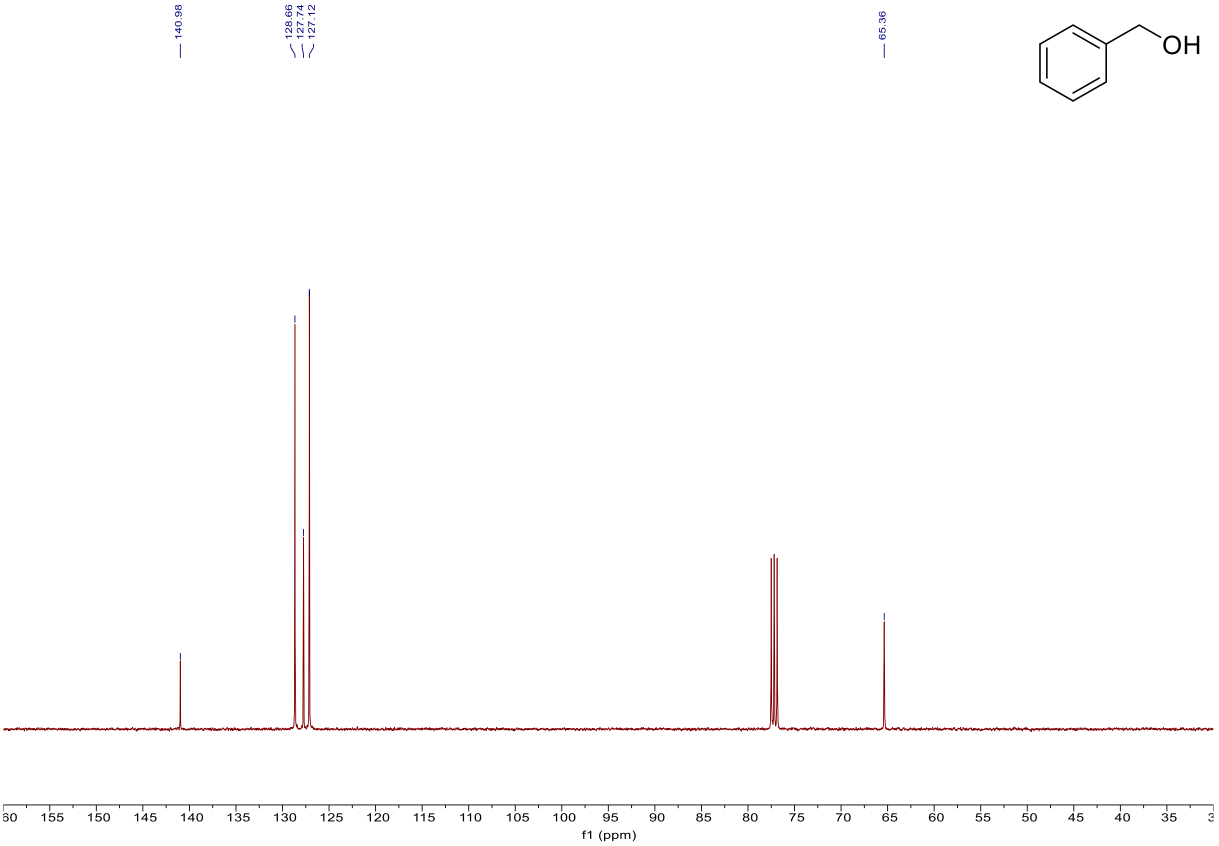

Figure S4 ^{13}C NMR of benzyl alcohol [S1] (2a')

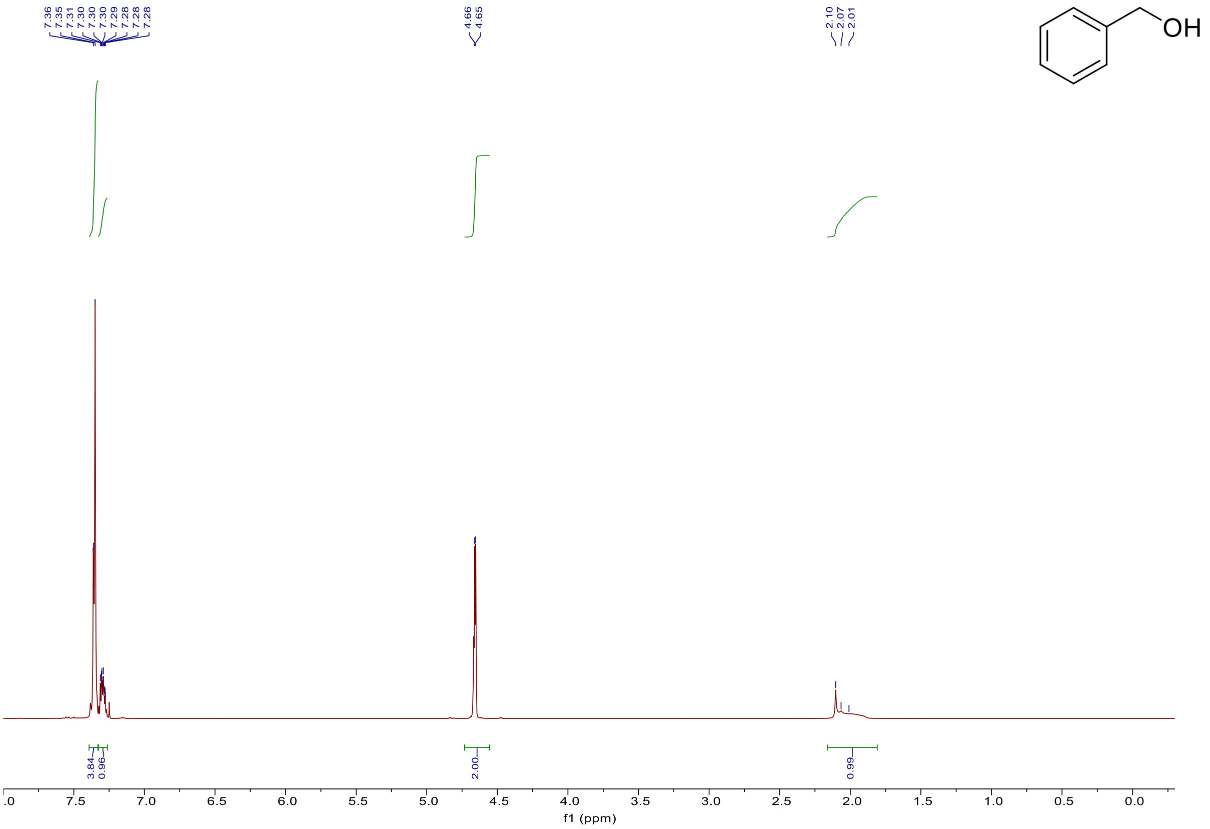

Figure S5 ^{1}H NMR of benzyl alcohol [S1](2a,,)

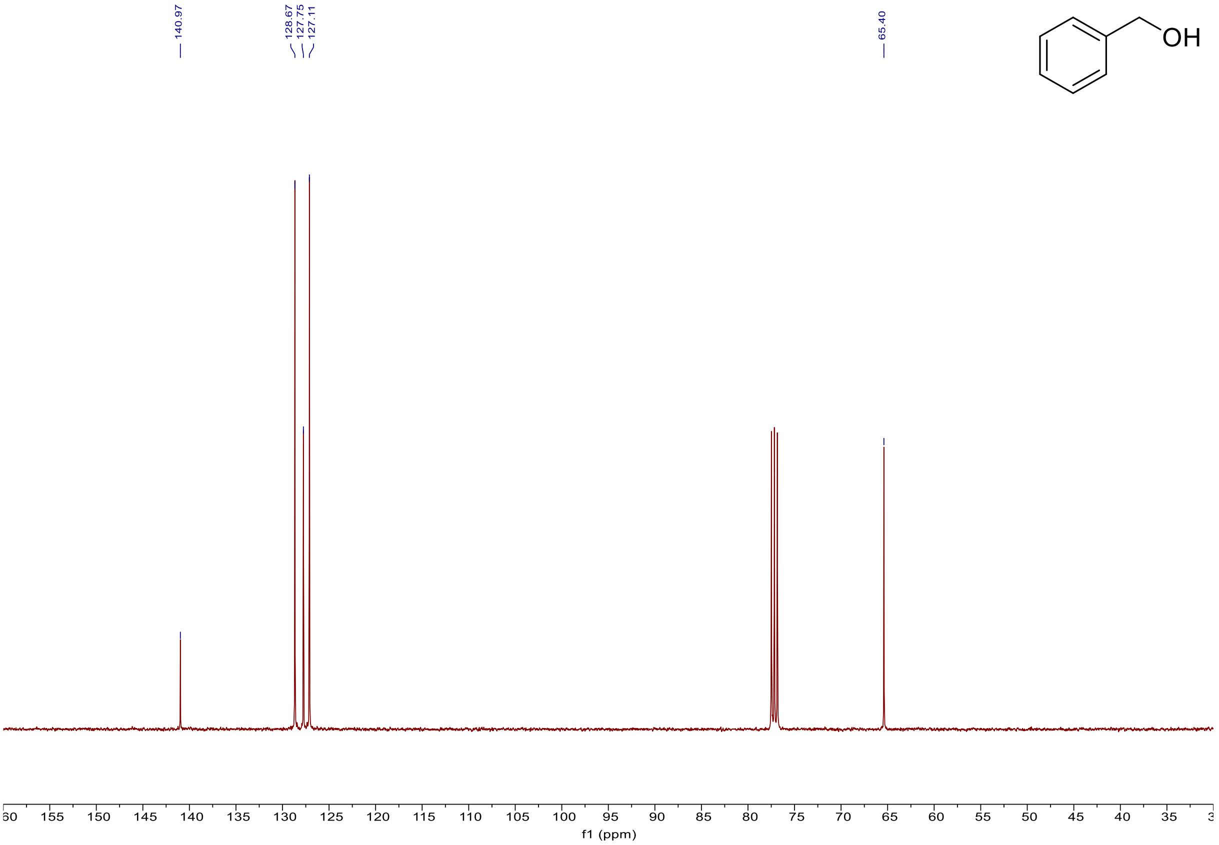

Figure S6 ^{13}C NMR of benzyl alcohol [S1](2a,,)

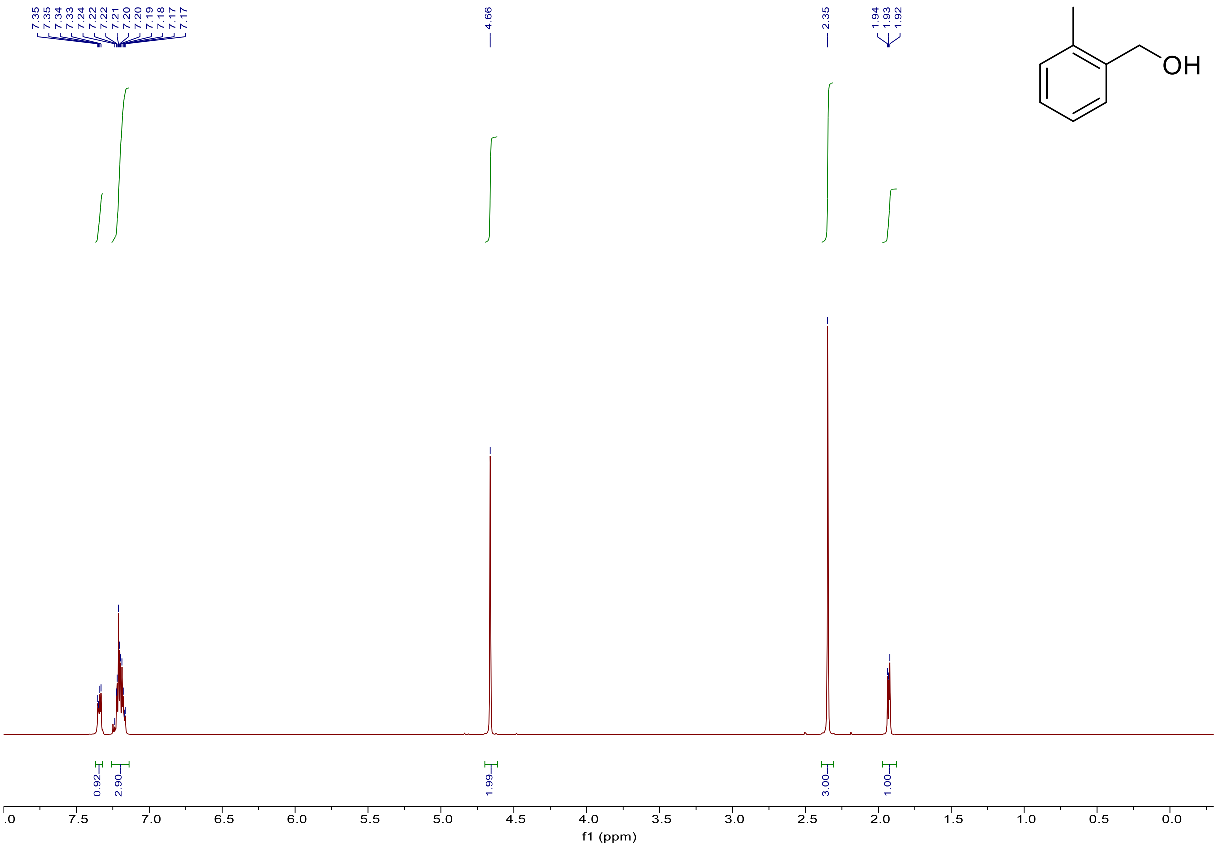

Figure S7 ^{1}H NMR of 2-metylbenzyl alcohol[S1](2b)

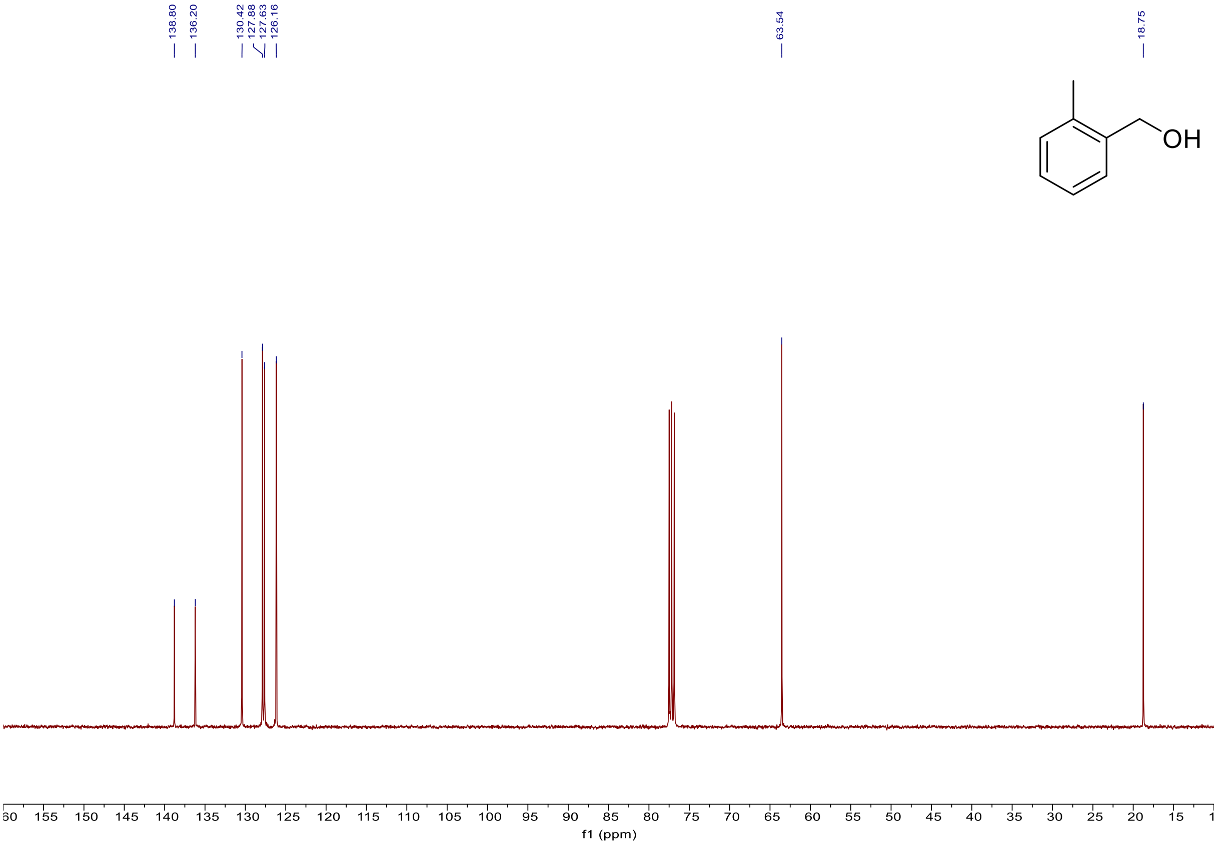

Figure S8 ^{13}C NMR of 2-metylbenzyl alcohol[S1](2b)

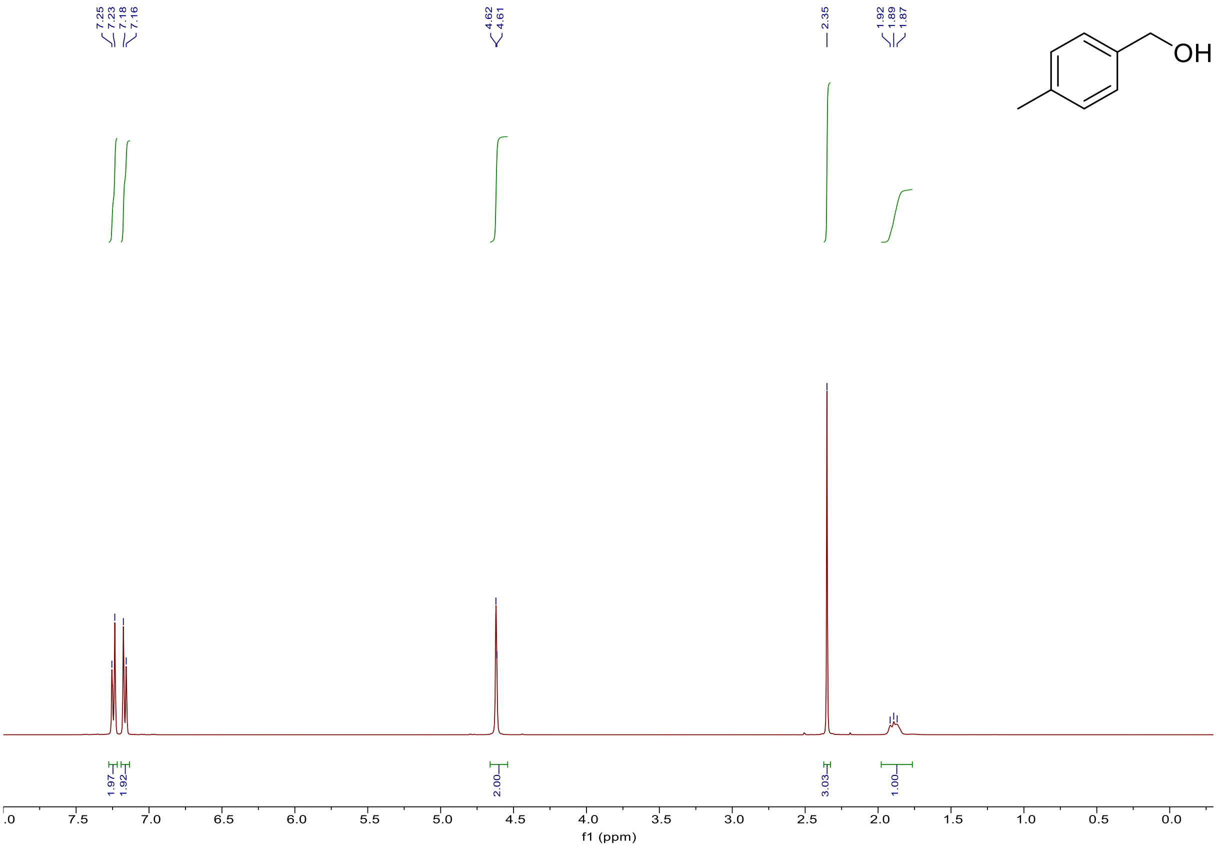

Figure S9 ^{1}H NMR of 4-metylbenzyl alcohol [S1] (2c)

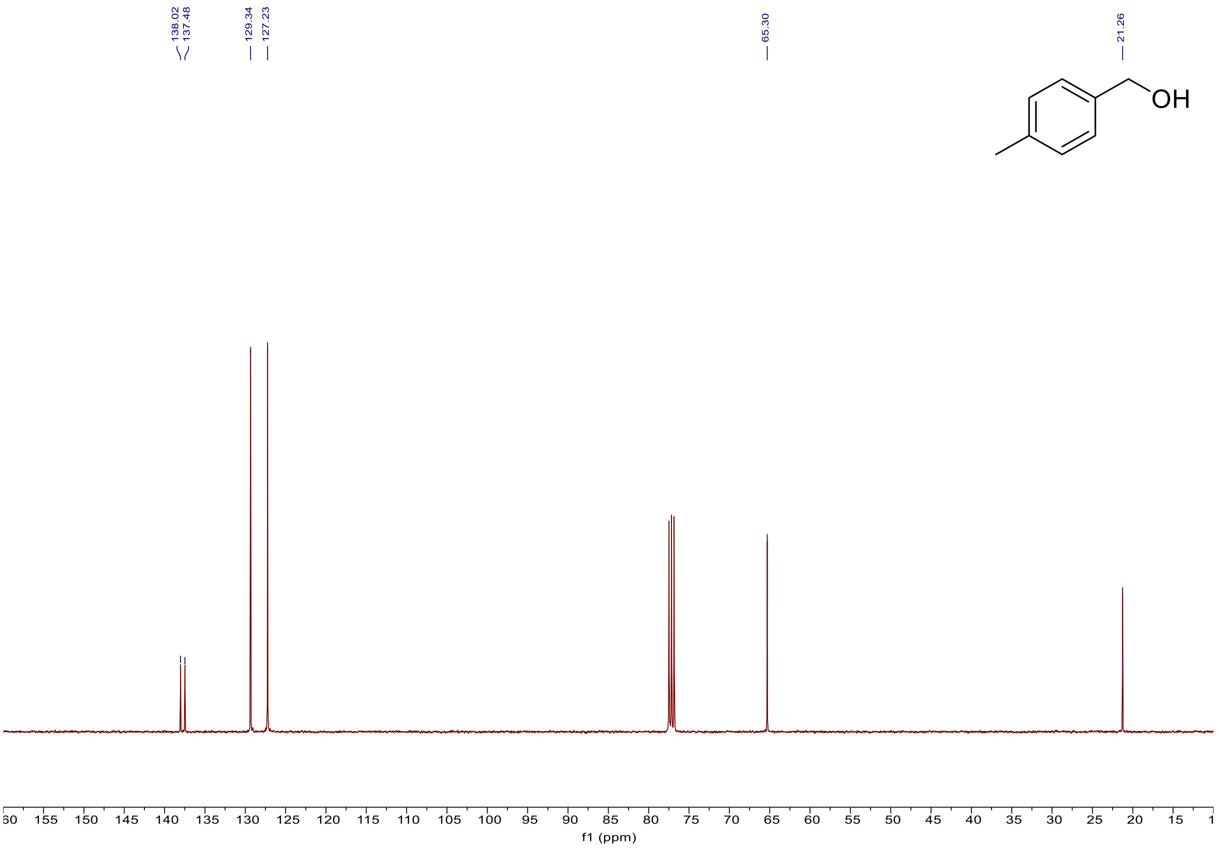

Figure S10 ^{13}C NMR of 4-metylbenzyl alcohol [S1] (2c)

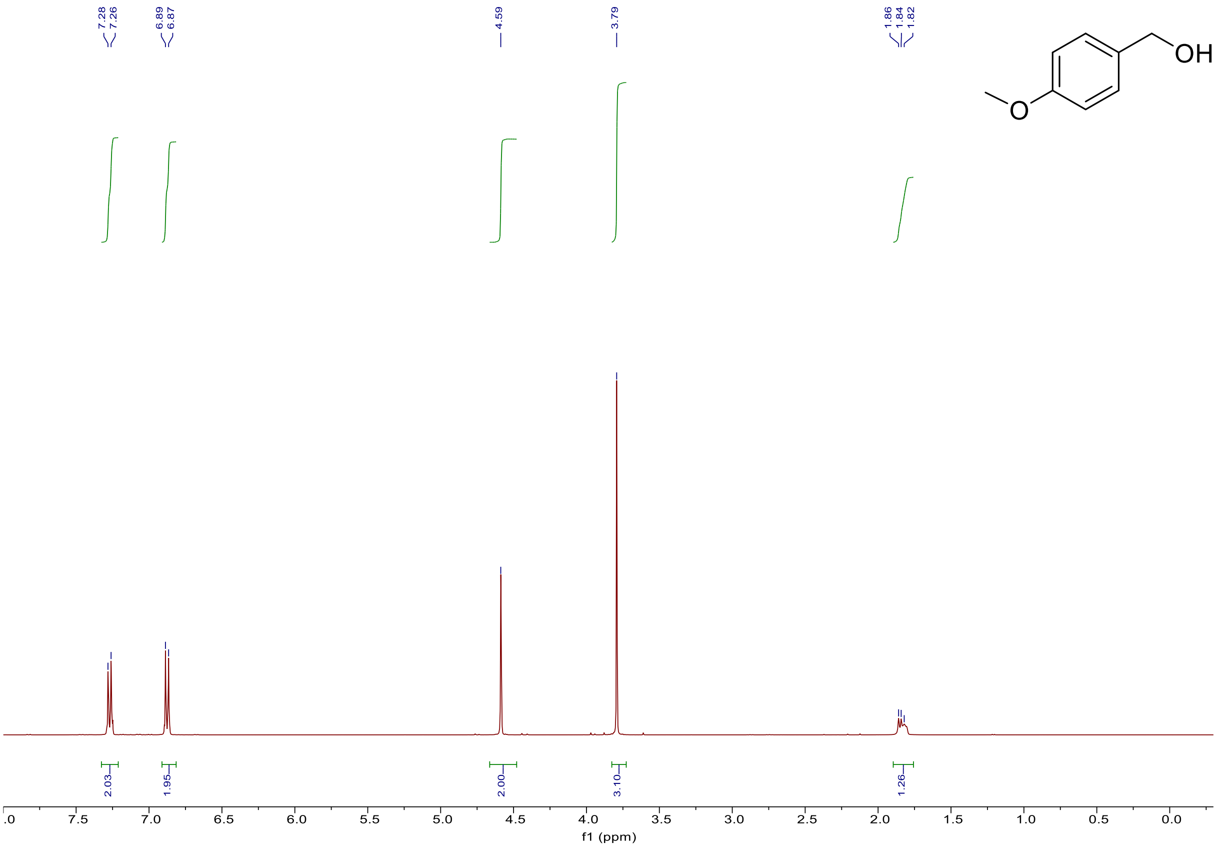

Figure S11 ^{1}H NMR of 4-methoxybenzyl alcohol [S1](2d)

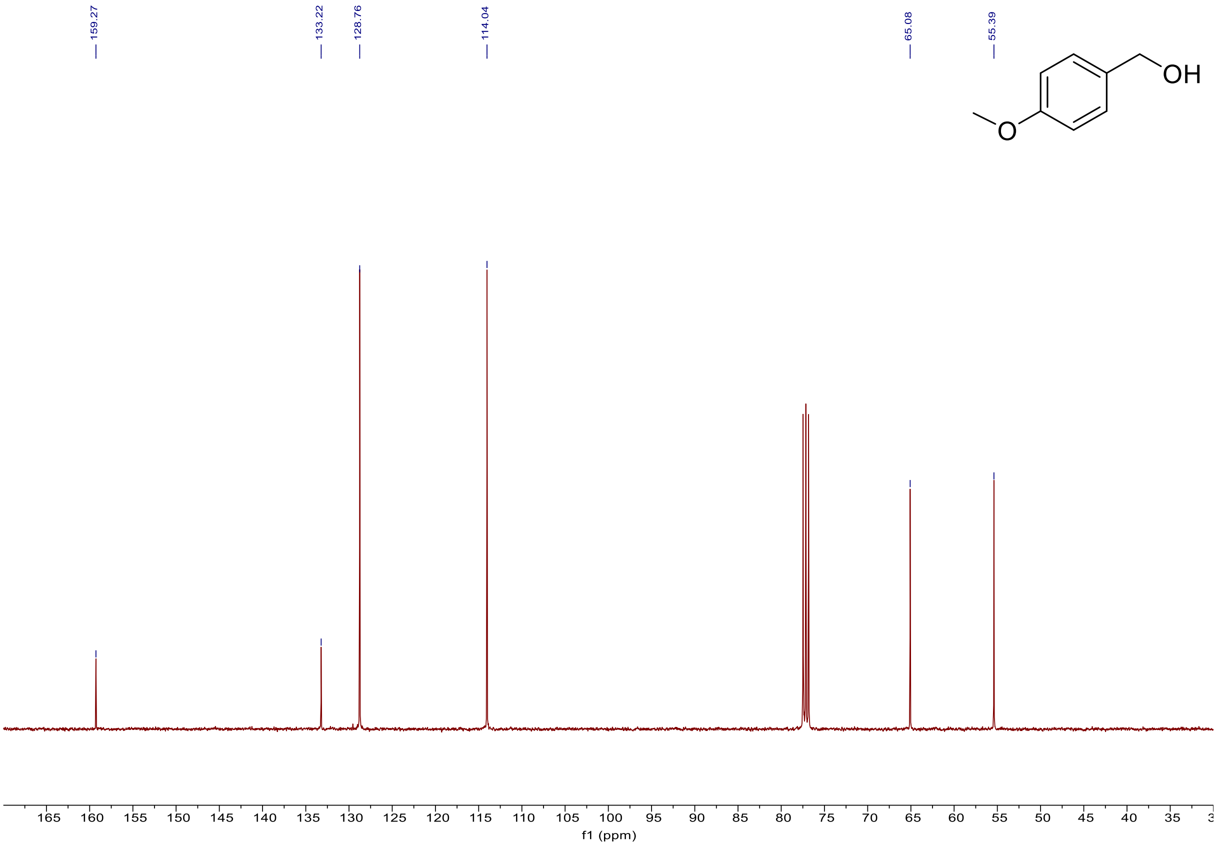

Figure S12 ^{13}C NMR of 4-methoxybenzyl alcohol [S1](2d)

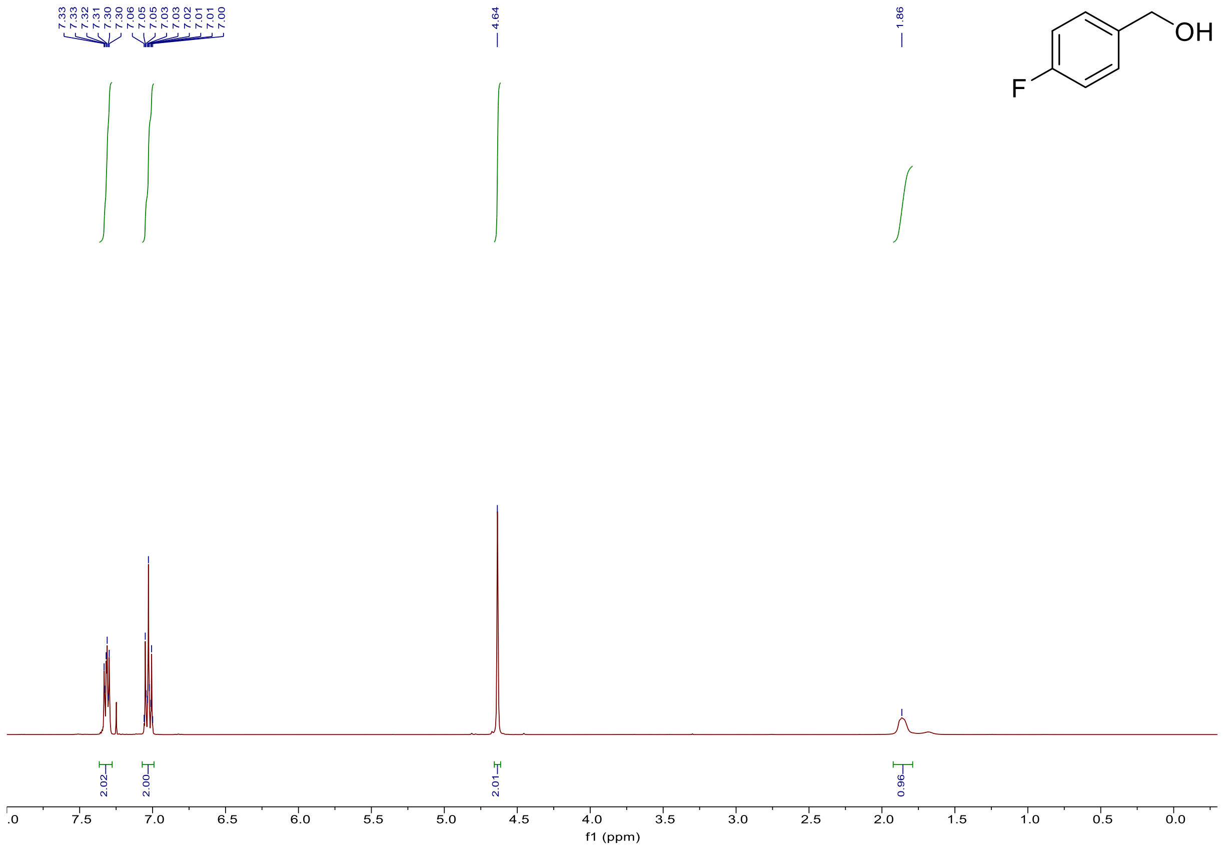

Figure S13 ^{1}H NMR of 4-fluorobenzyl alcohol [S1] (2e)

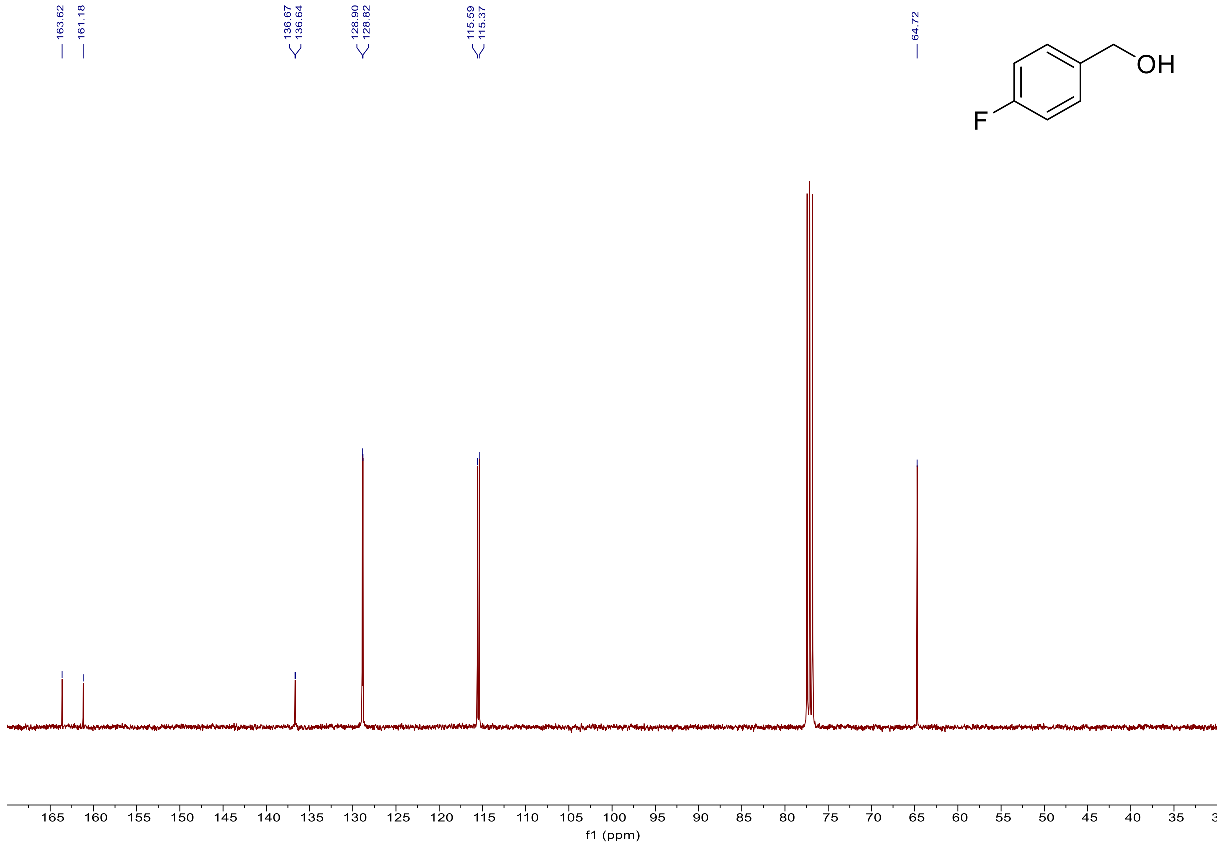

Figure S14 ^{13}C NMR of 4-fluorobenzyl alcohol [S1] (2e)

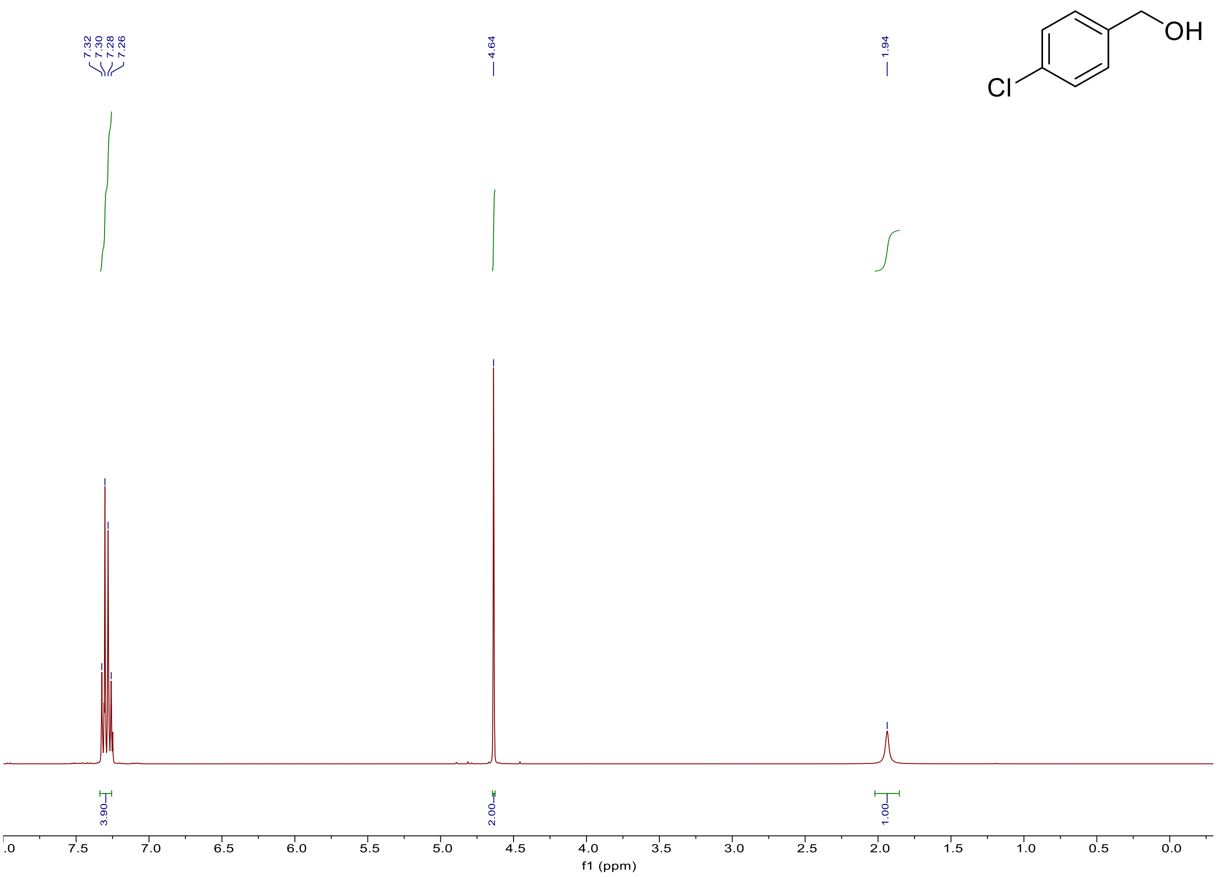

Figure S15 ^{1}H NMR of 4-chlorobenzyl alcohol [S2] (2f)

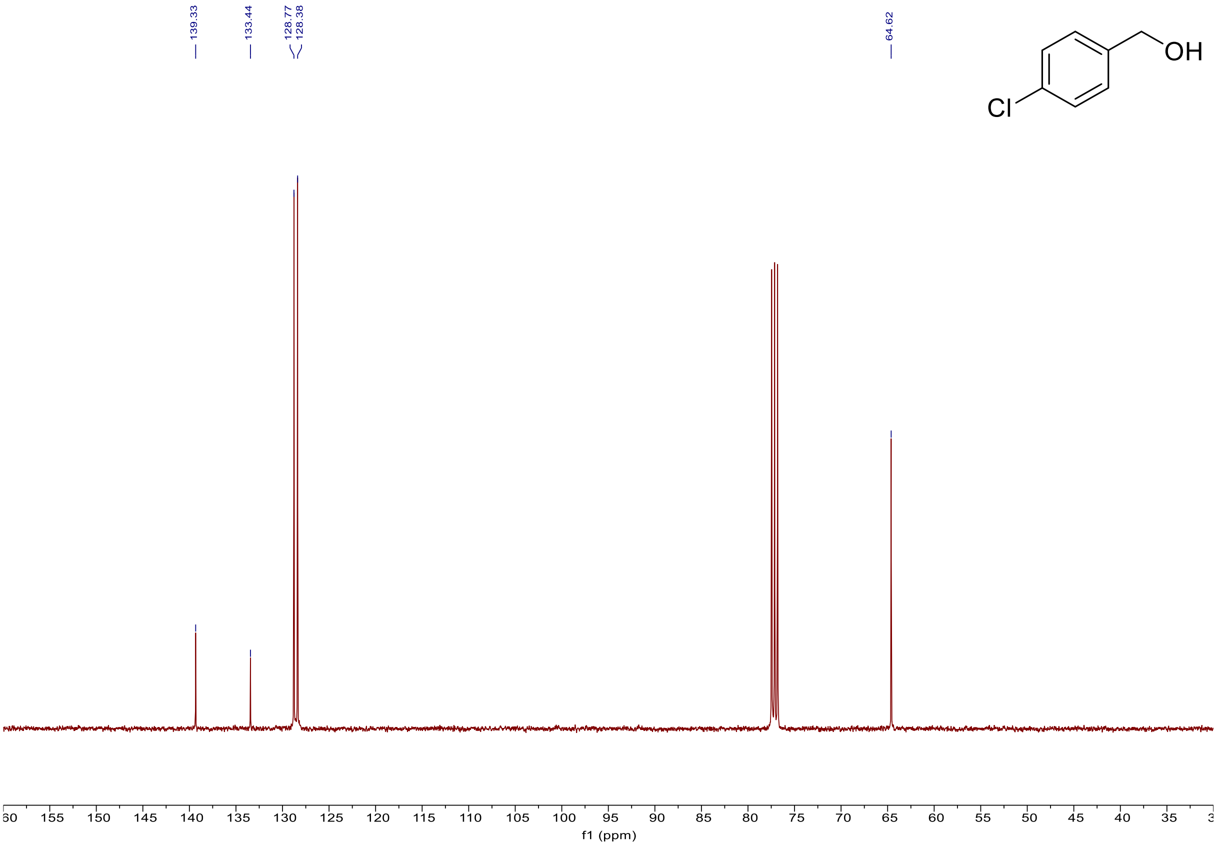

Figure S16 ^{13}C NMR of 4-chlorobenzyl alcohol [S2] (2f)

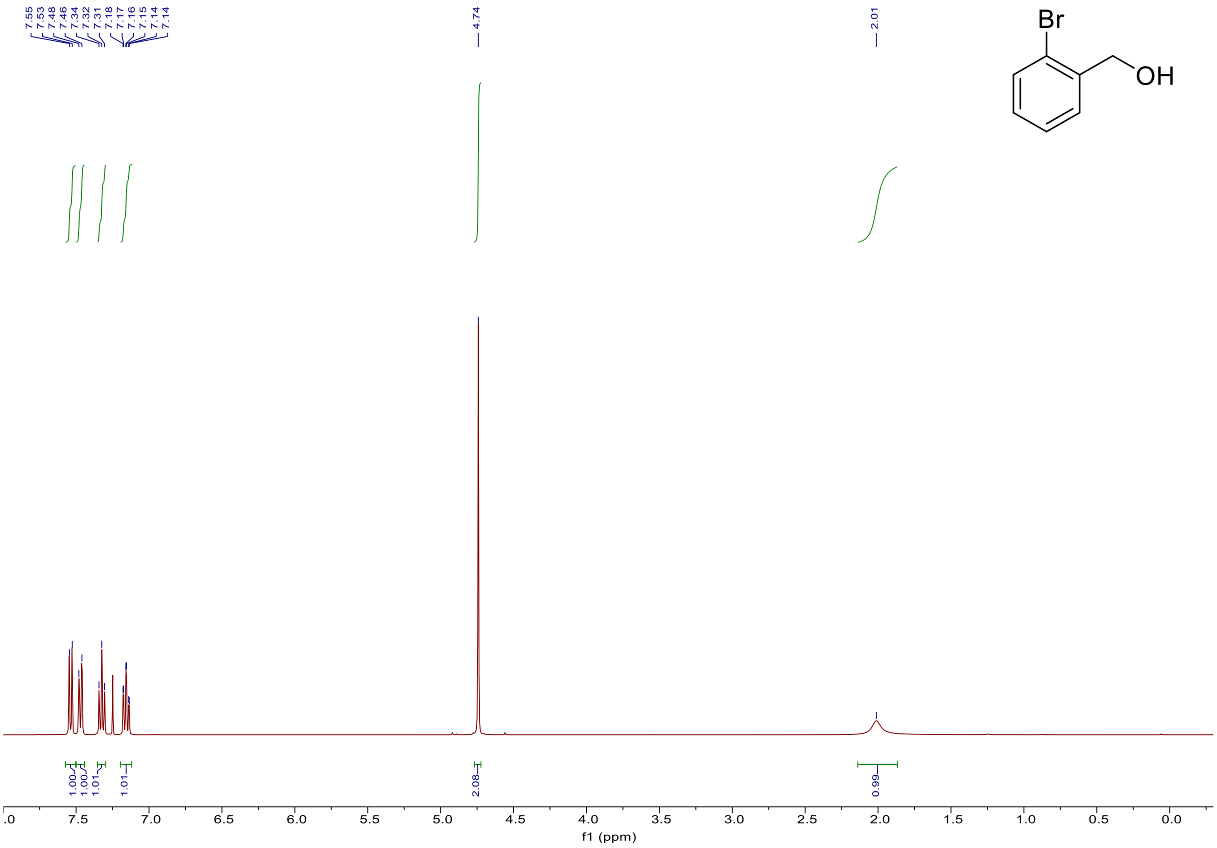

Figure S17 ^{1}H NMR of 2-bromobenzyl alcohol [S3] (2g)

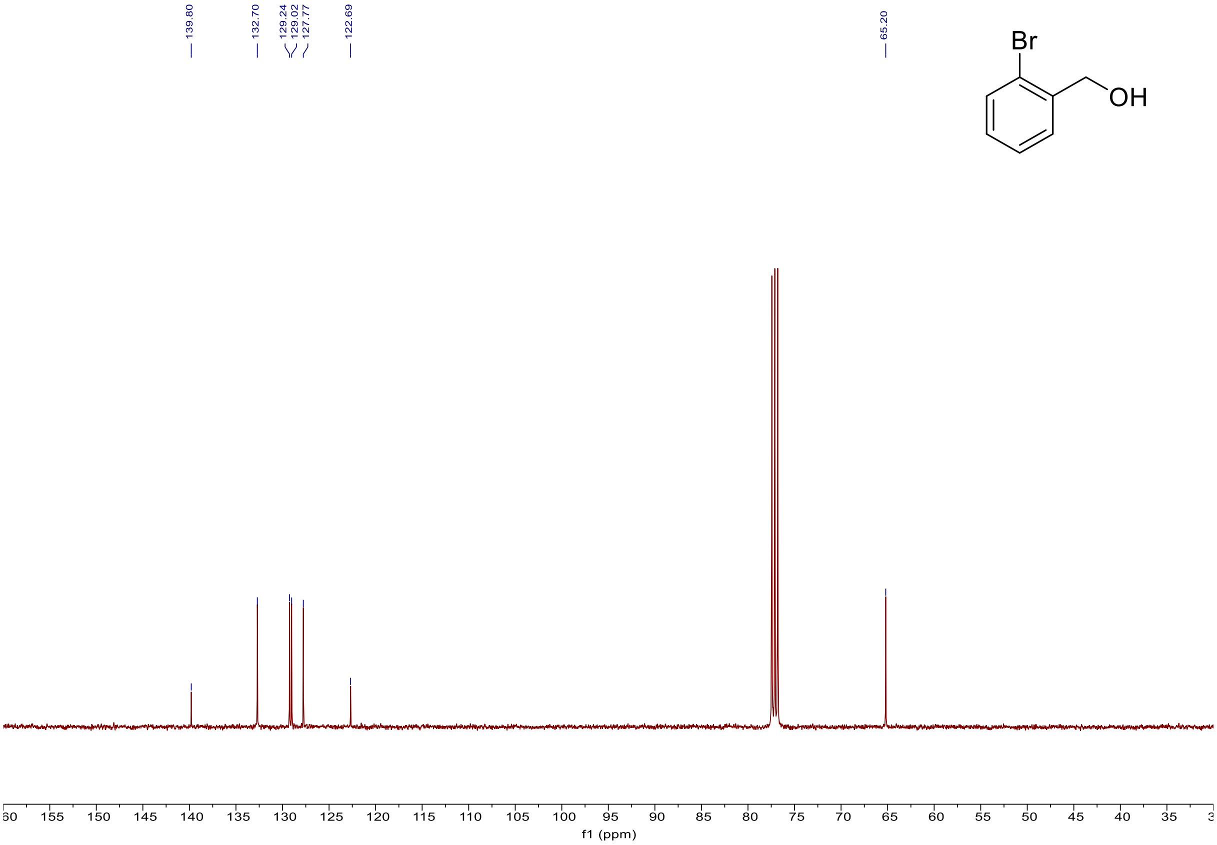

Figure S18 ^{13}C NMR of 2-bromobenzyl alcohol [S3] (2g)

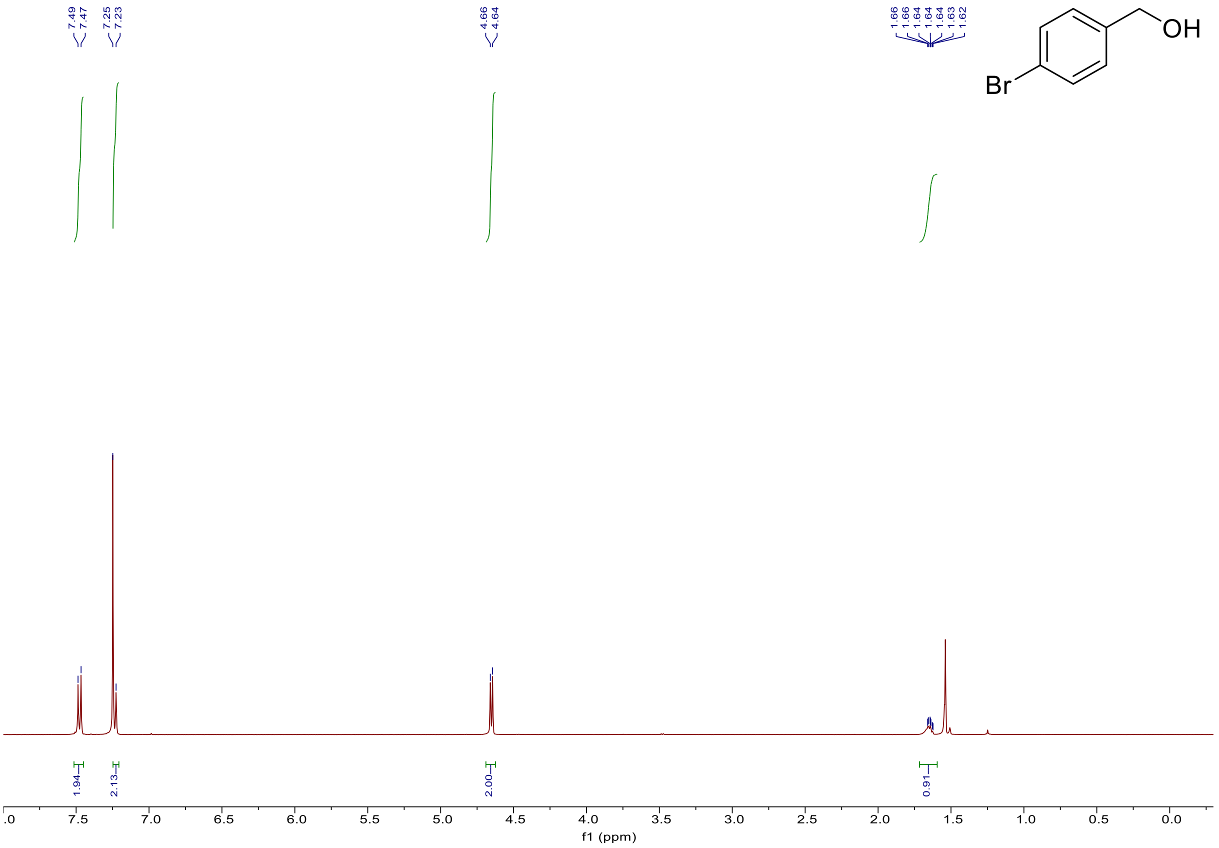

Figure S19 ^{1}H NMR of 4-bromobenzyl alcohol [S2] (2h)

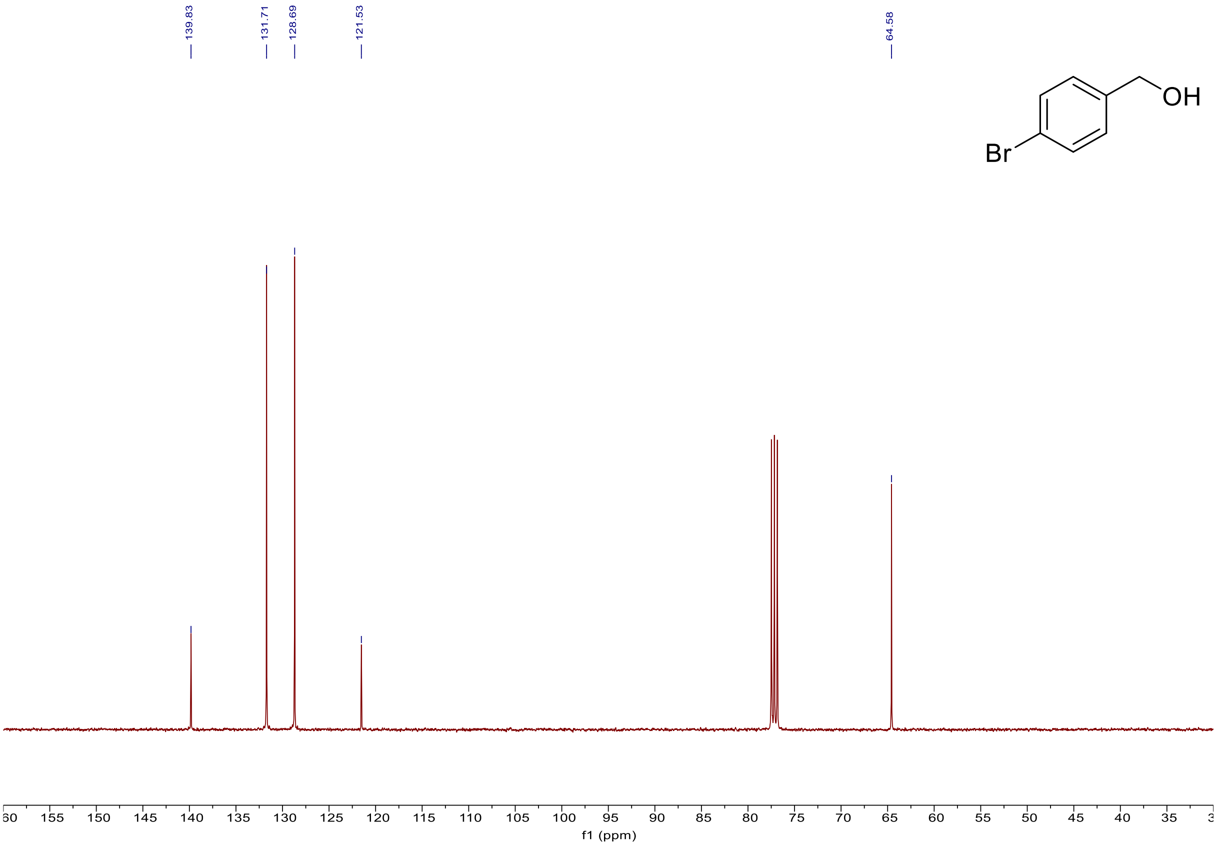

Figure S20 ^{13}C NMR of 4-bromobenzyl alcohol [S2] (2h)

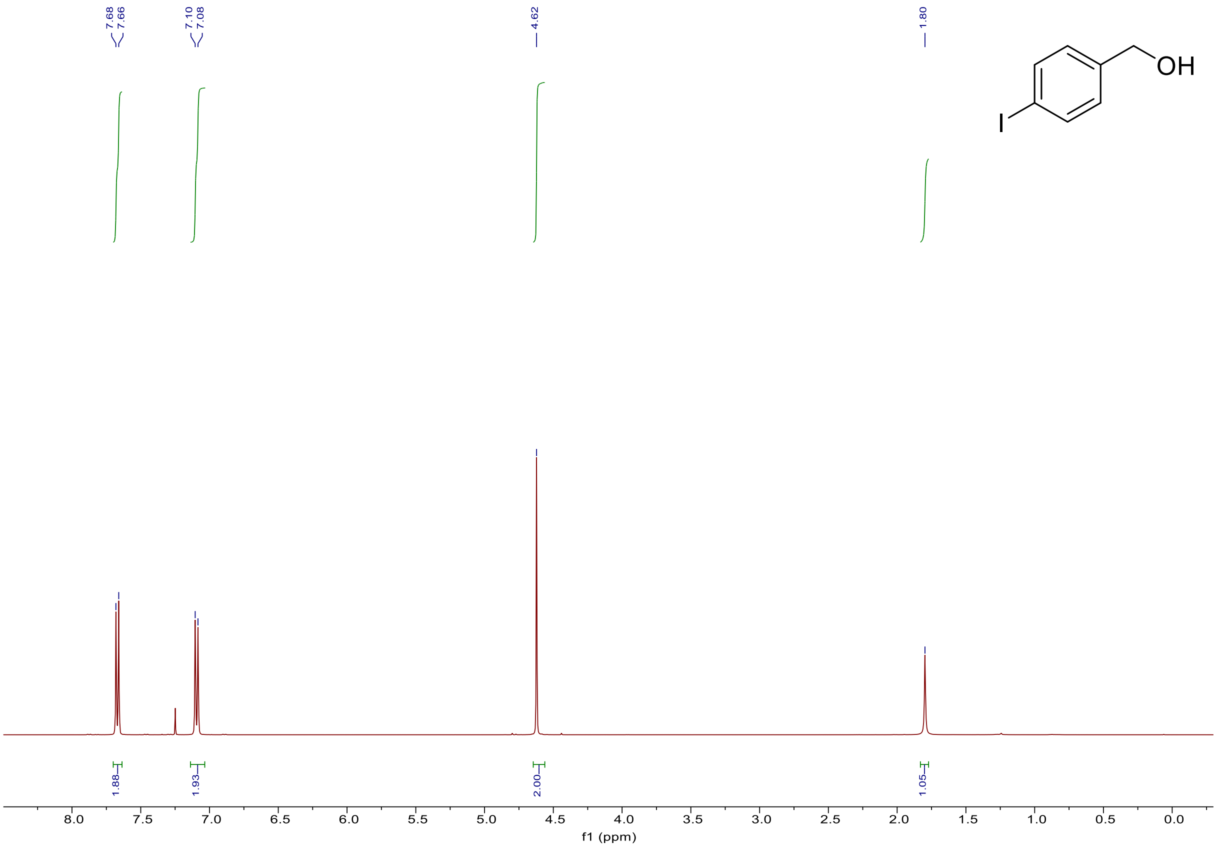

Figure S21 ^{1}H NMR of 4-iodobenzyl alcohol [S3](2i)

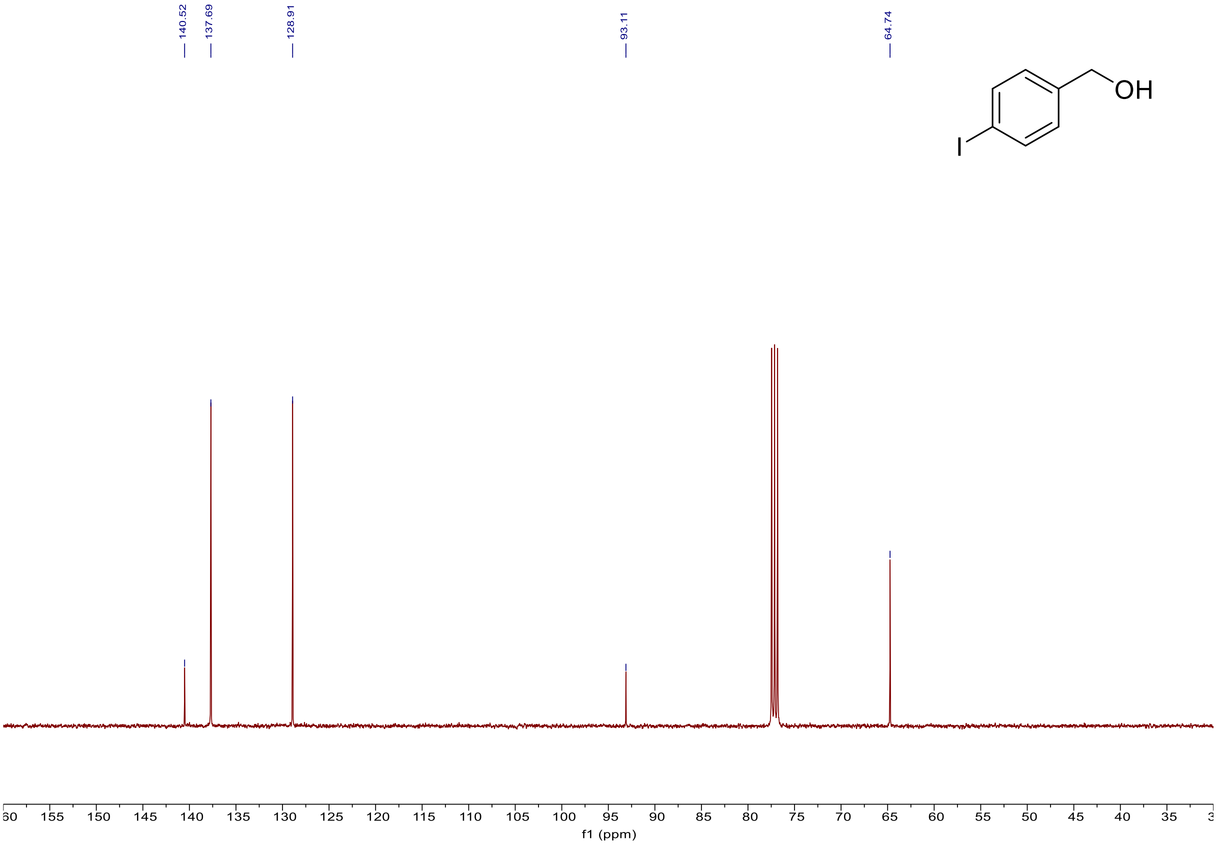

Figure S22 ^{13}C NMR of 4-iodobenzyl alcohol [S3](2i)

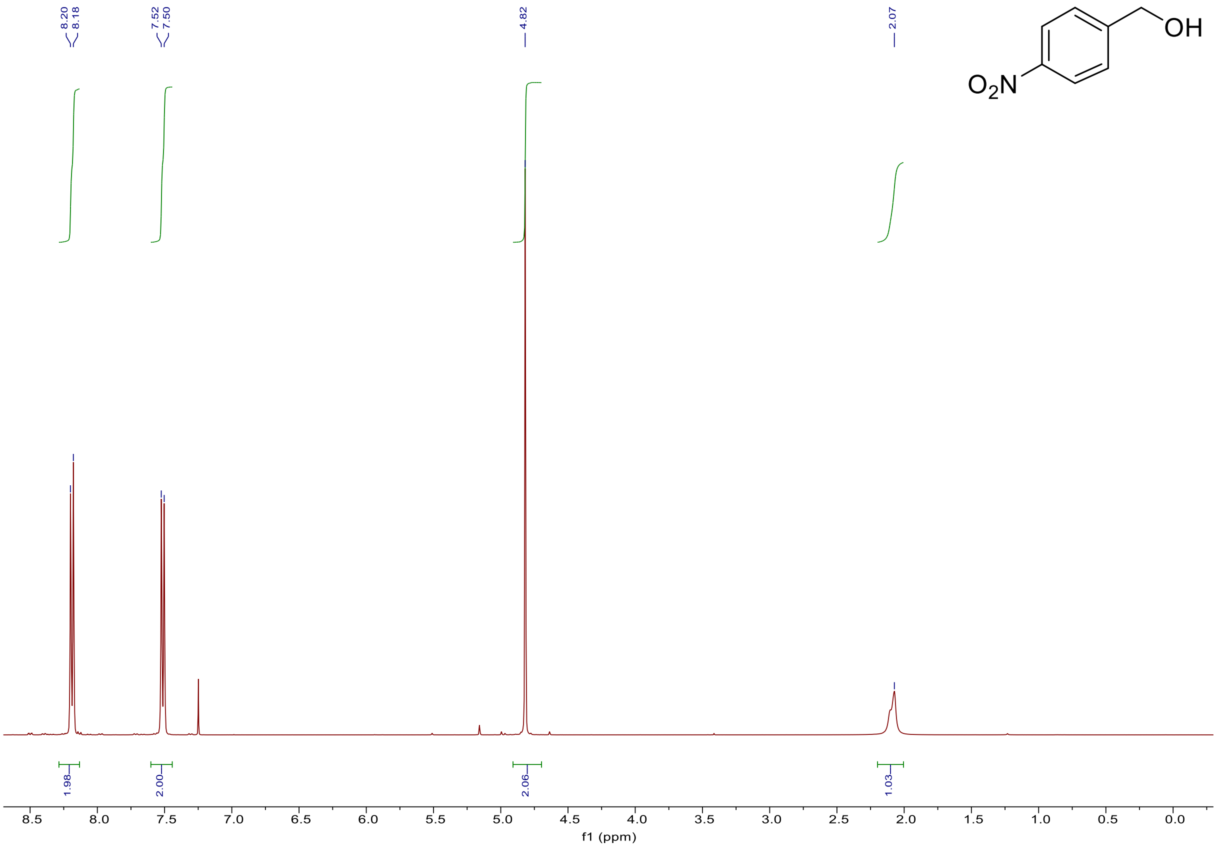

Figure S23 ^{1}H NMR of 4-nitrobenzyl alcohol [S2] (2j)

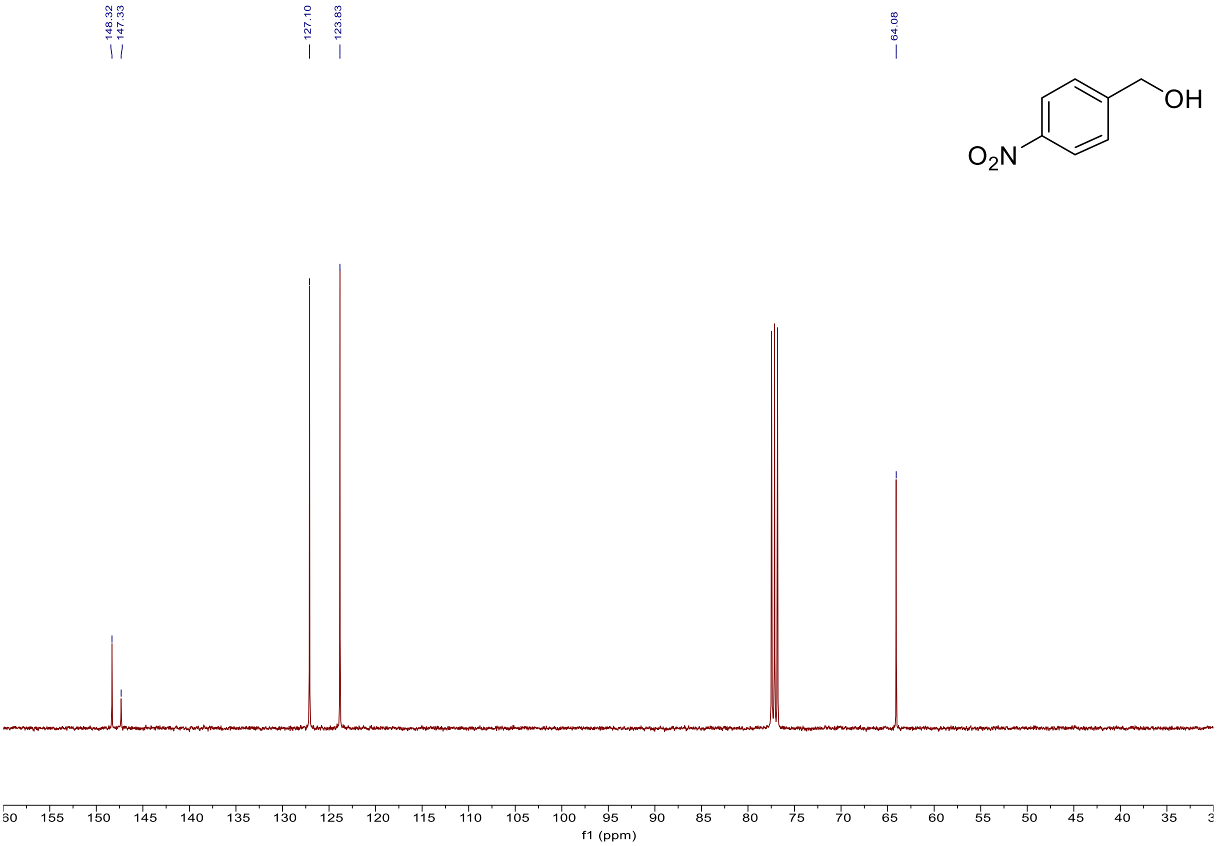

Figure S24 ^{13}C NMR of 4-nitrobenzyl alcohol [S2] (2j)

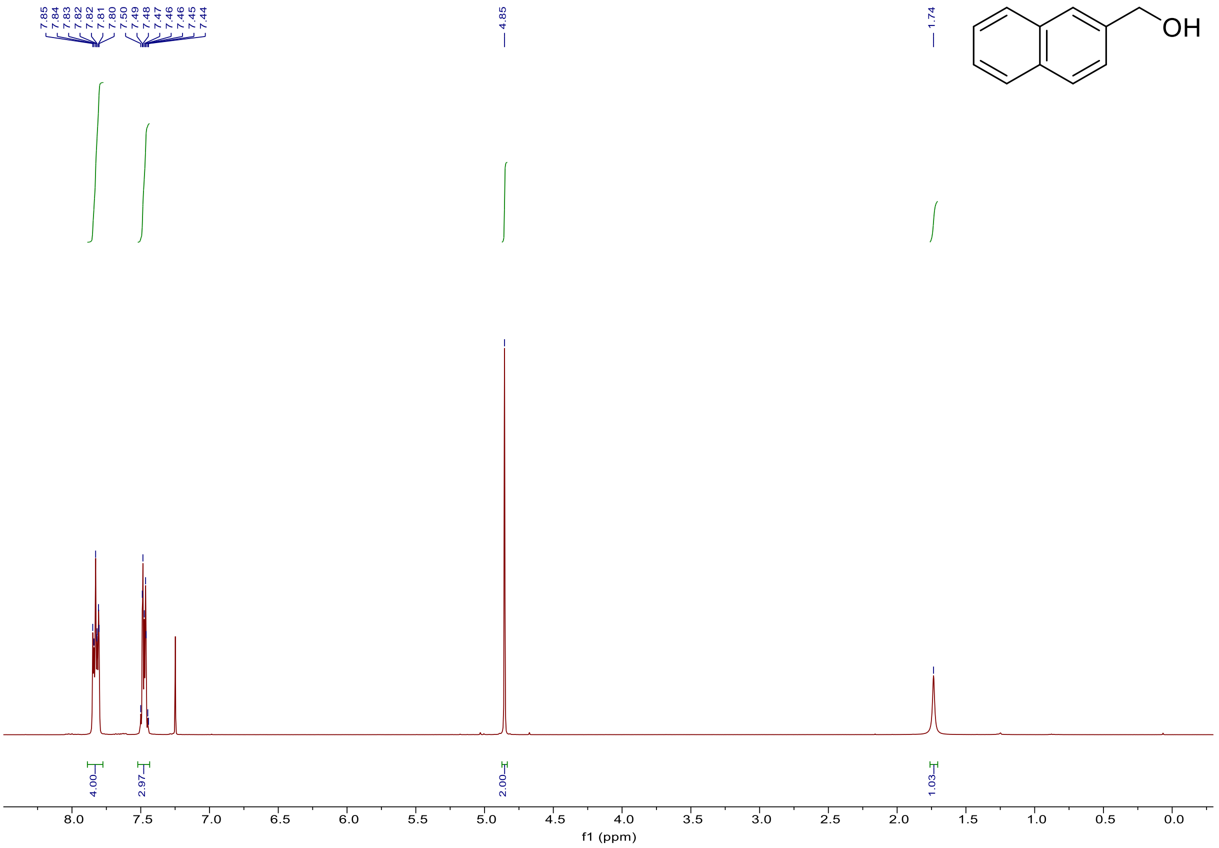

Figure S25 ^{1}H NMR of 2-naphthalenemethanol [S1](2k)

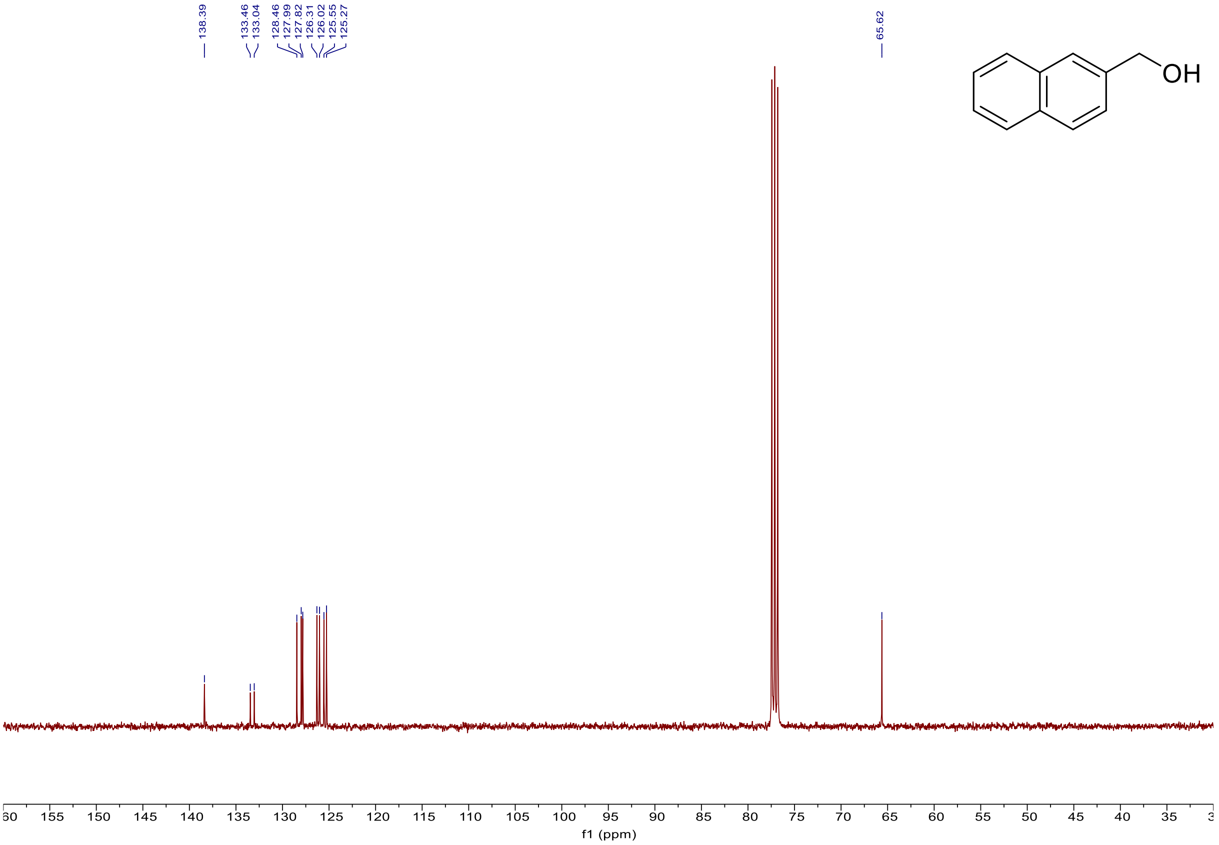

Figure S26 ^{13}C NMR of 2-naphthalenemethanol [S1](2k)

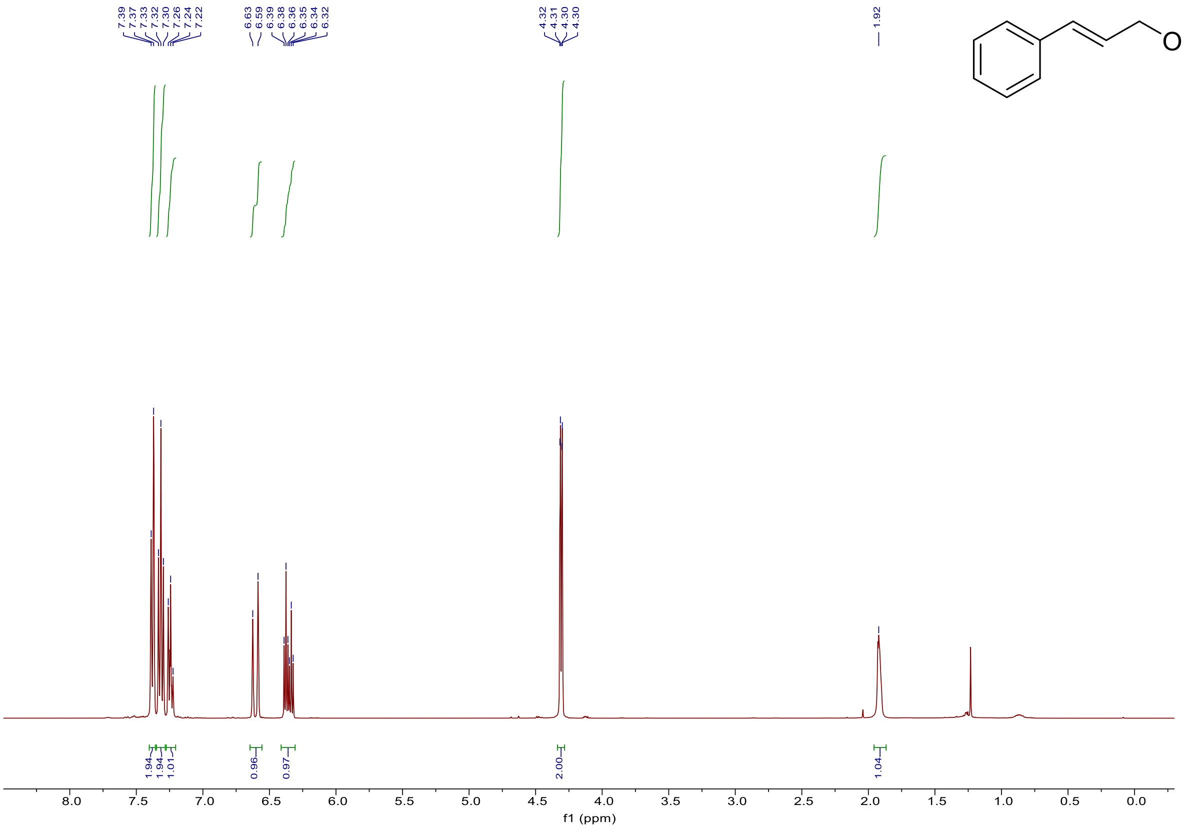

Figure S27 ^{1}H NMR of cinnamyl alcohol [S4] (2l)

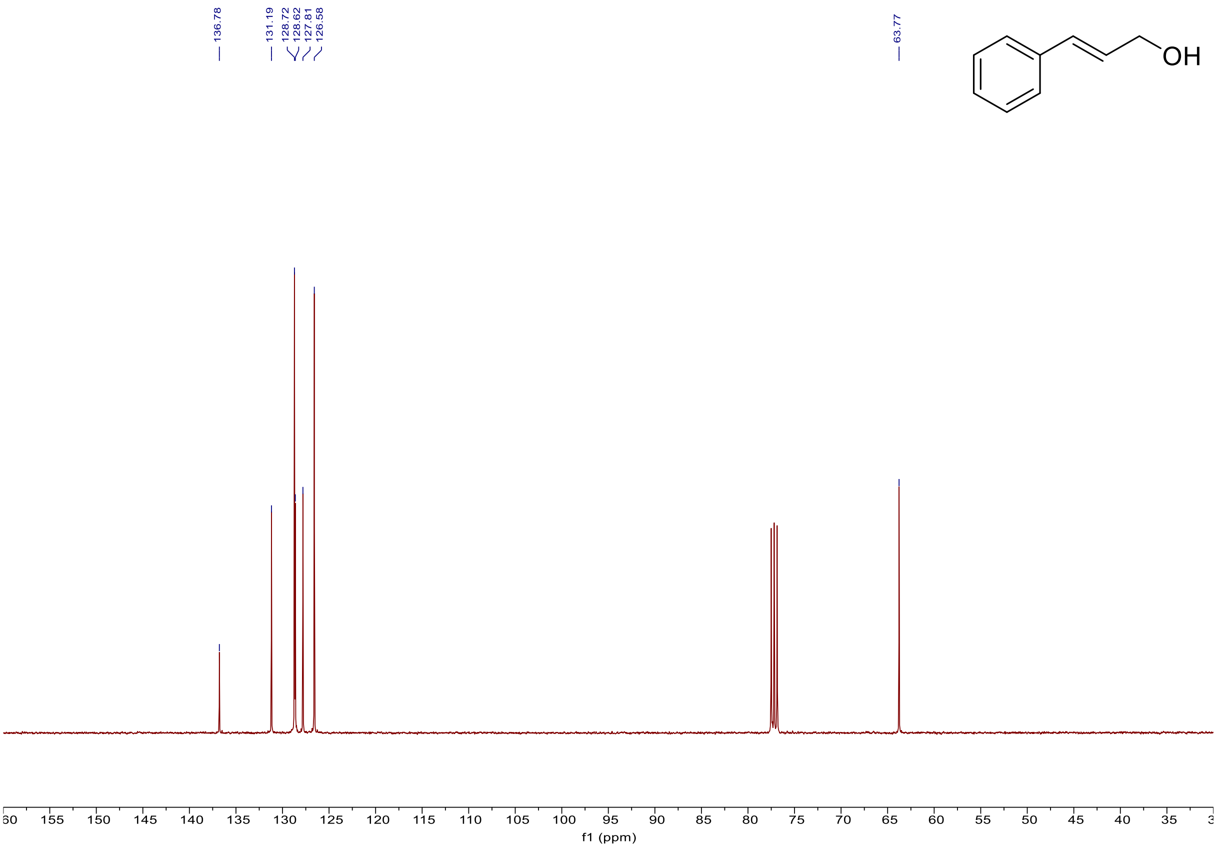

Figure S28 ^{13}C NMR of cinnamyl alcohol [S4] (2l)

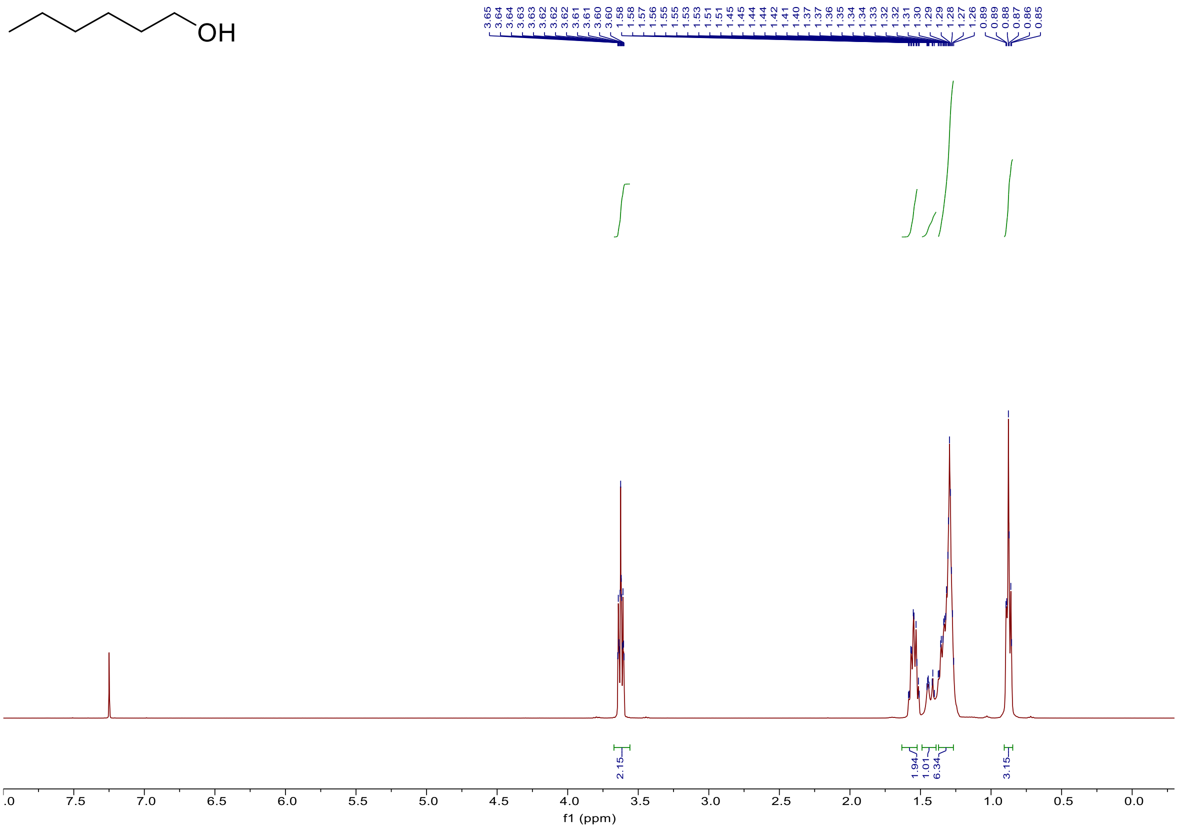

Figure S29 ^{1}H NMR of hexanol [S4] (2m)

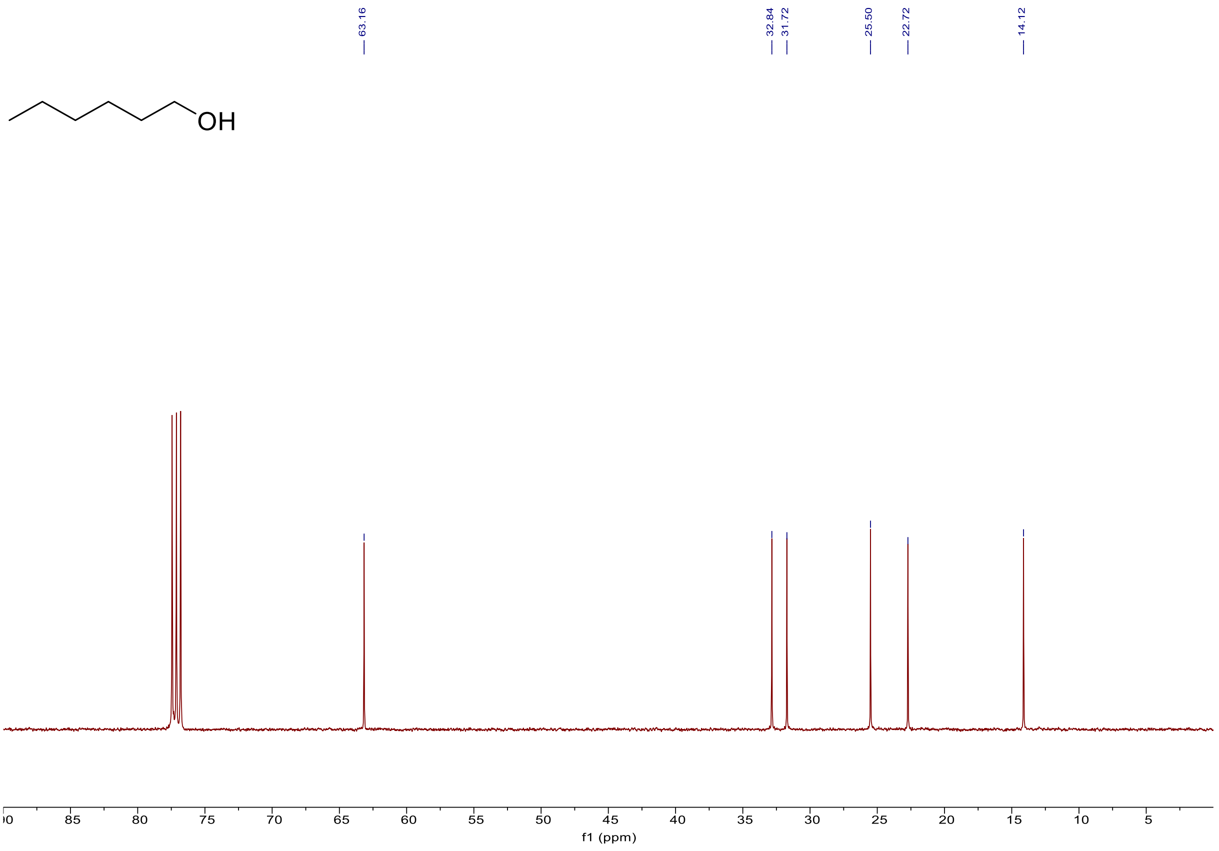

Figure S30 ^{13}C NMR of hexanol [S4] (2m)

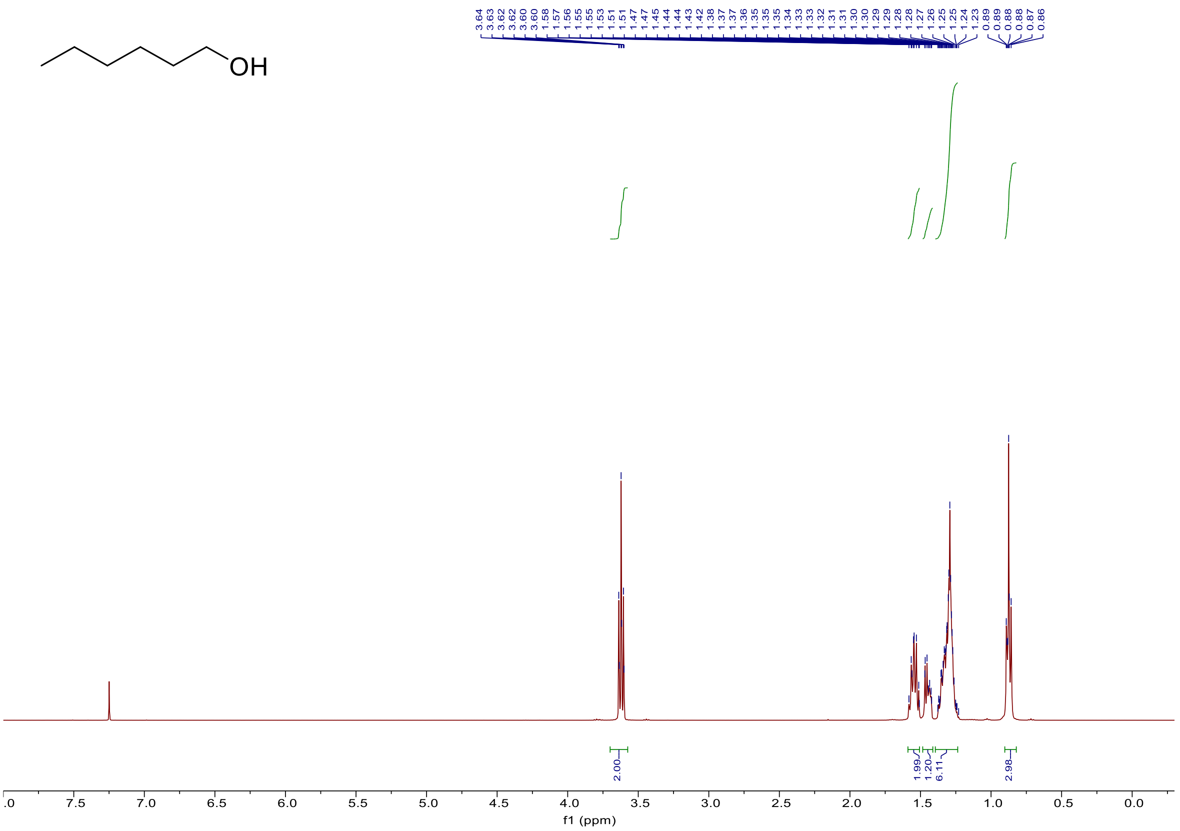

Figure S31 ^{1}H NMR of hexanol [S4] (2m')

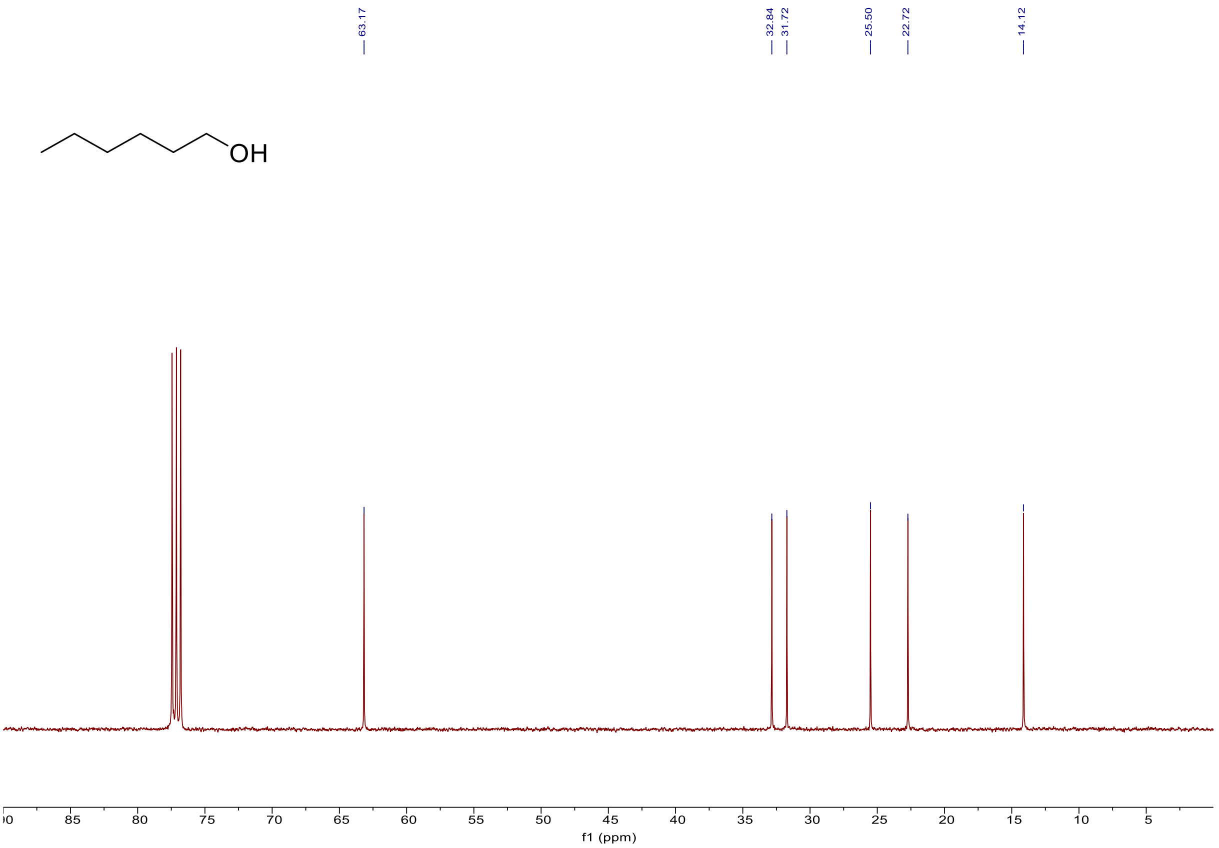

Figure S32 ^{13}C NMR of hexanol [S4] (2m')

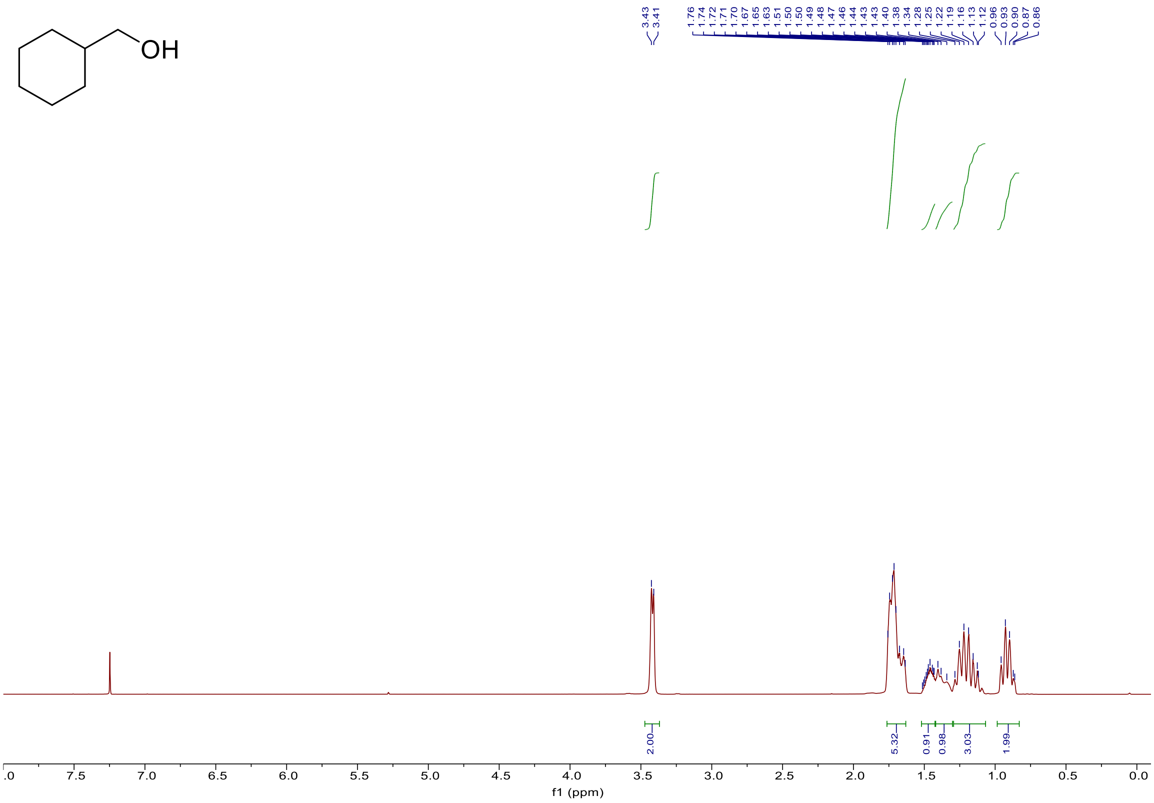

Figure S33 ^{1}H NMR of Cyclohexyl methanol [S4] (2n)

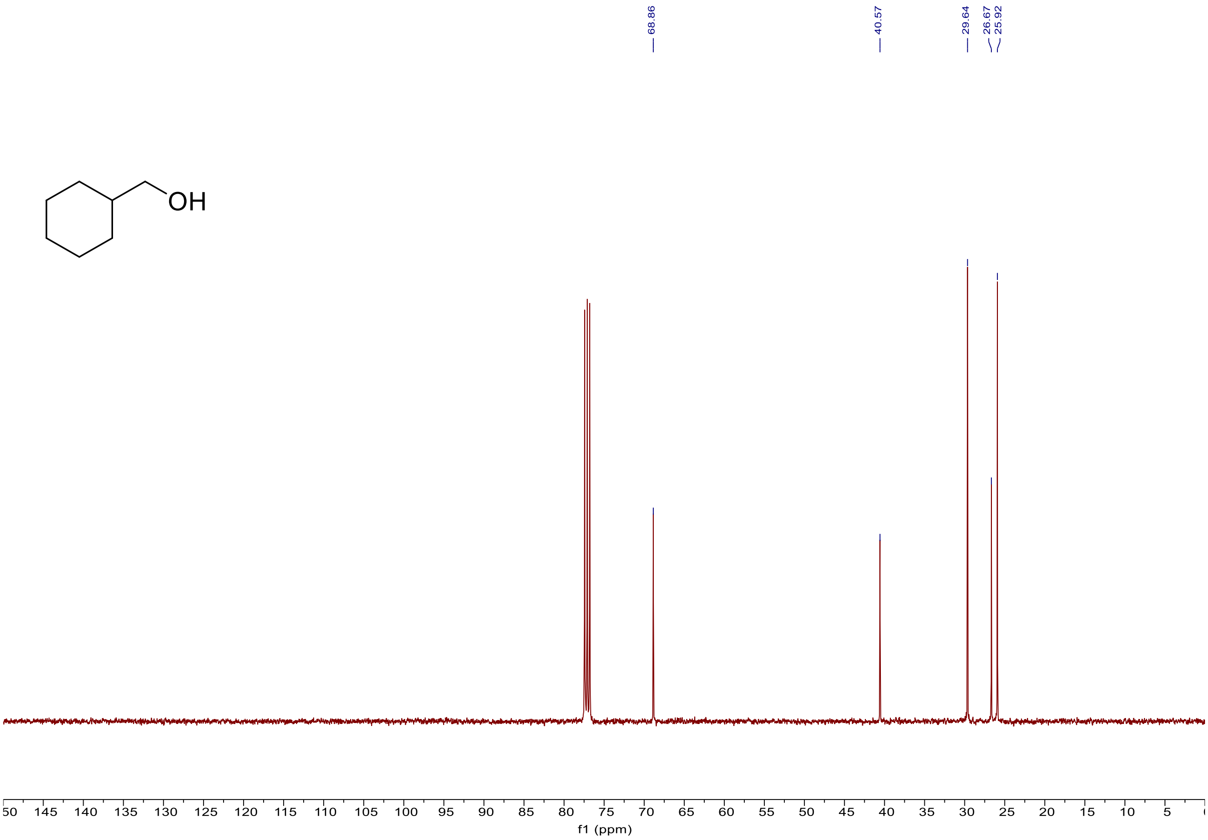

Figure S34 ^{13}C NMR of Cyclohexyl methanol [S4] (2n)

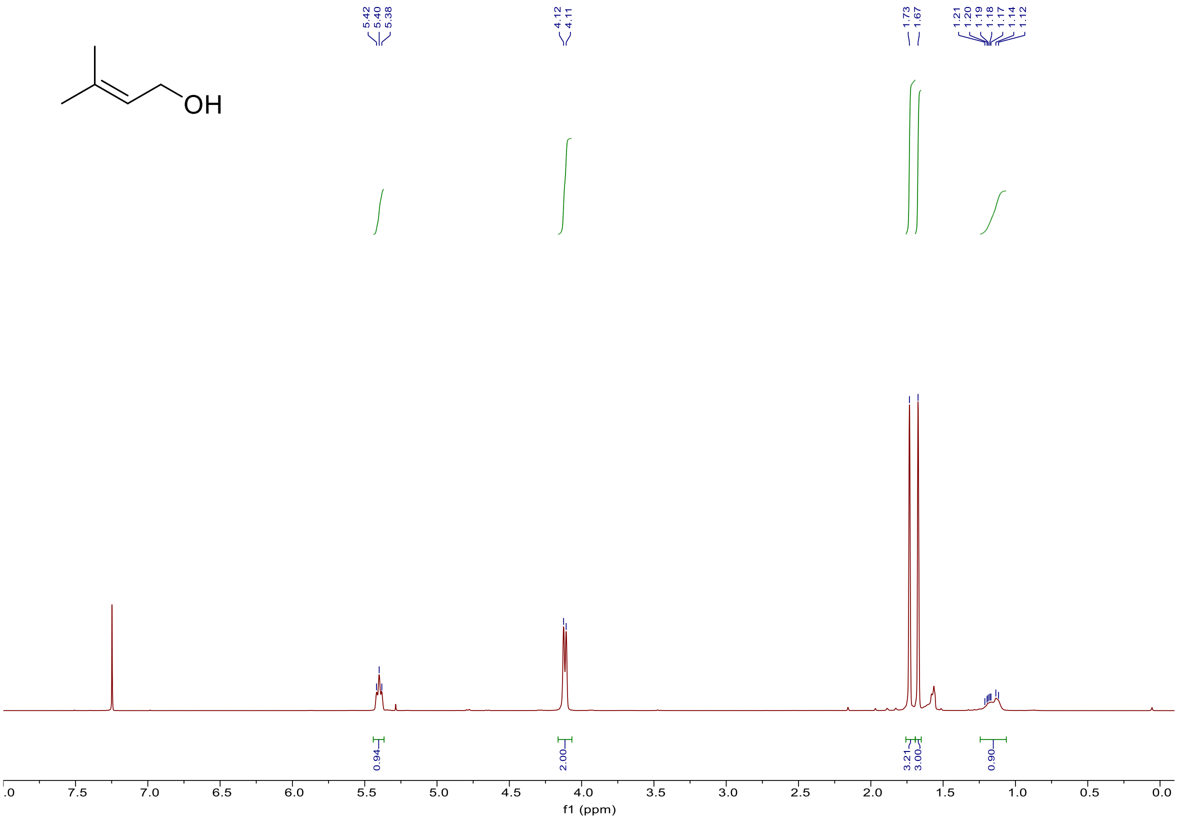

Figure S35 ^{1}H NMR of 3-Methyl-2-buten-1-ol [S5] (2o)

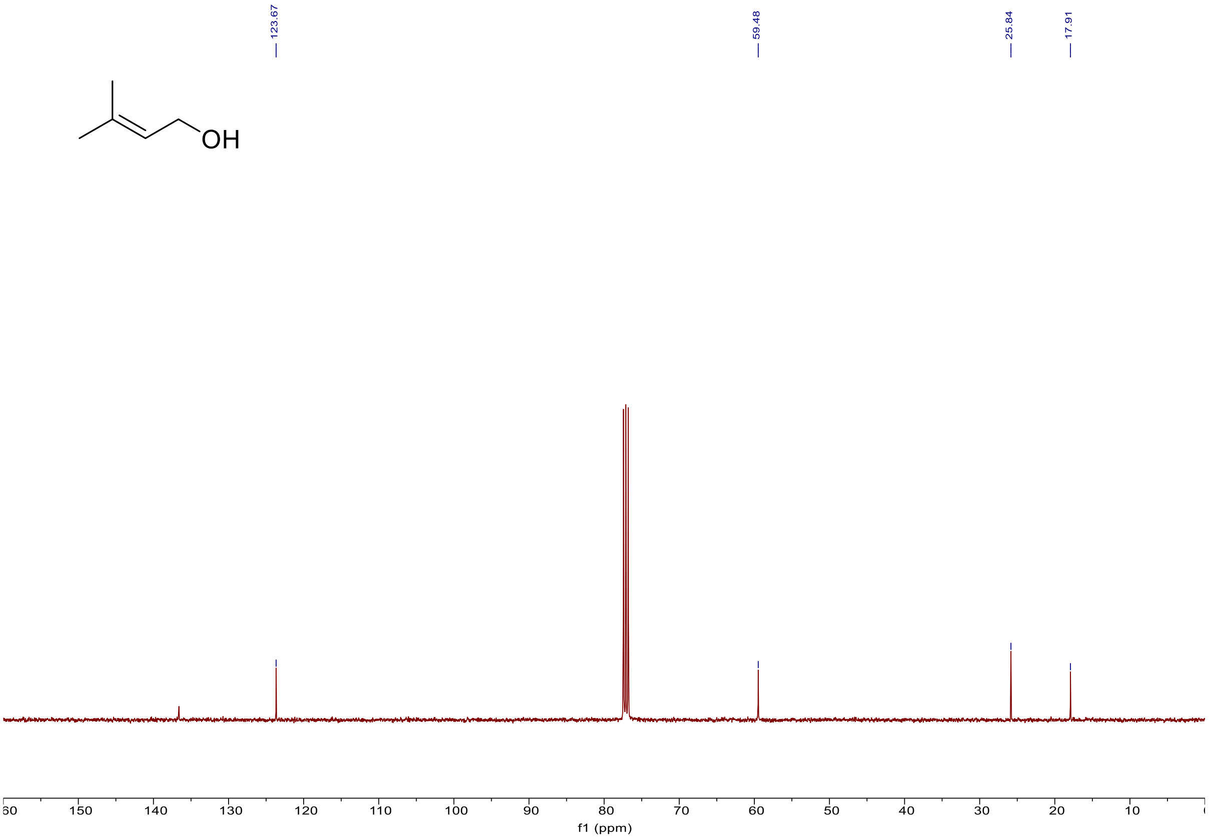

Figure S36 ^{13}C NMR of 3-Methyl-2-buten-1-ol [S5] (2o)

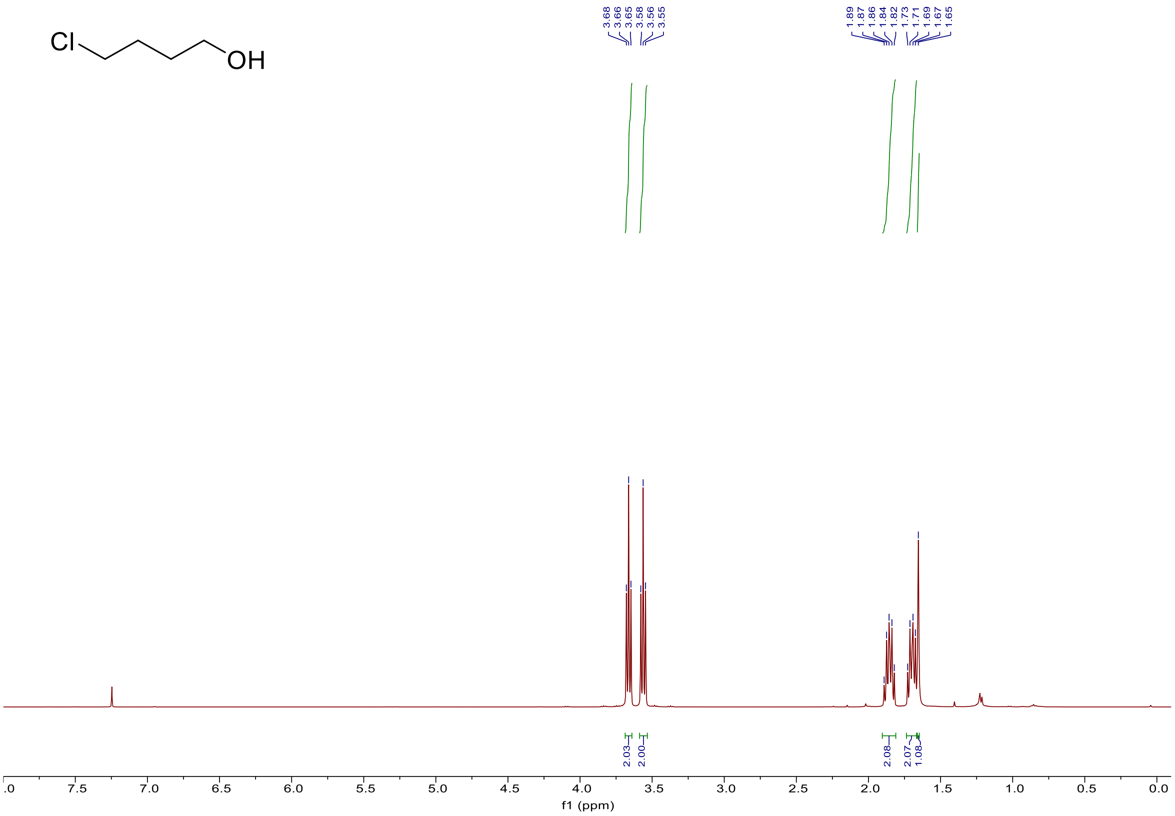

Figure S37 ^{1}H NMR of 4-Chlorobutan-1-ol [S6] (2p)

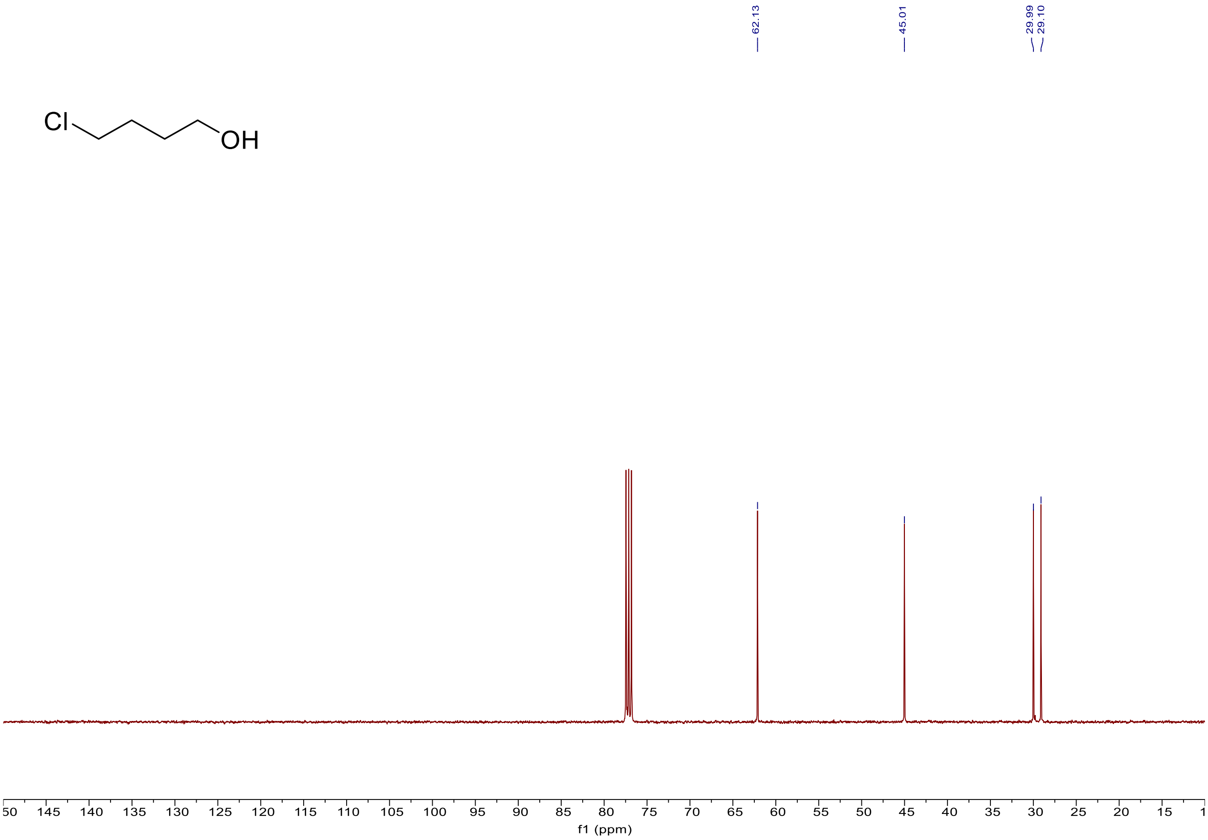

Figure S38 ^{13}C NMR of 4-Chlorobutan-1-ol [S6] (2p)

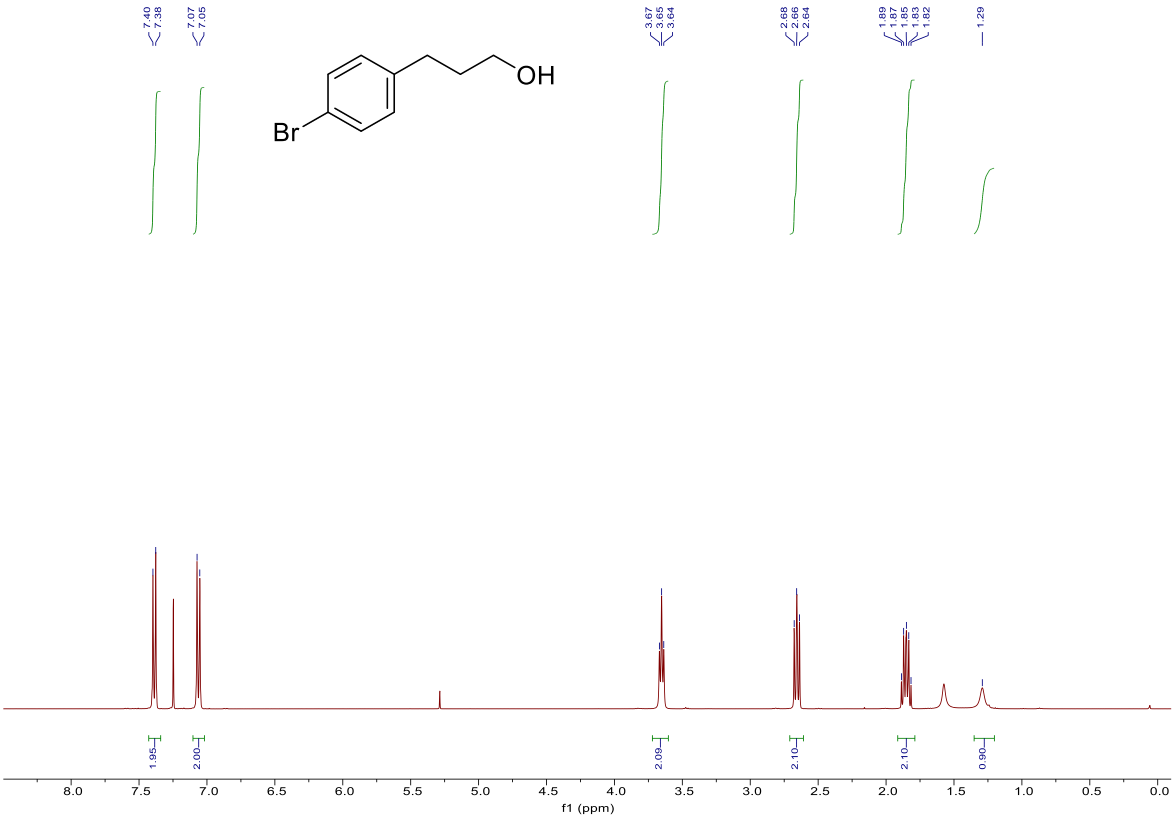

Figure S39 ^{1}H NMR of 3-(4-Bromophenyl)propan-1-ol [S7] (2q)

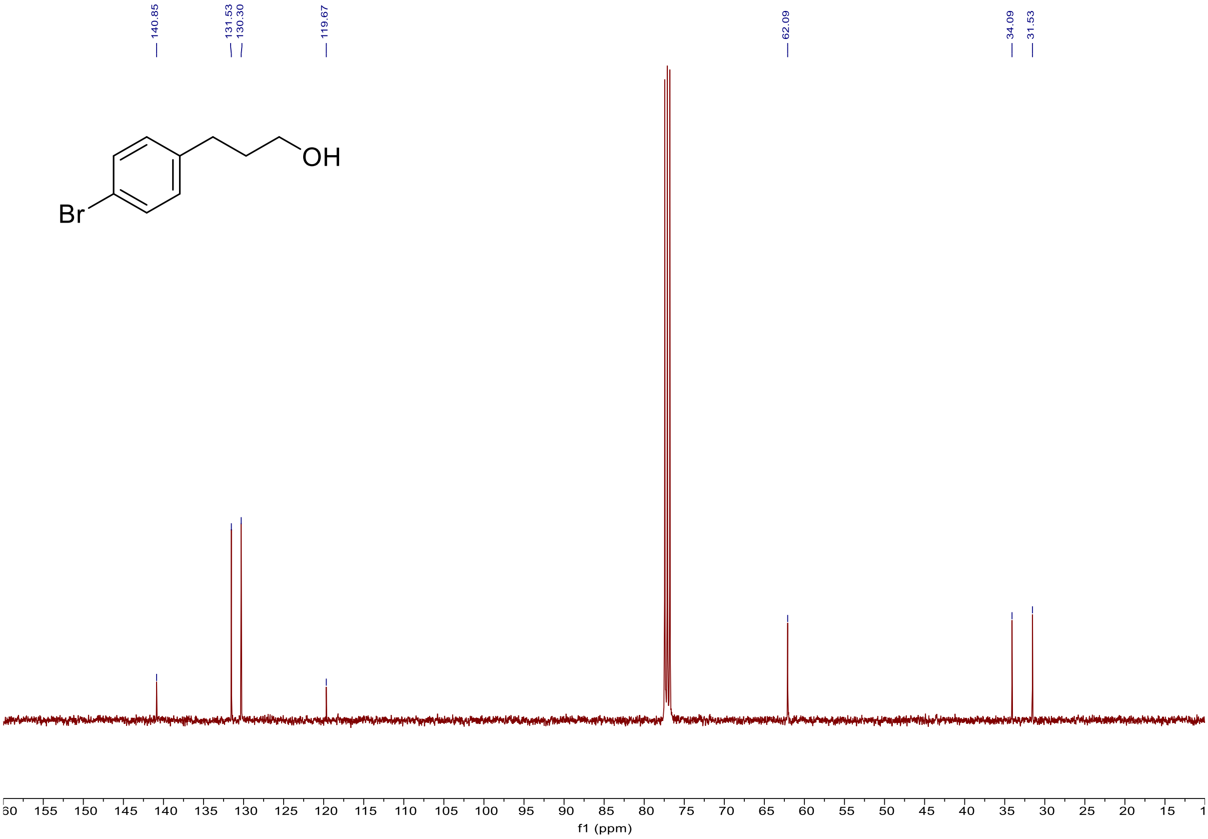

Figure S40 ^{13}C NMR of 3-(4-Bromophenyl)propan-1-ol [S7](2q)

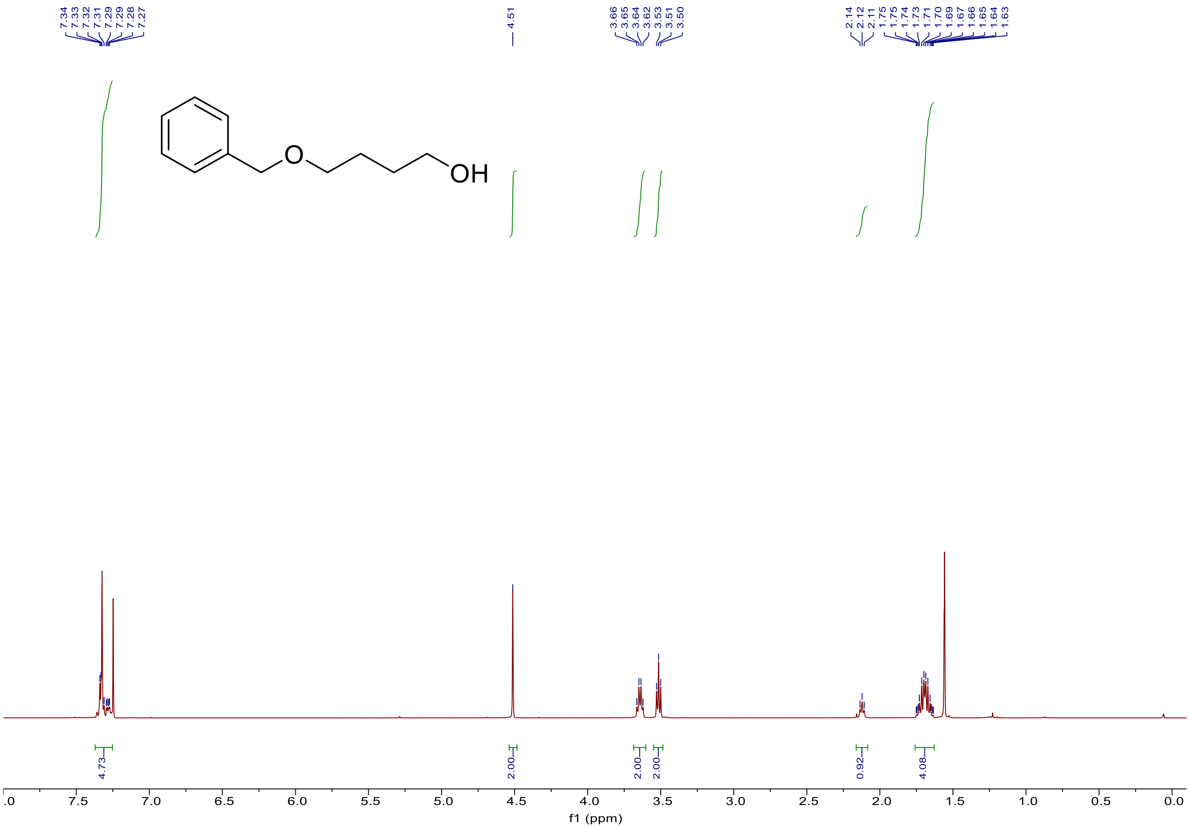

Figure S41 ^{1}H NMR of 4-(benzyloxy)butan-1-ol **S8** (2r)

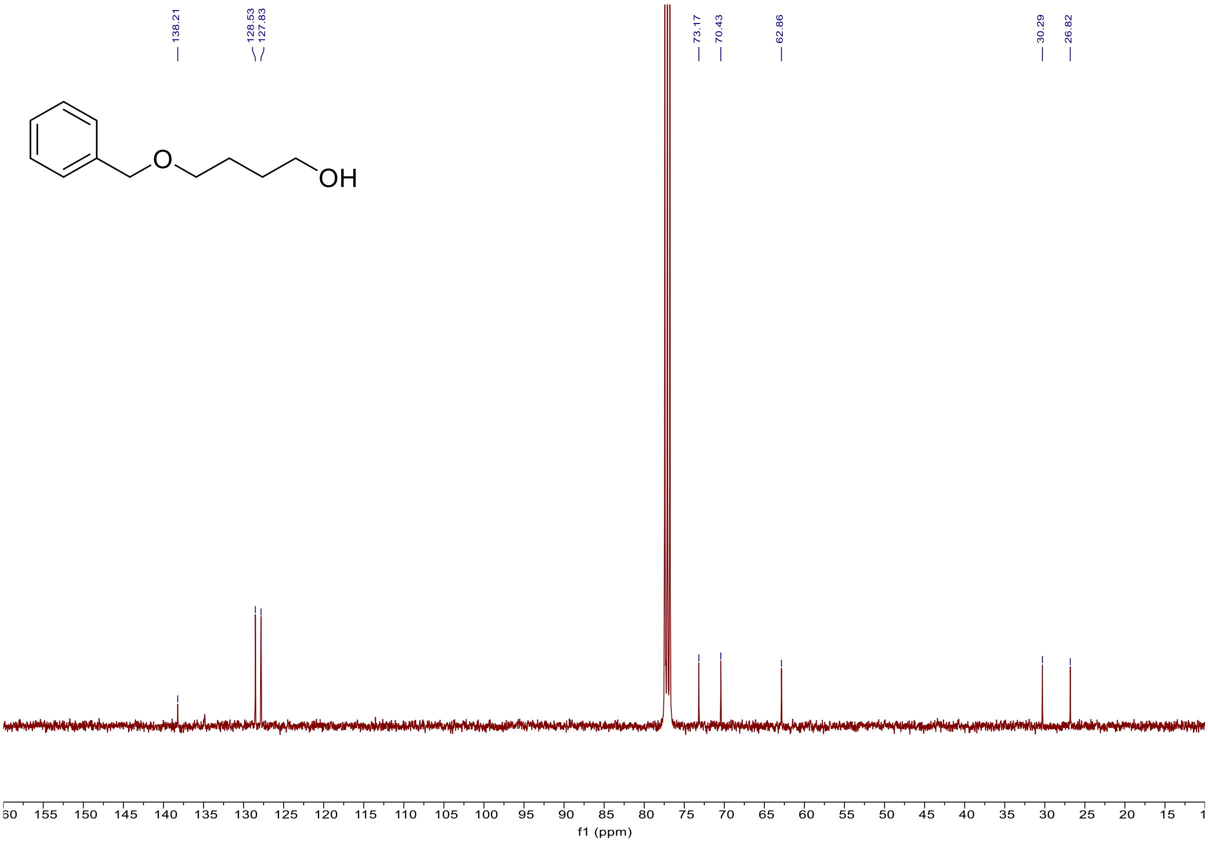

Figure S42 ^{13}C NMR of 4-(benzyloxy)butan-1-ol **S8** (2r)

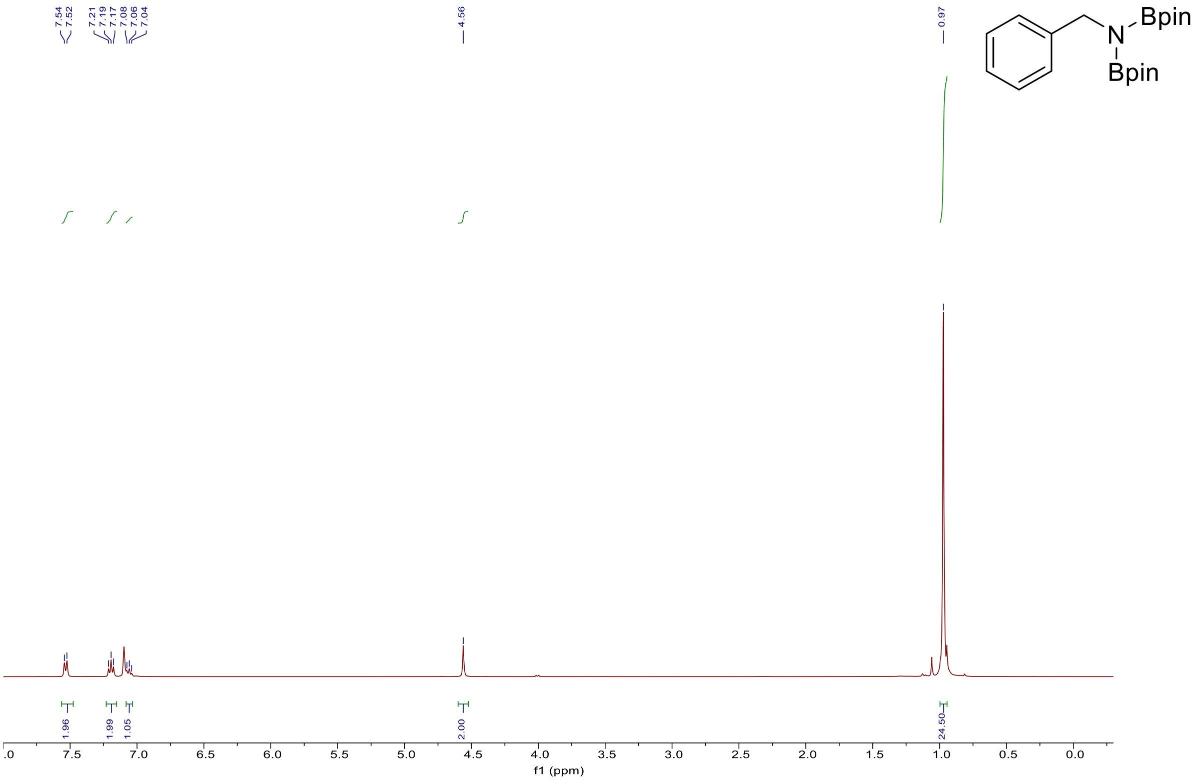

Figure S43 ^{1}H NMR of *N*-benzyl-4,4,5,5-tetramethyl-*N*-(4,4,5,5-tetramethyl-1,3,2-dioxaborolan-2-yl)-1,3,2 dioxaborolan-2- amine [S9] (4a)

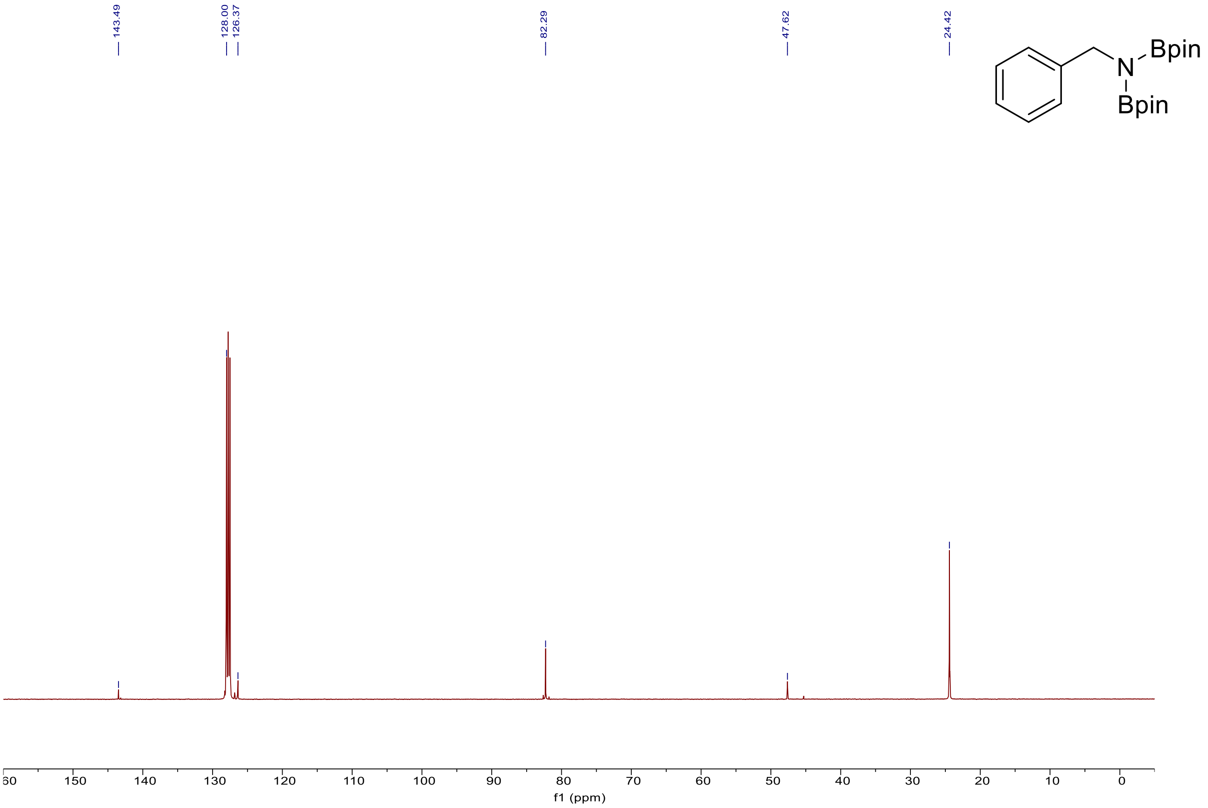

Figure S44 ^{13}C NMR of *N*-benzyl-4,4,5,5-tetramethyl-*N*-(4,4,5,5-tetramethyl-1,3,2-dioxaborolan-2-yl)-1,3,2 dioxaborolan-2- amine [S9] (4a)

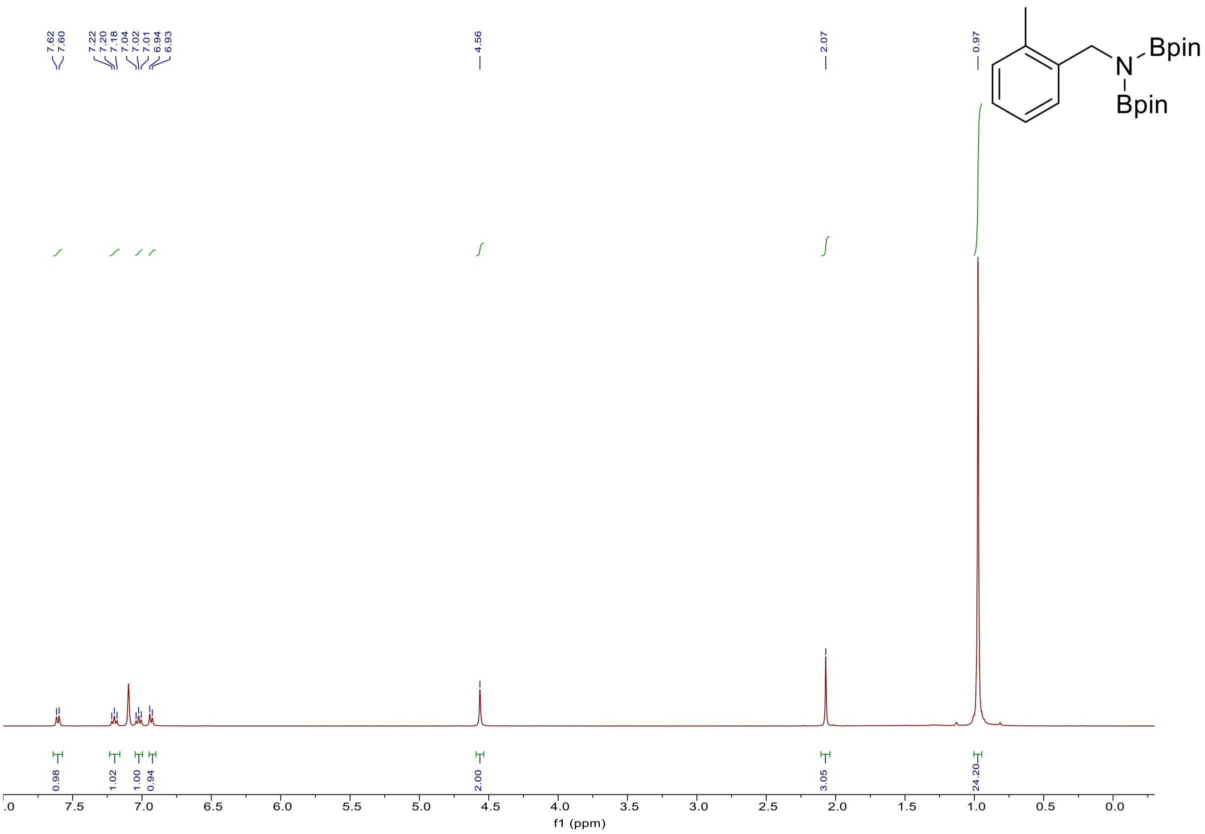

Figure S45 ^{1}H NMR of 4,4,5,5-Tetramethyl-*N*-(2-methylbenzyl)-*N*-(4,4,5,5-tetramethyl-1,3,2-dioxaborolan-2-yl)-1,3,2- dioxaborolan-2-amine [S9] (4b)

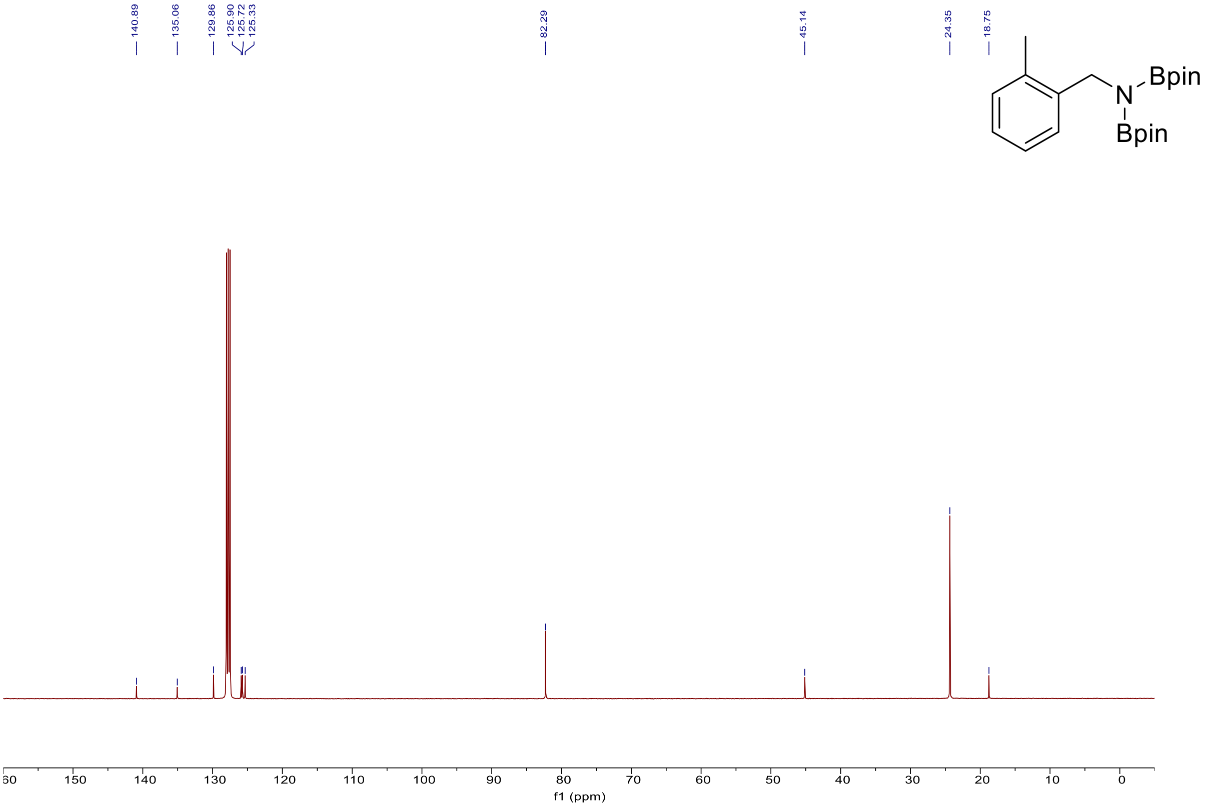

Figure S46 ^{13}C NMR of 4,4,5,5-Tetramethyl-*N*-(2-methylbenzyl)-*N*-(4,4,5,5-tetramethyl-1,3,2-dioxaborolan-2-yl)-1,3,2- dioxaborolan-2-amine [S9] (4b)

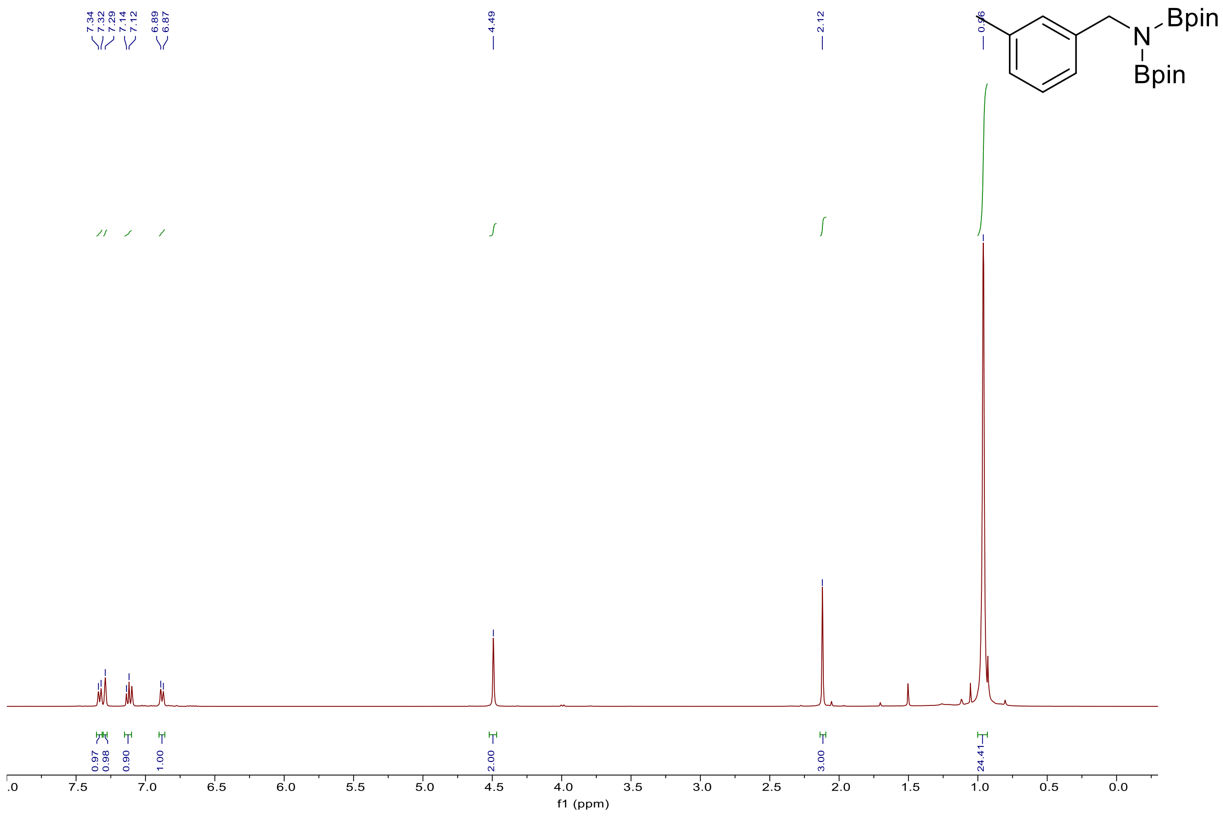

Figure S47 ^{1}H NMR of 4,4,5,5-tetramethyl-*N*-(3-methylbenzyl)-*N*-(1,4,4,5,5-pentamethyl-1l3,3,2-dioxaborolan-2-yl)-1,3,2-dioxaborolan-2-amine [S10] (4c)

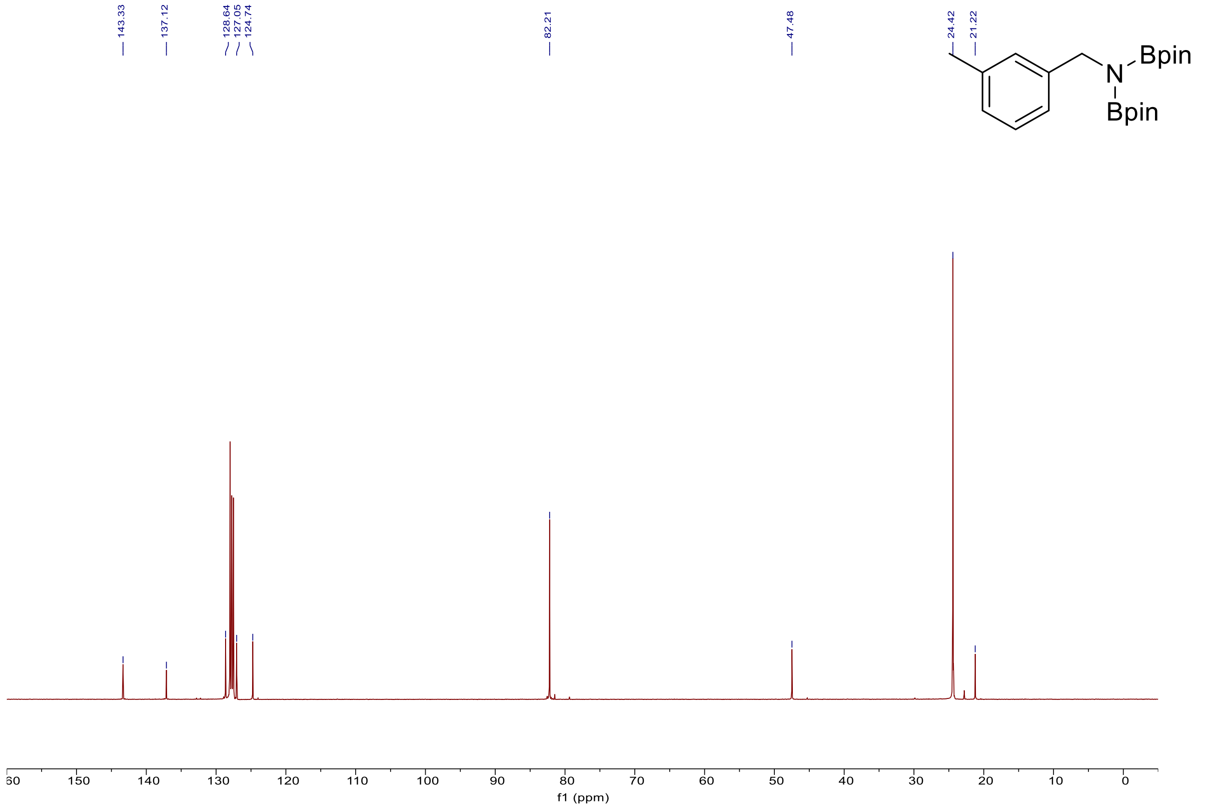

Figure S48 ^{13}C NMR of 4,4,5,5-tetramethyl-*N*-(3-methylbenzyl)-*N*-(1,4,4,5,5-pentamethyl-1l3,3,2-dioxaborolan-2-yl)-1,3,2-dioxaborolan-2-amine [S10] (4c)

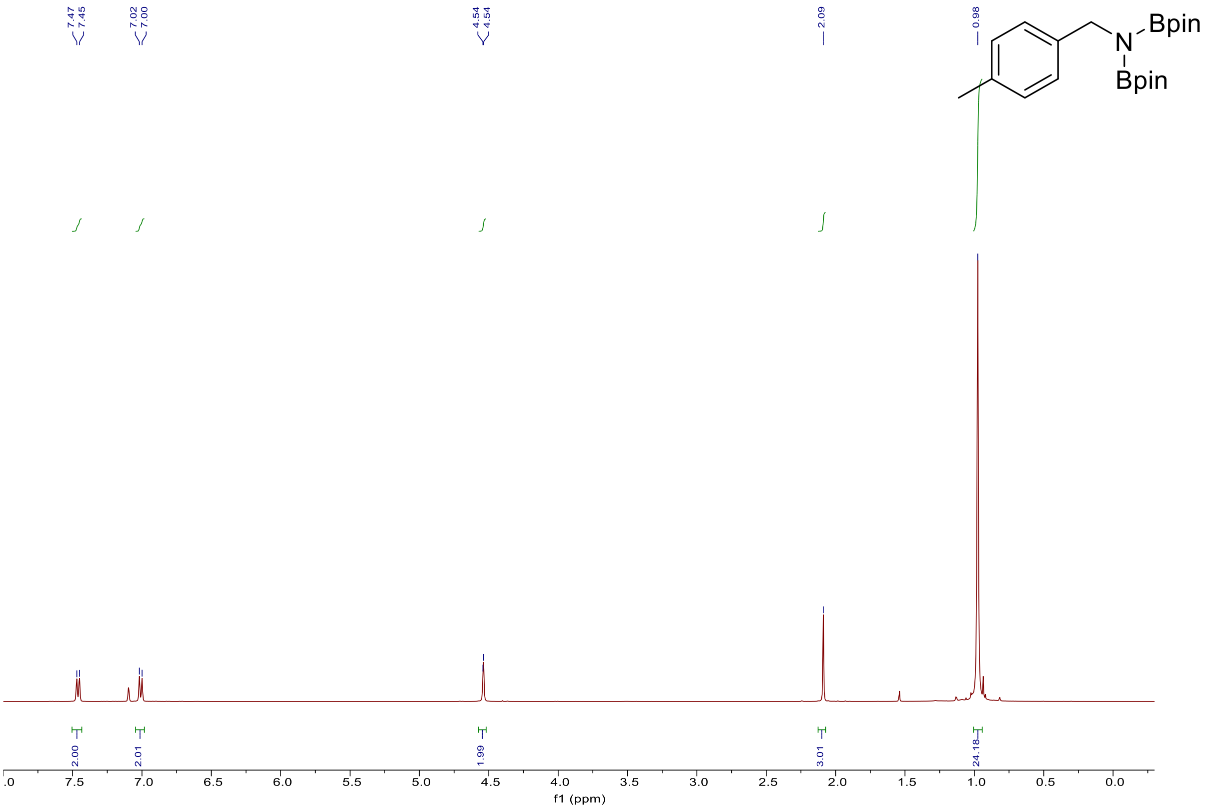

Figure S49 ^{1}H NMR of 4,4,5,5-Tetramethyl-*N*-(4-methylbenzyl)-*N*-(4,4,5,5-tetramethyl-1,3,2-dioxaborolan-2-yl)-1,3,2- dioxaborolan-2-amine [S9] (4d)

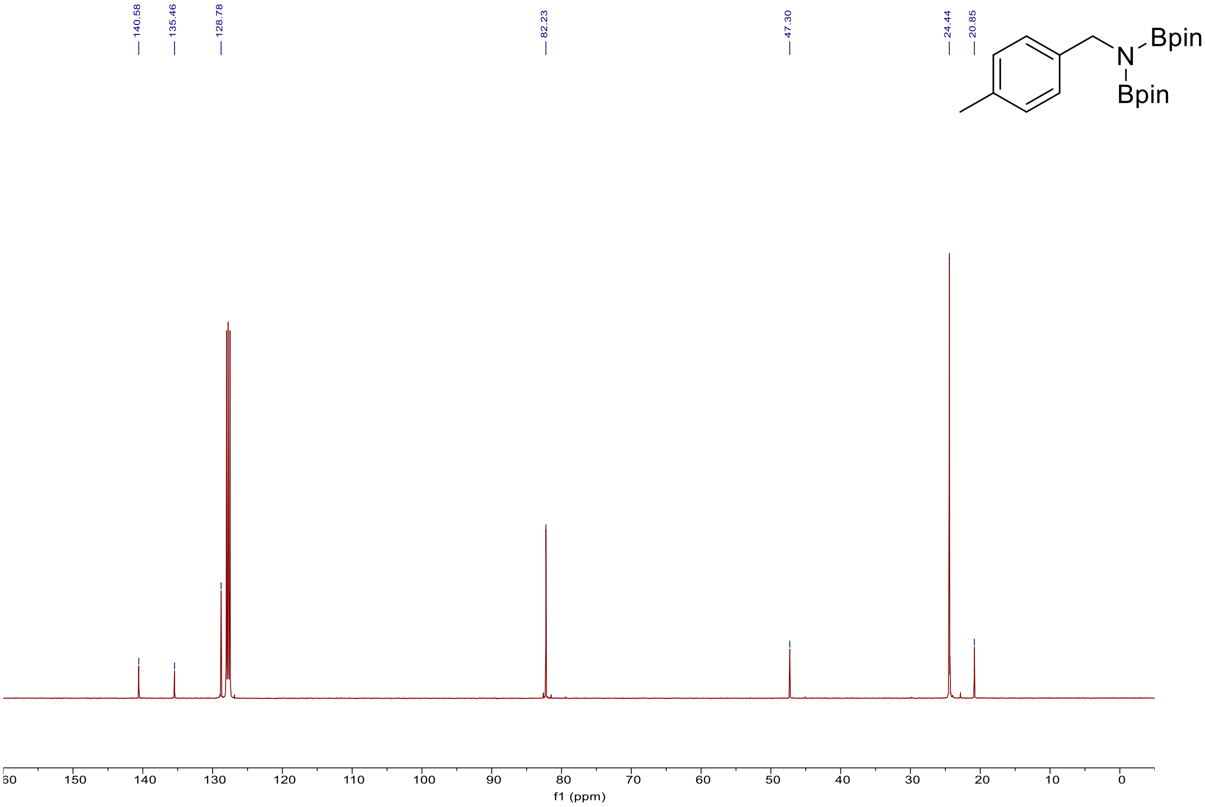

Figure S50 ^{13}C NMR of 4,4,5,5-Tetramethyl-*N*-(4-methylbenzyl)-*N*-(4,4,5,5-tetramethyl-1,3,2-dioxaborolan-2-yl)-1,3,2- dioxaborolan-2-amine [S9] (4d)

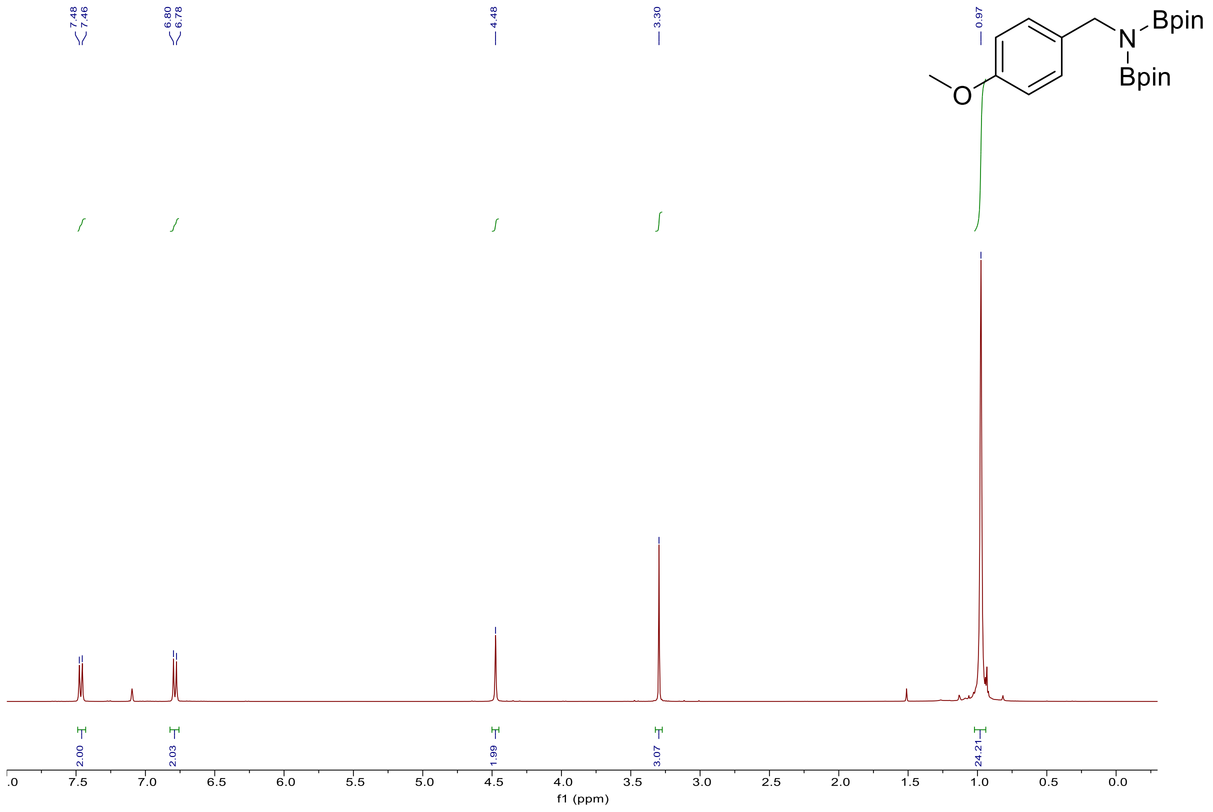

Figure S51 ^{1}H NMR of *N*-(4-methoxybenzyl)-4,4,5,5-tetramethyl-*N*-(4,4,5,5-tetramethyl-1,3,2-dioxaborolan-2-yl)-1,3,2- dioxaborolan-2-amine [S9] (4e)

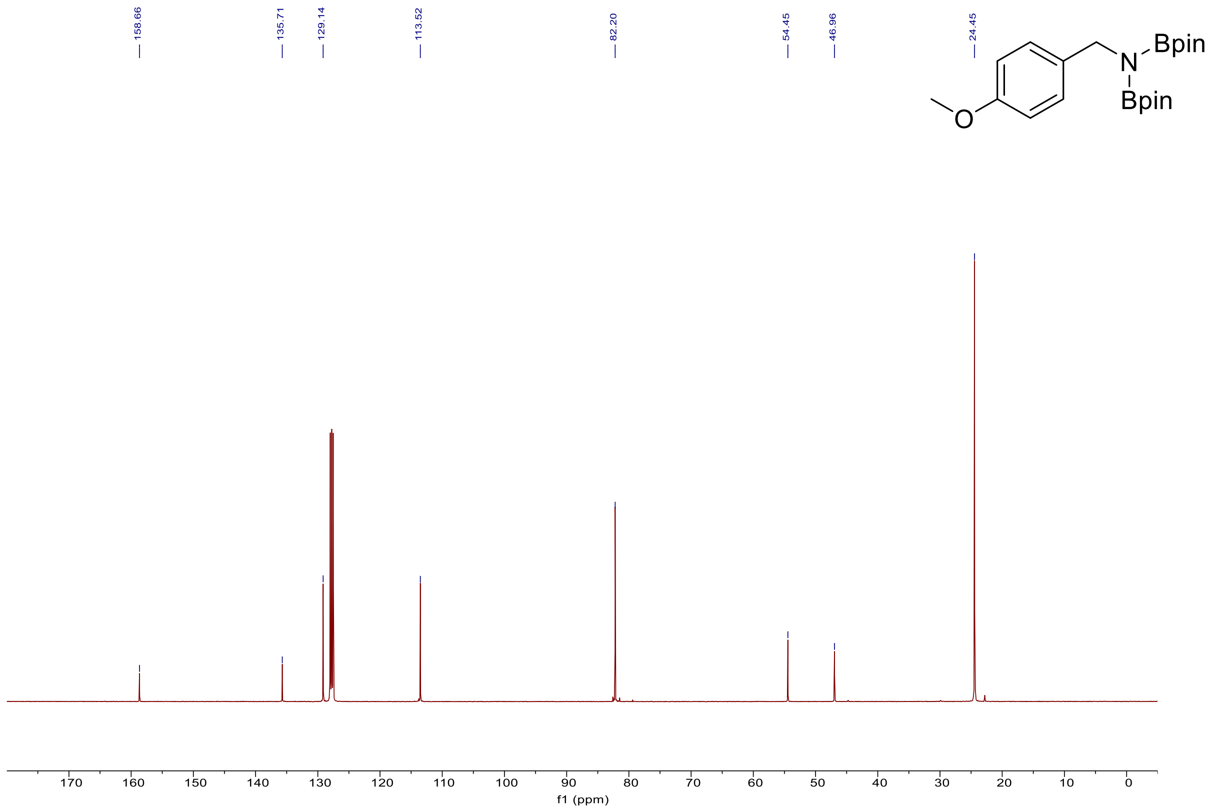

Figure S52 ^{13}C NMR of *N*-(4-methoxybenzyl)-4,4,5,5-tetramethyl-*N*-(4,4,5,5-tetramethyl-1,3,2-dioxaborolan-2-yl)-1,3,2- dioxaborolan-2-amine [S9] (4e)

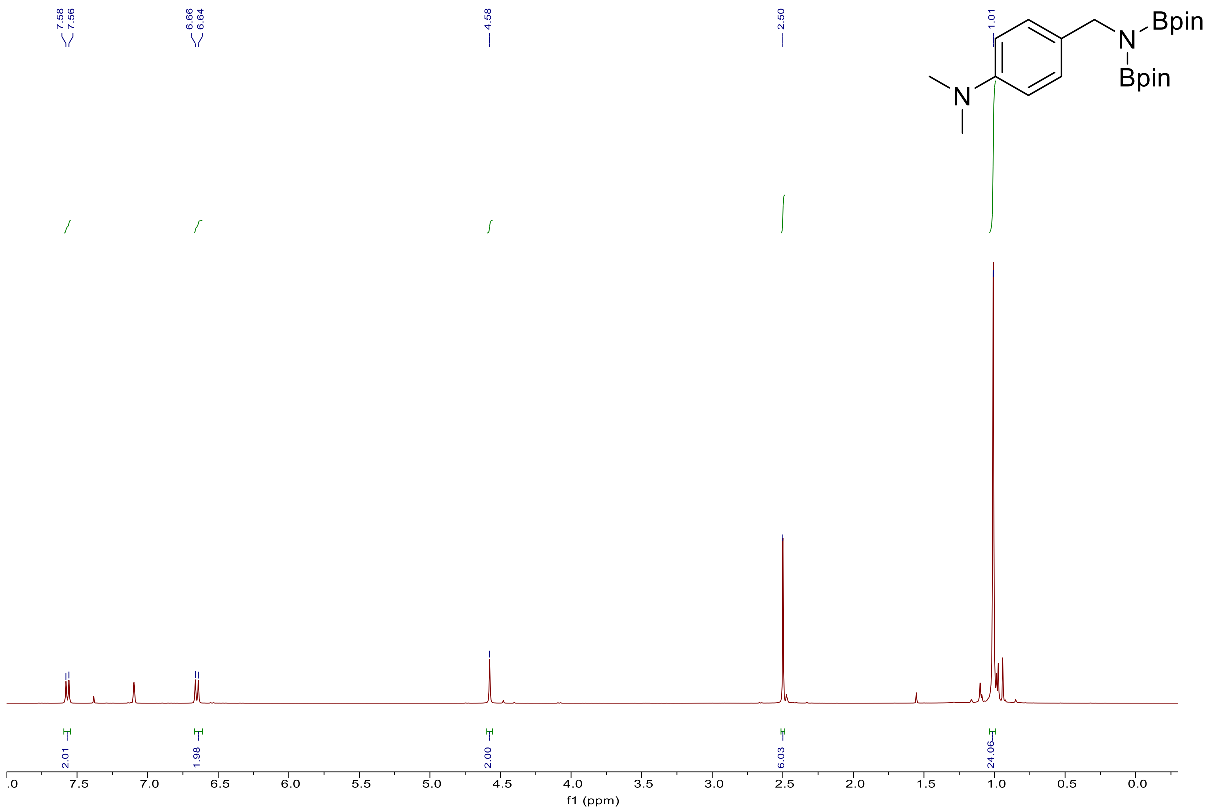

Figure S53 ^{1}H NMR of *N*-(4-(dimethylamino)benzyl)-4,4,5,5-tetramethyl-*N*-(4,4,5,5-tetramethyl-1,3,2-dioxaborolan-2-yl)- 1,3,2-dioxaborolan-2-amine) S9 (4f)

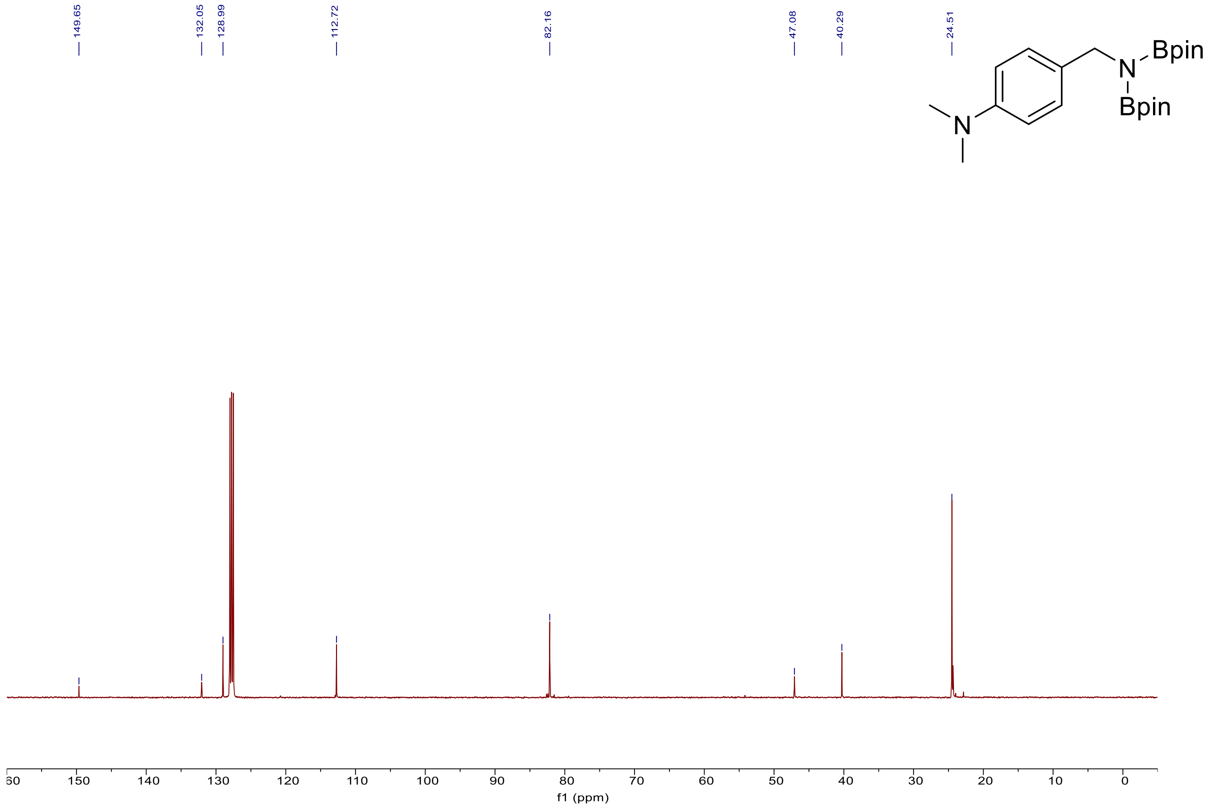

Figure S54 ^{13}C NMR of *N*-(4-(dimethylamino)benzyl)-4,4,5,5-tetramethyl-*N*-(4,4,5,5-tetramethyl-1,3,2-dioxaborolan-2-yl)- 1,3,2-dioxaborolan-2-amine) S9 (4f)

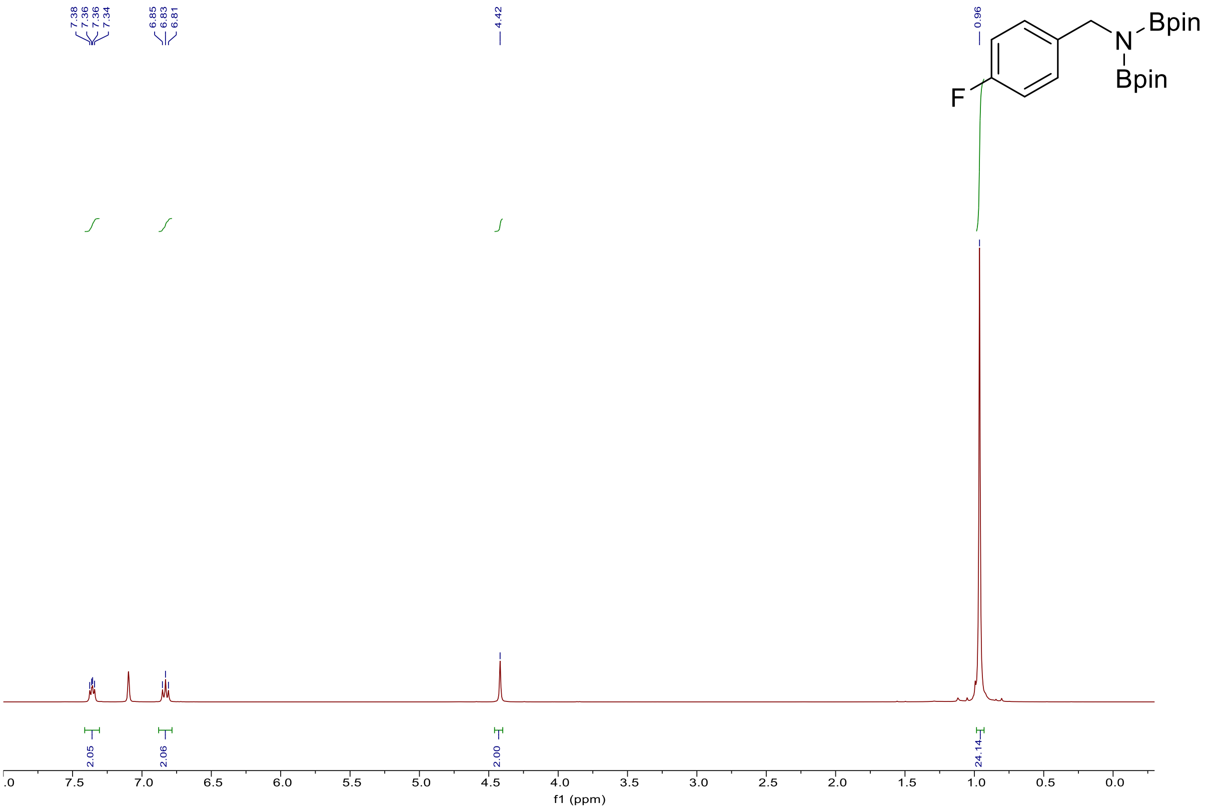

Figure S55 ^{1}H NMR of *N*-(4-fluorobenzyl)-4,4,5,5-tetramethyl-*N*-(4,4,5,5-tetramethyl-1,3,2-dioxaborolan-2-yl)-1,3,2- dioxaborolan-2-amine [S9] (4g)

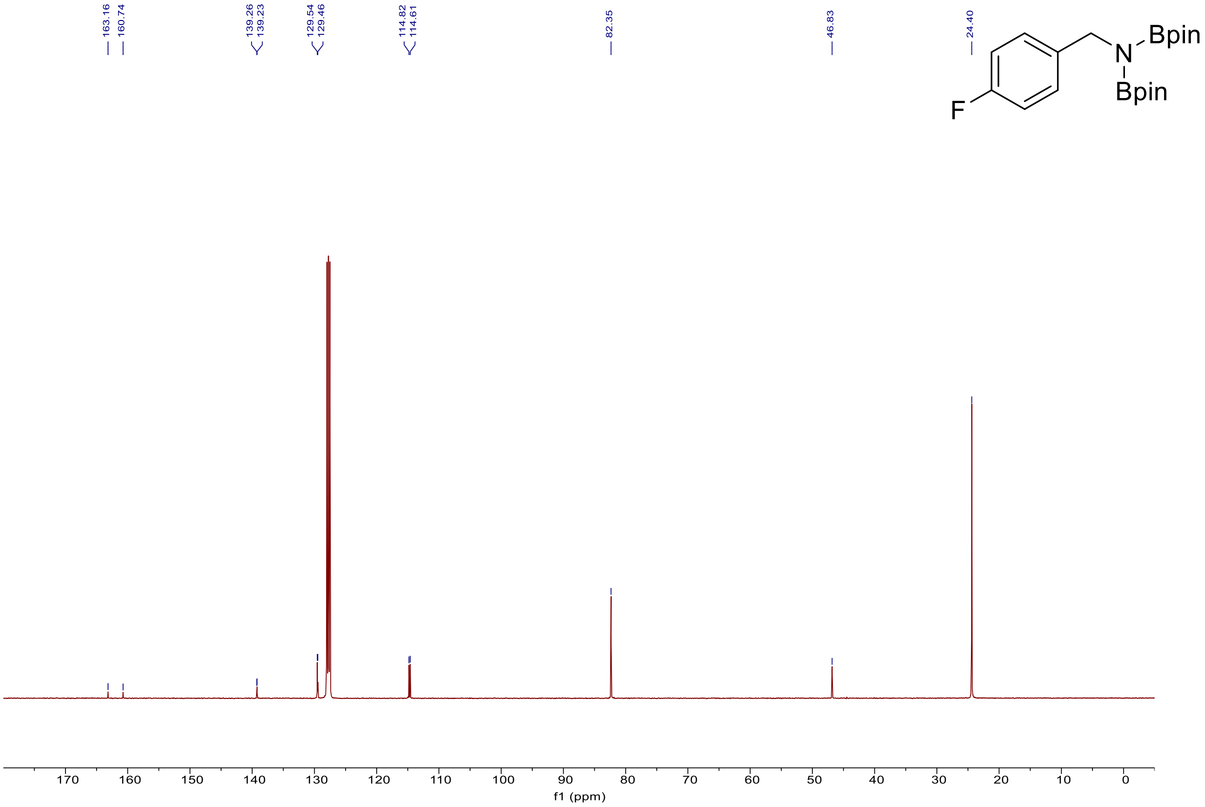

Figure S56 ^{13}C NMR of *N*-(4-fluorobenzyl)-4,4,5,5-tetramethyl-*N*-(4,4,5,5-tetramethyl-1,3,2-dioxaborolan-2-yl)-1,3,2- dioxaborolan-2-amine [S9] (4g)

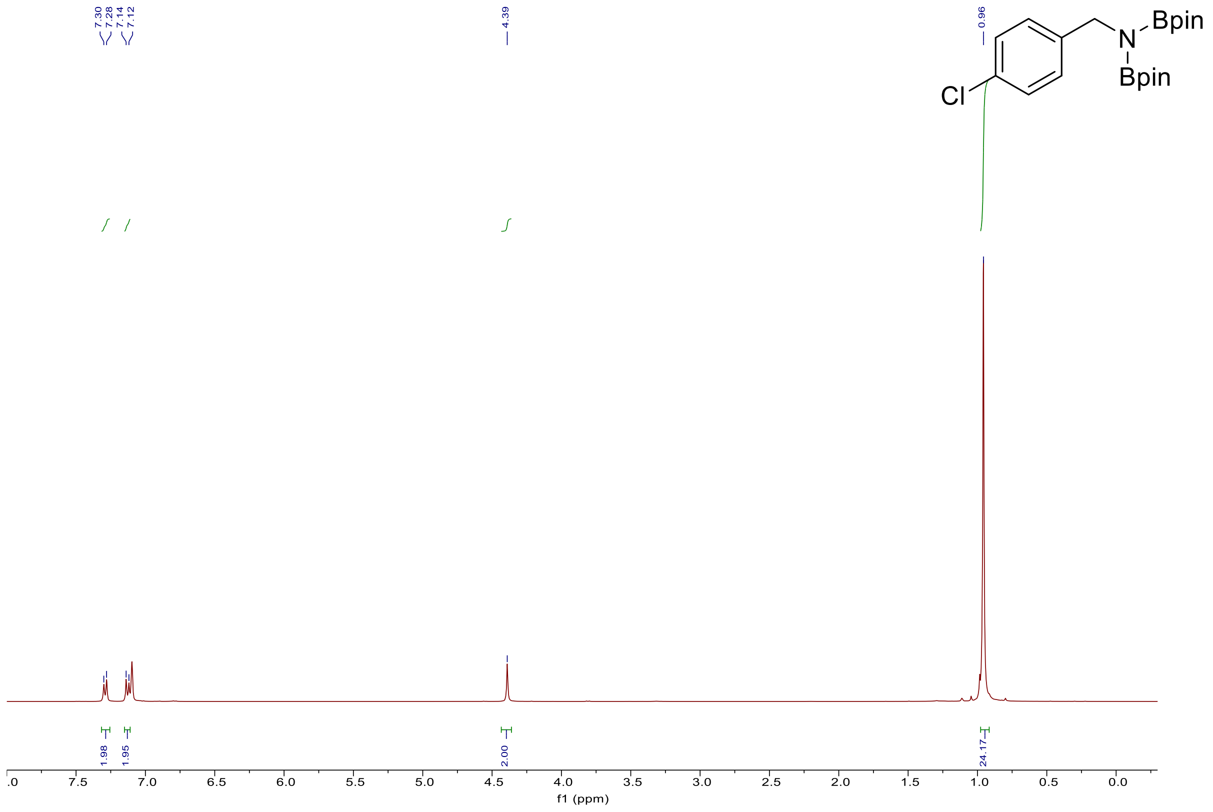

Figure S57 ^{1}H NMR of *N*-(4-chlorobenzyl)-4,4,5,5-tetramethyl-*N*-(4,4,5,5-tetramethyl-1,3,2-dioxaborolan-2-yl)-1,3,2- dioxaborolan-2-amine [S9] (4h)

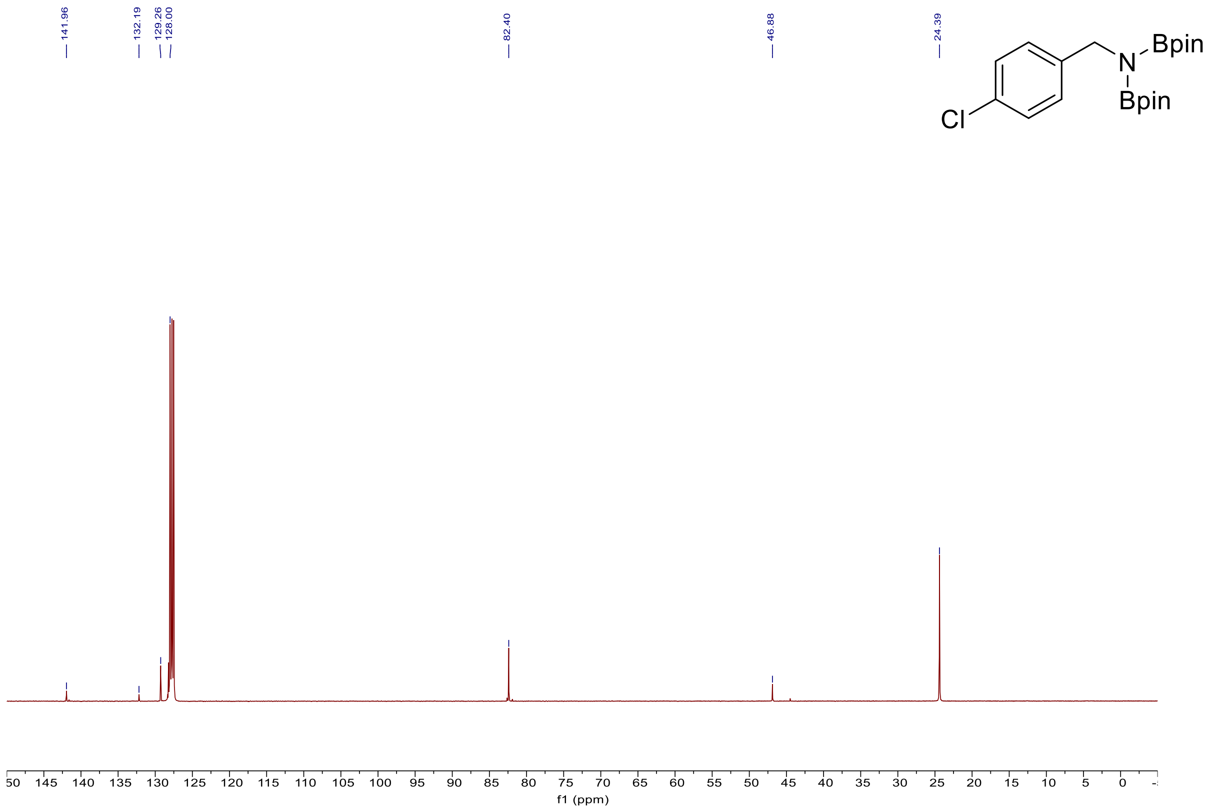

Figure S58 ^{13}C NMR of *N*-(4-chlorobenzyl)-4,4,5,5-tetramethyl-*N*-(4,4,5,5-tetramethyl-1,3,2-dioxaborolan-2-yl)-1,3,2- dioxaborolan-2-amine [S9] (4h)

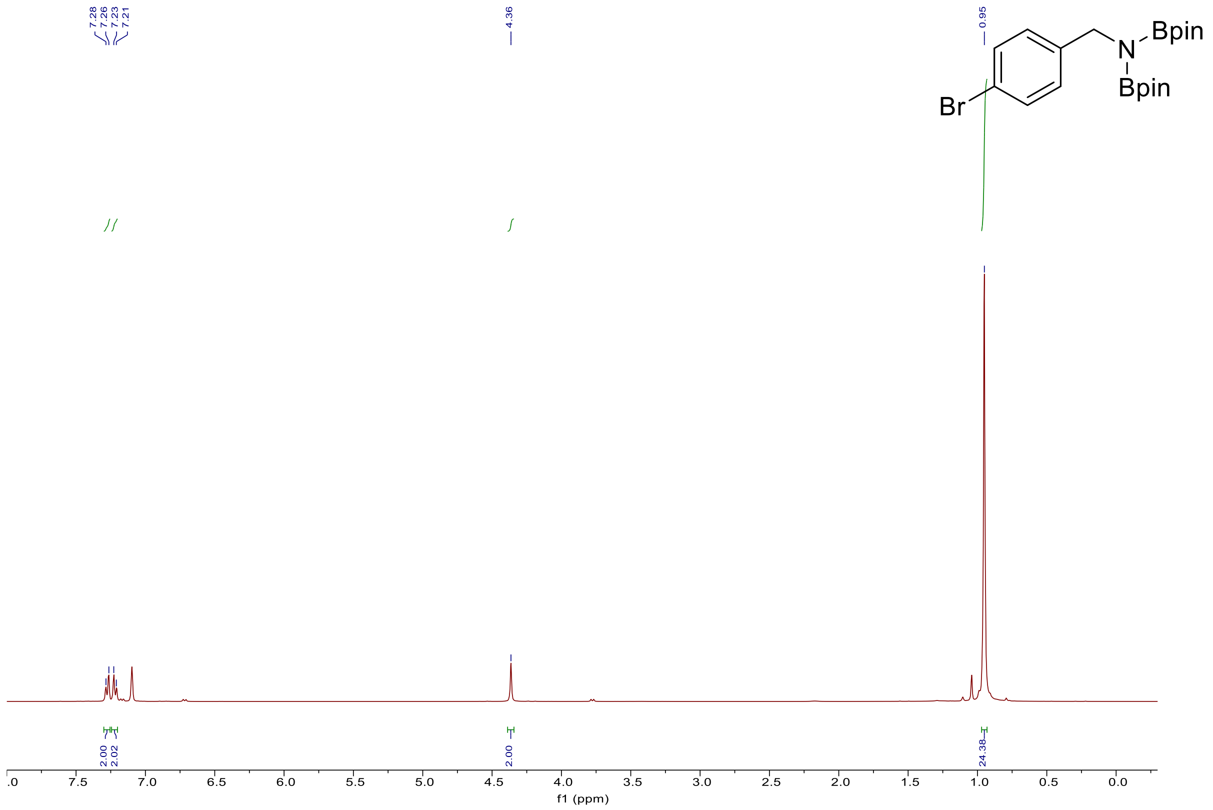

Figure S59 ^{1}H NMR of *N*-(4-bromobenzyl)-4,4,5,5-tetramethyl-*N*-(4,4,5,5-tetramethyl-1,3,2-dioxaborolan-2-yl)-1,3,2- S4 dioxaborolan-2-amine [S9] (4i)

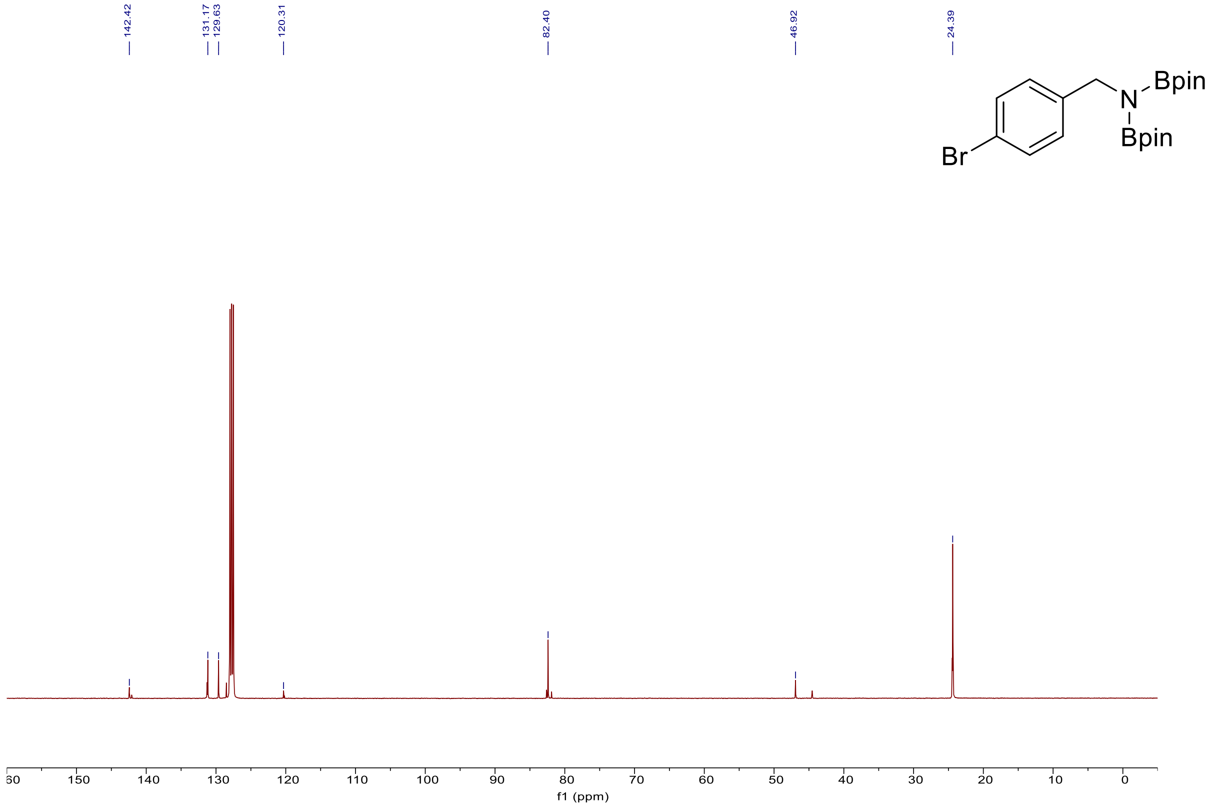

Figure S60 ^{13}C NMR of *N*-(4-bromobenzyl)-4,4,5,5-tetramethyl-*N*-(4,4,5,5-tetramethyl-1,3,2-dioxaborolan-2-yl)-1,3,2- S4 dioxaborolan-2-amine [S9] (4i)

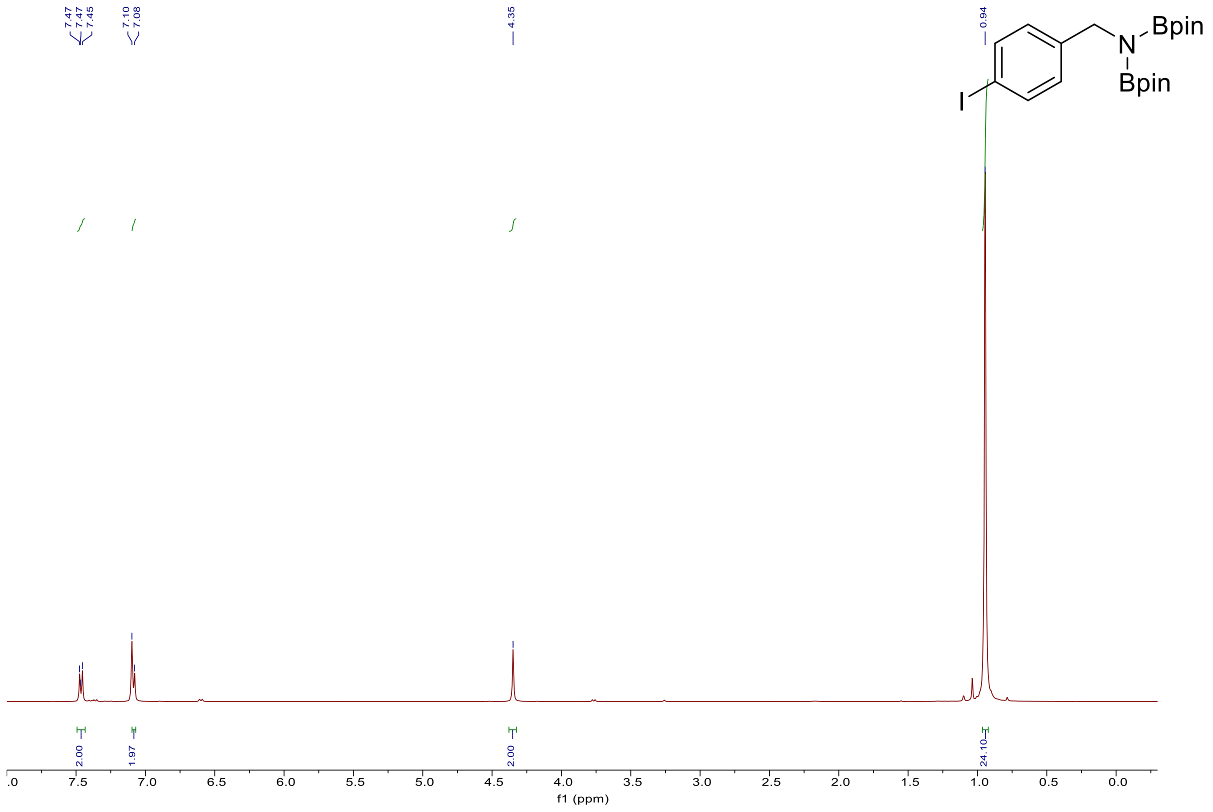

Figure S61 ^{1}H NMR of *N*-(4-iodobenzyl)-4,4,5,5-tetramethyl-*N*-(4,4,5,5-tetramethyl-1,3,2-dioxaborolan-2-yl)-1,3,2- dioxaborolan-2-amine [S9] (4j)

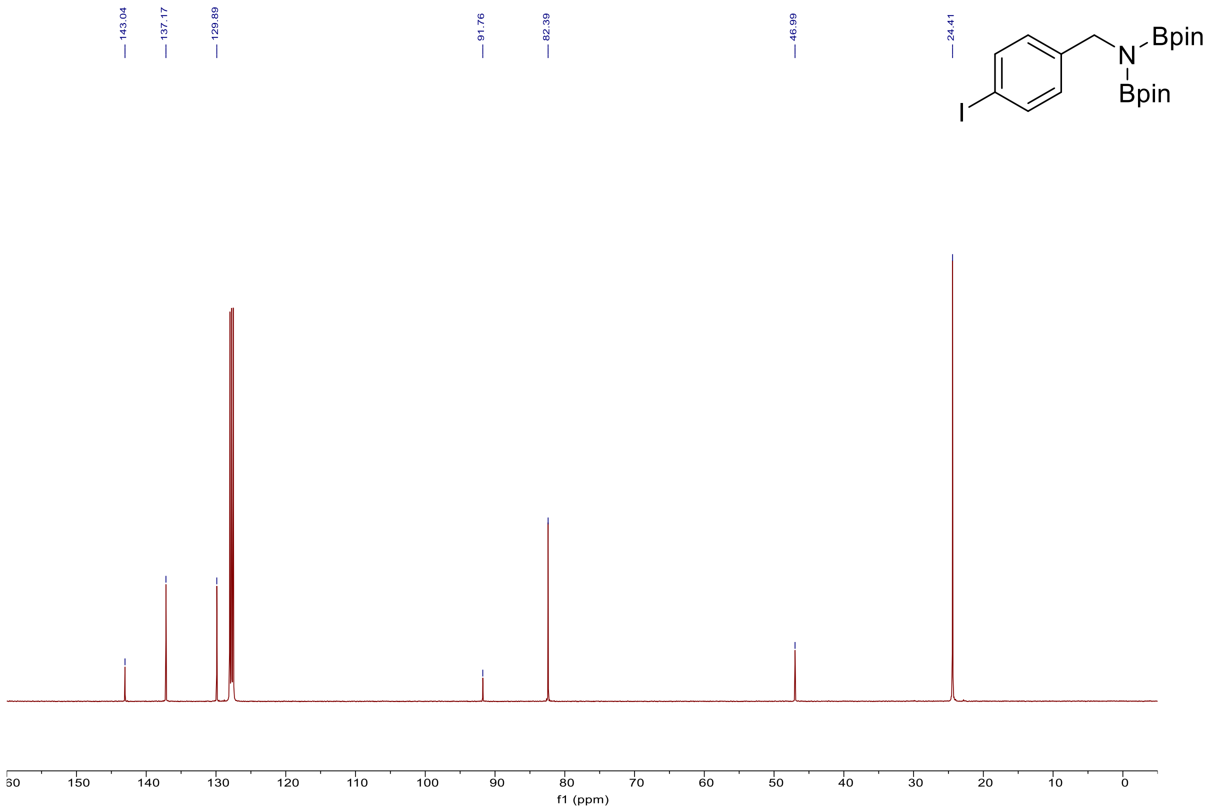

Figure S62 ^{13}C NMR of *N*-(4-iodobenzyl)-4,4,5,5-tetramethyl-*N*-(4,4,5,5-tetramethyl-1,3,2-dioxaborolan-2-yl)-1,3,2- dioxaborolan-2-amine [S9] (4j)

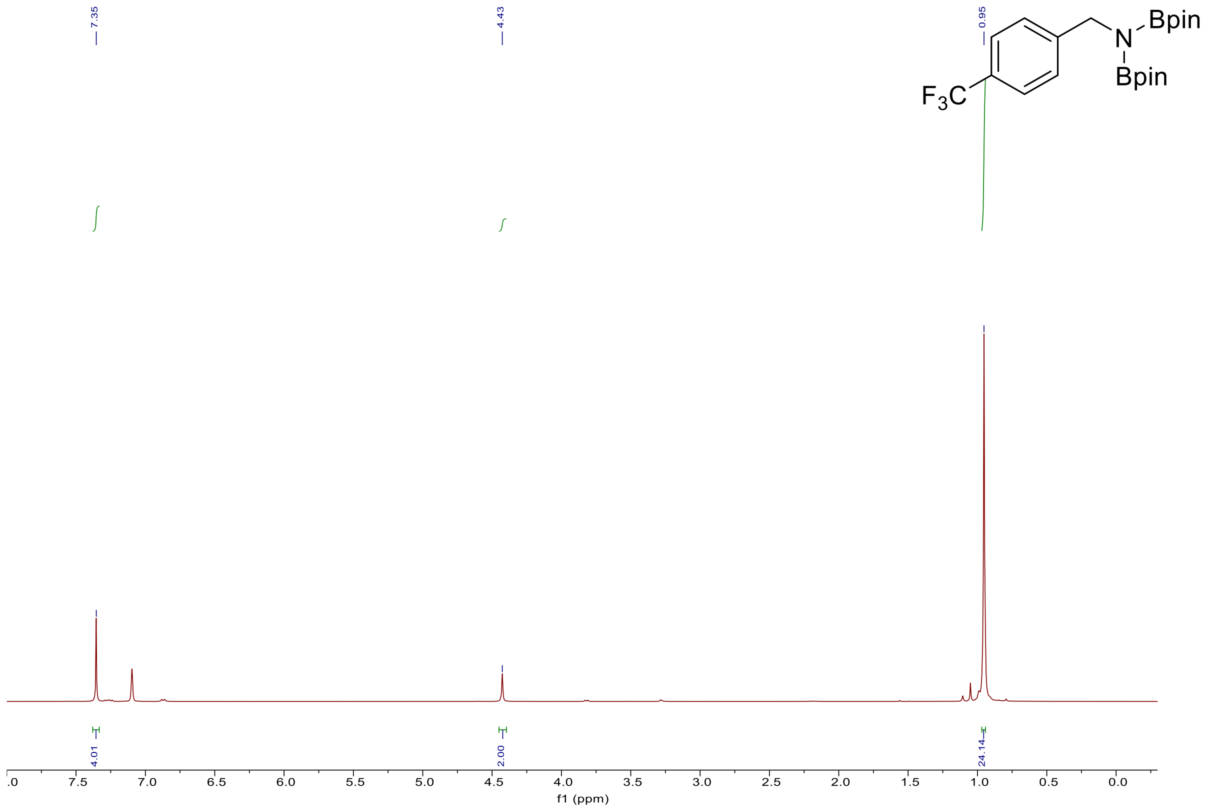

Figure S63 ^{1}H NMR of 4,4,5,5-Tetramethyl-*N*-(4,4,5,5-tetramethyl-1,3,2-dioxaborolan-2-yl)-*N*-(4-(trifluoromethyl)benzyl)- 1,3,2-dioxaborolan-2-amine [S9] (4k)

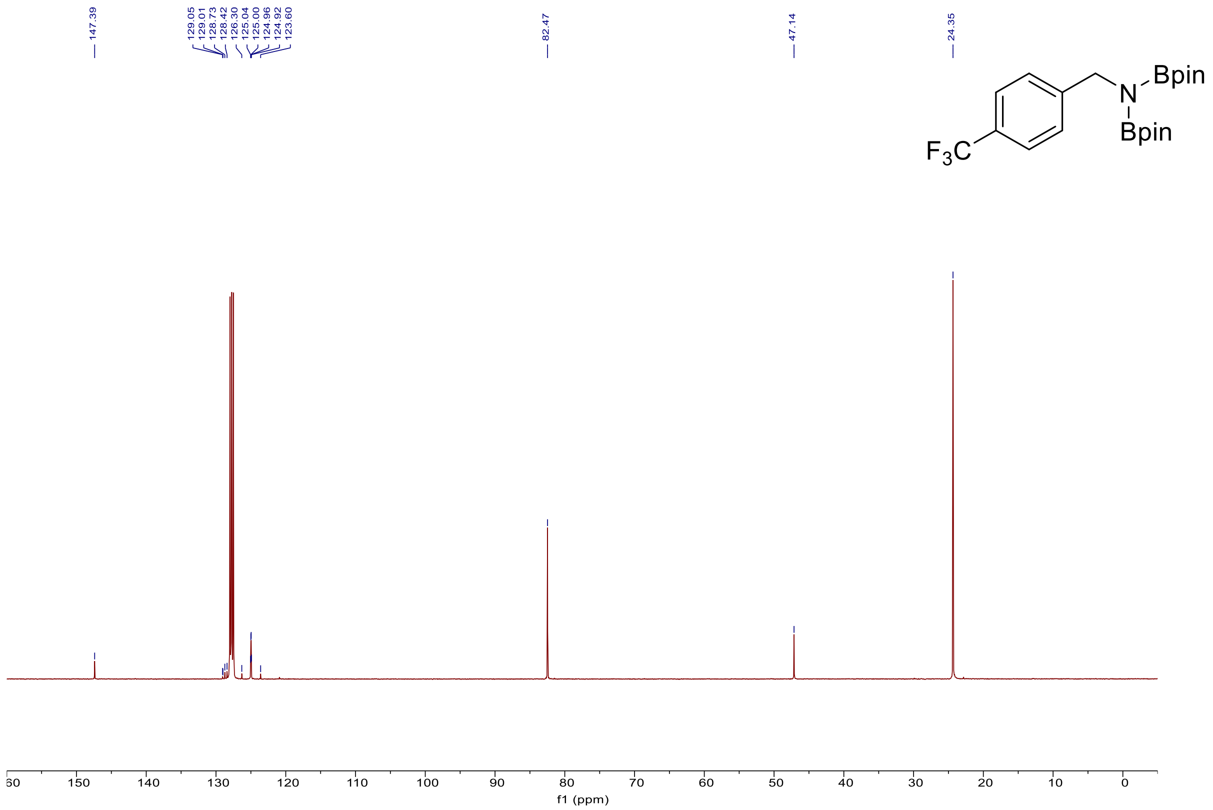

Figure S64 ^{13}C NMR of 4,4,5,5-Tetramethyl-*N*-(4,4,5,5-tetramethyl-1,3,2-dioxaborolan-2-yl)-*N*-(4-(trifluoromethyl)benzyl)- 1,3,2-dioxaborolan-2-amine [S9] (4k)

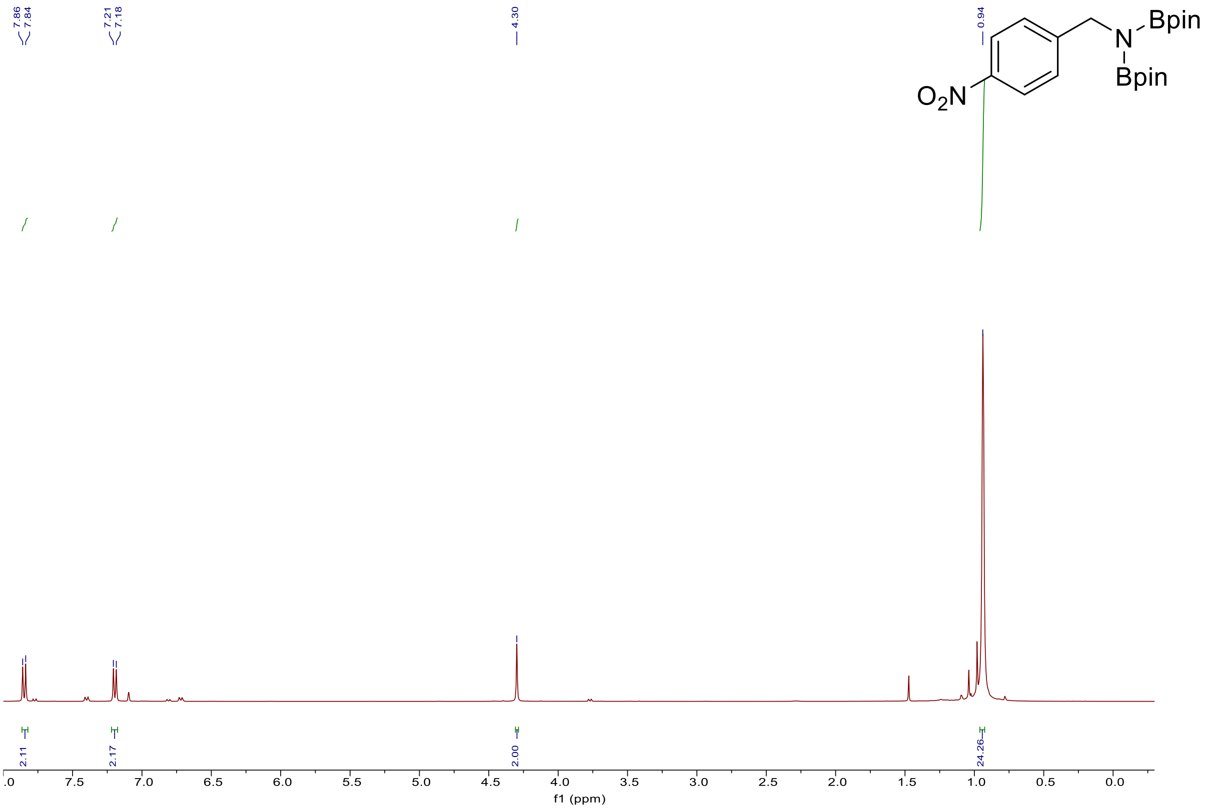

Figure S65 ^{1}H NMR of 4,4,5,5-Tetramethyl-*N*-(4-nitrobenzyl)-*N*-(4,4,5,5-tetramethyl-1,3,2-dioxaborolan-2-yl)-1,3,2- dioxaborolan-2-amine [S9] (4l)

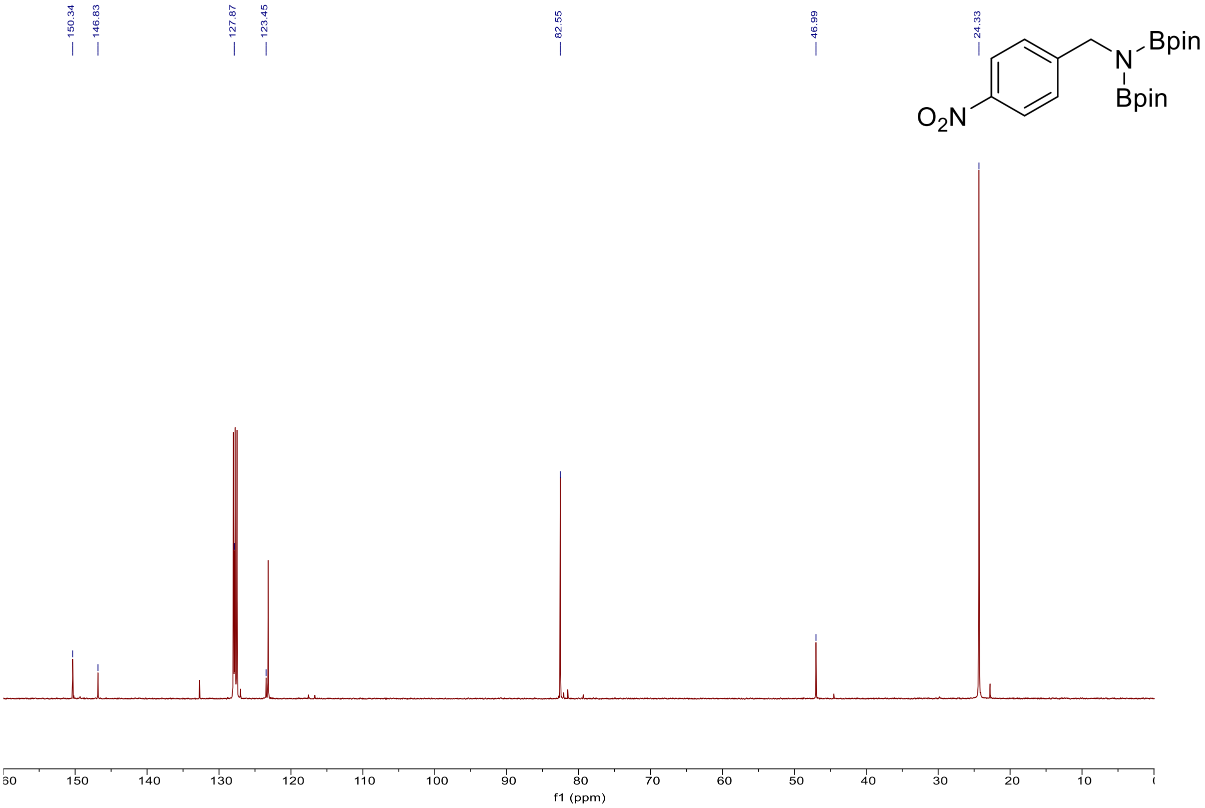

Figure S66 ^{13}C NMR of 4,4,5,5-Tetramethyl-*N*-(4-nitrobenzyl)-*N*-(4,4,5,5-tetramethyl-1,3,2-dioxaborolan-2-yl)-1,3,2- dioxaborolan-2-amine [S9] (4l)

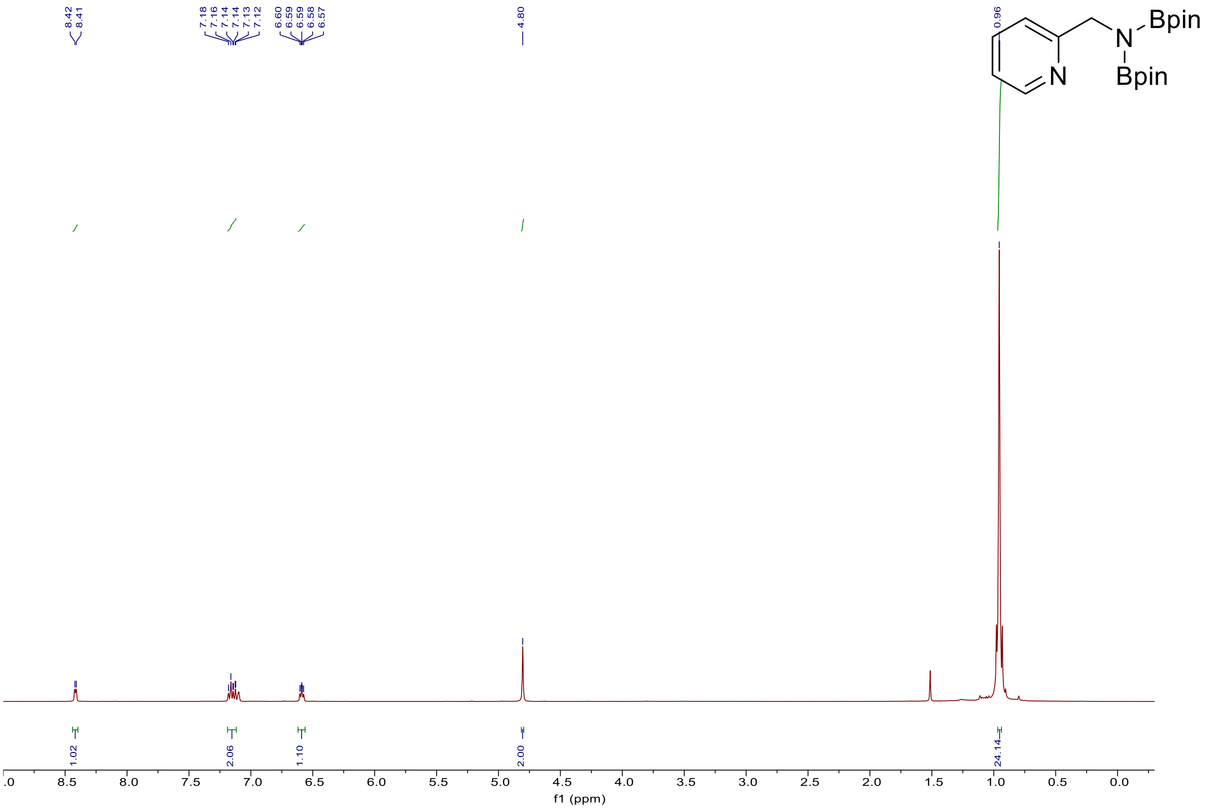

Figure S67 ^{1}H NMR of 4,4,5,5-Tetramethyl-*N*-(pyridin-2-ylmethyl)-*N*-(4,4,5,5-tetramethyl-1,3,2-dioxaborolan-2-yl)-1,3,2- dioxaborolan-2-amine [S9] (4m)

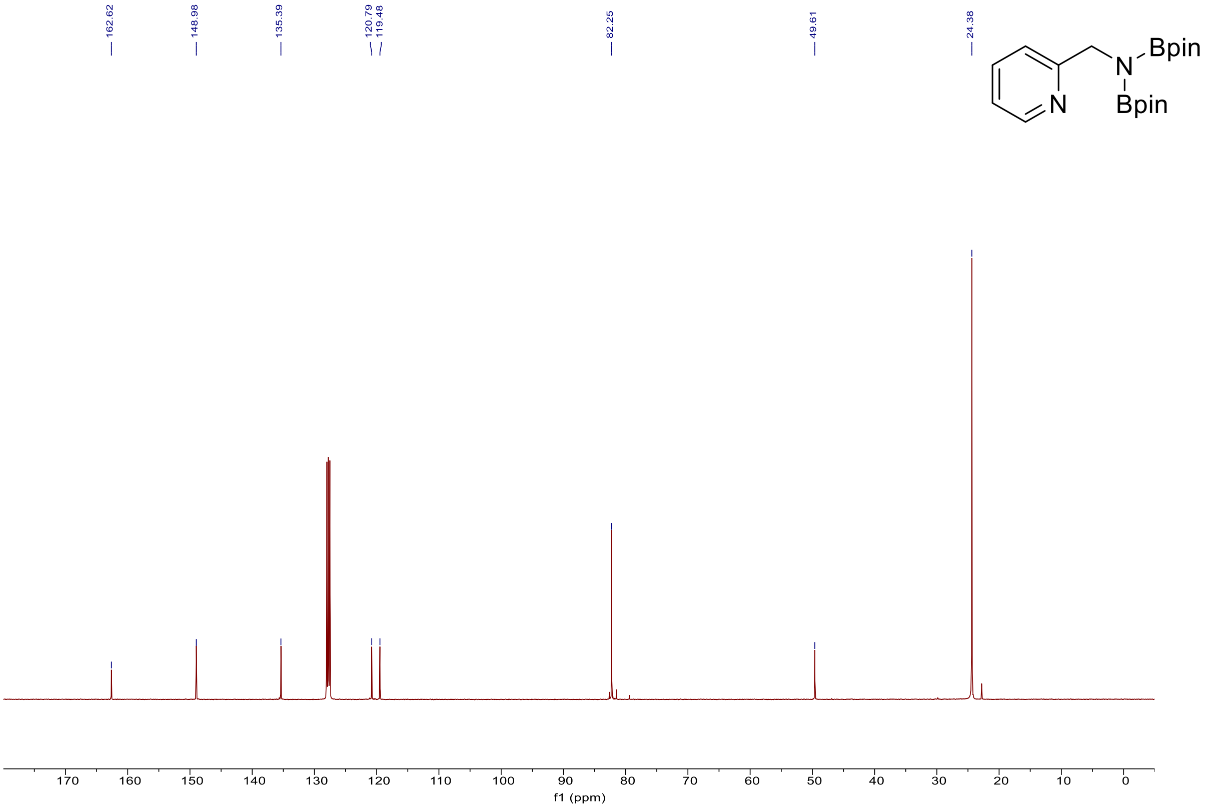

Figure S68 ^{13}C NMR of 4,4,5,5-Tetramethyl-*N*-(pyridin-2-ylmethyl)-*N*-(4,4,5,5-tetramethyl-1,3,2-dioxaborolan-2-yl)-1,3,2- dioxaborolan-2-amine [S9] (4m)

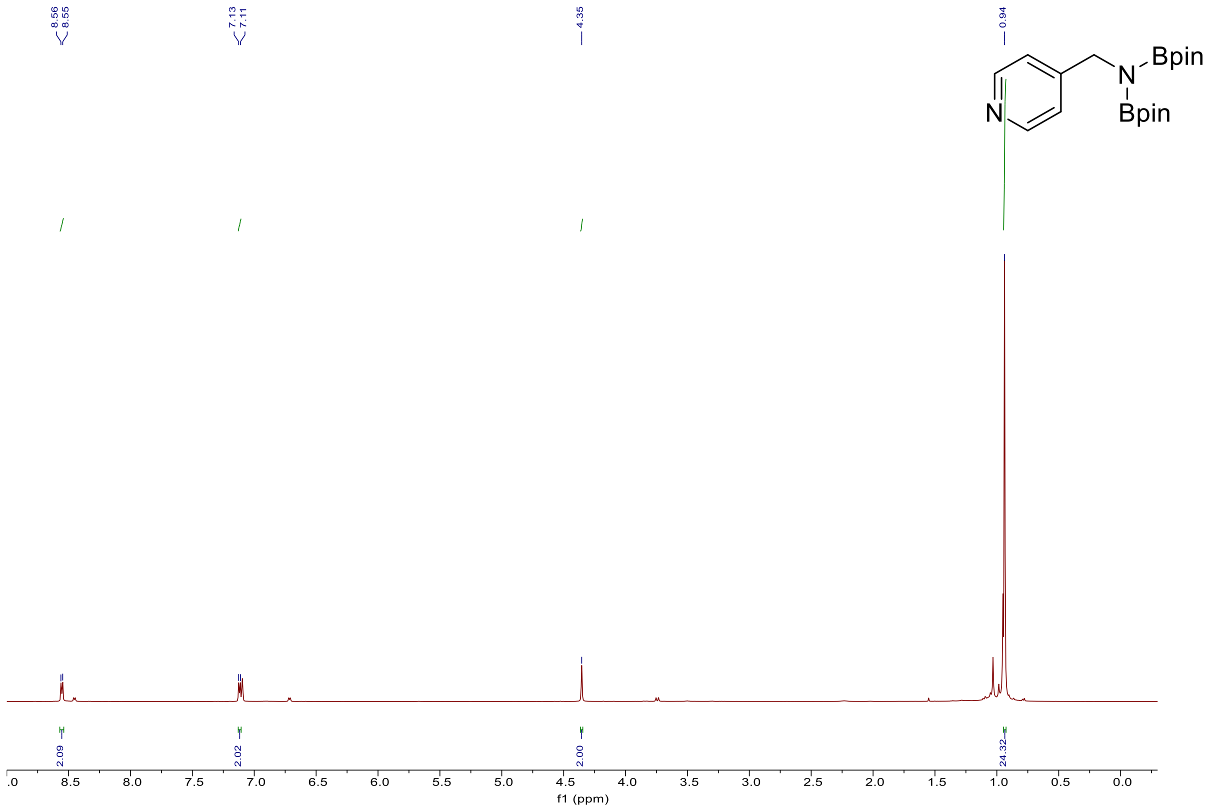

Figure S69 ^{1}H NMR of 4,4,5,5-Tetramethyl-*N*-(pyridin-4-ylmethyl)-*N*-(4,4,5,5-tetramethyl-1,3,2-dioxaborolan-2-yl)-1,3,2- dioxaborolan-2-amine [S9] (4n)

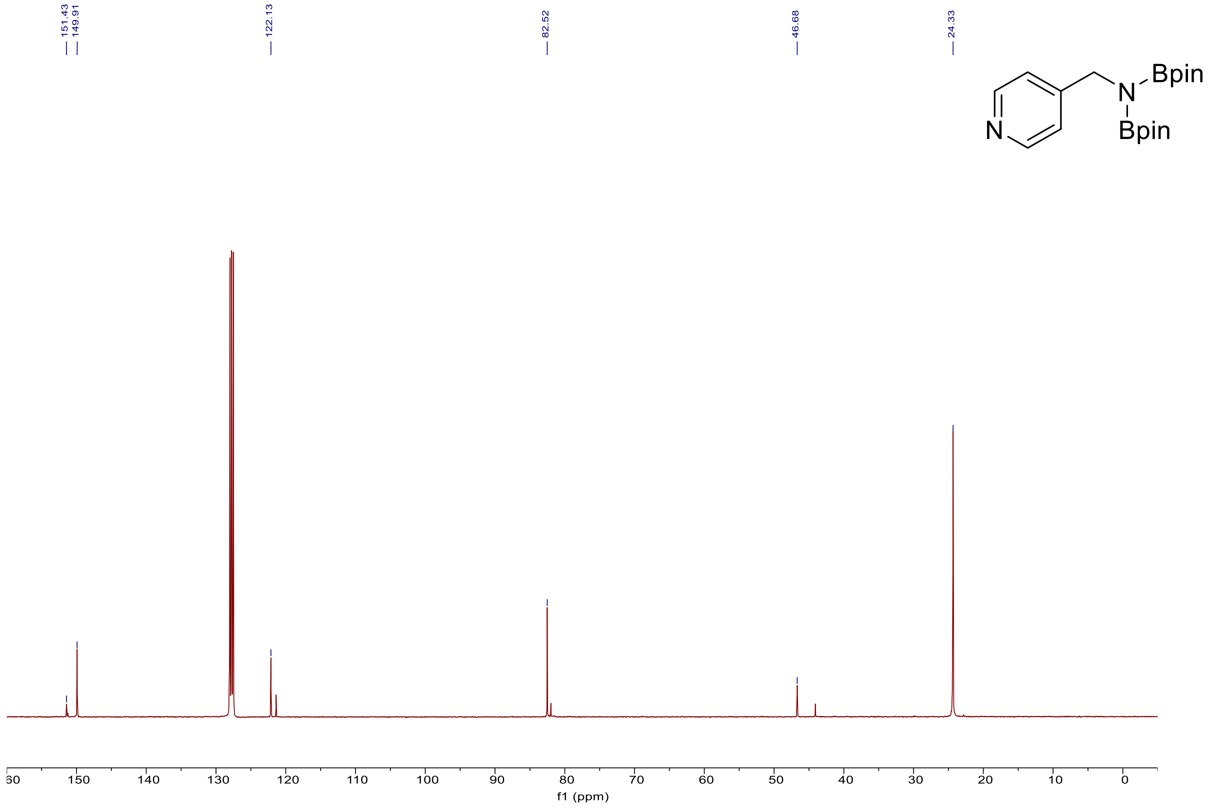

Figure S70 ^{13}C NMR of 4,4,5,5-Tetramethyl-*N*-(pyridin-4-ylmethyl)-*N*-(4,4,5,5-tetramethyl-1,3,2-dioxaborolan-2-yl)-1,3,2- dioxaborolan-2-amine [S9] (4n)

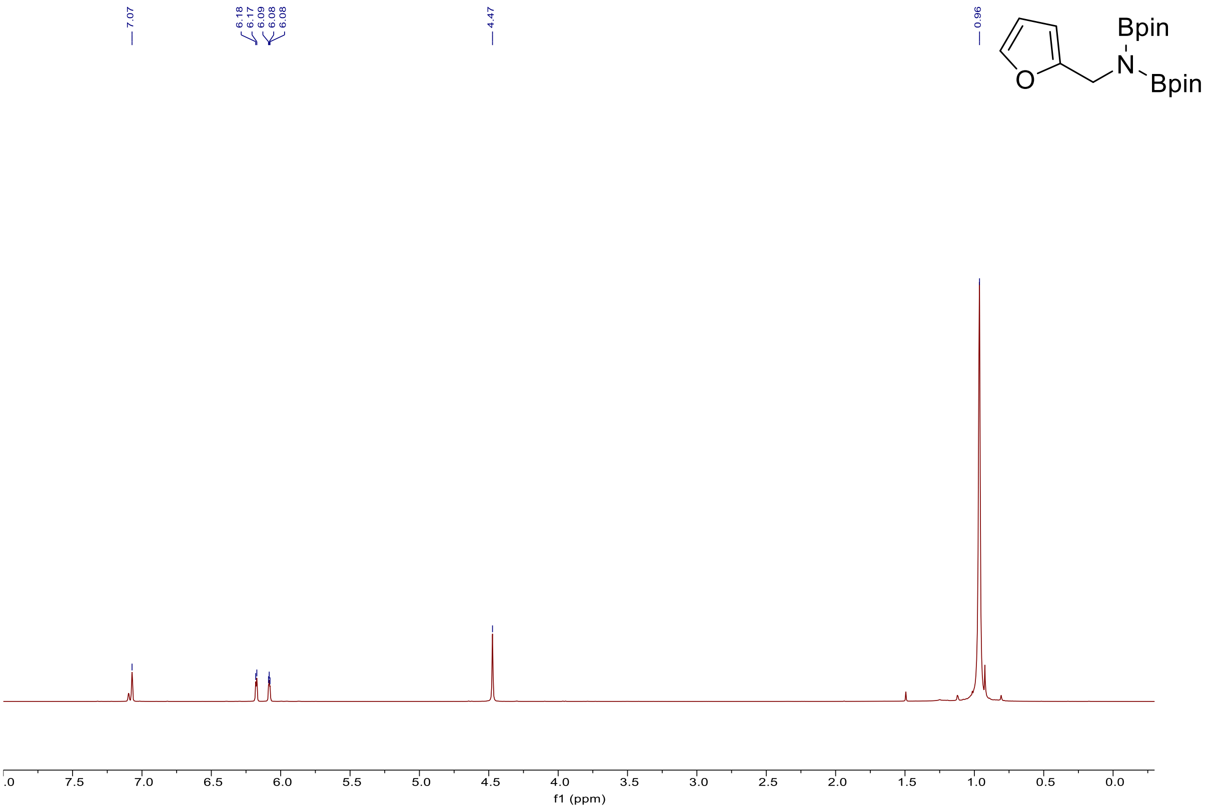

Figure S71 ^{1}H NMR of *N*-(Furan-2-ylmethyl)-4,4,5,5-tetramethyl-*N*-(4,4,5,5-tetramethyl-1,3,2-dioxaborolan-2-yl)-1,3,2- dioxaborolan-2-amine [S9] (4o)

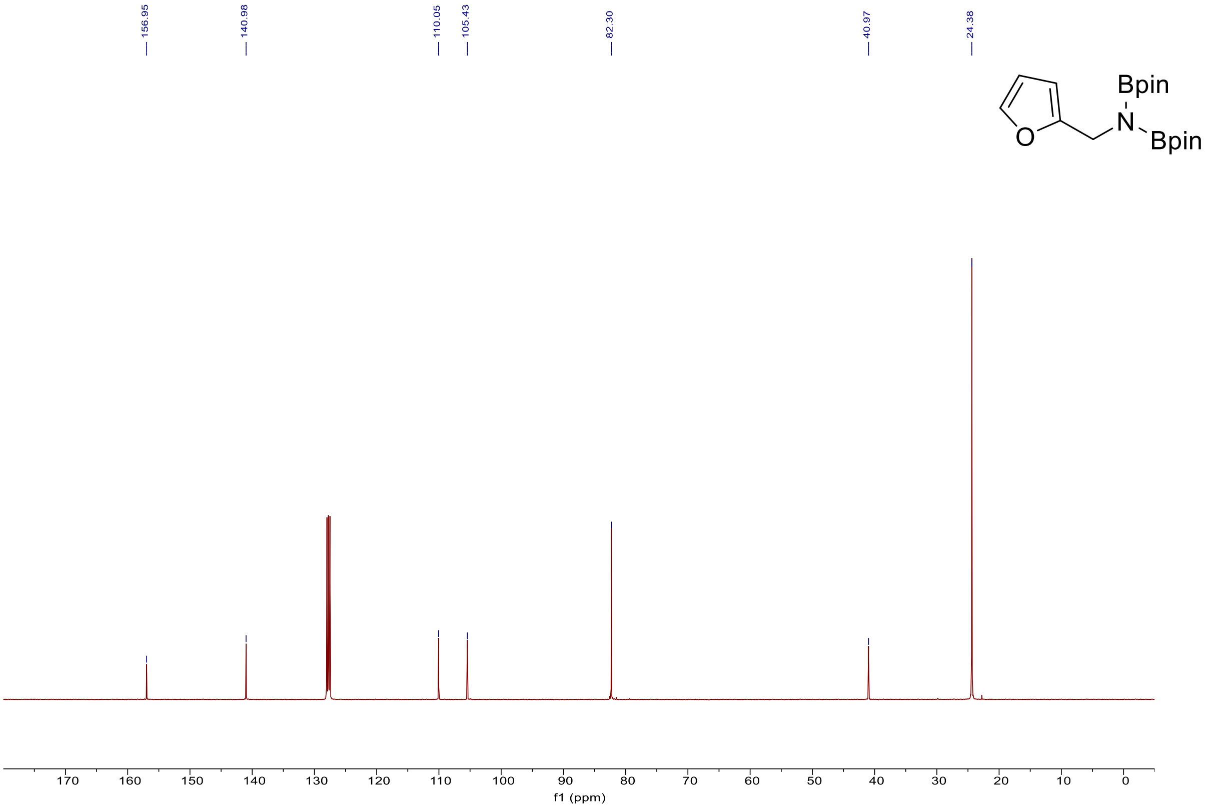

Figure S72 ^{13}C NMR of *N*-(Furan-2-ylmethyl)-4,4,5,5-tetramethyl-*N*-(4,4,5,5-tetramethyl-1,3,2-dioxaborolan-2-yl)-1,3,2- dioxaborolan-2-amine [S9] (4o)

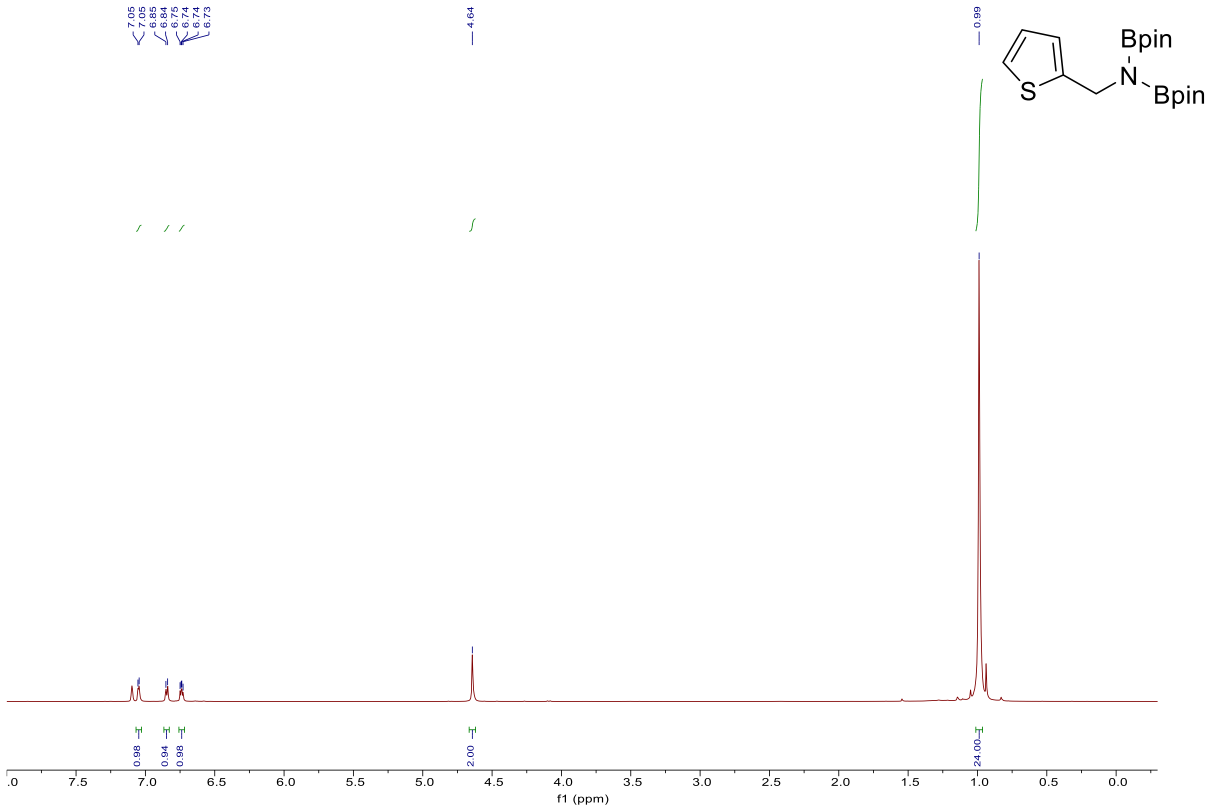

Figure S73 ^{1}H NMR of 4,4,5,5-tetramethyl-*N*-(4,4,5,5-tetramethyl-1,3,2-dioxaborolan-2-yl)-*N*-(thiophen-2-ylmethyl)-1,3,2-dioxaborolan-2-amine [S10] (4p)

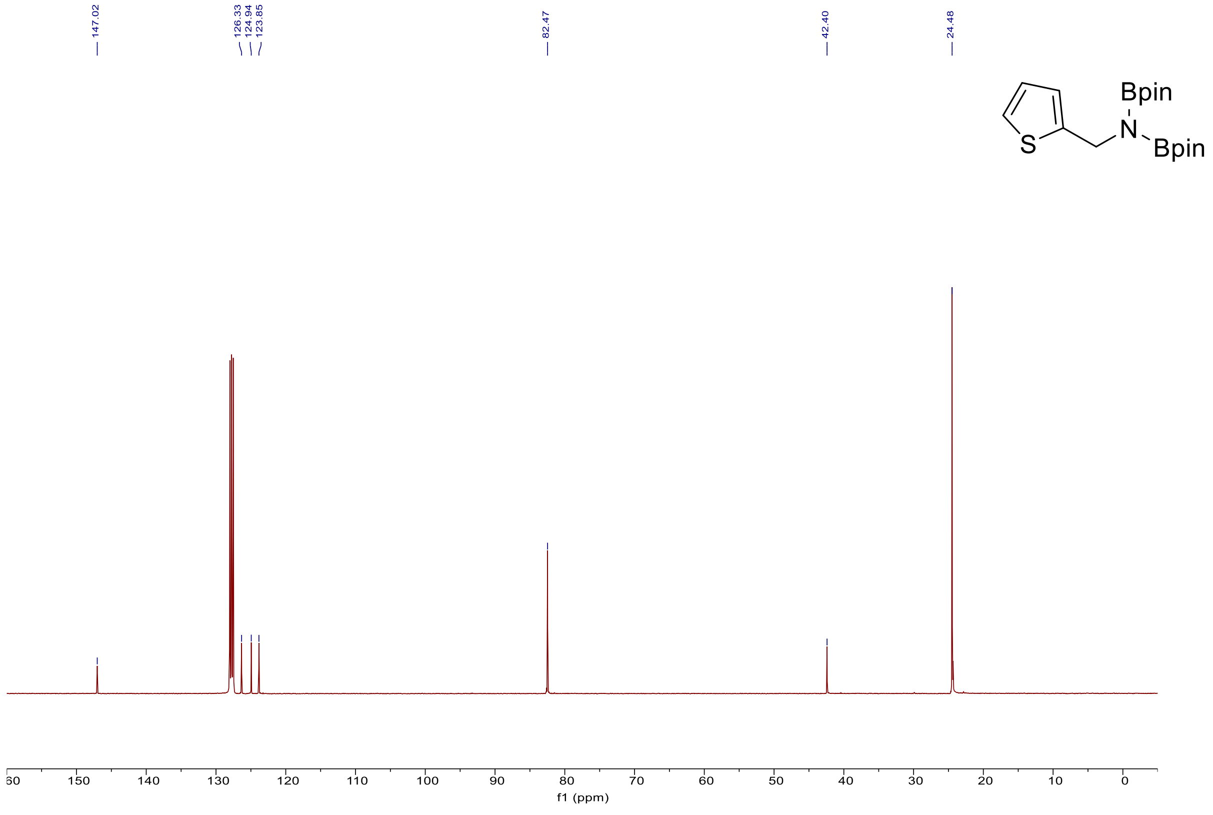

Figure S74 ^{13}C NMR of 4,4,5,5-tetramethyl-*N*-(4,4,5,5-tetramethyl-1,3,2-dioxaborolan-2-yl)-*N*-(thiophen-2-ylmethyl)-1,3,2-dioxaborolan-2-amine [S10] (4p)

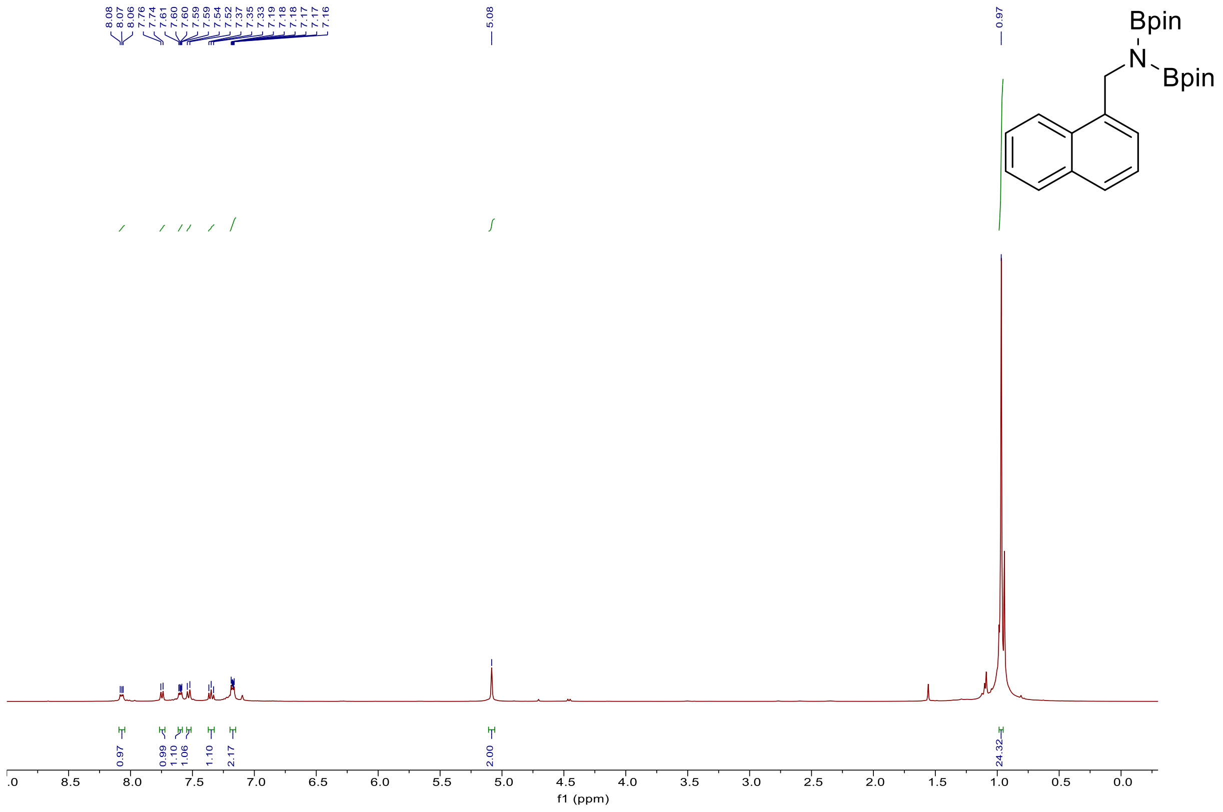

Figure S75 ^{1}H NMR of 4,4,5,5-Tetramethyl-*N*-(naphthalen-1-ylmethyl)-*N*-(4,4,5,5-tetramethyl-1,3,2-dioxaborolan-2-yl)- 1,3,2-dioxaborolan-2-amine [S9] (4q)

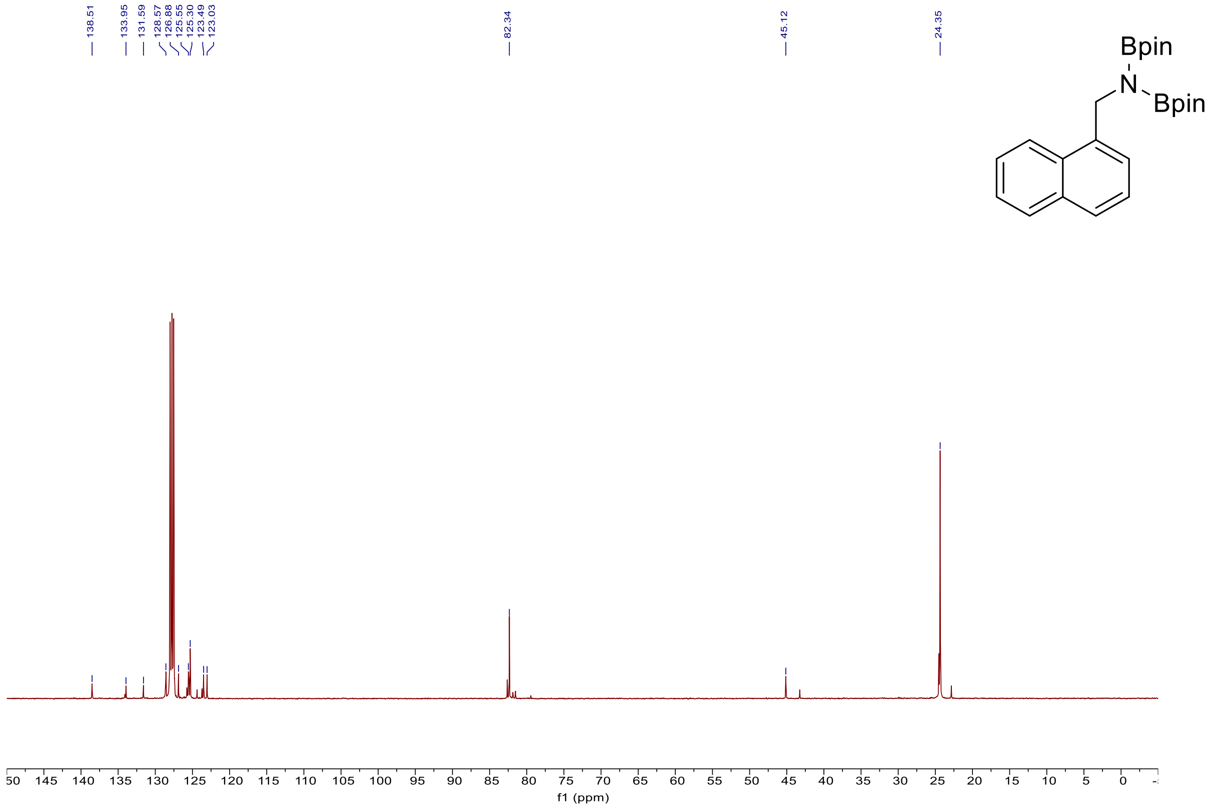

Figure S76 ^{13}C NMR of 4,4,5,5-Tetramethyl-*N*-(naphthalen-1-ylmethyl)-*N*-(4,4,5,5-tetramethyl-1,3,2-dioxaborolan-2-yl)- 1,3,2-dioxaborolan-2-amine [S9] (4q)

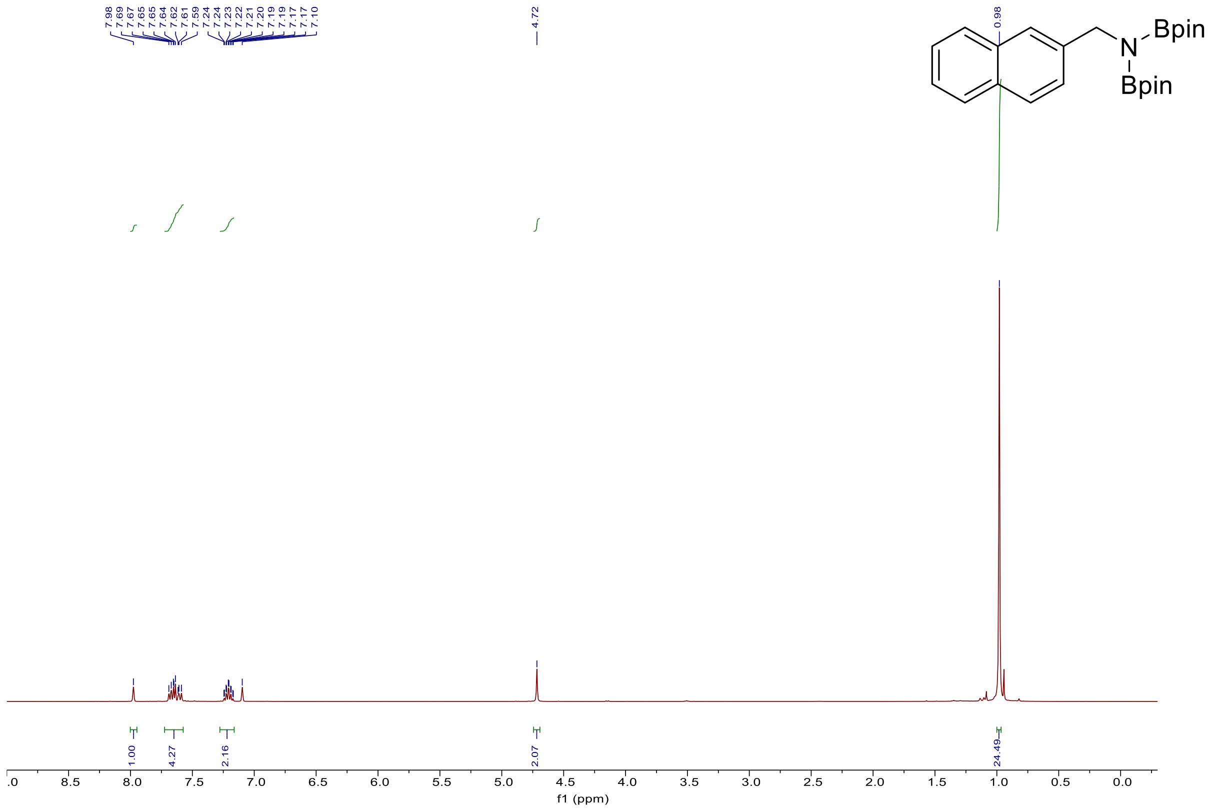

Figure S77 ^{1}H NMR of 4,4,5,5-tetramethyl-*N*-(naphthalen-2-ylmethyl)-*N*-(4,4,5,5-tetramethyl-1,3,2-dioxaborolan-2-yl)-1,3,2-dioxaborolan-2-amine [S11] (4r)

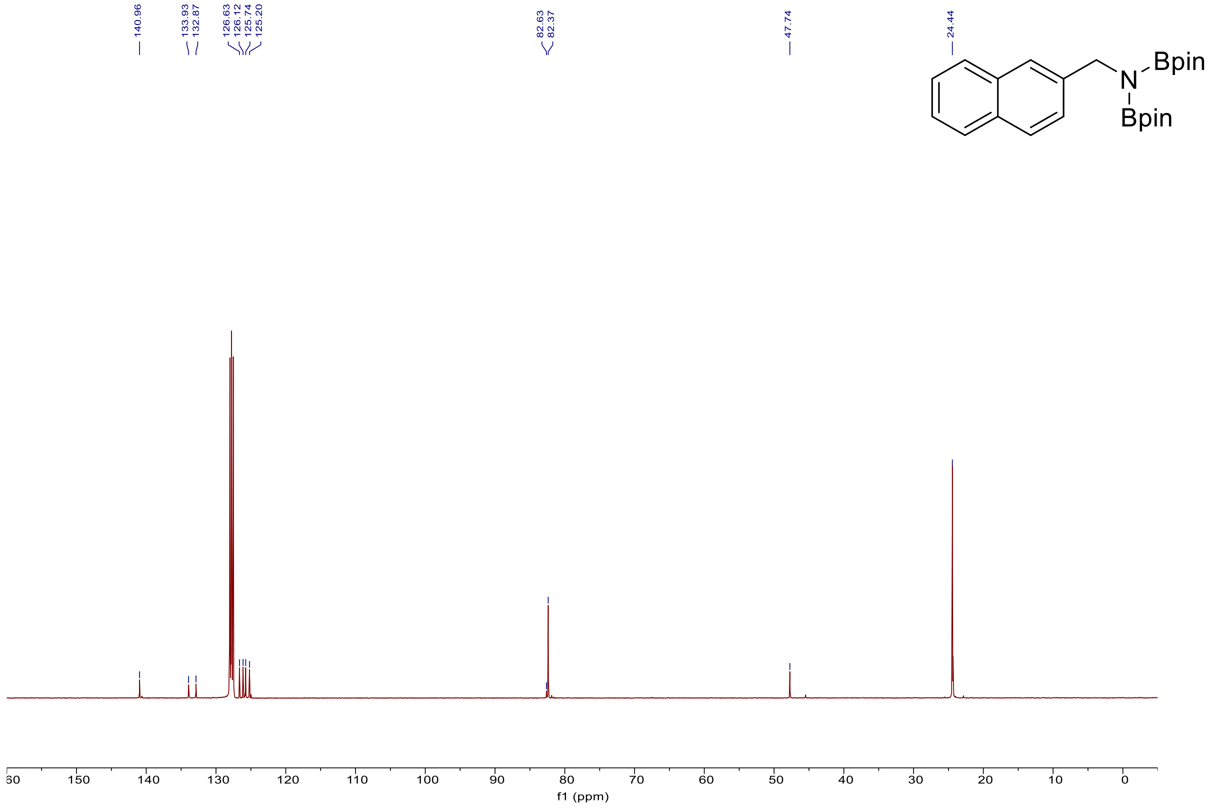

Figure S78 ^{1}H NMR of 4,4,5,5-tetramethyl-*N*-(naphthalen-2-ylmethyl)-*N*-(4,4,5,5-tetramethyl-1,3,2-dioxaborolan-2-yl)-1,3,2-dioxaborolan-2-amine [S12] (4r)

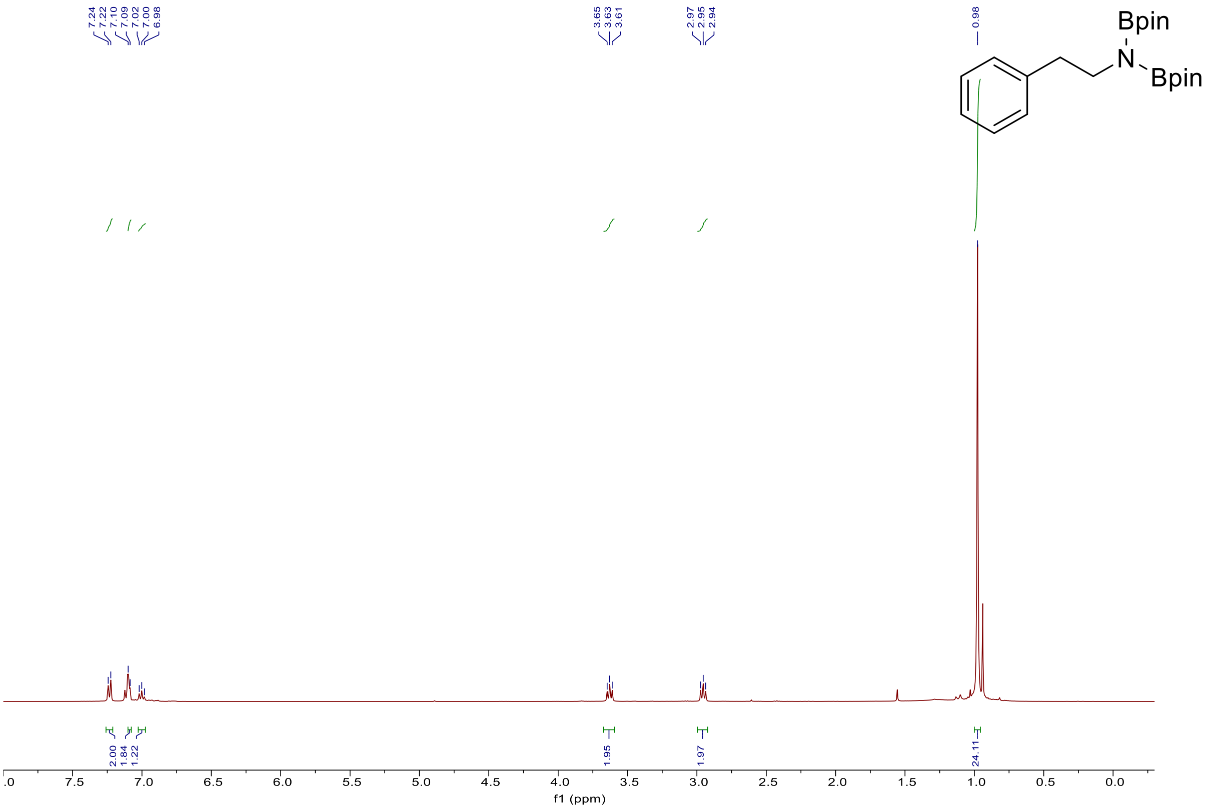

Figure S79 ^{1}H NMR of 4,4,5,5-Tetramethyl-*N*-phenethyl-*N*-(4,4,5,5-tetramethyl-1,3,2-dioxaborolan-2-yl)-1,3,2- dioxaborolan-2-amine [S9] (4s)

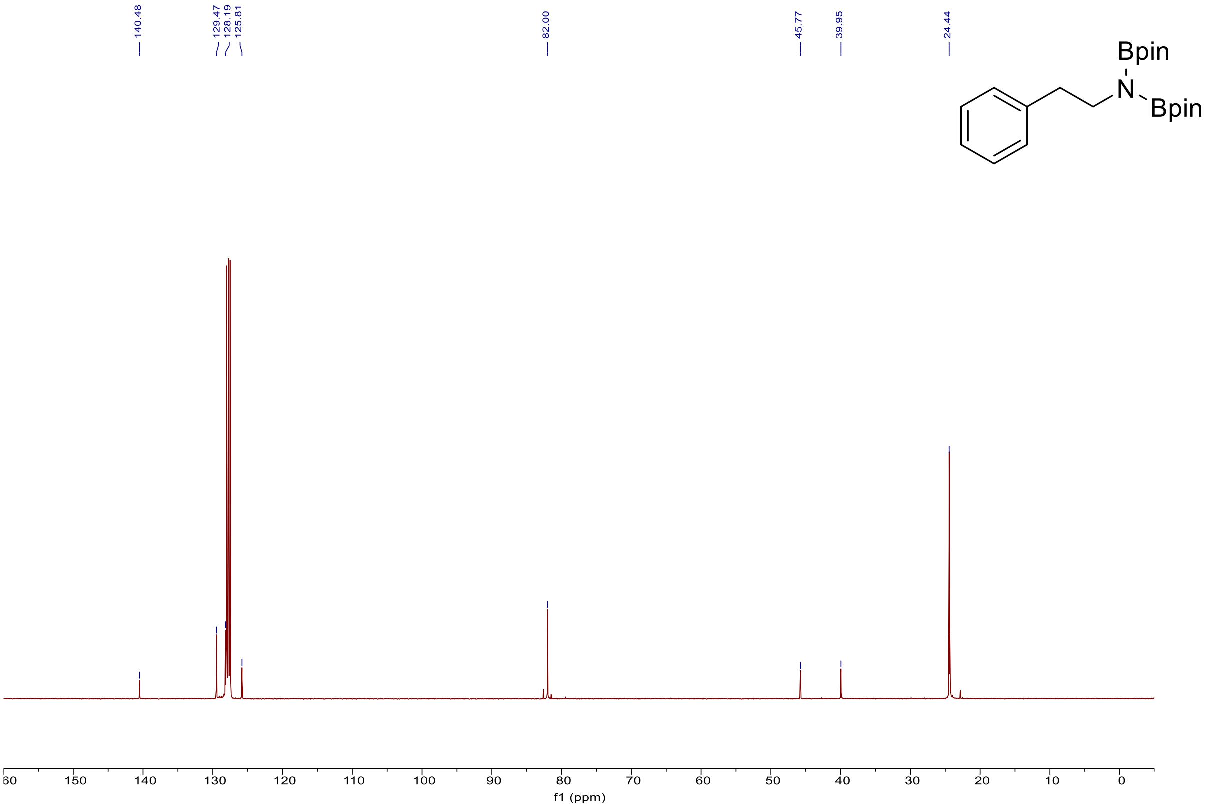

Figure S80 ^{13}C NMR of 4,4,5,5-Tetramethyl-*N*-phenethyl-*N*-(4,4,5,5-tetramethyl-1,3,2-dioxaborolan-2-yl)-1,3,2- dioxaborolan-2-amine [S9] (4s)

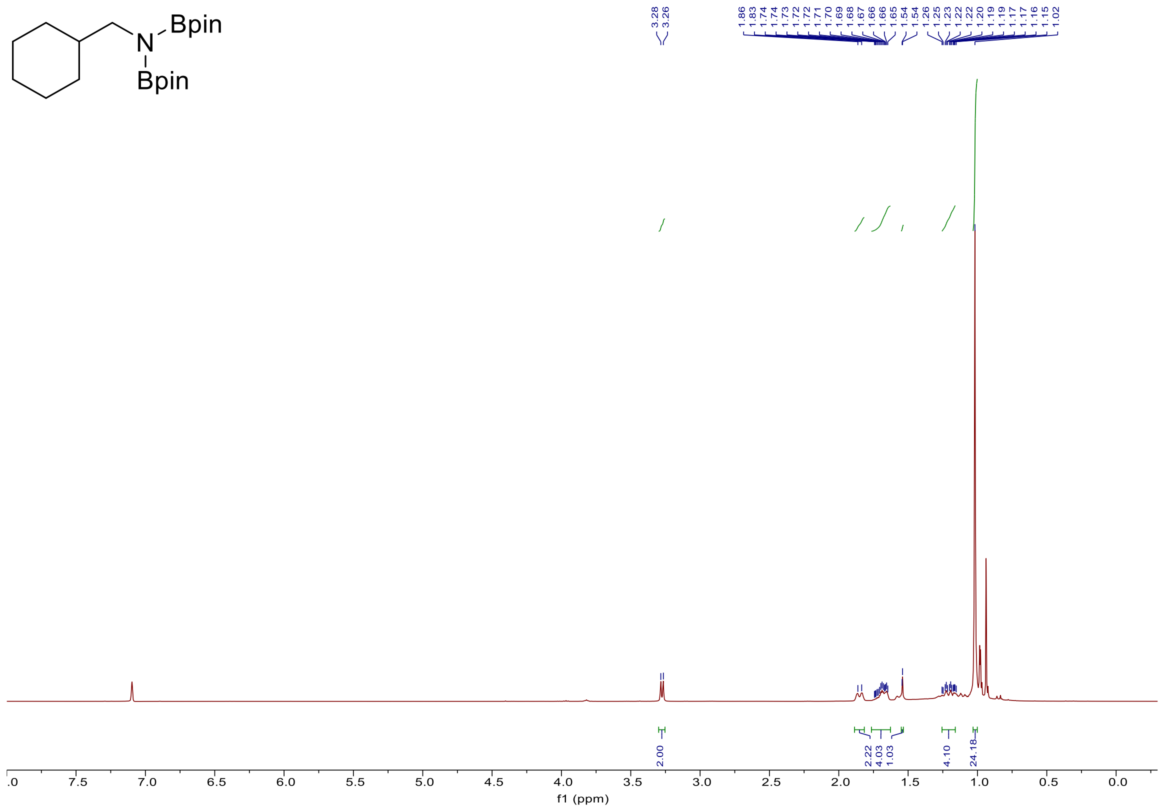

Figure S81 ^{1}H NMR of *N*-(cyclohexylmethyl)-4,4,5,5-tetramethyl-*N*-(4,4,5,5-tetramethyl-1,3,2-dioxaborolan-2-yl)-1,3,2-dioxaborolan-2-amine [S13] (4t)

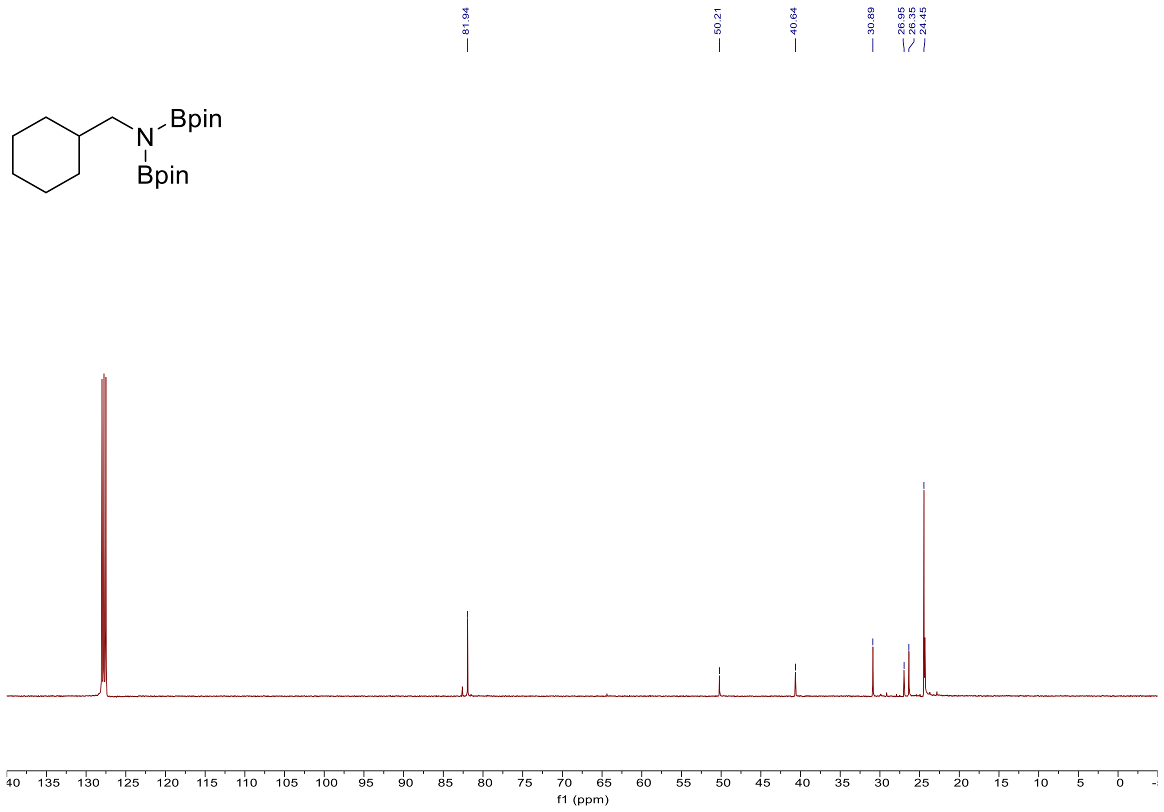

Figure S82 ^{13}C NMR of *N*-(cyclohexylmethyl)-4,4,5,5-tetramethyl-*N*-(4,4,5,5-tetramethyl-1,3,2-dioxaborolan-2-yl)-1,3,2-dioxaborolan-2-amine [S13] (4t)

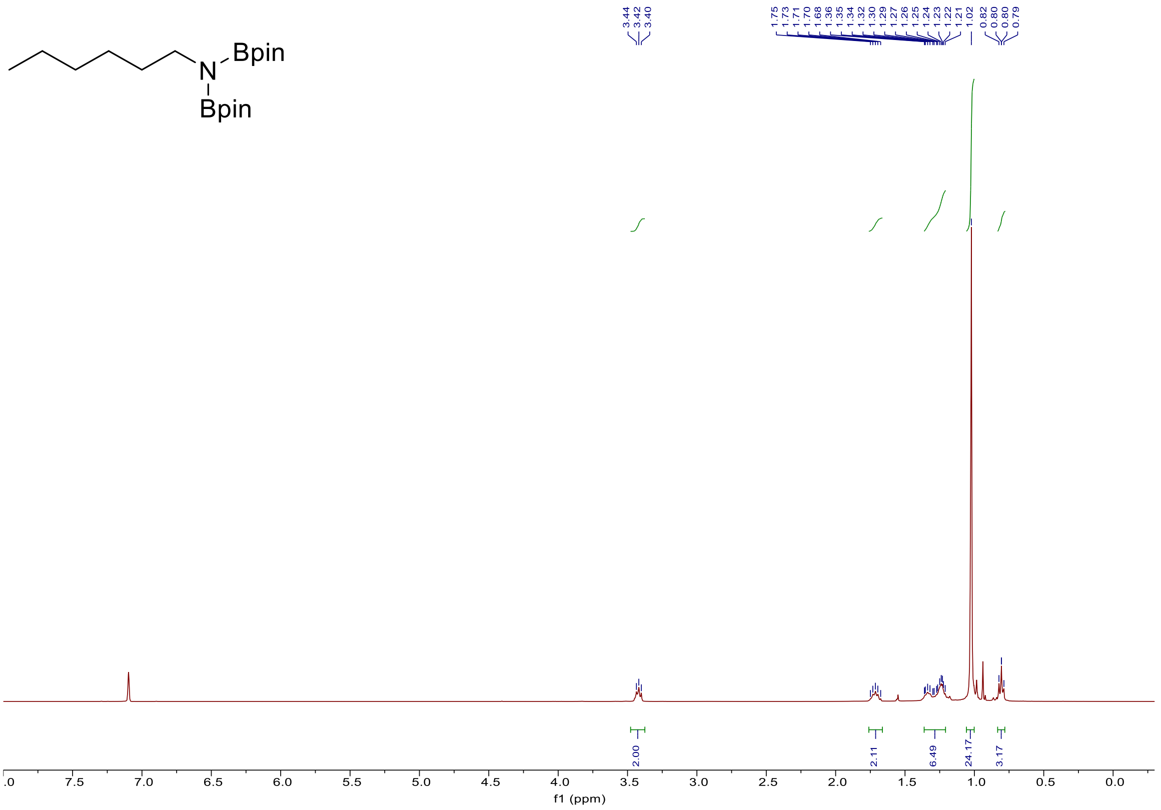

Figure S83 ^{1}H NMR of *N*-Hexyl-4,4,5,5-tetramethyl-*N*-(4,4,5,5-tetramethyl-1,3,2-dioxaborolan-2-yl)-1,3,2-dioxaborolan-2- amine [S9] (4u)

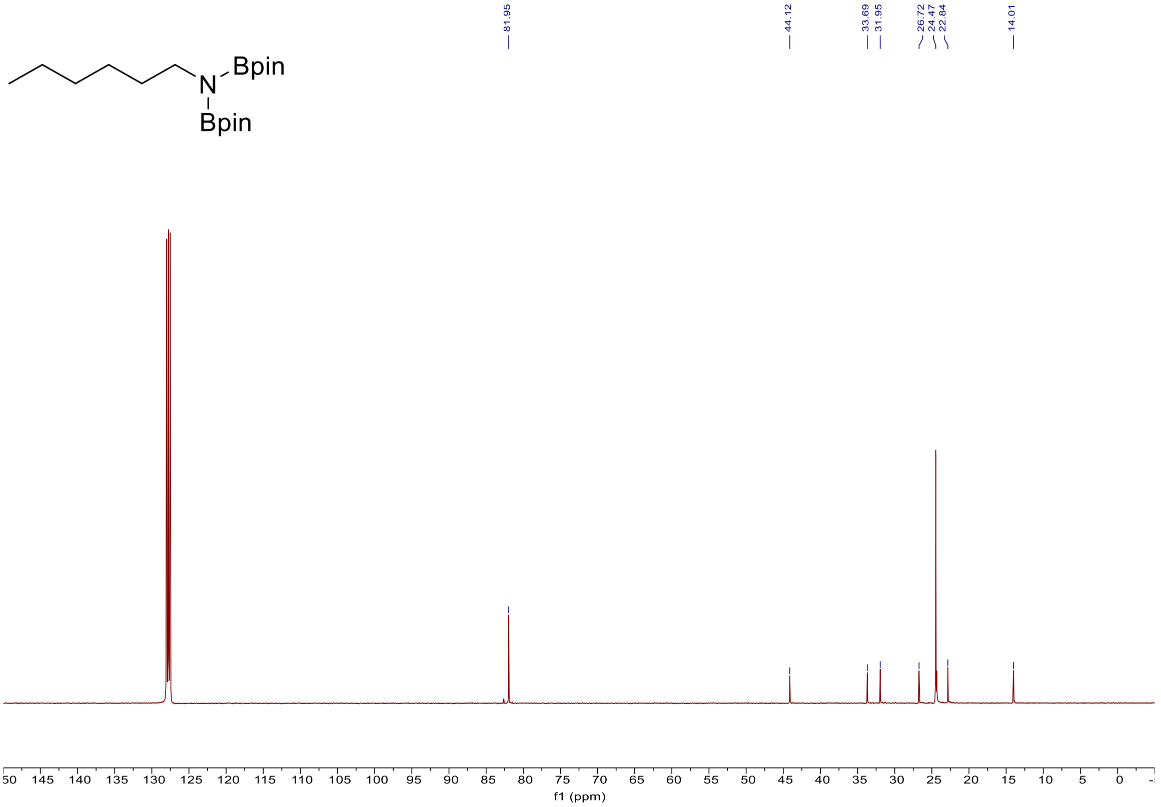

Figure S84 ^{13}C NMR of *N*-Hexyl-4,4,5,5-tetramethyl-*N*-(4,4,5,5-tetramethyl-1,3,2-dioxaborolan-2-yl)-1,3,2-dioxaborolan-2- amine [S9] (4u)

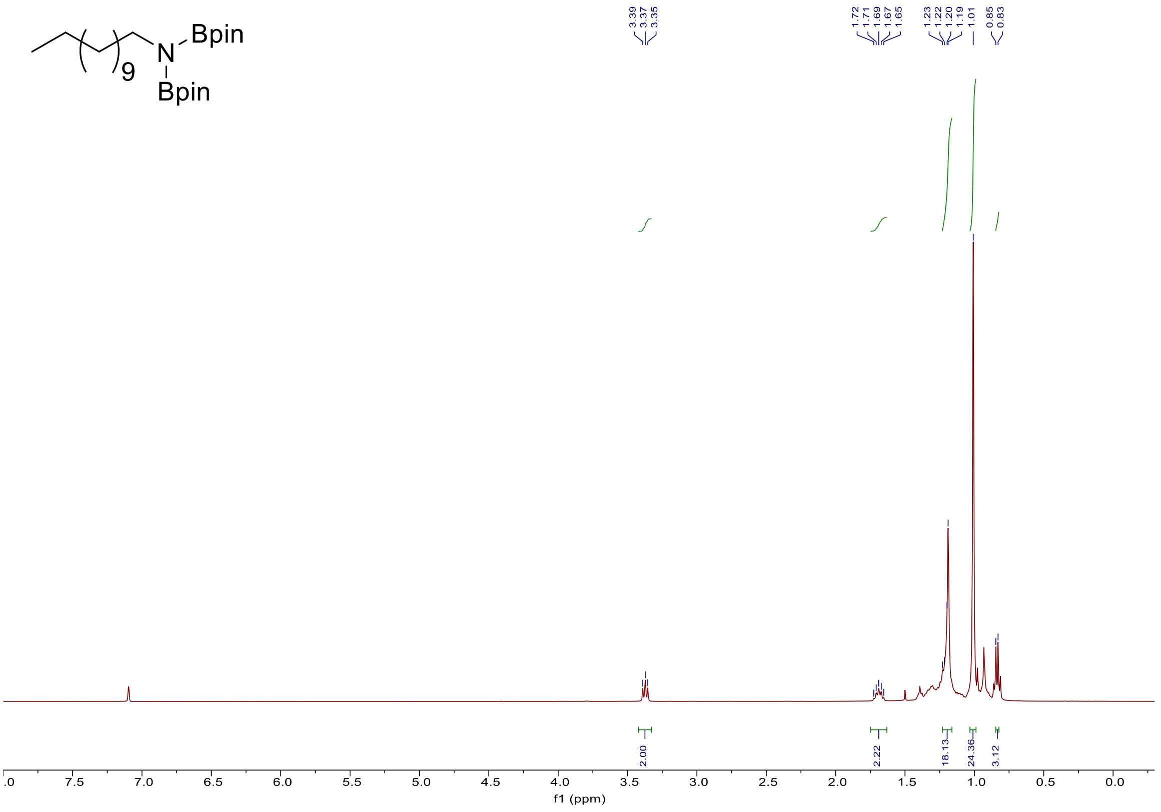

Figure S85 ^{1}H NMR of *N*-dodecyl-4,4,5,5-tetramethyl-*N*-(4,4,5,5-tetramethyl-1,3,2-dioxaborolan-2-yl)-1,3,2-dioxaborolan-2-amine [S14] (4v)

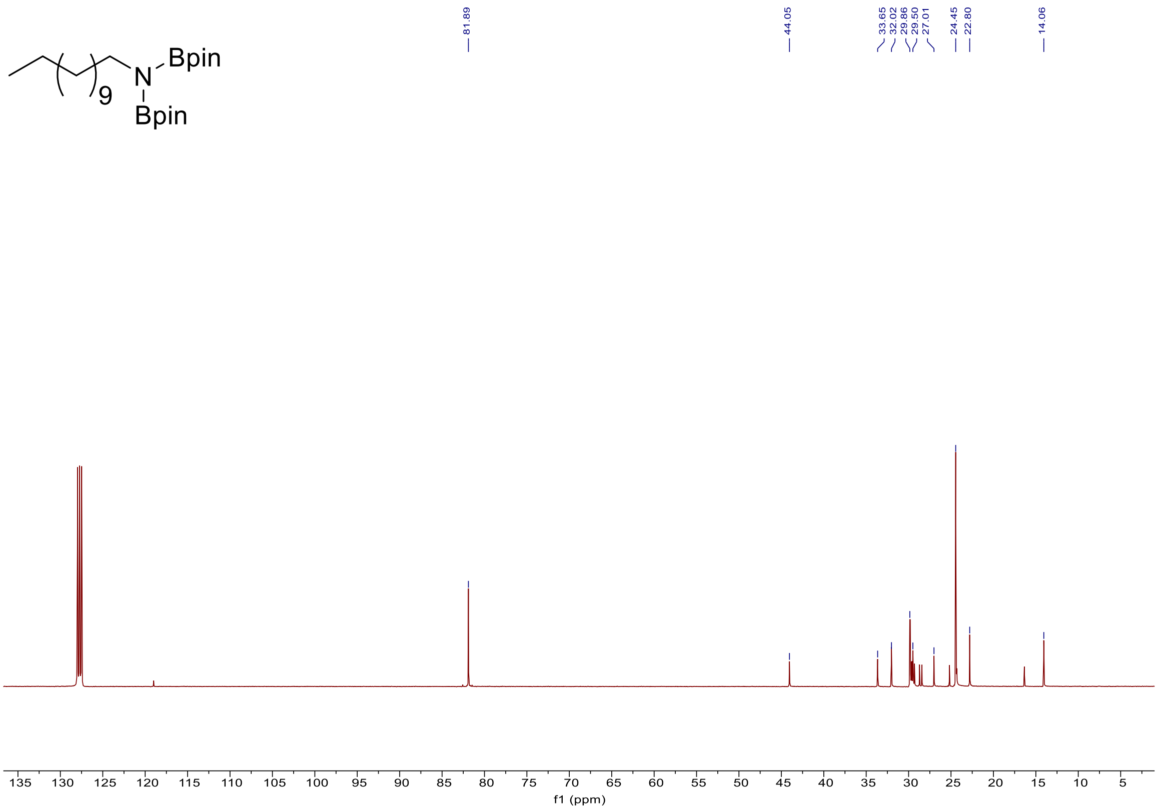

Figure S86 ^{13}C NMR of *N*-dodecyl-4,4,5,5-tetramethyl-*N*-(4,4,5,5-tetramethyl-1,3,2-dioxaborolan-2-yl)-1,3,2-dioxaborolan-2-amine [S15] (4v)

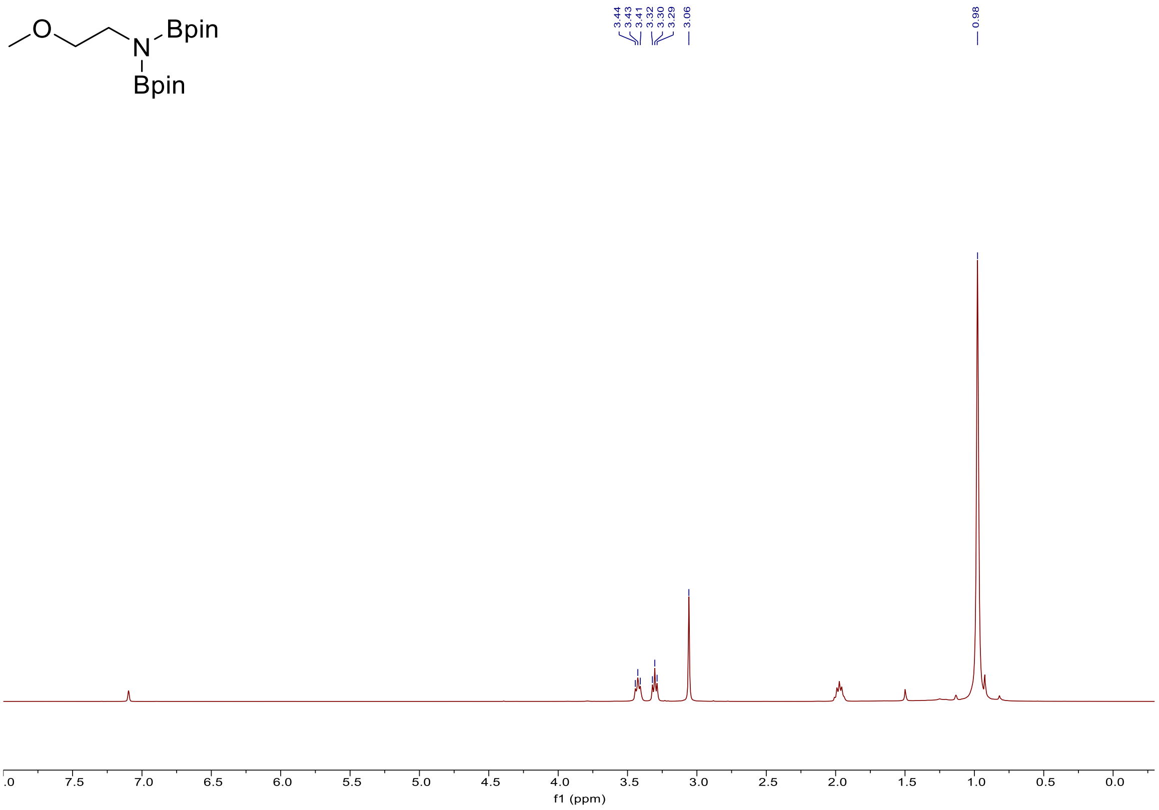

Figure S87 ^{1}H NMR of *N*-(2-methoxyethyl)-4,4,5,5-tetramethyl-*N*-(4,4,5,5-tetramethyl-1,3,2-dioxaborolan-2-yl)-1,3,2- dioxaborolan-2-amine [S9] (4w)

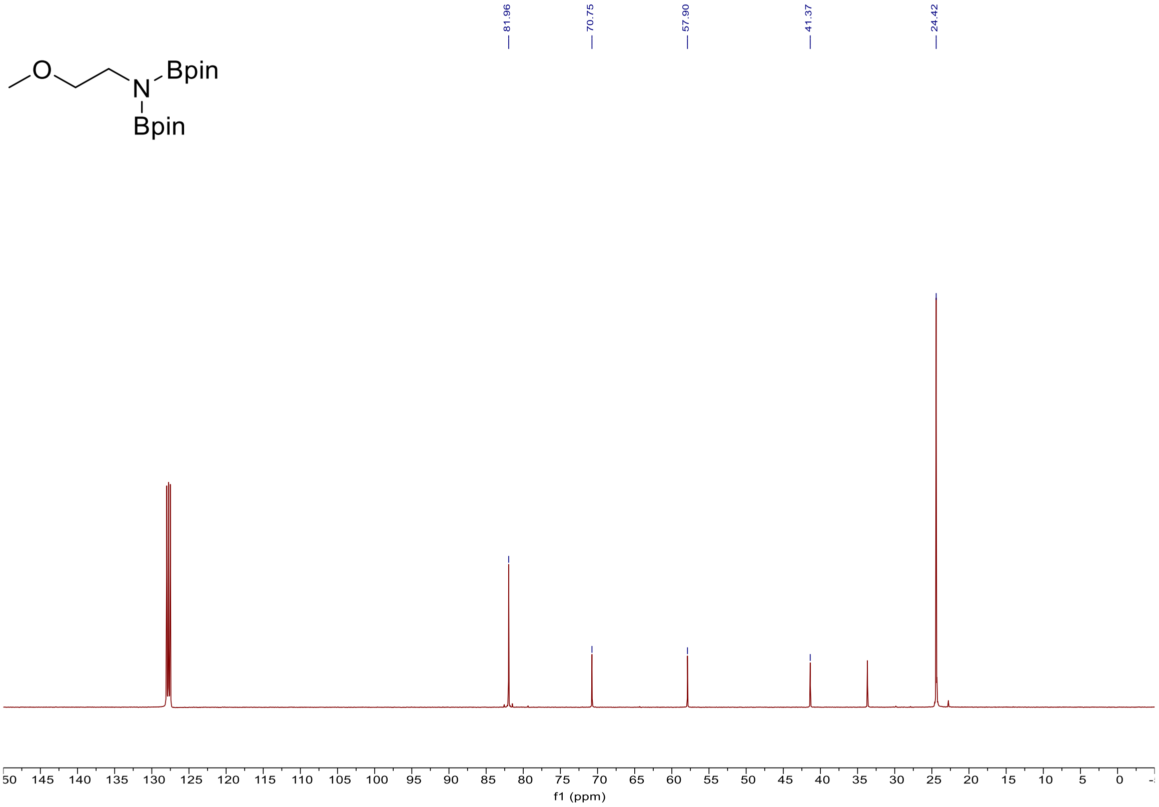

Figure S88 ^{1}H NMR of *N*-(2-methoxyethyl)-4,4,5,5-tetramethyl-*N*-(4,4,5,5-tetramethyl-1,3,2-dioxaborolan-2-yl)-1,3,2- dioxaborolan-2-amine [S9] (4w)

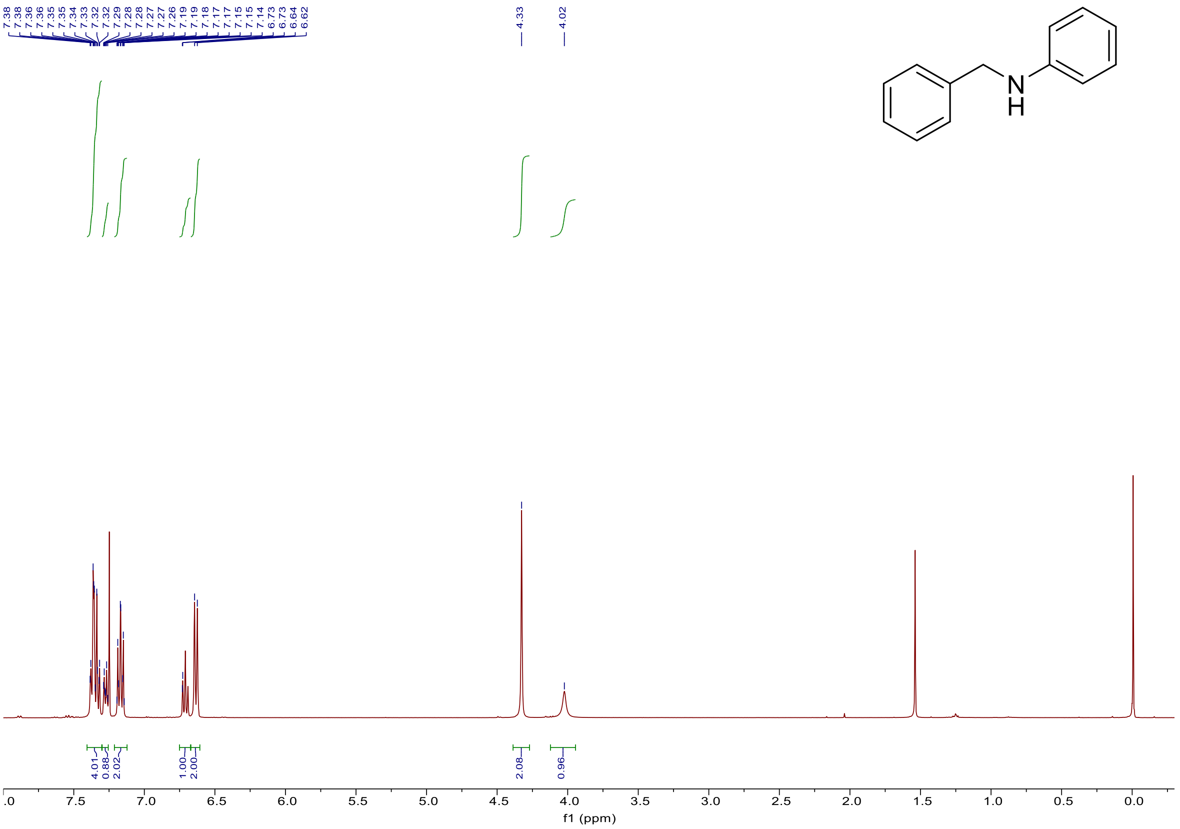

Figure S89 ^{1}H NMR of *N*-Benzylaniline[S16] (4a)

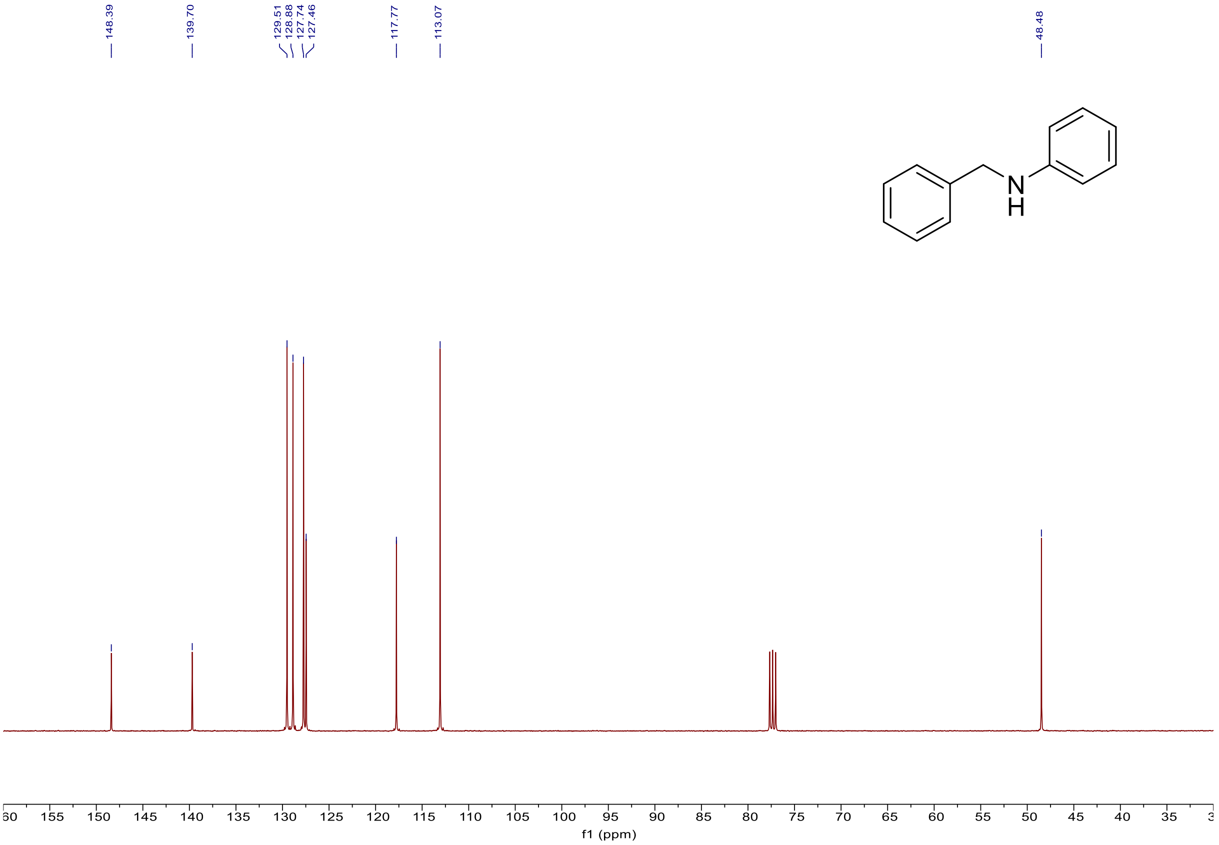

Figure S90 ^{13}C NMR of *N*-Benzylaniline[S16] (4a)

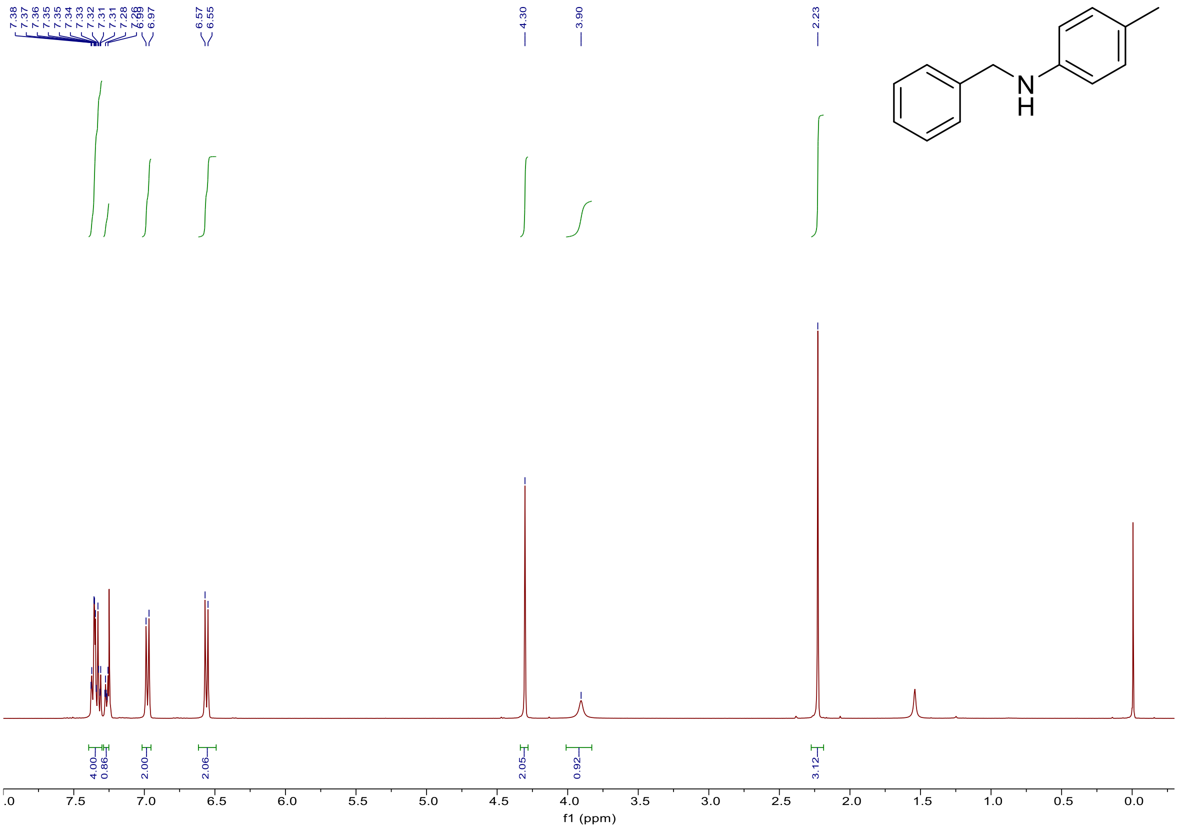

Figure S91 ^{1}H NMR of *N*-Benzyl-4-methylaniline [S16] (4b)

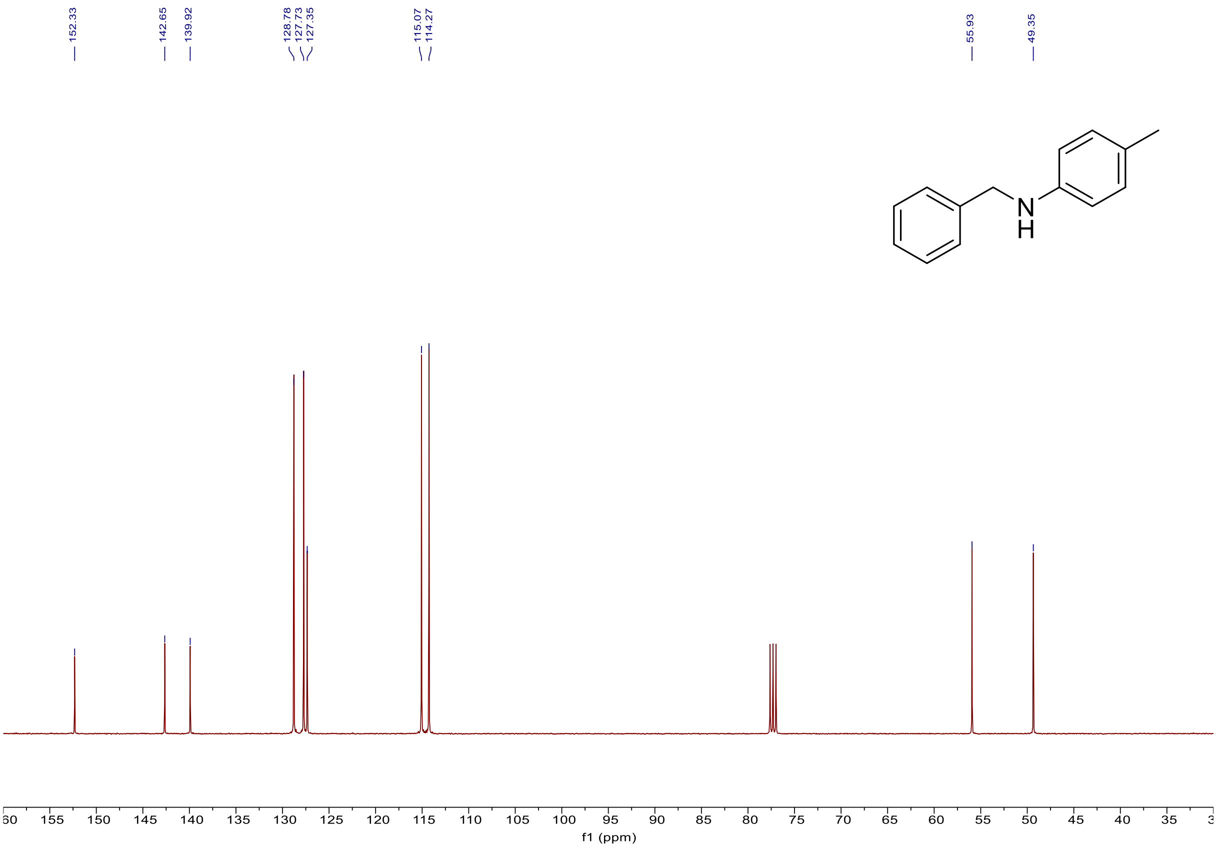

Figure S92 ^{13}C NMR of *N*-Benzyl-4-methylaniline [S16] (4b)

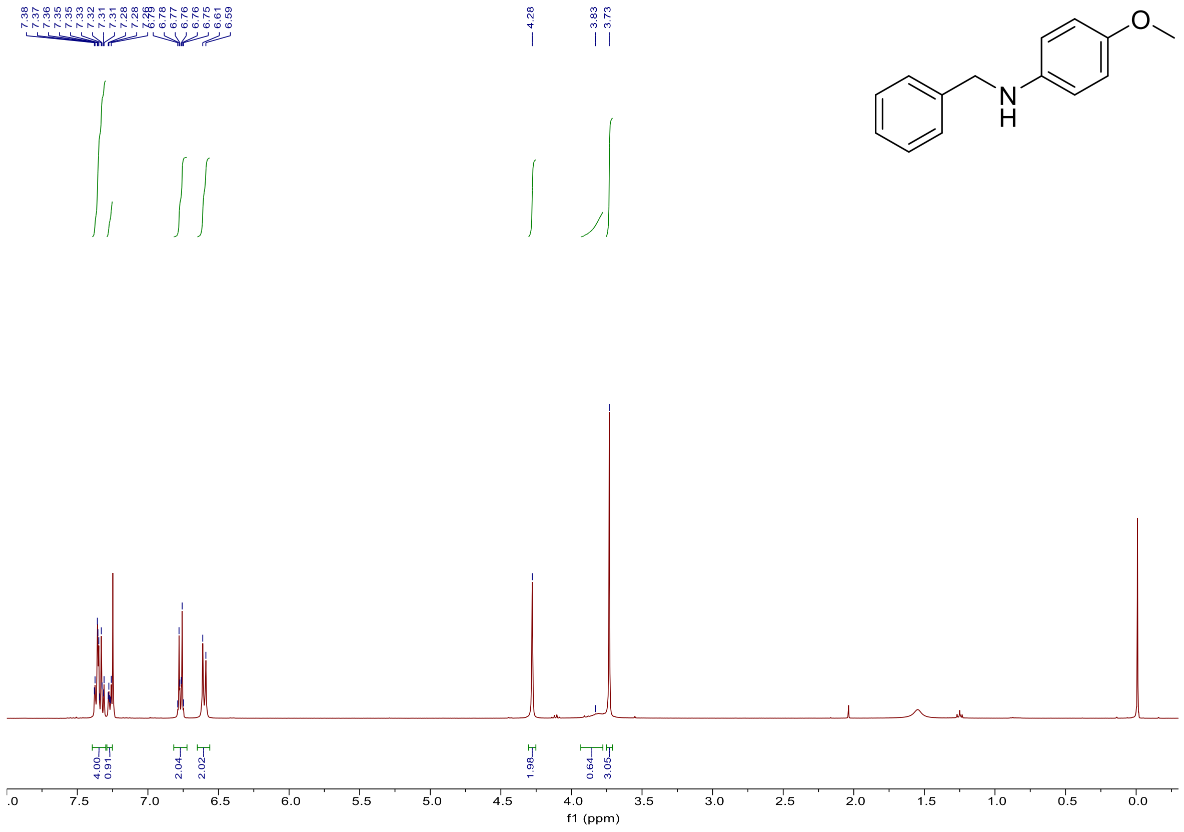

Figure S93 ^{1}H NMR of *N*-Benzyl-4-methoxyaniline [S16] (4c)

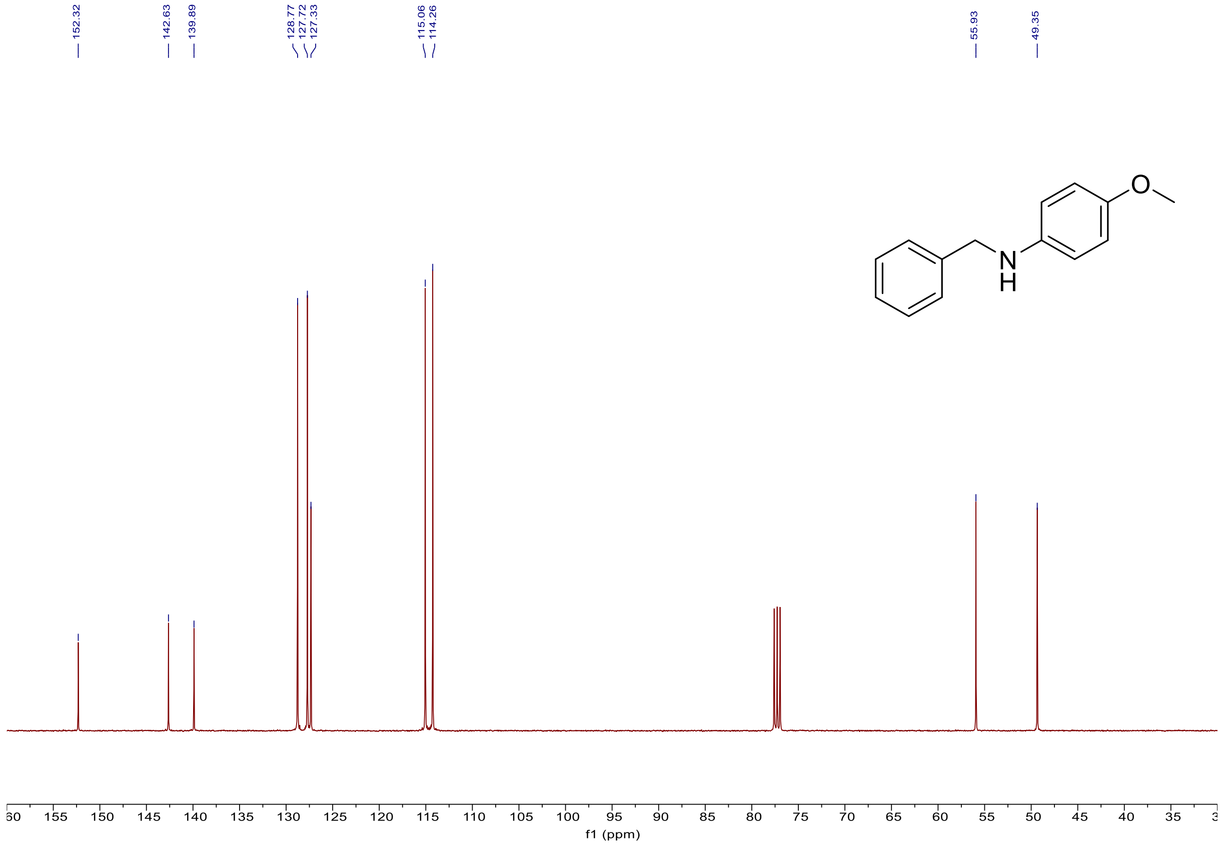

Figure S94 ^{13}C NMR of *N*-Benzyl-4-methoxyaniline [S16] (4c)

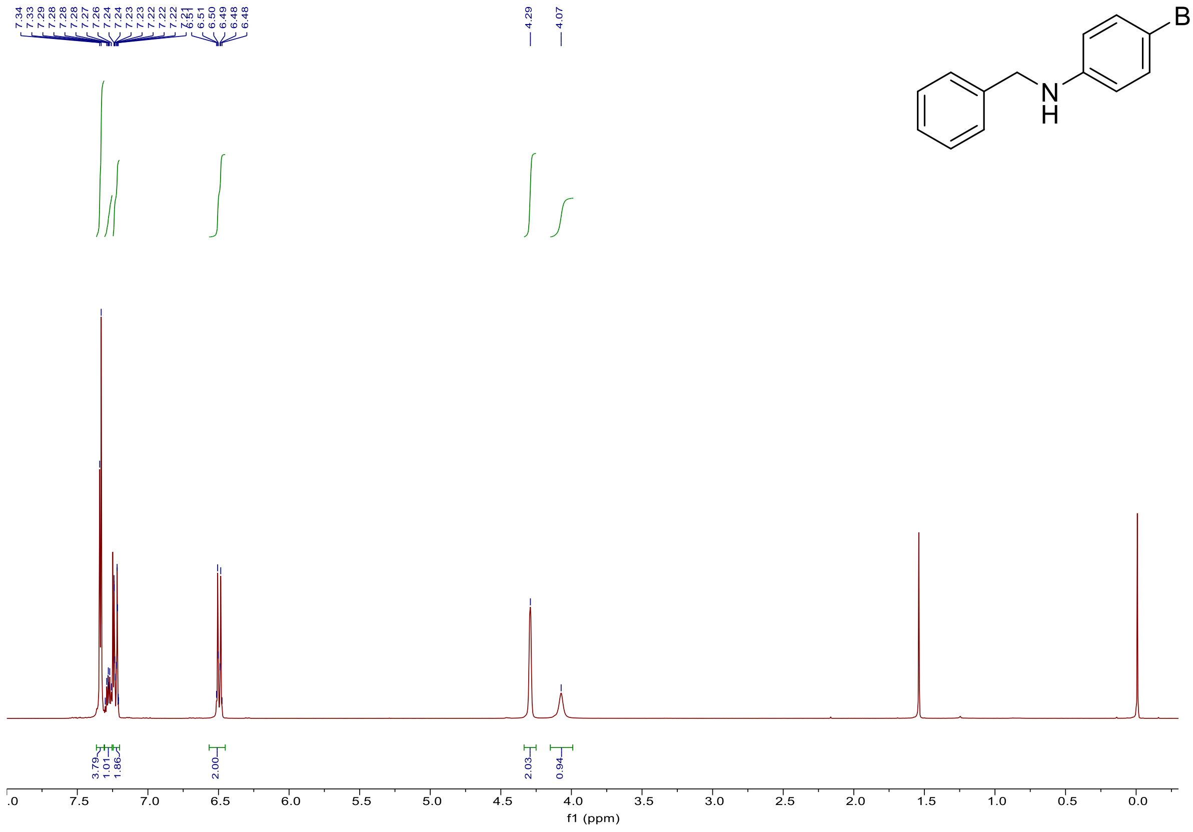

Figure S95 ^{1}H NMR of *N*-Benzyl-4-bromoaniline [S16] (4d)

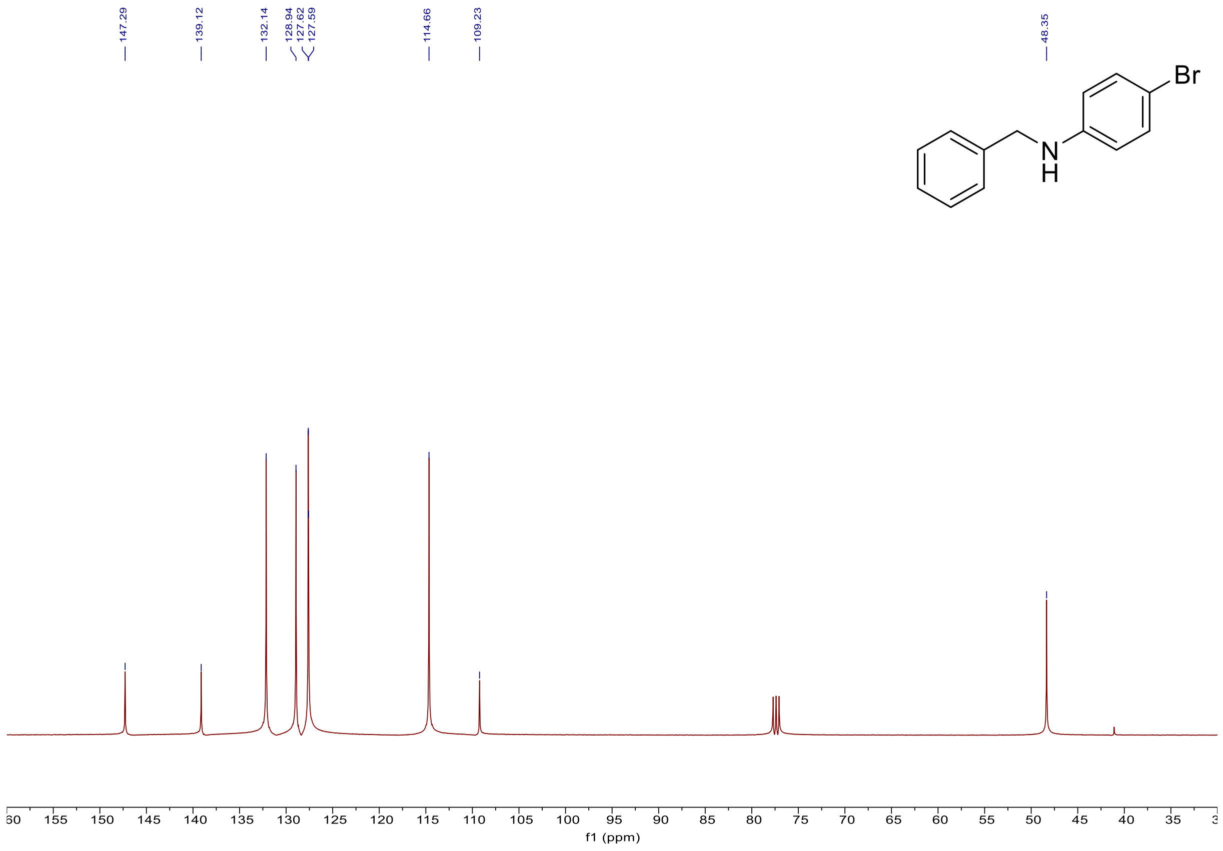

Figure S96 ^{13}C NMR of *N*-Benzyl-4-bromoaniline [S16] (4d)

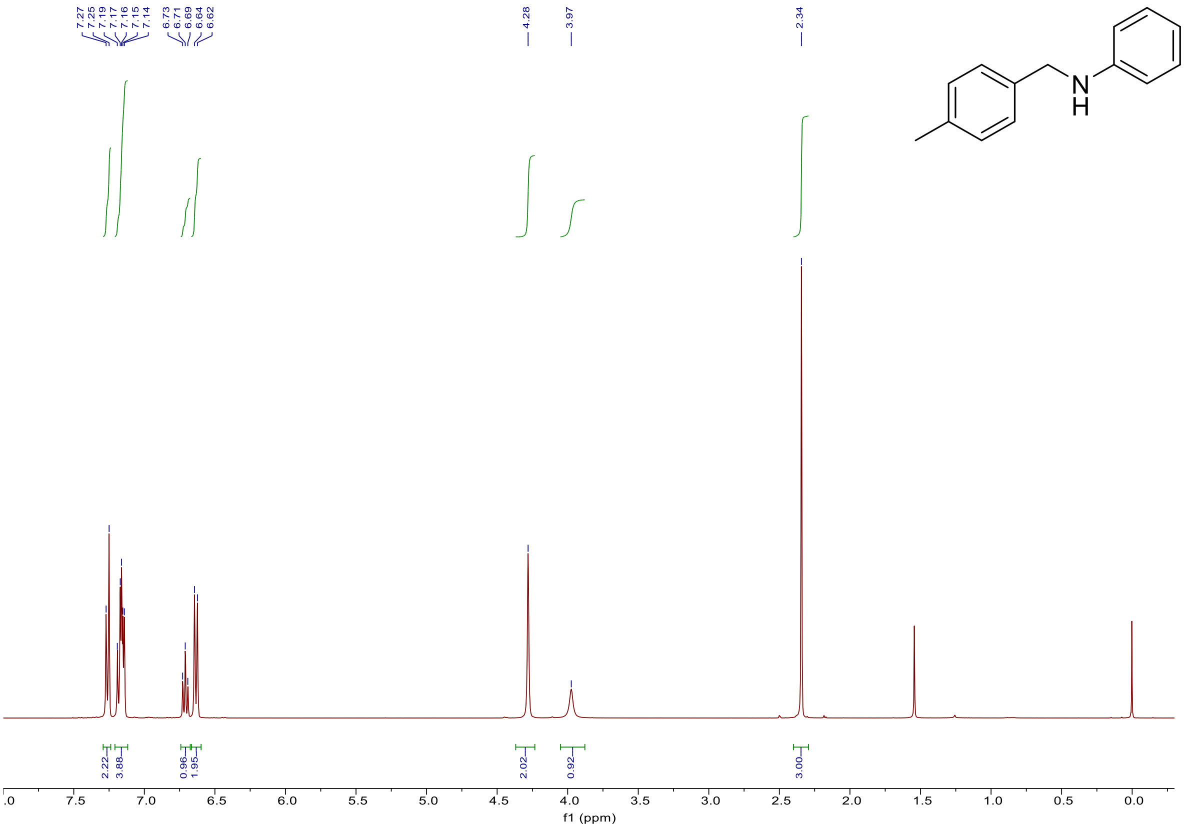

Figure S97 ^{1}H NMR of *N*-(4-Methylbenzyl)aniline [S16] (4e)

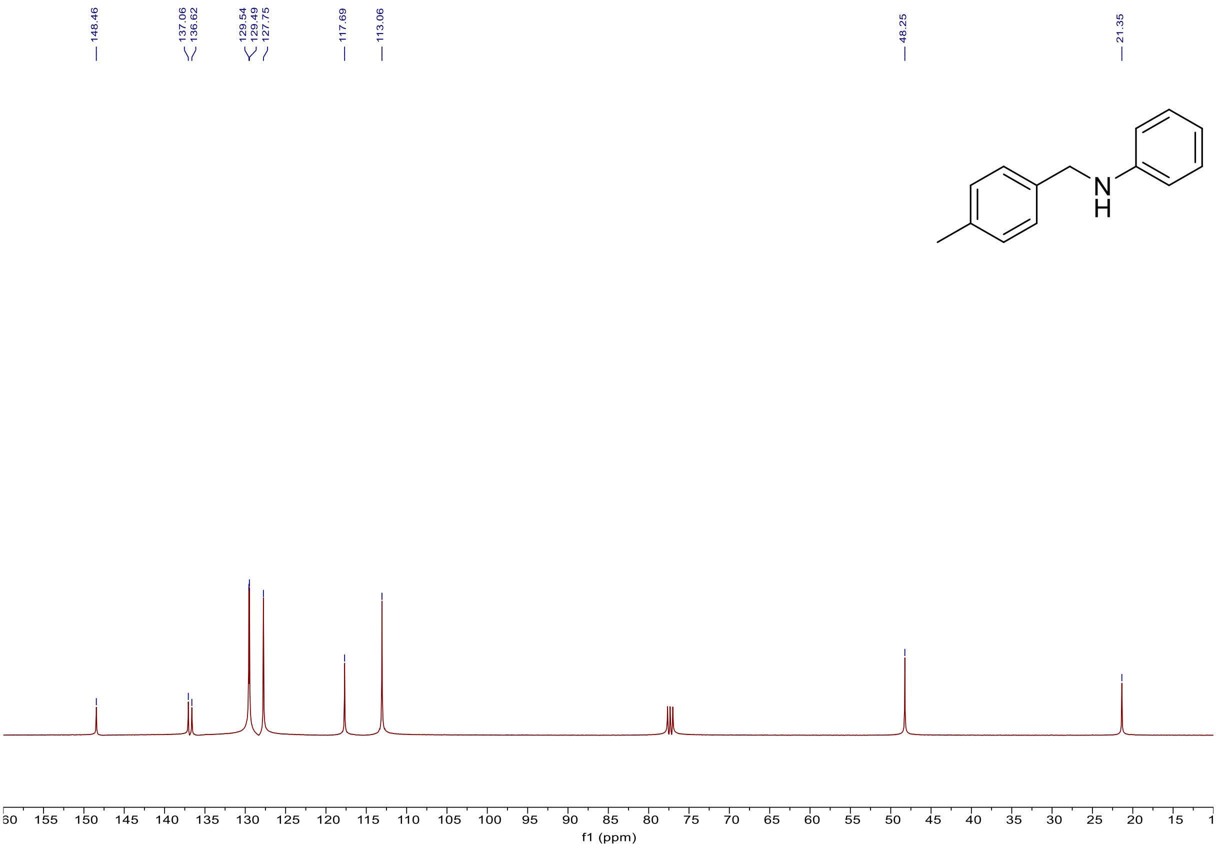

Figure S98 ^{13}C NMR of *N*-(4-Methylbenzyl)aniline [S16] (4e)

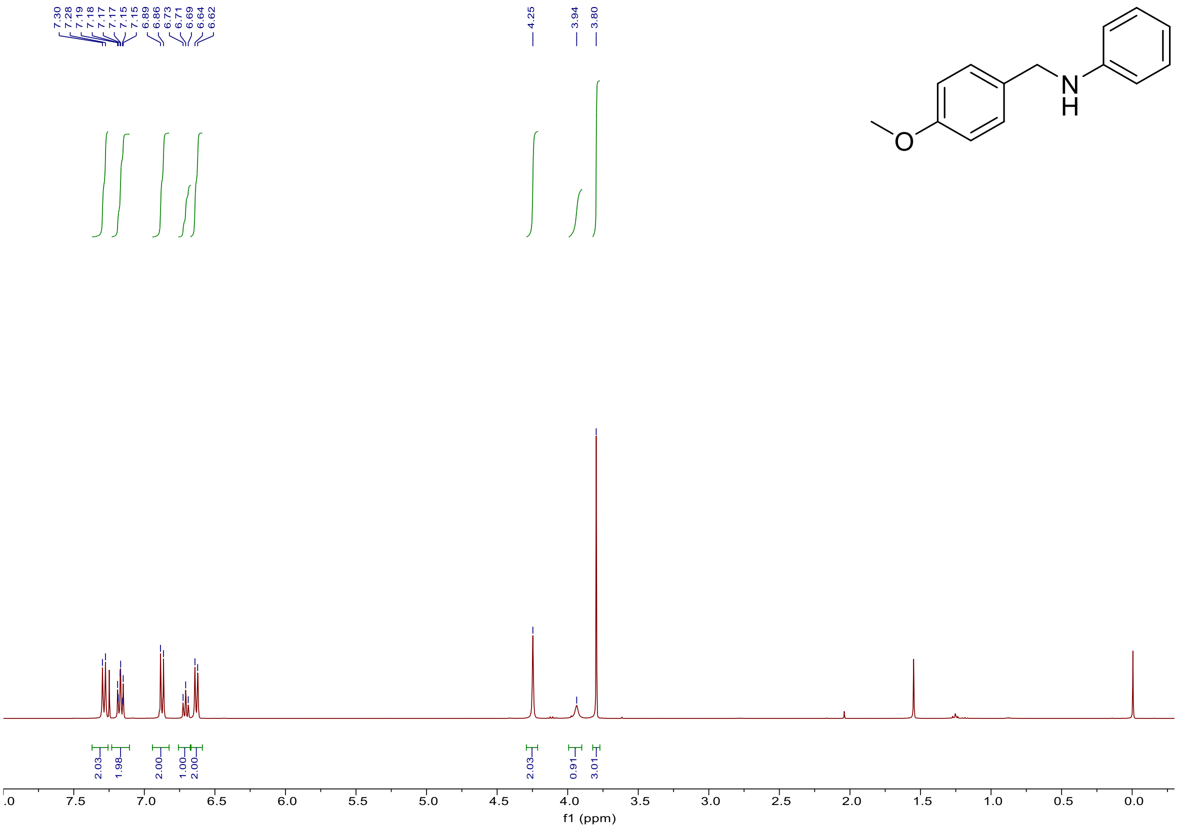

Figure S99 ^{1}H NMR of *N*-(4-Methoxybenzyl)aniline [S16] (4f)

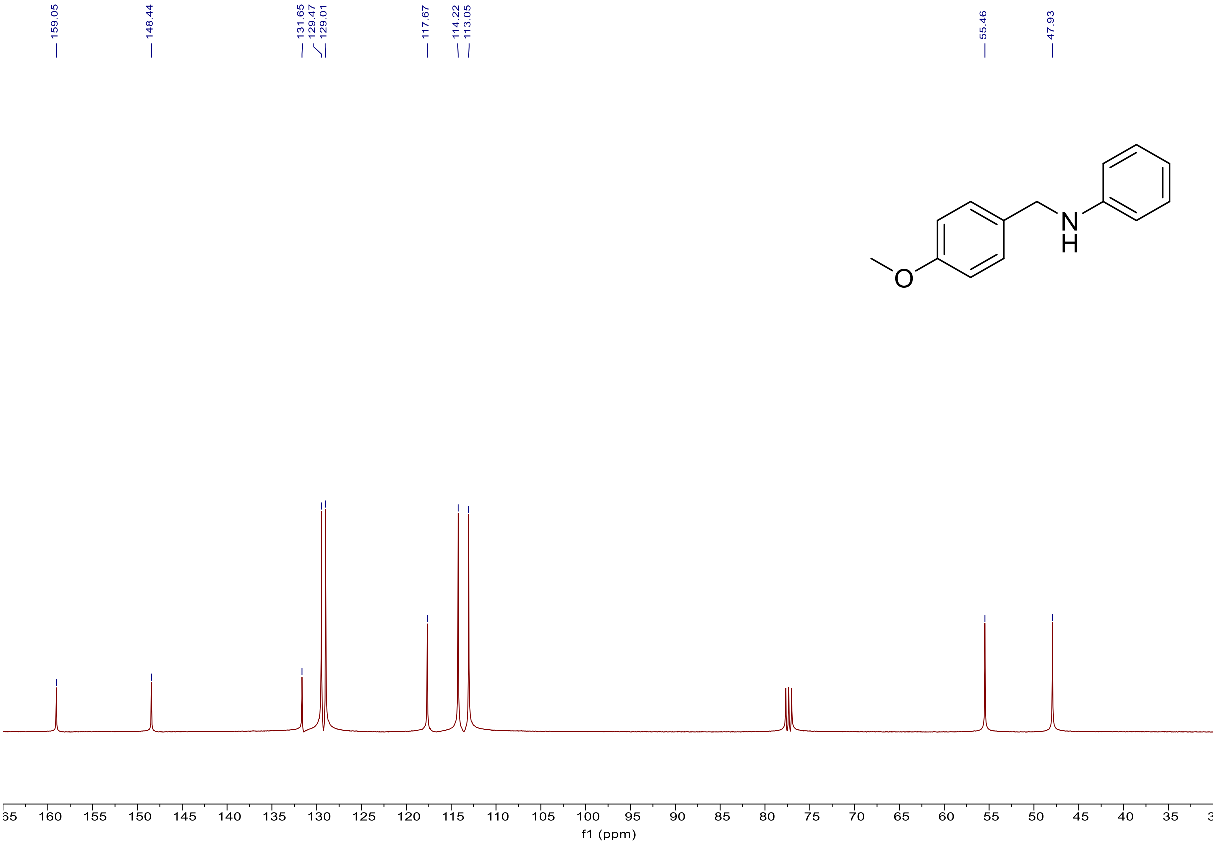

Figure S100 ^{13}C NMR of *N*-(4-Methoxybenzyl)aniline [S16] (4f)

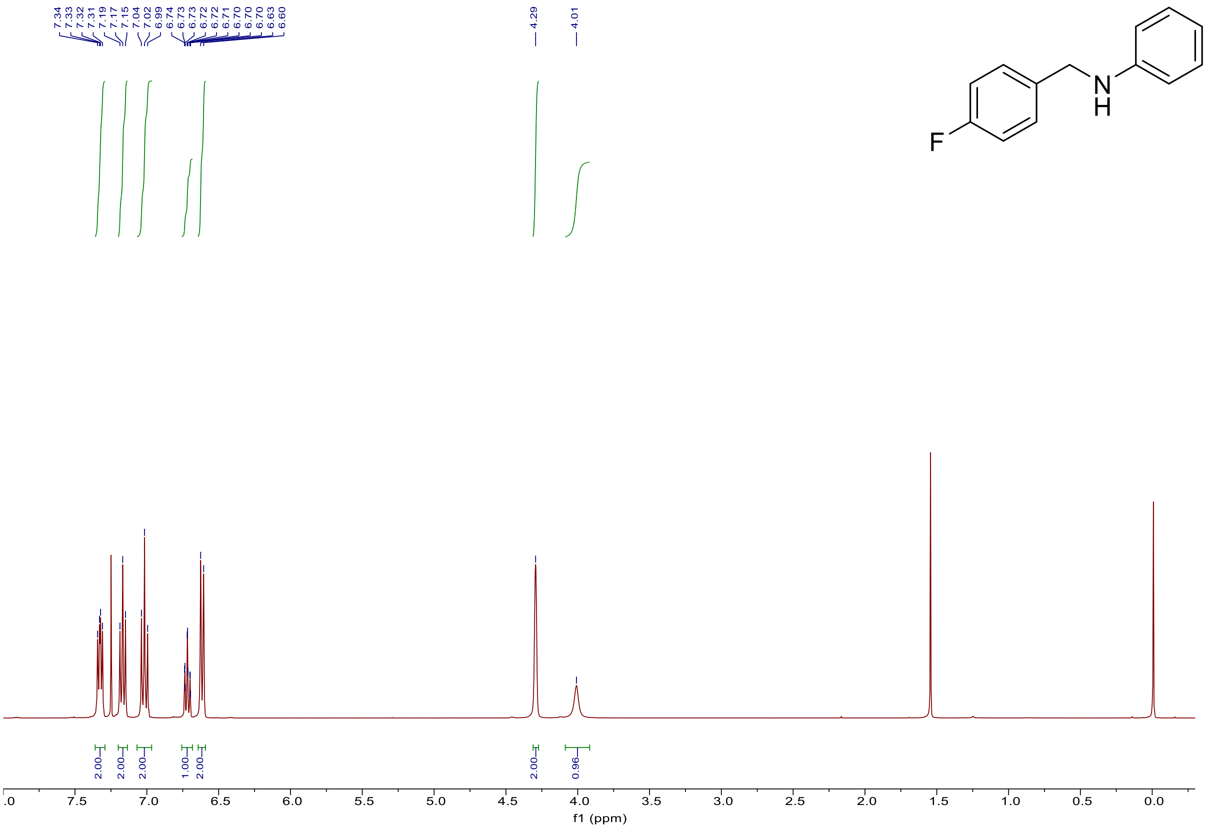

Figure S101 ^{1}H NMR of *N*-(4-Fluorobenzyl)aniline [S16] (4g)

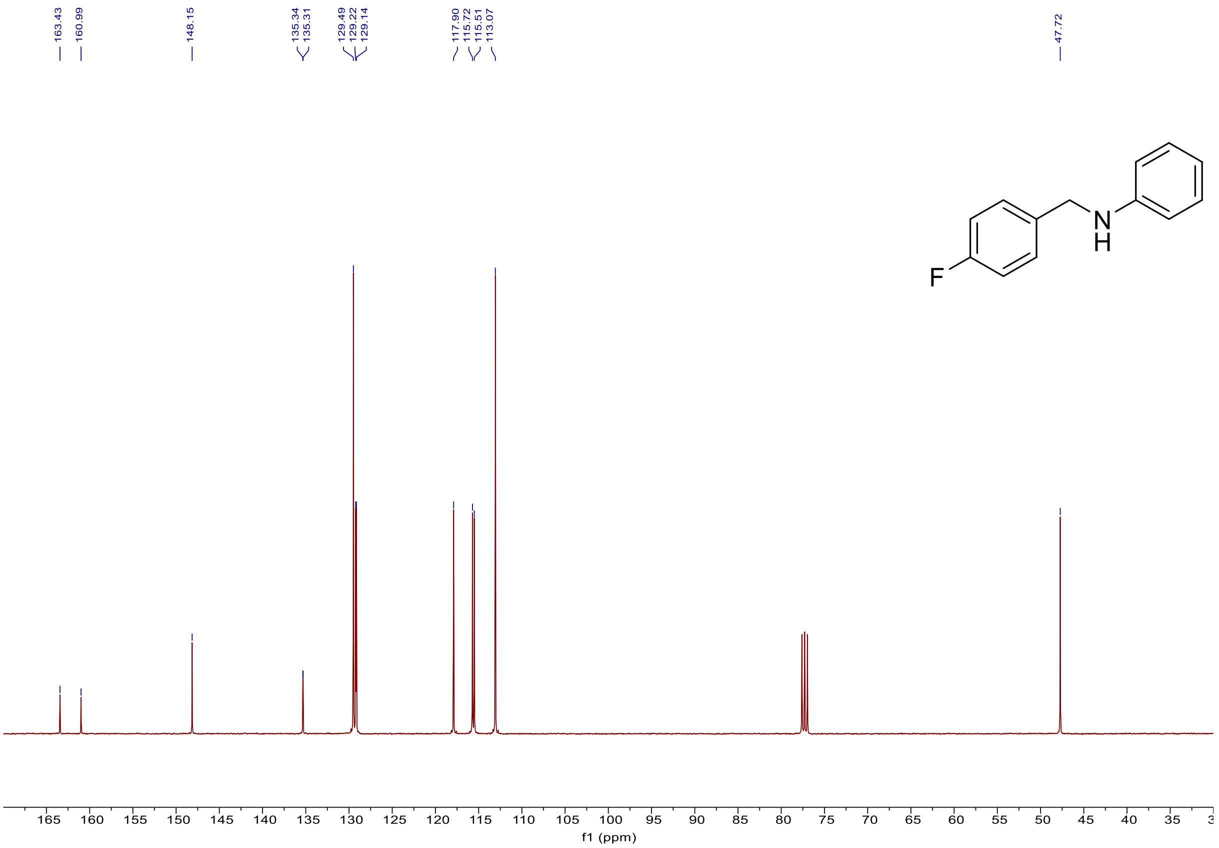

Figure S102 ^{13}C NMR of *N*-(4-Fluorobenzyl)aniline [S16] (4g)

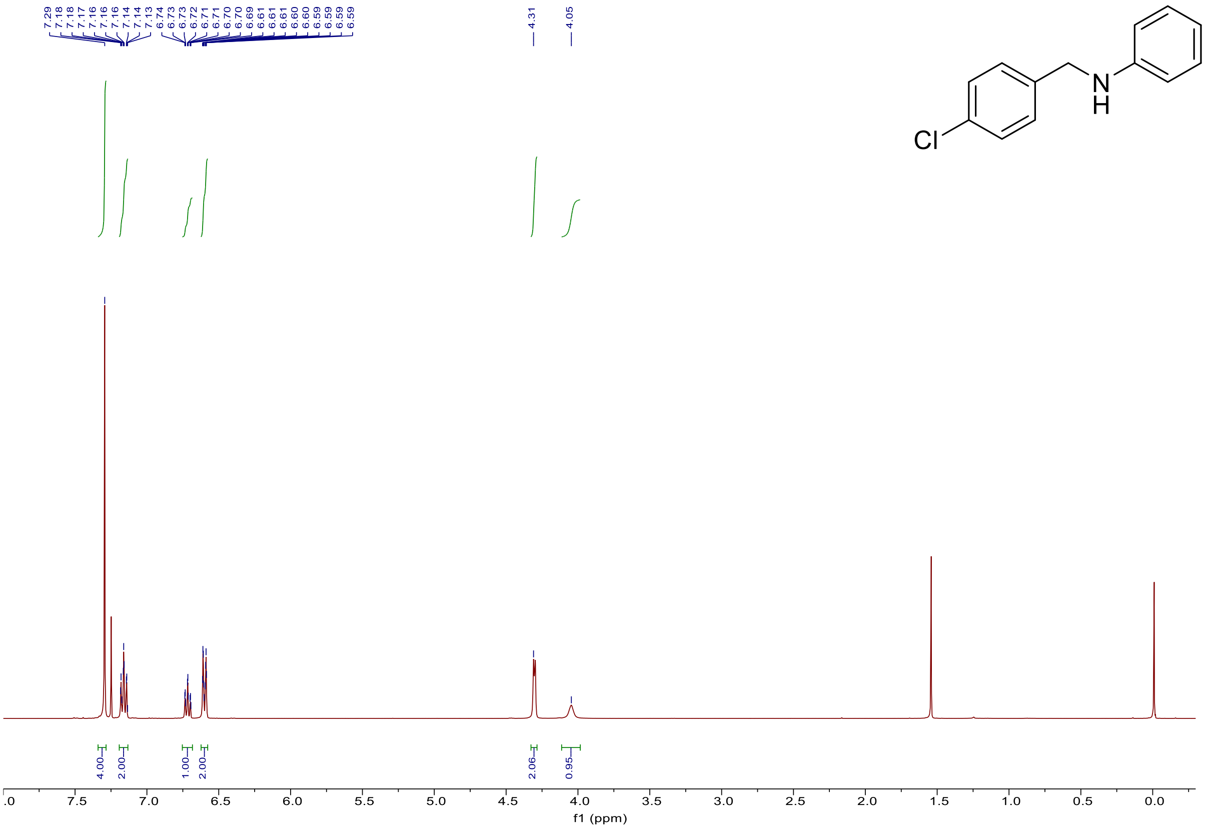

Figure S103 ^{1}H NMR of *N*-(4-Chlorobenzyl)aniline [S16] (4h)

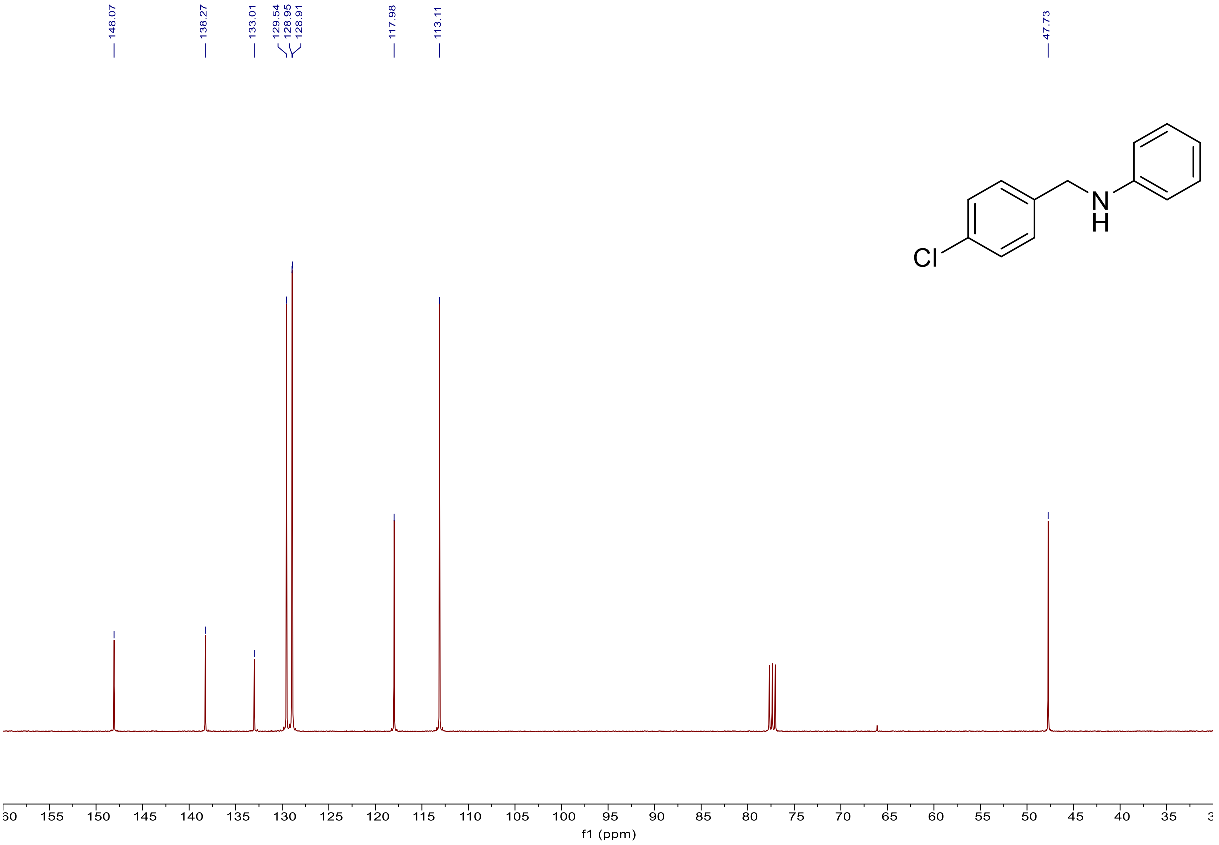

Figure S104 ^{13}C NMR of *N*-(4-Chlorobenzyl)aniline [S16] (4h)

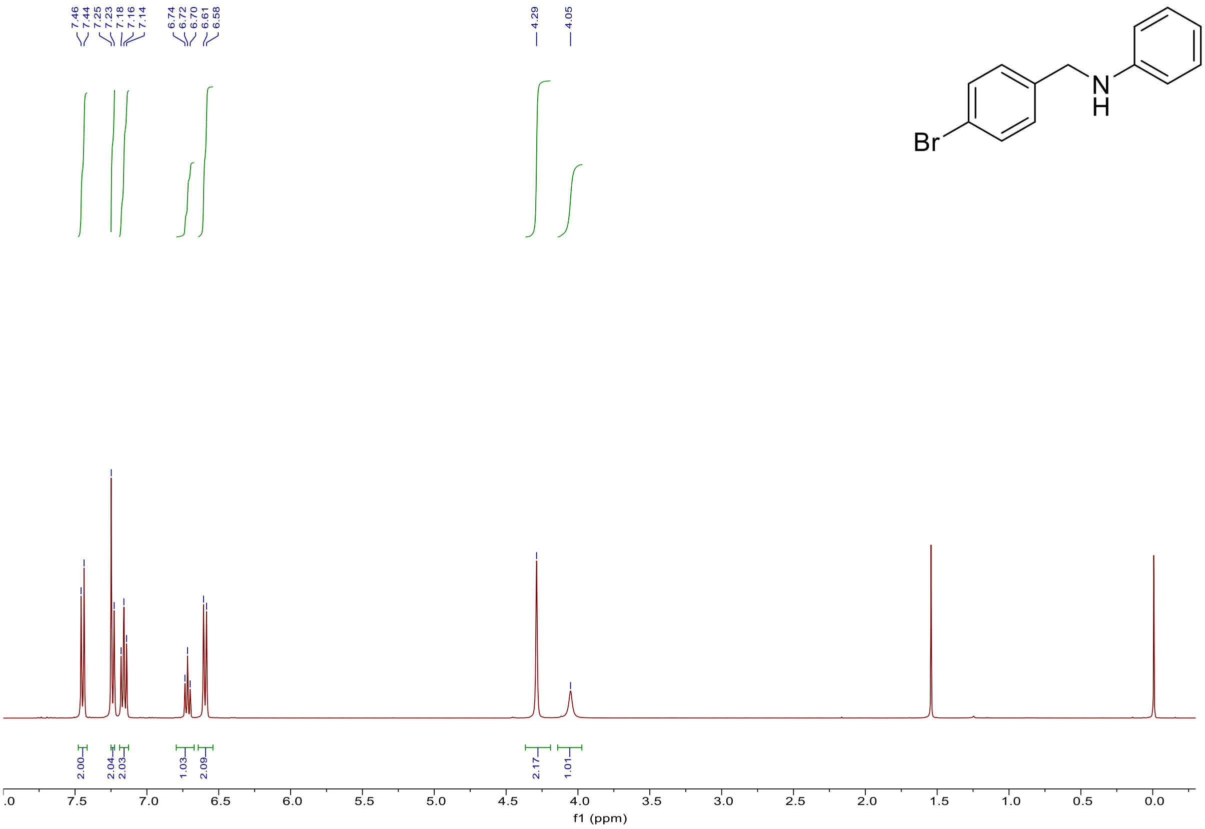

Figure S105 ^{1}H NMR of *N*-(4-Bromobenzyl)aniline [S16] (4i)

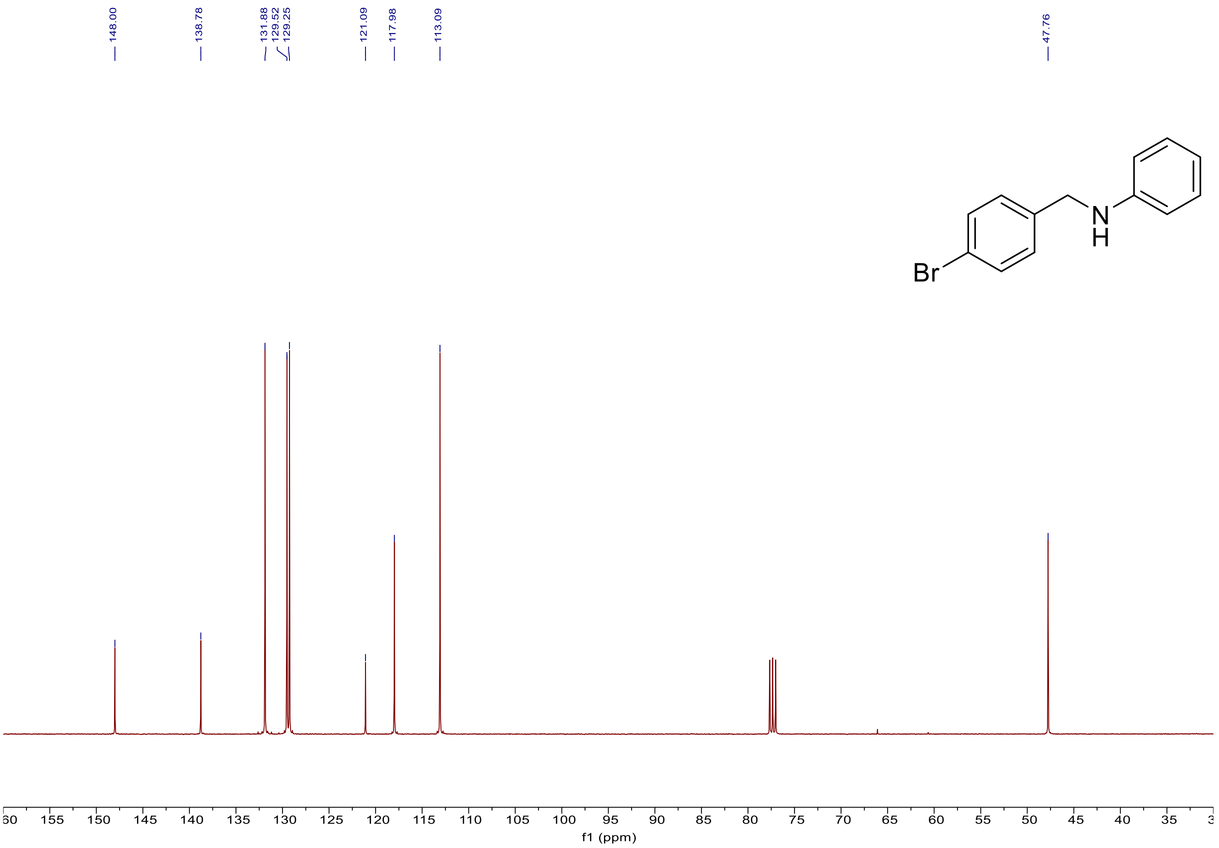

Figure S106 ^{13}C NMR of *N*-(4-Bromobenzyl)aniline [S16] (4i)

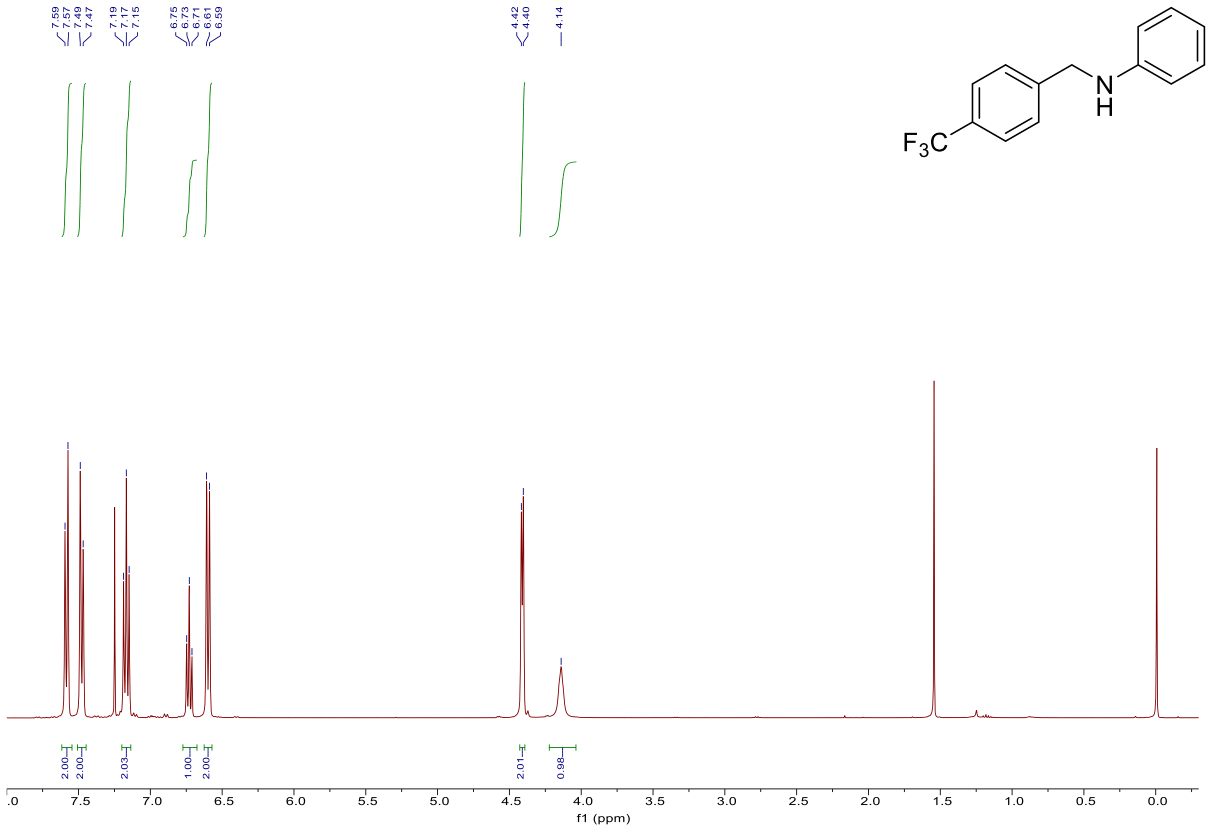

Figure S107 ^{1}H NMR of *N*-(4-(Trifluoromethyl)benzyl)aniline [S16] (4j)

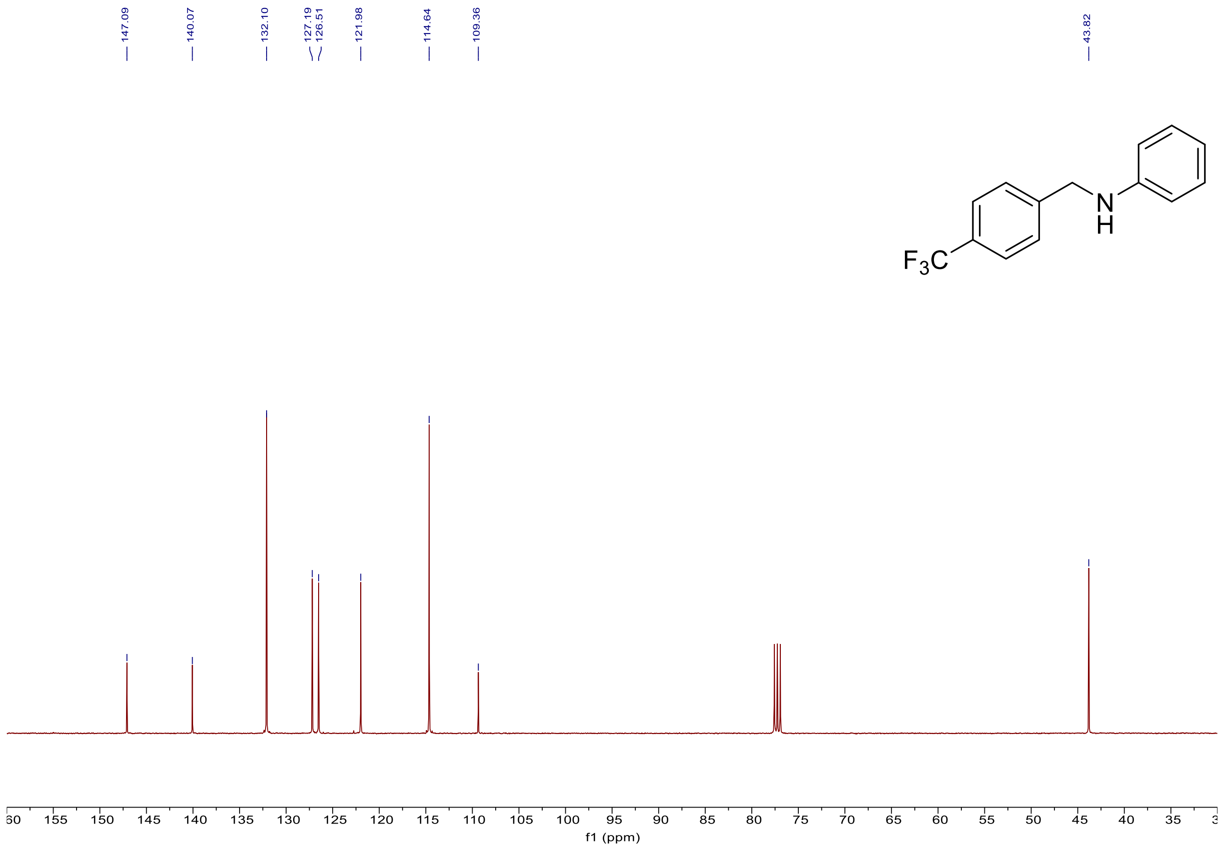

Figure S108 ^{13}C NMR of *N*-(4-(Trifluoromethyl)benzyl)aniline [S16] (4j)

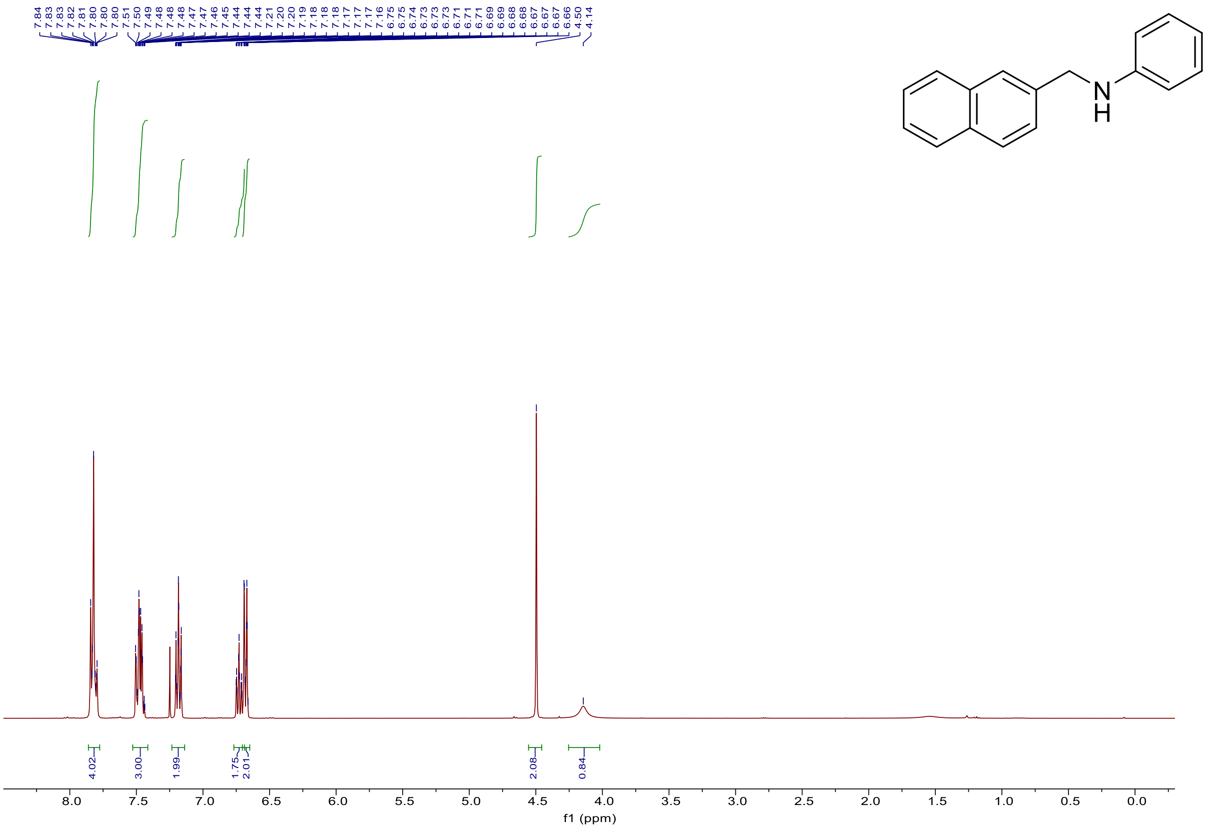

Figure S109 ^{1}H NMR of *N*-(Naphthalen-2-ylmethyl)aniline [S16] (4k)

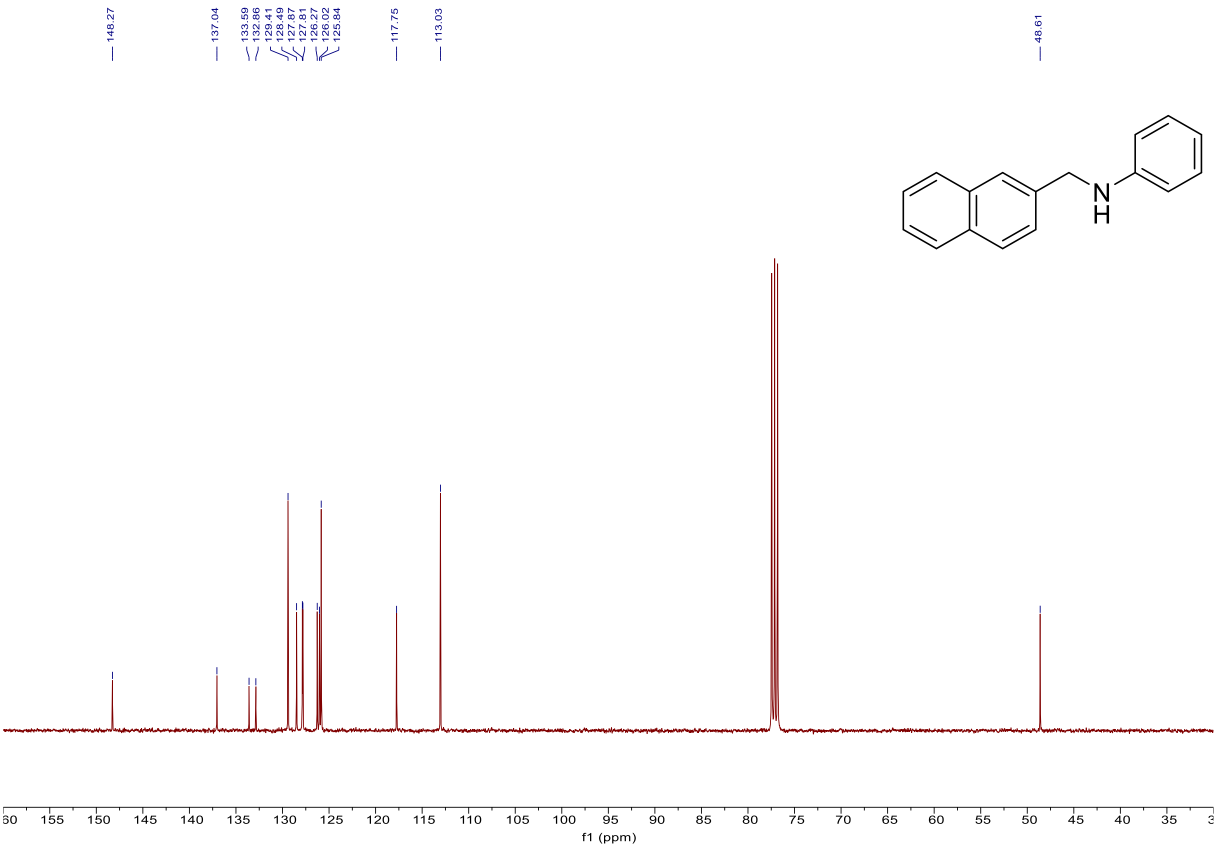

Figure S110 ^{13}C NMR of *N*-(Naphthalen-2-ylmethyl)aniline [S16] (4k)

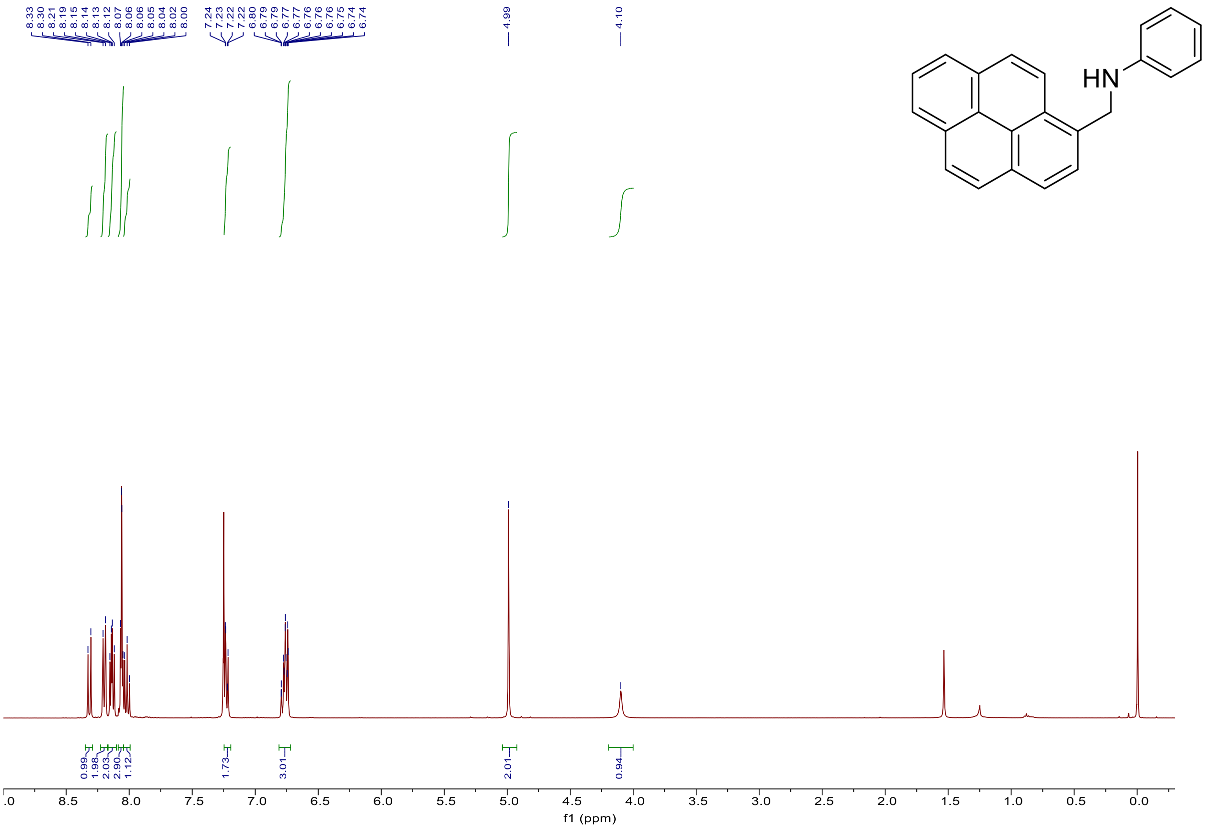

Figure S111 ^{1}H NMR of *N*-(Pyren-1-ylmethyl)aniline [S16] (4l)

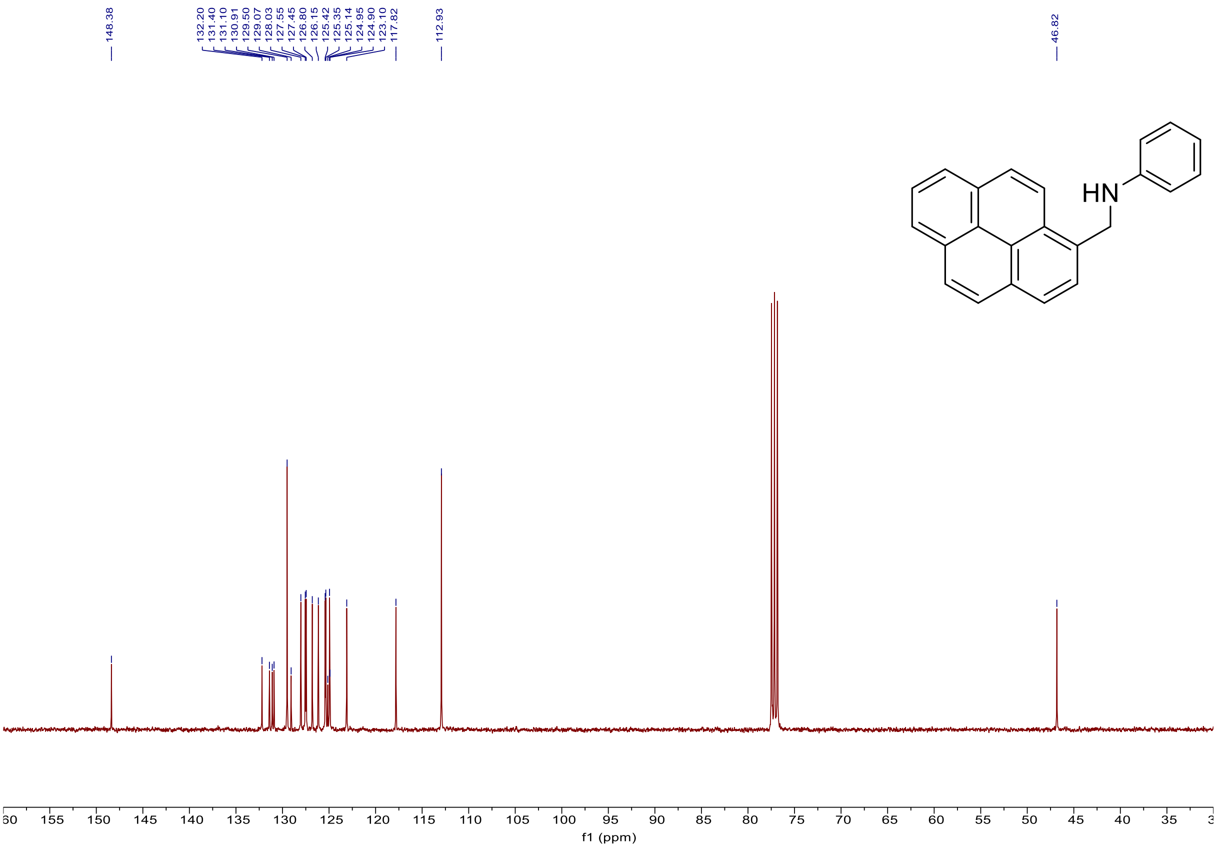

Figure S112 ^{13}C NMR of *N*-(Pyren-1-ylmethyl)aniline [S16] (4l)

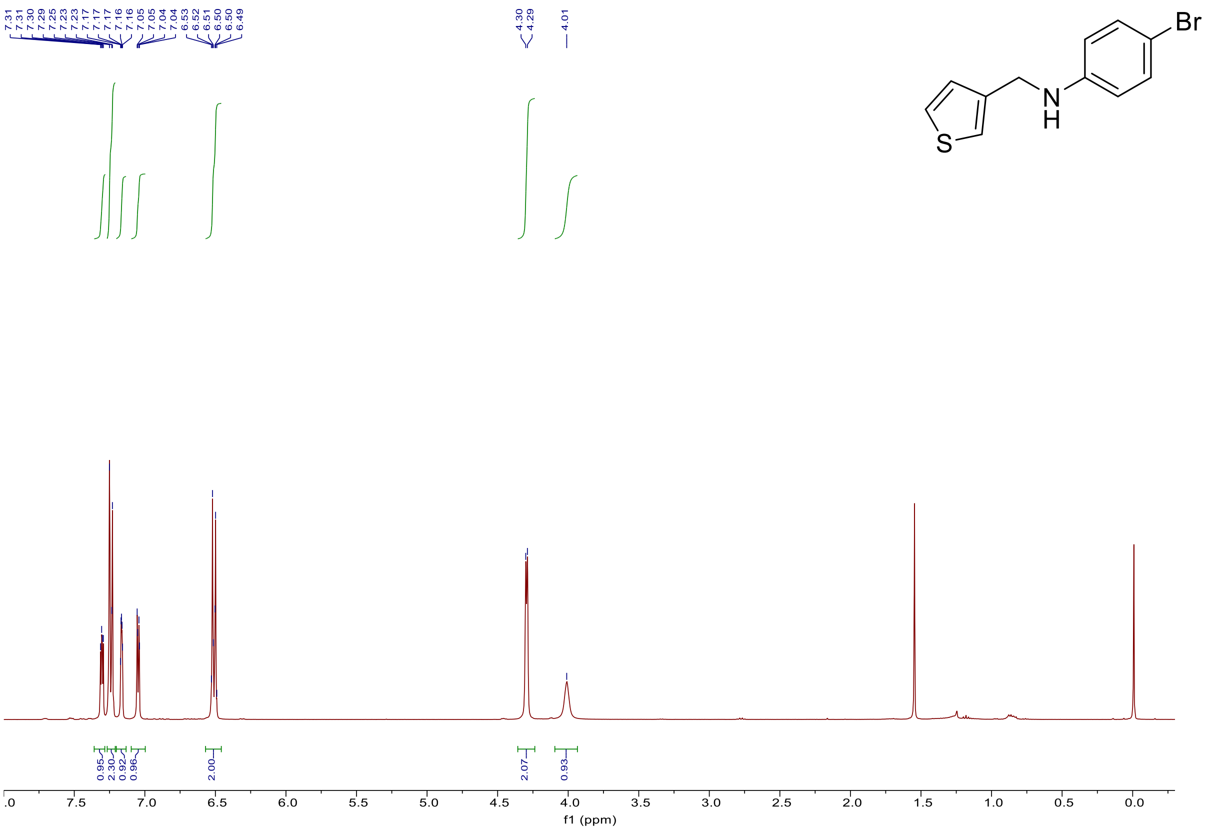

Figure S113 ^{1}H NMR of *N*-(1-Phenylethyl)aniline [S16] (4m)

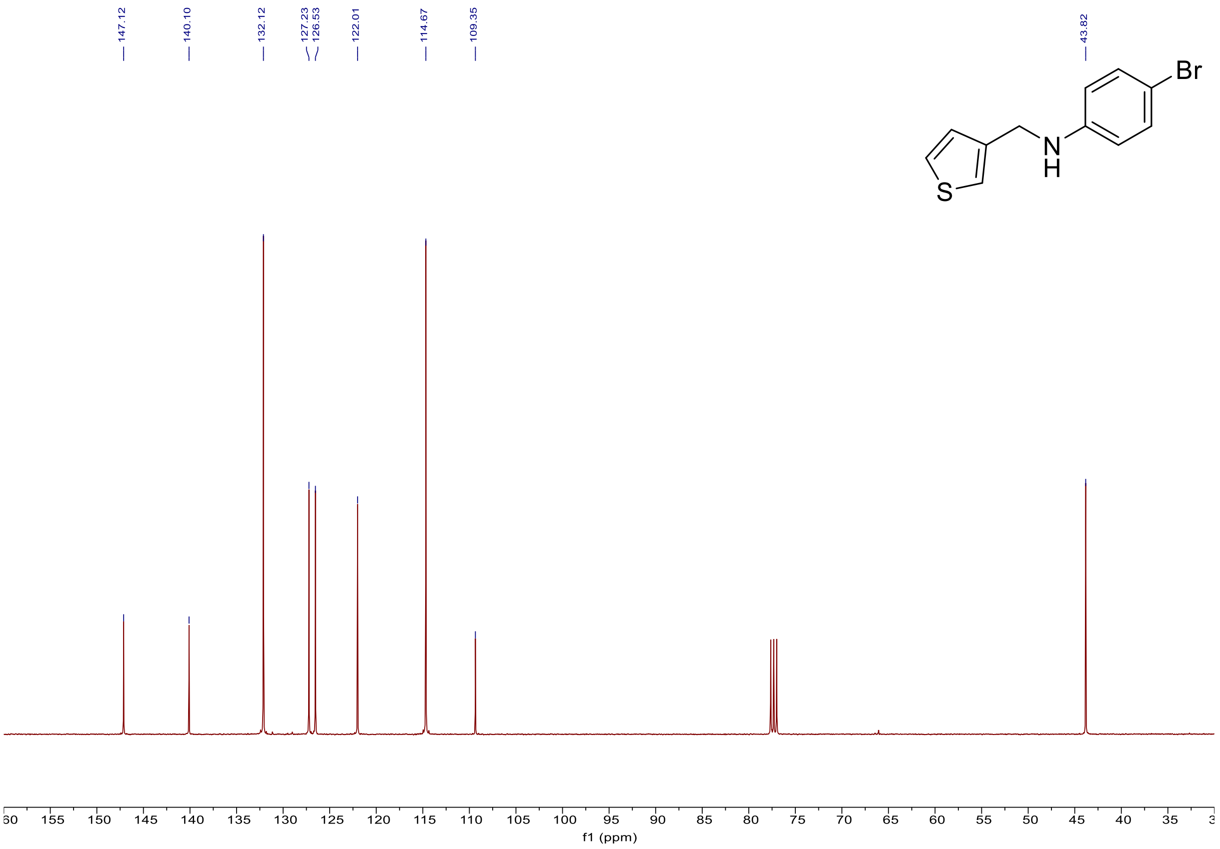

Figure S114 ^{13}C NMR of *N*-(1-Phenylethyl)aniline [S16] (4m)

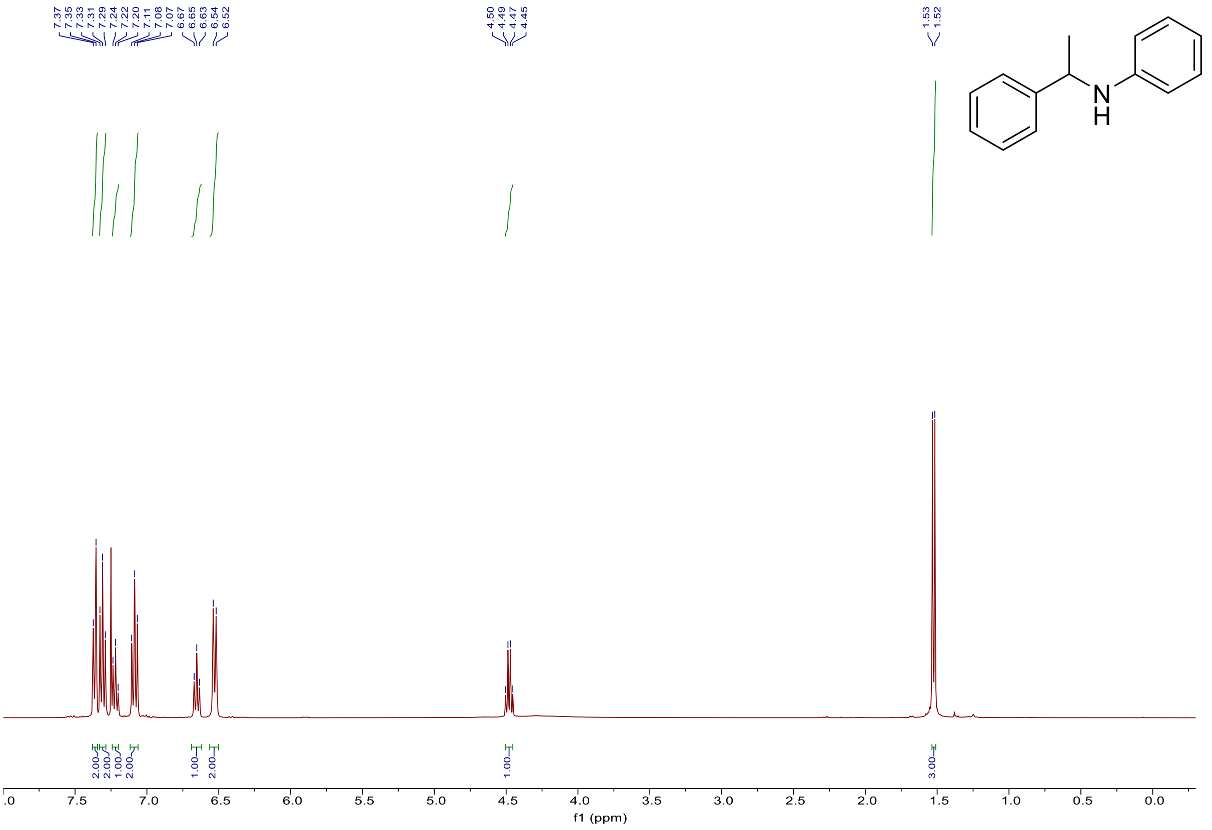

Figure S115 ^{1}H NMR of *N*-(1-Phenylethyl)aniline [S16] (4n)

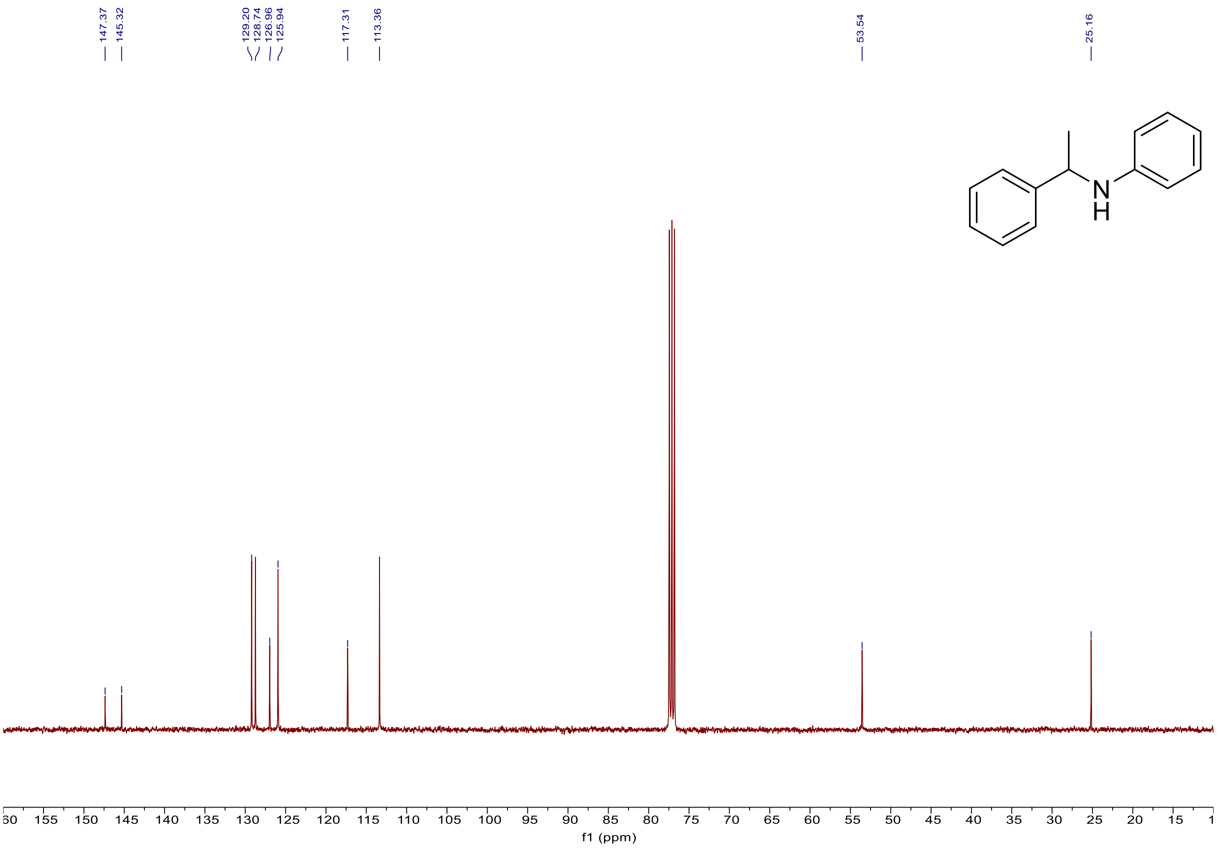

Figure S116 **[13]**C NMR of *N*-(1-Phenylethyl)aniline [S16] (4n)

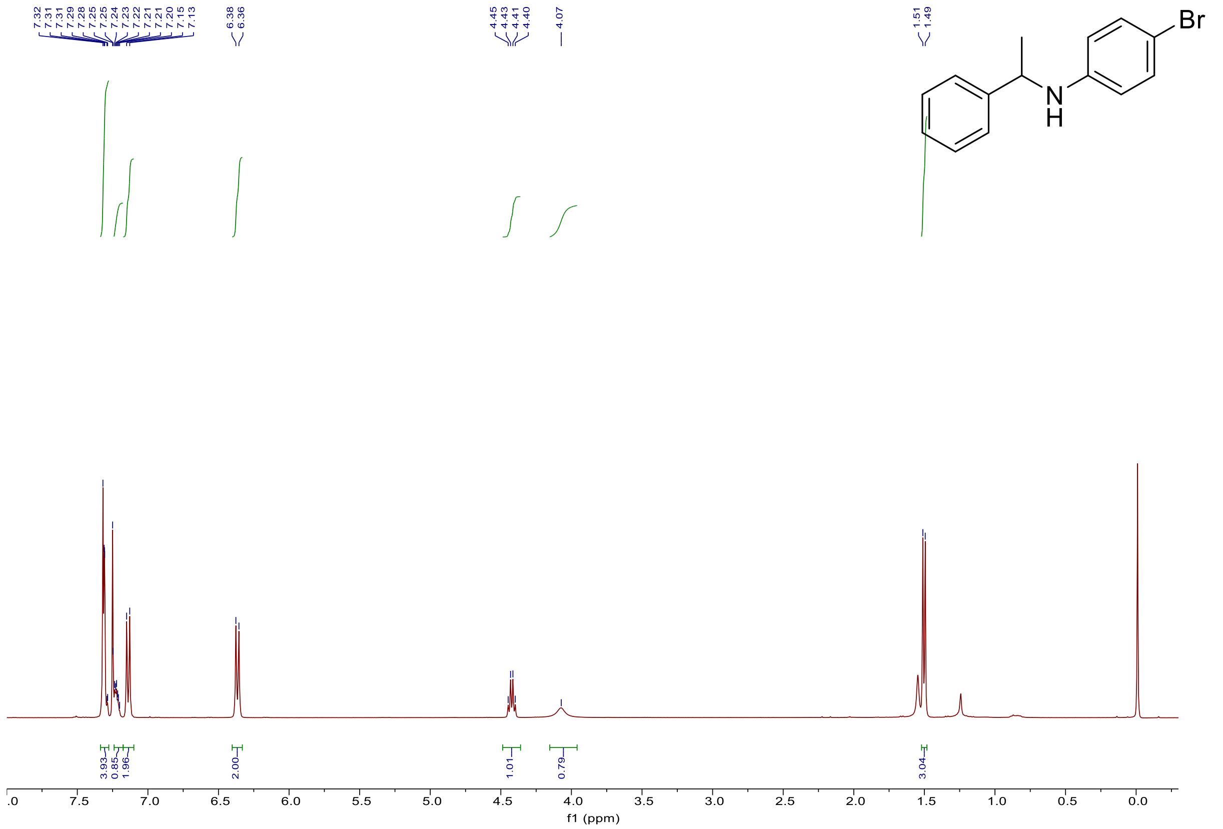

Figure S117 ^{1}H NMR of 4-Bromo-*N*-(1-phenylethyl)aniline [S16] (4o)

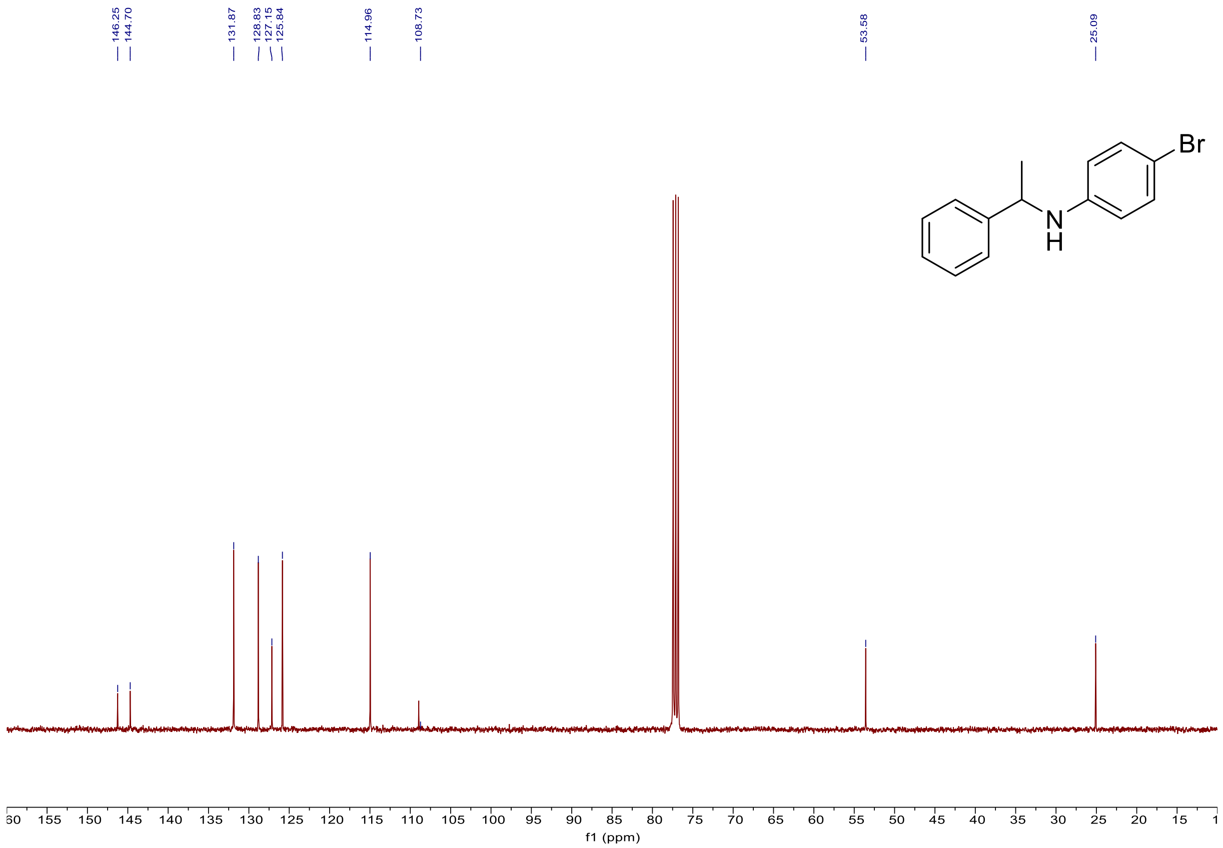

Figure S118 ^{13}C NMR of 4-Bromo-*N*-(1-phenylethyl)aniline [S16] (4o)

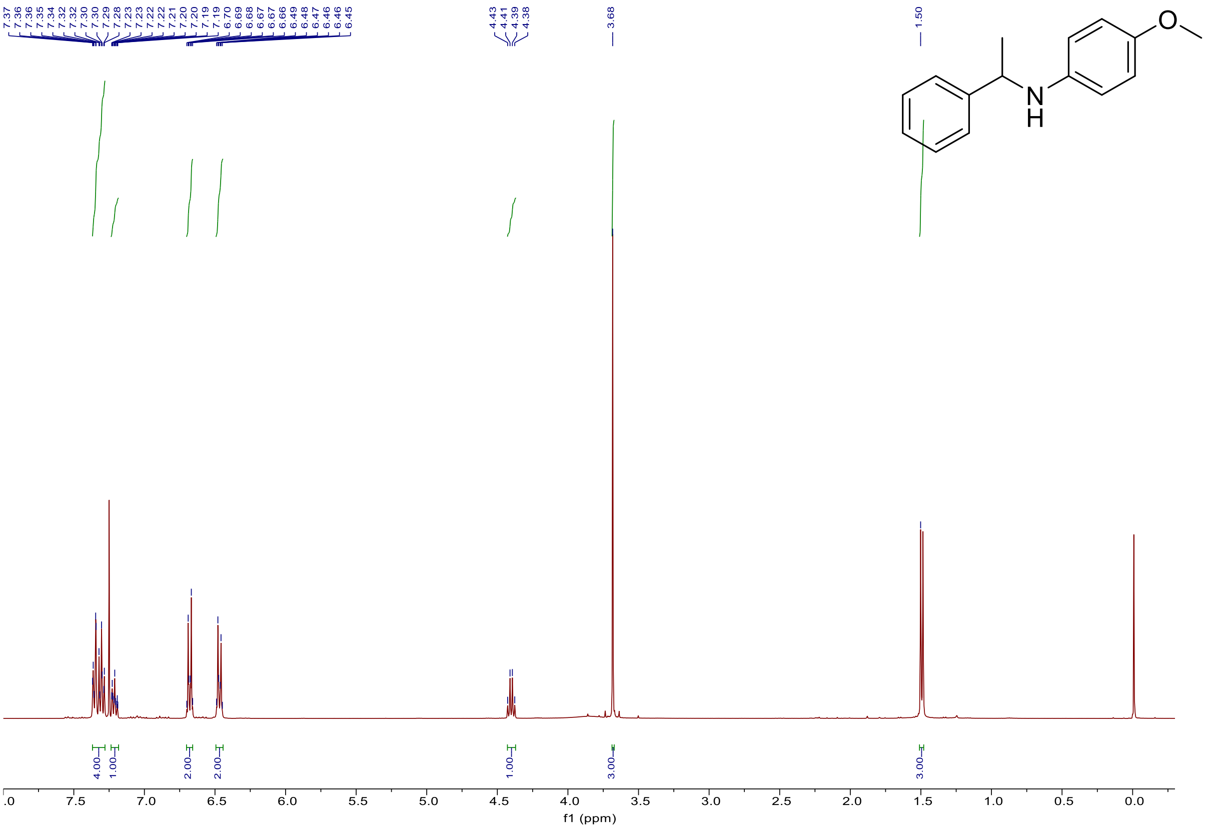

Figure S119 ¹H NMR of 4-Methoxy-*N*-(1-phenylethyl)aniline [S16] (4p)

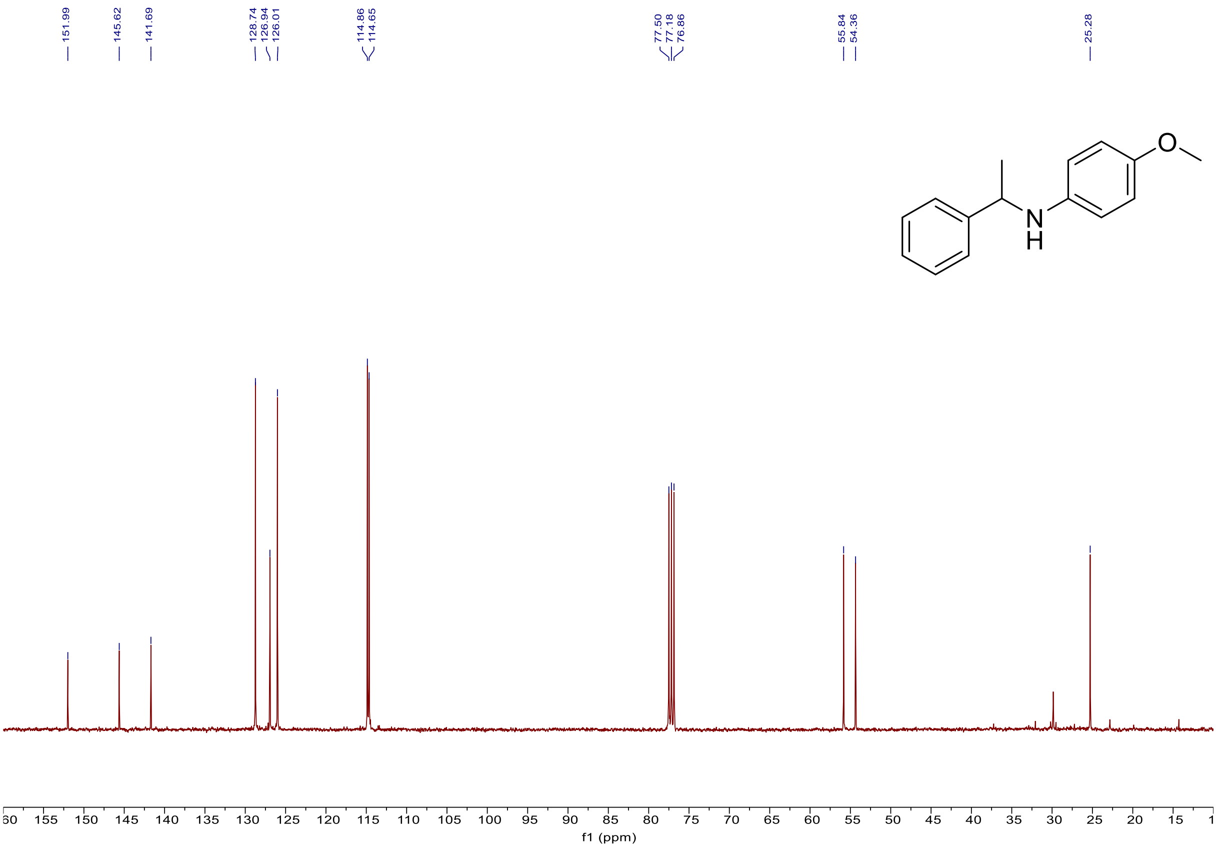

Figure S120 ¹³C NMR of 4-Methoxy-*N*-(1-phenylethyl)aniline [S16] (4p)

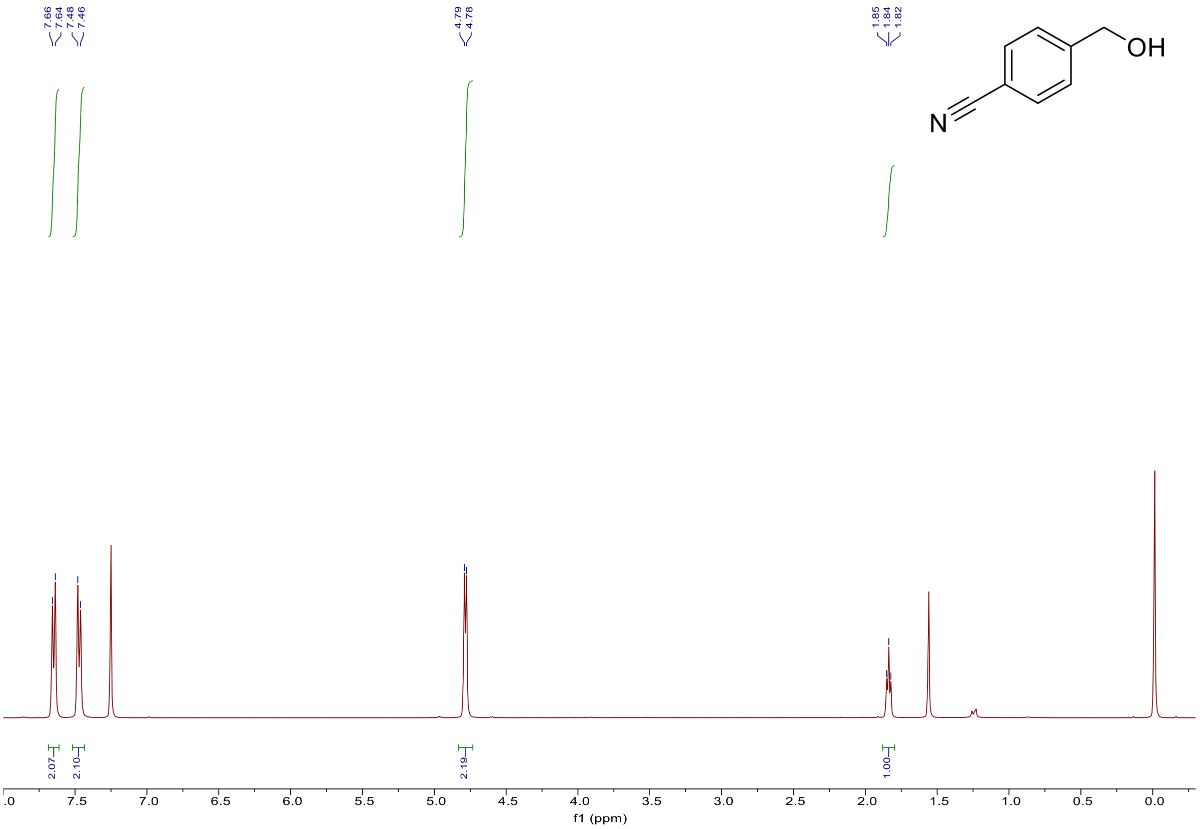

Figure S121 ^{1}H NMR of 4-(hydroxymethyl)benzonitrile [S17] (6a)

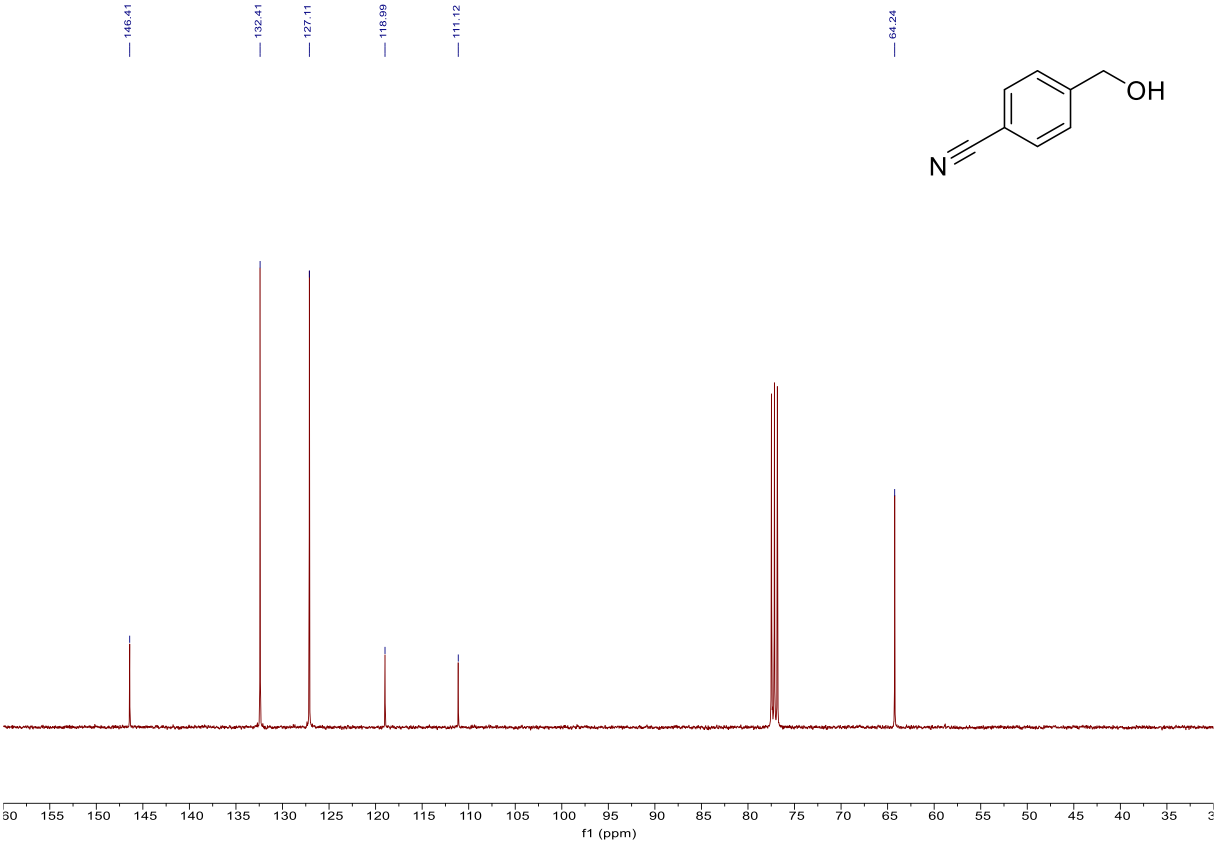

Figure S122 ^{13}C NMR of 4-(hydroxymethyl)benzonitrile [S17] (6a)

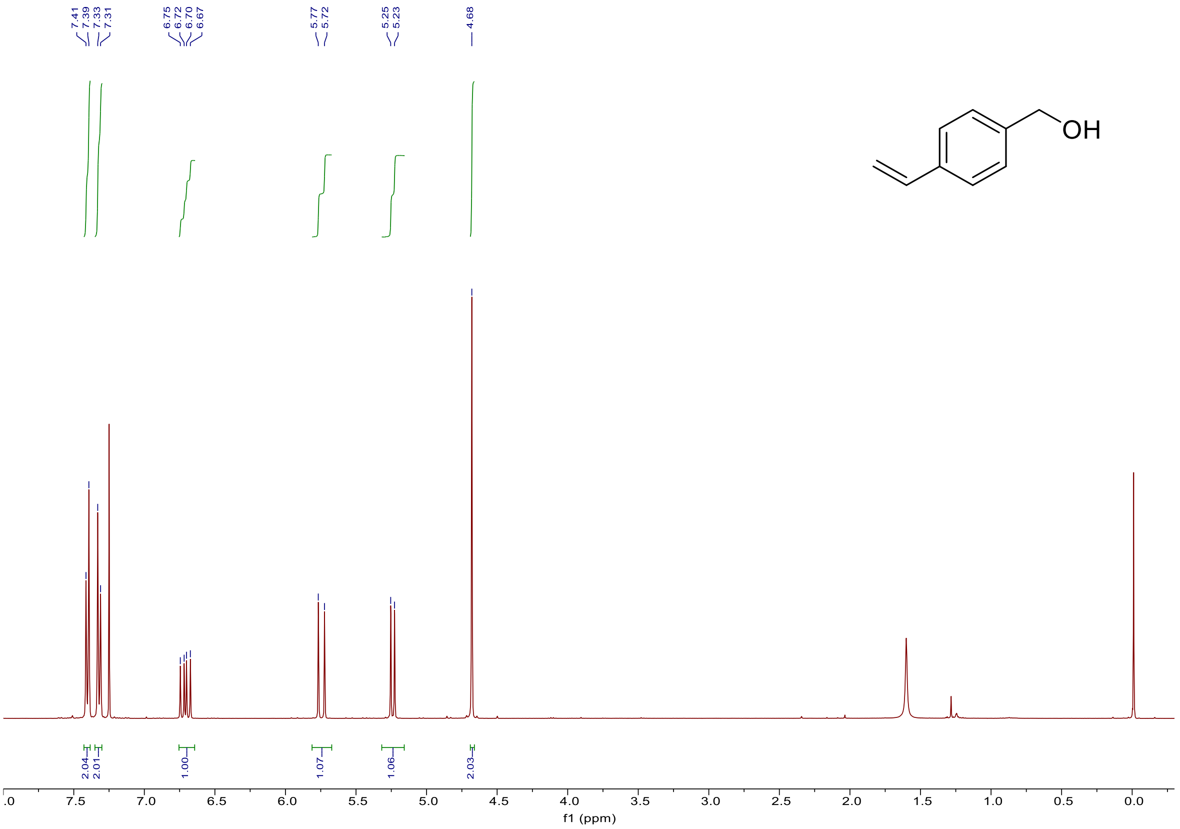

Figure S123 ^{1}H NMR of (4-vinylphenyl)methanol [S18] (6b)

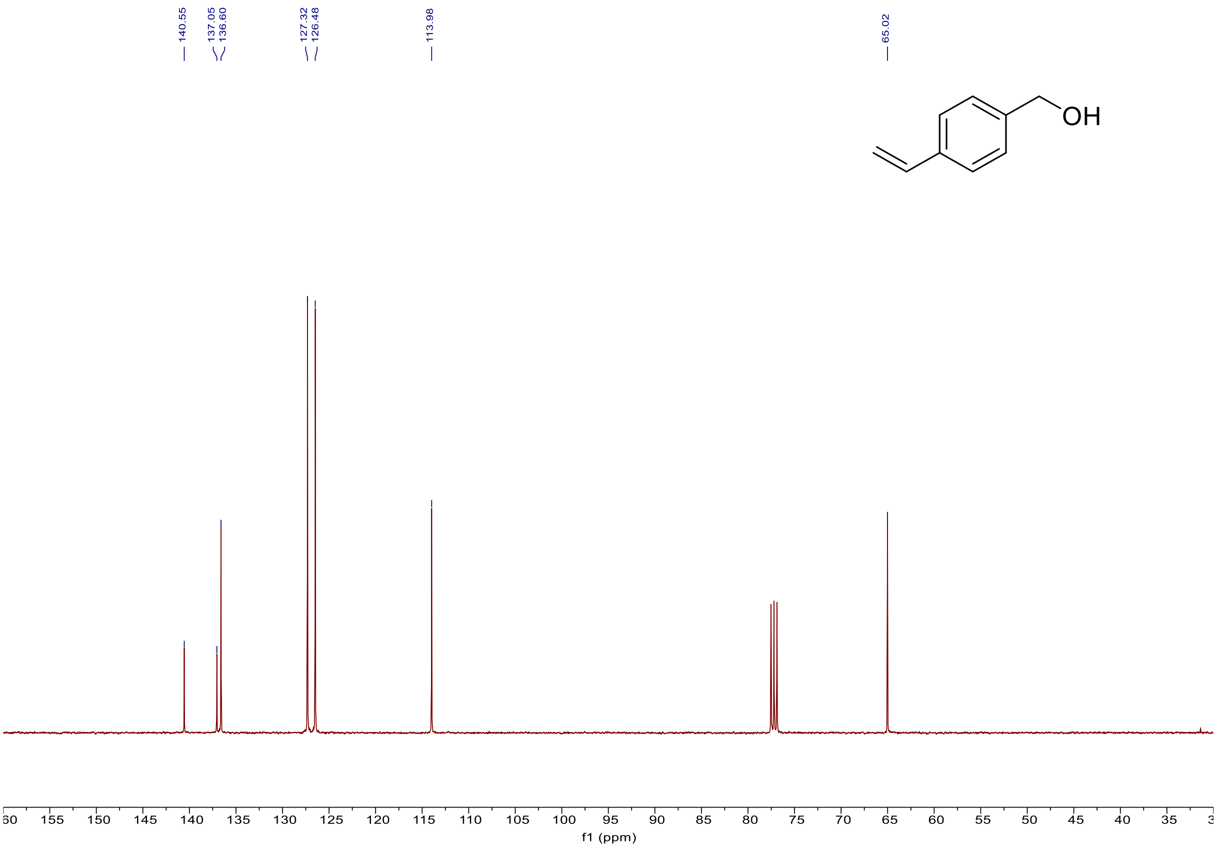

Figure S124 ^{13}C NMR of (4-vinylphenyl)methanol [S18] (6b)

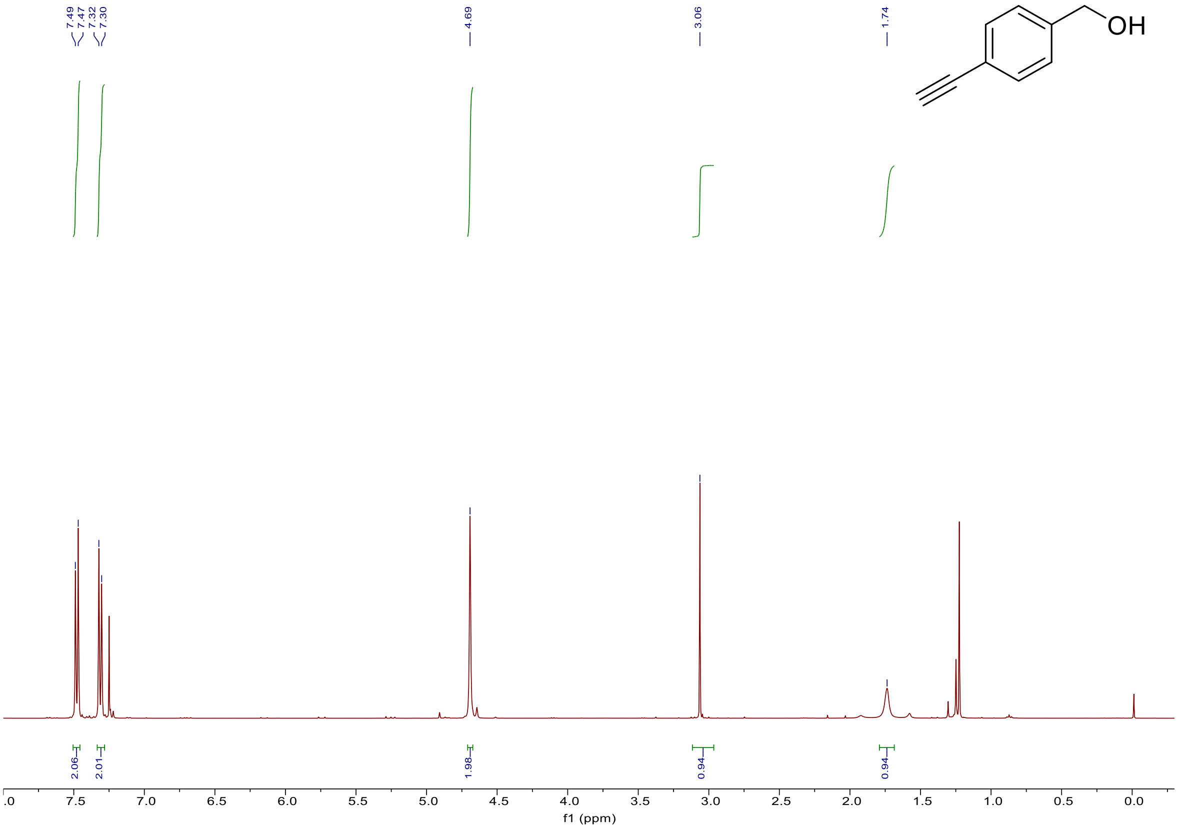

Figure S125 ^{1}H NMR of (4-ethynylphenyl)methanol [S19] (6c)

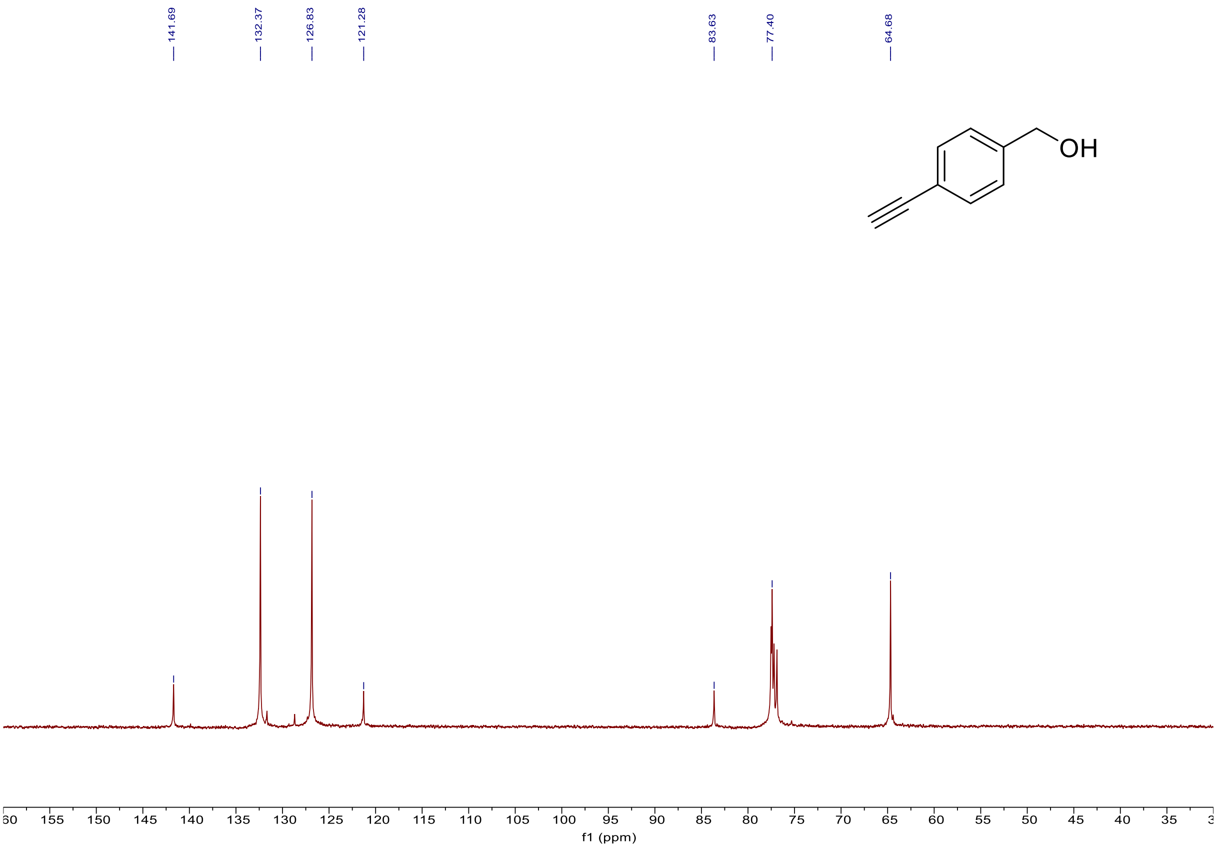

Figure S126 ^{13}C NMR of (4-ethynylphenyl)methanol [S19] (6c)

5. Calculation method

Geometry optimizations and energy calculations for all compounds were conducted in the gas phase at the M06-2X/6-31G(d,p) level of theory. Frequency calculations at the same level of theory were performed to confirm the optimized geometries with no imaginary frequency and transition states with one imaginary frequency and to evaluate the thermodynamic free energies. Intrinsic reaction coordinate (IRC) calculations were also employed to validate the transition states. All calculations were carried out using the Gaussian 16 programs.[S20]

HBpin

Atom	X	Y	Z
C	0.000000	0.781643	-0.185909
C	0.000000	-0.781643	-0.185909
O	0.345563	-1.089282	1.183730
O	-0.345563	1.089282	1.183730
B	0.000000	0.000000	1.933736
H	0.000000	0.000000	3.119573
C	1.385541	1.374929	-0.433526
H	1.697869	1.242328	-1.472913
H	1.354233	2.443742	-0.210382
H	2.130325	0.910290	0.218848
C	-1.023692	1.421393	-1.109470
H	-0.950044	2.509147	-1.037679
H	-0.835232	1.132064	-2.148228
H	-2.039597	1.129362	-0.840178
C	1.023692	-1.421393	-1.109470
H	0.950044	-2.509147	-1.037679
H	0.835232	-1.132064	-2.148228
H	2.039597	-1.129362	-0.840178
C	-1.385541	-1.374929	-0.433526
H	-1.697869	-1.242328	-1.472913
H	-1.354233	-2.443742	-0.210382
H	-2.130325	-0.910290	0.218848

MeMgCl

Atom	X	Y	Z
Mg	0.000000	0.000000	-0.464648
C	0.000000	0.000000	-2.532838
H	0.000000	1.019517	-2.927163

H	0.882927	-0.509758	-2.927163
H	-0.882927	-0.509758	-2.927163
Cl	0.000000	0.000000	1.738488

INT1

Atom	X	Y	Z
C	-1.907274	-0.394755	-0.103817
O	-1.690090	-0.925536	1.229118
C	-0.898444	0.795361	-0.163968
C	-1.433271	2.088830	0.435901
H	-0.608844	2.797896	0.543921
H	-2.193081	2.532405	-0.212236
H	-1.872323	1.912177	1.421481
C	-0.282491	1.040881	-1.531880
H	-1.066921	1.286004	-2.253958
H	0.409723	1.887854	-1.488664
H	0.247001	0.156550	-1.900878
C	-3.363493	0.017388	-0.231901
H	-3.537885	0.501534	-1.197686
H	-3.995326	-0.871471	-0.176126
H	-3.659158	0.699945	0.565963
C	-1.566208	-1.516951	-1.080065
H	-2.186561	-2.383147	-0.840546
H	-1.770701	-1.218495	-2.111738
H	-0.515819	-1.814354	-0.993688
B	-0.433963	-0.613388	1.607388
H	0.123972	-1.027525	2.563469
O	0.152306	0.325574	0.752295
Mg	2.136300	0.287811	0.162925
Cl	2.271730	-1.932350	-0.185533
C	3.005082	2.166181	-0.045608
H	3.233460	2.386970	-1.094893
H	2.340442	2.963380	0.309836
H	3.945693	2.271524	0.504481

TS1

Atom	X	Y	Z
C	1.693148	0.180501	-0.548297
O	1.607715	1.381212	0.230869

C	1.006168	-0.872942	0.375336
C	1.925276	-1.382037	1.479836
H	2.689169	-2.051516	1.076716
H	2.418201	-0.549019	1.987997
H	1.329645	-1.932698	2.211166
C	0.333051	-2.027860	-0.345123
H	-0.421665	-1.694393	-1.062617
H	1.082349	-2.613132	-0.886844
H	-0.157414	-2.684812	0.377588
C	3.160395	-0.109023	-0.827104
H	3.742156	-0.101856	0.095386
H	3.277298	-1.080660	-1.317260
H	3.562603	0.660747	-1.489584
C	0.949530	0.399996	-1.866934
H	-0.121801	0.582962	-1.724025
H	1.361801	1.287075	-2.352582
H	1.061442	-0.455179	-2.538123
B	0.499113	1.323922	1.051118
H	0.413984	1.985600	2.033972
O	-0.010066	-0.054621	1.031679
Mg	-1.770025	0.466889	0.299630
Cl	-3.402848	-0.883327	-0.326388
C	-1.182200	2.497462	0.107984
H	-0.259560	3.034235	-0.122095
H	-1.775208	2.594706	-0.820560
H	-1.703245	3.082913	0.873738

INT2

Atom	X	Y	Z
C	-1.963650	-0.160366	0.276836
O	-1.419012	0.931109	1.004063
C	-0.704447	-0.836626	-0.341573
C	-0.948654	-1.654128	-1.596863
H	-0.014238	-2.104818	-1.943823
H	-1.660999	-2.459780	-1.393375
H	-1.343407	-1.025477	-2.395619
C	0.068360	-1.655925	0.698939
H	-0.453668	-2.576269	0.969505
H	1.040298	-1.974636	0.289187
H	0.208776	-1.082172	1.625465
C	-2.898483	0.378563	-0.809646

H	-3.442939	-0.425248	-1.313912
H	-3.620666	1.047372	-0.336192
H	-2.340336	0.949374	-1.556864
C	-2.735487	-1.054265	1.238098
H	-3.622587	-0.522046	1.589087
H	-3.062159	-1.975418	0.744357
H	-2.130694	-1.308350	2.110522
B	-0.268495	1.434019	0.333233
H	0.778514	1.410895	1.204135
O	0.101249	0.318452	-0.685952
Mg	1.853314	0.332023	0.173861
Cl	3.926859	-0.368980	0.041686
C	-0.260721	2.884890	-0.322861
H	0.682793	3.112431	-0.836768
H	-1.066693	2.985684	-1.057584
H	-0.405294	3.657399	0.438246

PhCOOEt

Atom	X	Y	Z
C	2.304595	-1.575765	-0.000023
C	0.979421	-1.153988	-0.000027
C	0.690444	0.211653	-0.000003
C	1.722711	1.150654	0.000026
C	3.045161	0.724514	0.000031
C	3.335759	-0.638674	0.000007
H	2.534277	-2.636181	-0.000043
H	0.166810	-1.871427	-0.000051
H	1.464133	2.204207	0.000044
H	3.849744	1.452339	0.000053
H	4.368803	-0.971844	0.000011
C	-0.709690	0.725186	-0.000010
O	-1.004555	1.897515	-0.000036
O	-1.621594	-0.260931	0.000010
C	-2.988701	0.172275	-0.000019
H	-3.160748	0.796255	-0.881989
H	-3.160755	0.796342	0.881889
C	-3.852490	-1.069401	0.000039
H	-3.653967	-1.675699	0.886539
H	-4.908413	-0.789607	0.000022
H	-3.653961	-1.675785	-0.886400

INT3

Atom	X	Y	Z
C	2.981421	-0.354744	-0.784377
O	2.234987	-0.886475	0.319196
C	2.503037	1.142704	-0.829905
C	1.294541	1.349627	-1.748556
H	0.922225	2.367474	-1.602881
H	1.561571	1.227907	-2.802777
H	0.481867	0.657664	-1.513688
C	3.604914	2.111969	-1.253696
H	3.985703	1.868725	-2.251542
H	3.197487	3.125759	-1.280571
H	4.432298	2.099923	-0.543209
C	2.655033	-1.169669	-2.028191
H	3.145916	-0.744346	-2.908952
H	3.011861	-2.195244	-1.900101
H	1.577968	-1.196947	-2.219312
C	4.463857	-0.503293	-0.444629
H	4.669731	-1.553976	-0.226403
H	5.094778	-0.187272	-1.279792
H	4.725789	0.086608	0.435197
B	2.015062	0.260365	1.331511
O	2.129935	1.443007	0.506654
Mg	0.444600	-1.523247	0.699317
Cl	-0.273191	-3.539039	1.341506
H	0.781900	0.050065	1.718090
C	2.904608	0.209062	2.659192
H	2.567216	0.971867	3.369593
H	2.823808	-0.759717	3.165938
H	3.966004	0.398536	2.466910
O	-1.019472	-0.712480	-0.347267
C	-2.135531	-0.177453	-0.307749
O	-3.219894	-0.845853	-0.609990
C	-3.066377	-2.255092	-0.934115
H	-2.456857	-2.323485	-1.839258
H	-2.526258	-2.740227	-0.116843
C	-2.345449	1.236211	0.053382
C	-3.626458	1.799013	0.002234
C	-1.243684	2.001457	0.451004

C	-3.800224	3.132568	0.343665
H	-4.468921	1.189558	-0.304127
C	-1.430650	3.335915	0.792474
H	-0.248470	1.569382	0.493148
C	-2.703053	3.899533	0.737768
H	-4.789081	3.576155	0.304607
H	-0.576364	3.928434	1.101059
H	-2.843969	4.942098	1.004789
C	-4.456355	-2.811824	-1.130912
H	-4.974329	-2.295486	-1.941848
H	-4.386828	-3.872337	-1.381239
H	-5.043537	-2.711814	-0.216030

TS2

Atom	X	Y	Z
C	-2.450824	-1.752042	-0.705300
O	-1.626659	-2.375234	0.253022
C	-2.891140	-0.442771	0.020044
C	-3.961913	-0.699753	1.076109
H	-3.673018	-1.548871	1.699959
H	-4.060933	0.185501	1.710571
H	-4.932860	-0.906022	0.617041
C	-3.305001	0.694364	-0.903539
H	-4.152457	0.392221	-1.526623
H	-3.611802	1.573664	-0.326828
H	-2.487852	0.986916	-1.572619
C	-3.610050	-2.683951	-1.036458
H	-4.097446	-3.032003	-0.124492
H	-4.350543	-2.188219	-1.673319
H	-3.228492	-3.557852	-1.570607
C	-1.641880	-1.459725	-1.976474
H	-1.173223	-2.393122	-2.299316
H	-2.274134	-1.087356	-2.788560
H	-0.850223	-0.730326	-1.778780
B	-0.827883	-1.385085	0.925299
O	-1.680555	-0.078192	0.717641
Mg	-0.761835	1.599636	0.626008
Cl	-1.286106	3.733505	0.305076
C	2.619765	-0.370619	0.149824
C	2.789545	-1.195845	-0.965829

C	3.612413	-0.269141	1.125301
C	3.970606	-1.908711	-1.110409
C	4.795243	-0.984325	0.969900
H	3.442966	0.365570	1.988741
C	4.974104	-1.797723	-0.145909
H	4.107630	-2.561705	-1.965275
H	5.573941	-0.910667	1.721067
H	5.895652	-2.358739	-0.262013
C	-0.505332	-1.684209	2.463140
H	-1.428461	-1.745344	3.051831
H	0.009138	-2.645998	2.559932
H	0.135253	-0.924612	2.924587
O	1.036749	0.952183	1.385702
C	1.387260	0.414080	0.329214
H	0.259493	-1.133402	0.304846
H	1.976115	-1.302601	-1.678311
O	0.713925	0.878814	-0.793762
C	1.475042	1.676875	-1.750782
H	0.714759	2.004209	-2.460967
H	2.177771	1.011252	-2.257130
C	2.170992	2.848224	-1.090027
H	2.928812	2.509160	-0.377850
H	2.673881	3.439855	-1.859004
H	1.450620	3.489759	-0.575439

INT4

Atom	X	Y	Z
C	-2.804396	-0.348130	0.875655
O	-2.043211	-0.020915	2.065639
C	-2.363184	0.764250	-0.116747
C	-3.072797	2.095765	0.092869
H	-2.538068	2.872515	-0.460325
H	-4.100928	2.044351	-0.274527
H	-3.092580	2.369835	1.151528
C	-2.365548	0.364540	-1.582693
H	-3.377401	0.076276	-1.882533
H	-2.077925	1.221635	-2.204521
H	-1.681119	-0.464531	-1.781173
C	-4.282728	-0.326475	1.219321
H	-4.884905	-0.474354	0.317665

H	-4.504863	-1.137589	1.916233
H	-4.569160	0.616970	1.686323
C	-2.351981	-1.735530	0.429786
H	-2.497445	-2.430938	1.260279
H	-2.936709	-2.088209	-0.424974
H	-1.289841	-1.735697	0.157217
B	-0.914147	0.630492	1.687650
O	-0.972030	0.971101	0.325518
Mg	0.443186	1.167757	-1.098642
Cl	0.415895	3.295708	-1.775032
C	2.398264	-0.637616	-0.152822
C	2.917293	0.634301	-0.437026
C	2.629668	-1.190638	1.109668
C	3.631524	1.351492	0.526460
H	2.831980	1.040801	-1.446162
C	3.357937	-0.483356	2.061908
H	2.231078	-2.174733	1.326727
C	3.853890	0.789832	1.779217
H	4.021142	2.334809	0.284085
H	3.531062	-0.922927	3.039675
H	4.412179	1.337421	2.531264
C	0.280899	1.031981	2.601119
H	-0.026350	1.864207	3.243840
H	0.577900	0.208748	3.253974
H	1.156176	1.359259	2.032504
O	0.741857	-0.511811	-1.885206
O	0.915342	-2.422946	-0.583295
C	0.234659	-3.265465	-1.492892
H	-0.598230	-2.721385	-1.958135
H	0.919544	-3.562570	-2.302174
C	-0.257176	-4.482436	-0.734754
H	-0.787943	-5.163353	-1.404841
H	0.584560	-5.016765	-0.287938
H	-0.937476	-4.186361	0.068184
C	1.590822	-1.356544	-1.236949
H	2.299060	-1.819259	-1.955163

PhCHO

Atom	X	Y	Z
C	1.732653	1.056053	0.000023

C	0.361260	1.290648	-0.000012
C	-0.528492	0.216948	-0.000031
C	-0.050589	-1.096196	-0.000027
C	1.317502	-1.329330	-0.000008
C	2.207442	-0.253348	0.000020
H	2.429038	1.887880	0.000048
H	-0.024674	2.307341	-0.000023
H	-0.770260	-1.908740	-0.000042
H	1.696868	-2.345862	-0.000008
H	3.276973	-0.438485	0.000036
C	-1.989446	0.471181	-0.000039
H	-2.277329	1.544282	-0.000032
O	-2.829074	-0.397769	0.000058

EtOBpin

Atom	X	Y	Z
C	1.162603	0.823254	0.027606
O	-0.243416	0.851943	0.345803
C	1.458381	-0.713321	-0.018367
C	1.734809	-1.297342	1.366116
H	1.715234	-2.387209	1.296191
H	2.711928	-0.986721	1.746114
H	0.966248	-0.985522	2.079513
C	2.548922	-1.122514	-0.994812
H	3.498572	-0.645230	-0.732651
H	2.686653	-2.205732	-0.954333
H	2.285995	-0.850461	-2.017999
C	1.928173	1.589826	1.093290
H	3.006704	1.501152	0.928692
H	1.660539	2.648343	1.046255
H	1.691771	1.221523	2.092550
C	1.327406	1.492390	-1.335621
H	0.901924	2.497493	-1.287588
H	2.380374	1.570837	-1.619464
H	0.797323	0.929834	-2.109738
B	-0.761895	-0.349116	-0.092759
O	0.201720	-1.255217	-0.465323
O	-2.084692	-0.622004	-0.156333
C	-3.006918	0.403227	0.200642
H	-2.873041	0.666007	1.256626

H	-2.803397	1.305378	-0.387796
C	-4.408402	-0.110080	-0.055884
H	-5.149123	0.648014	0.210332
H	-4.530384	-0.364715	-1.111054
H	-4.596573	-1.007820	0.537151

INT5

Atom	X	Y	Z
C	-0.580541	-1.891467	-0.271423
O	-0.341941	-1.180508	0.935254
C	-1.785917	-1.115953	-0.970728
C	-2.950533	-2.009726	-1.386286
H	-3.743562	-1.390358	-1.812389
H	-2.637399	-2.738535	-2.139879
H	-3.362532	-2.543747	-0.528843
C	-1.373819	-0.251162	-2.163560
H	-1.038103	-0.857944	-3.008672
H	-2.239042	0.334672	-2.490656
H	-0.559490	0.437243	-1.910207
C	-0.904315	-3.351142	0.071282
H	-1.067672	-3.953917	-0.826703
H	-0.052541	-3.766284	0.615617
H	-1.780561	-3.434753	0.714019
C	0.707310	-1.877326	-1.093674
H	1.504424	-2.344198	-0.508439
H	0.587558	-2.442106	-2.023516
H	1.024703	-0.863059	-1.342560
B	-1.588155	-0.568601	1.389900
O	-2.257999	-0.219059	0.050299
Mg	-1.600105	1.614131	0.279435
Cl	-2.575629	3.566803	-0.085021
C	2.554595	0.681046	0.417177
C	3.311019	-0.130166	1.267438
C	3.089189	1.152058	-0.788523
C	4.609016	-0.475792	0.910745
H	2.868790	-0.499039	2.188834
C	4.387451	0.811347	-1.135008
H	2.475434	1.778605	-1.428085
C	5.142741	-0.002231	-0.286250
H	5.202808	-1.110582	1.558937

H	4.816938	1.173419	-2.062634
H	6.157601	-0.269121	-0.563439
C	-2.535083	-1.348778	2.417038
H	-3.356398	-0.709457	2.757734
H	-2.992070	-2.243573	1.979864
H	-1.978649	-1.667576	3.304934
O	0.404452	1.593403	0.045607
C	1.182139	0.996141	0.796673
H	0.855325	0.695380	1.799521
H	-1.313425	0.580937	1.910489

TS3

Atom	X	Y	Z
C	-0.580541	-1.891467	-0.271423
O	-0.341941	-1.180508	0.935254
C	-1.785917	-1.115953	-0.970728
C	-2.950533	-2.009726	-1.386286
H	-3.743562	-1.390358	-1.812389
H	-2.637399	-2.738535	-2.139879
H	-3.362532	-2.543747	-0.528843
C	-1.373819	-0.251162	-2.163560
H	-1.038103	-0.857944	-3.008672
H	-2.239042	0.334672	-2.490656
H	-0.559490	0.437243	-1.910207
C	-0.904315	-3.351142	0.071282
H	-1.067672	-3.953917	-0.826703
H	-0.052541	-3.766284	0.615617
H	-1.780561	-3.434753	0.714019
C	0.707310	-1.877326	-1.093674
H	1.504424	-2.344198	-0.508439
H	0.587558	-2.442106	-2.023516
H	1.024703	-0.863059	-1.342560
B	-1.588155	-0.568601	1.389900
O	-2.257999	-0.219059	0.050299
Mg	-1.600105	1.614131	0.279435
Cl	-2.575629	3.566803	-0.085021
C	2.554595	0.681046	0.417177
C	3.311019	-0.130166	1.267438
C	3.089189	1.152058	-0.788523
C	4.609016	-0.475792	0.910745

H	2.868790	-0.499039	2.188834
C	4.387451	0.811347	-1.135008
H	2.475434	1.778605	-1.428085
C	5.142741	-0.002231	-0.286250
H	5.202808	-1.110582	1.558937
H	4.816938	1.173419	-2.062634
H	6.157601	-0.269121	-0.563439
C	-2.535083	-1.348778	2.417038
H	-3.356398	-0.709457	2.757734
H	-2.992070	-2.243573	1.979864
H	-1.978649	-1.667576	3.304934
O	0.404452	1.593403	0.045607
C	1.182139	0.996141	0.796673
H	0.855325	0.695380	1.799521
H	-1.313425	0.580937	1.910489

INT6

Atom	X	Y	Z
C	-2.555715	-0.974403	-0.123349
O	-2.737341	-0.423227	1.200499
C	-1.666486	0.097379	-0.824888
C	-2.458065	1.264218	-1.401166
H	-1.764397	2.059130	-1.686901
H	-3.011356	0.948643	-2.289298
H	-3.169216	1.657398	-0.668656
C	-0.677651	-0.456041	-1.834492
H	-1.217399	-0.993183	-2.620940
H	-0.122128	0.361147	-2.304571
H	0.028155	-1.145913	-1.363929
C	-3.919479	-1.166903	-0.764791
H	-3.809381	-1.495055	-1.802894
H	-4.471023	-1.935301	-0.218553
H	-4.502591	-0.245507	-0.744628
C	-1.846373	-2.314658	0.051068
H	-2.441533	-2.939600	0.720562
H	-1.732394	-2.832608	-0.904970
H	-0.853858	-2.178213	0.490425
B	-1.703368	0.409480	1.483860
O	-0.919094	0.627870	0.329552
Mg	0.862311	1.589408	0.240676
Cl	0.946159	2.891607	-1.577684

C	2.274590	-0.769779	0.919629
C	1.850182	-2.100217	0.852567
C	2.837144	-0.194769	-0.229225
C	1.985337	-2.835134	-0.323070
H	1.411579	-2.561172	1.734745
C	2.960142	-0.922452	-1.413978
H	3.230112	0.820793	-0.179094
C	2.535629	-2.246934	-1.462428
H	1.648550	-3.867150	-0.355188
H	3.395625	-0.452366	-2.289913
H	2.630911	-2.818476	-2.379766
C	-1.407342	1.090332	2.850945
H	-0.332165	1.236856	2.998295
H	-1.870969	2.084052	2.866001
H	-1.833052	0.520815	3.678702
O	1.686720	1.367188	1.897418
C	2.111413	0.077895	2.179546
H	3.080146	0.089615	2.708127
H	1.408562	-0.457370	2.847707

$PhCH_2OBpin$

Atom	X	Y	Z
C	2.799096	0.780853	0.008280
O	1.380329	0.958401	0.204884
C	2.950563	-0.775418	0.089086
C	3.064529	-1.278804	1.526862
H	2.948285	-2.364856	1.527478
H	4.034912	-1.028888	1.964344
H	2.276947	-0.849291	2.153001
C	4.067158	-1.351026	-0.766188
H	5.035211	-0.942031	-0.459821
H	4.098575	-2.436179	-0.642380
H	3.908621	-1.132777	-1.823165
C	3.548425	1.552190	1.081786
H	4.623176	1.356249	1.013929
H	3.386483	2.623977	0.943052
H	3.202037	1.280136	2.079788
C	3.130183	1.330931	-1.377475
H	2.800566	2.371086	-1.431127
H	4.204406	1.293106	-1.577267

H	2.609745	0.763430	-2.154511
B	0.788582	-0.221543	-0.190286
O	1.684252	-1.234771	-0.422646
C	-2.834185	0.302327	-0.054240
C	-3.822316	1.273304	0.124045
C	-3.202483	-1.037224	-0.145930
C	-5.160988	0.911063	0.210801
H	-3.538926	2.321194	0.195620
C	-4.545694	-1.400067	-0.058787
H	-2.432263	-1.786659	-0.286286
C	-5.526987	-0.431005	0.119451
H	-5.919786	1.674688	0.349553
H	-4.823826	-2.446877	-0.131398
H	-6.571803	-0.716542	0.186731
O	-0.547969	-0.383210	-0.337776
C	-1.391792	0.738111	-0.142471
H	-1.103922	1.271003	0.772410
H	-1.267293	1.442177	-0.975532

6. References

S1) Bhattacharya, P.; Krause, J. A.; Guan, H. Iron Hydride Complexes Bearing Phosphinite-Based Pincer Ligands: Synthesis, Reactivity, and Catalytic Application in Hydrosilylation Reactions. *Organometallics* **2011**, *30*, 4720-4729.

S2) Kovalenko, O. O.; Adolfsson, H. Highly Efficient and Chemoselective Zinc-Catalyzed Hydrosilylation of Esters under Mild Conditions. *Chem. - Eur. J.* **2015**, *21*, 2785-2788.

S3) Zhang, J.; Gao, X.; Zhang, C.; Ma, J.; Zhao, D. Highly Efficient System for Reduction of Carboxylic Acids and Their Derivatives to Alcohols by HfCl4/KBH4. *Synth. Commun.* **2009**, *39*, 1640-1654.

S4) Kim, H.; Shin, H. L.; Yi, J.; Choi, H. S.; Lee, J. H.; Hwang, H.; An, D. K. Lithium Bromide/HBpin: A Mild and Effective Catalytic System for the Selective Hydroboration of Aldehydes and Ketones. *Bull. Korean Chem. Soc.* **2020**, *41*, 1009-1018.

S5) Cano, I.; Martínez-Prieto, L. M.; Vendier, L.; van Leeuwen, P. W. N. M. An iridium–SPO complex as bifunctional catalyst for the highly selective hydrogenation of aldehydes. *Catal. Sci. Technol.* **2018**, *8*, 221-228.

S6) Barry, C. N.; Evans, S. A.**,** Jr. Triphenylphosphine-tetrachloromethane-promoted chlorination and cyclodehydration of simple diols**.** *J. Org. Chem.* **1981**, *46*, 3361–3364**.**

S7) Chen, X.; Zhang, Y.; Wan, H.; Wang, W.; Zhang, S. Stereoselective organocatalytic oxidation of alcohols to enals: a homologation method to prepare polyenes. *Chem. Commun.* **2016**, *52*, 3532-3535.

S8) Avuluri, S.; Bujaranipalli, S.; Das, S.; Yadav, J. S. Stereoselective synthesis of 5′-hydroxyzearalenone. *Tetrahedron lett.* **2018**, *59*, 3547-3549.

S9) Seok, J. E.; Kim, H. T.; Kim, J.; Lee, J. H.; Jaladi, A. K.; Hwang, H.; An, D. K. Effective Magnesium-catalyzed Hydroboration of Nitriles and Imines. *Asian J. Org. Chem.* **2022**, *11*, e2022004.

S10) Ghosh, P.; Jacobi von Wangelin, A. Manganese-Catalyzed Hydroborations with Broad Scope. *Chem., Int. Ed.* **2021**, *60*, 16035-16043.

S11) Huang, Z.; Wang, S.; Zhu, X.; Yuan, Q.; Wei, Y.; Zhou, S.; Mu, X. Well-Defined Amidate-Functionalized N-Heterocyclic Carbene -Supported Rare-Earth Metal Complexes as Catalysts for Efficient Hydroboration of Unactivated Imines and Nitriles. *Inorg. Chem.* **2018**, *57*, 15069-15078.

S12) Das, S.; Bhattacharjee, J.; Panda, T. K. An imidazolin-2-iminato ligand organozinc complex as a catalyst for hydroboration of organic nitriles. *New J. Chem.* **2019**, *43*, 16812-16818.

S13) Saha, S.; Eisen, M. S. Catalytic Recycling of a Th–H Bond via Single or Double Hydroboration of Inactivated Imines or Nitriles. *ACS Catal*. **2019**, *9*, 5947-5956.

S14) Thenarukandiyil, R.; Satheesh, V.; Shimon, L. J. W.; Ruiter, G. Hydroboration of Nitriles, Esters, and Carbonates Catalyzed by Simple Earth-Abundant Metal Triflate Salts. *Asian J. Org. Chem.* **2021**, *16*, 999-1006.

S15) Kaithal, A.; Chatterjee, B.; Gunanathan, C. Ruthenium-Catalyzed Selective Hydroboration of Nitriles and Imines. *J. Org. Chem.* **2016**, *81*, 11153-11161.

S16) Kim, H.; Kim, H. T.; Lee, J. H.; Hwang, H.; An, D. K. Lithium bromide: an inexpensive and efficient catalyst for imine hydroboration with pinacolborane at room temperature. *RSC Adv.* **2020**, *10*, 34421-34427.

S17) Boobalan, R.; Liu, K.; Chao, J.; Chen, C. Synthesis and biological assay of erlotinib analogues and BSA-conjugated erlotinib analogue. *Bioorg. Med. Chem.* **2017**, *27*, 1784-1788.

S18) Duan, Y.; Du, X.; Cui, Z.; Zeng, Y.; Liu, Y.; Yang, T.; Wen, J.; Zhang, X. Homogeneous Hydrogenation with a Cobalt/Tetraphosphine Catalyst: A Superior Hydride Donor for Polar Double Bonds and N-Heteroarenes. *J. Am. Chem. Soc.* **2019**, *141*, 20424-20433.

S19) Vestberg, R.; Westlund, R.; Eriksson, A.; Lopes, C.; Carlsson, M.; Eliasson, B.; Glimsdal, E.; Lindgren, M.; Malmström, E. Dendron Decorated Platinum (II) Acetylides for Optical Power Limiting. *Macromolecules* **2006**, *39*, 2238-2246.

S20) Gaussian 16, Revision C.01, M. J. Frisch, G. W. Trucks, H. B. Schlegel, G. E. Scuseria, M. A. Robb, J. R. Cheeseman, G. Scalmani, V. Barone, G. A. Petersson, H. Nakatsuji, X. Li, M. Caricato, A. V. Marenich, J. Bloino, B. G. Janesko, R. Gomperts, B. Mennucci, H. P. Hratchian, J. V. Ortiz, A. F. Izmaylov, J. L. Sonnenberg, D. Williams-Young, F. Ding, F. Lipparini, F. Egidi, J. Goings, B. Peng, A. Petrone, T. Henderson, D. Ranasinghe, V. G. Zakrzewski, J. Gao, N. Rega, G. Zheng, W. Liang, M. Hada, M. Ehara, K. Toyota, R. Fukuda, J. Hasegawa, M. Ishida, T. Nakajima, Y. Honda, O. Kitao, H. Nakai, T. Vreven, K. Throssell, J. A. Montgomery, Jr., J. E. Peralta, F. Ogliaro, M. J. Bearpark, J. J. Heyd, E. N. Brothers, K. N. Kudin, V. N. Staroverov, T. A. Keith, R. Kobayashi, J. Normand, K. Raghavachari, A. P. Rendell, J. C. Burant, S. S. Iyengar, J. Tomasi, M. Cossi, J. M. Millam, M. Klene, C. Adamo, R. Cammi, J. W. Ochterski, R. L. Martin, K. Morokuma, O. Farkas, J. B. Foresman, and D. J. Fox, Gaussian, Inc., Wallingford CT, **2019**.
